# Novel *Laccaria* Species From Juglandaceae Forest in Panama With Notes on Their Ecology

**DOI:** 10.3389/fmicb.2020.01597

**Published:** 2020-07-17

**Authors:** Adriana Corrales, Andrew W. Wilson, Gregory M. Mueller, Clark Ovrebo

**Affiliations:** ^1^Department of Biology, Faculty of Natural Sciences, Universidad del Rosario, Bogotá, Colombia; ^2^Research and Conservation, Denver Botanic Gardens, Denver, CO, United States; ^3^Negaunee Institute for Plant Conservation Science and Action, Chicago Botanic Garden, Glencoe, IL, United States; ^4^Department of Biology, University of Central Oklahoma, Edmond, OK, United States

**Keywords:** agaricomycetes, Central America, *Laccaria*, mycorrhiza, neotropical fungi, systematics, taxonomy

## Abstract

Since 2013 there have been 22 new species of *Laccaria* described worldwide. Only three of these represent species from the neotropics. In Panama, *Laccaria* is abundant in monodominant *Oreomunnea mexicana* (Juglandaceae) forests based on sporocarps and environmental sequencing of roots. This study uses a combination of morphological and phylogenetic evidence to document up to seven species of *Laccaria* from these forests, one previously described, three described as new, and three requiring more data before being formally described. Molecular data used for phylogenetic analysis include the nuclear ribosomal ITS and 28S regions, along with *TEF1* and *RPB2*. *Laccaria stellata*, has previously been reported from *O. mexicana* cloud forests of Panama. *Laccaria dallingii* sp. nov., *L. nitrophila* sp. nov., and *L. fortunensis* sp. nov. are described as new based on morphology and phylogenetic analysis of multiple collections. A taxon referred to as “PAN sp3” is resolved sister to *L. stellata.* Phylogenetic analysis also resolved two separate clades of Panamanian *Laccaria* as sister to *L. roseoalbescens*, a species previously described from Mexico. These three taxa are not described in this paper as there is too little material from which to make effective morphological descriptions even though their placement in phylogenetic analysis identify them as being unique. Ecologically, all described species except for *L. fortunensis* were amplified from *O. mexicana* ectomycorrhizal root tips. *L. nitrophila* was one of the most recovered species from the roots of *O. mexicana* in a previous study, and it has been shown to respond positively to long term nitrogen addition. Our results expand the knowledge of *Laccaria* diversity for Central America and highlight that at least some species of *Laccaria* are nitrophilic in neotropical Juglandaceae forests as well as in temperate forests.

## Introduction

*Laccaria* (Agaricales) is considered a model genus for understanding ectomycorrhizal (ECM) ecology and evolution (Martin et al., 2008; Wilson et al., 2017a). In a little more than a decade, studies incorporating morphological and molecular data have described 22 *Laccaria* species worldwide: one from New Zealand (Wilson et al., 2017a), 18 from Asia (Wilson et al., 2013; Popa et al., 2014; Vincenot et al., 2017; Cho et al., 2018; Latha et al., 2019; Li, 2020), and three from subtropical and tropical North and Central America (Montoya et al., 2015; Popa et al., 2016; Ramos et al., 2017).

The relatively low number of newly described *Laccaria* species in the Neotropics compared to Asia may be indicative of the difficulty of morphological species recognition within *Laccaria*, as cryptic species are considered the norm (Vincenot et al., 2017; Wilson et al., 2017b). Thus, the use of DNA sequence data is seen as critical for the recognition of *Laccaria* species. Montoya et al. (2015) described *L. roseoalbescens* from *Quercus* dominated forest in Mexico using a combination of morphological data and nuclear ribosomal internal transcribed spacer region (ITS) and the 5′ end of the nuclear ribosomal large subunit (28S). The following year, Popa et al. (2016) described *L. stellata* from *Quercus/Oreomunnea* dominated forest in Panama based on morphology and ITS sequence data. Lastly, Ramos et al. (2017) combined morphology and ITS and 28S sequence data to identify and describe *L. squarrosa* from forests of endangered *Fagus grandifolia* var. *mexicana* in Mexico.

Mueller and Halling (1995) surveyed the macrofungi of Costa Rican *Quercus* montane forests. Their study documented seven species of *Laccaria*: *L. amethystina, L. gomezii, L. laccata, L. major* nom prov*., L. ohiensis, L. proxima*, and *L. trichodermophora* (see Table 1 in Mueller and Halling, 1995). Although these species have yet to be thoroughly studied using molecular data, this brings the total number of neotropical and subtropical *Laccaria* species to ten.

In montane tropical forest of western Panama, the ECM host tree *Oreomunnea mexicana* (Juglandaceae) forms monodominant stands and associates with a highly diverse community of ECM fungi (Corrales et al., 2016). Several inventories employing environmental sequencing of roots in these forests indicate that *Laccaria* is a dominant genus of the fungal community (Corrales et al., 2016, 2017; Corrales and Ovrebo, 2020). However, despite all the sequencing efforts, much of the reported *Laccaria* diversity from these forests remains unidentified and it is apparent that this region has a number of undescribed species. Based on previous research in neotropical monodominant forest of Guyana, as many as 70% of the ECM fungal species in a newly explored area may be undescribed (Henkel et al., 2012). We have observed this same pattern in the *Oreomunnea* monodominant forest where many ECM fungal species appear to be new (Corrales and Ovrebo, 2020).

This study adds to the known diversity of neotropical species of *Laccaria* by using a combination of ITS, 28S, *RPB2* and *EF1*α sequence data, along with macro- and micro-morphological characters. Phylogenetic analysis used the most comprehensive dataset available for each molecular marker. In addition, ITS sequences of species of *Laccaria* growing on root tips of neotropical plant hosts were evaluated to better estimate the diversity of neotropical *Laccaria*, and evaluate how much diversity remains undescribed.

## Materials and Methods

Specimens were collected in a primary lower montane forest (1000–1400 m.a.s.l) at the Fortuna Forest Reserve in western Panama. The mean annual temperature at the reserve ranges from 19 to 22°C and the annual rainfall varies from ca. 5800–9000 mm (Cavelier et al., 1996; Andersen et al., 2012). Trips to the study area were made between April 2012 and May 2015 to collect fleshy macrofungi of Agaricomycetes and Pezizomycotina. Specimens were collected in forests dominated by the ECM host tree *Oreomunnea mexicana* with occasional *Quercus* species. Other ECM host species, i.e., *Coccoloba* spp. and *Alfaroa costaricensis*, were also present in the study area at low abundances (Corrales et al., 2016, 2017). *Laccaria* specimens were collected in four different watersheds showing contrasting soil nitrogen (N) availability (Honda, Hornito, Alto Frio, and Zarciadero; 8°45′12″N, 82°13′08″W–8°45.707′ N, W 82°15.677′ W; see Corrales et al., 2016 for detail information about the sites). Additionally, specimens were collected in a long-term N addition experiment in the Honda watershed that has been ongoing since 2006 (Corre et al., 2010; Corrales et al., 2017).

Macromorphological features like color, size, shape and surface features of the pileus, lamellae and stipes were recorded immediately after returning from the field. Sporocarps were then dried for later analysis of micromorphological features. Colors were described using alphanumeric codes from the Methuen Handbook of Color (Kornerup and Wanscher, 1993) in the format of page number, column letter, row number.

When possible, micromorphological measurements were taken from multiple samples. Measurements separated by an “×” indicate length by width aspects, respectively. Individual extremes, or outliers, from this range are given in parentheses. For each description the symbol “$\bar{x}$” describes the range of specimen means. For basidiospores, the *Q* value indicates a long axis and short axis ratio although this is not necessarily a length and width ratio because of the difficulty of observing globose spores in a profile dimension. Basidiospore length and width measurements do not include ornamentation (echinulae). An important diagnostic feature of *Laccaria* species is the length and the width at the base of the basidiospore ornamentation. Species with an average spine length ($\bar{x}$) less than 1.5 μm are described as having echinulate spores. Spores with mean spine length $\bar{x}$ > 1.5 μm are described as echinate.

Total DNA genomic content was extracted from fresh sporocarp tissue stored in CTAB 2X using REDExtract-N-Amp Plant PCR Kit following the manufacturer’s instructions (Sigma-Aldrich). The following primers were used for PCR and cycle sequencing: ITS1F (Gardes and Bruns, 1993) and ITS4 (White et al., 1990) for the ITS region; LR0R and LR5 (Vilgalys and Hester, 1990) for 28S region domains 1–3; and fRPB2-6F (Liu et al., 1999) and bRPB2-7R (Matheny, 2005) for the *RPB2* regions 5–7. PCR conditions consisted of 95°C for 1 min, and then 35 cycles of 95°C for 30 s, annealing temperature for 30 s to 1 min, and 72°C for 1 min, with a final extension time at 72°C for 10 min. Annealing temperatures were 50°C for ITS and 58°C for *RPB2*. Also EF1-983F, EF1-2218R, and EFcf (Rehner and Buckley, 2005) were used for the *EF1*α. PCR for *EF1*α was performed using a touchdown PCR procedure. PCR conditions consisted of 94°C for 2 min, then the initial annealing temperature was 66°C, and was subsequently incrementally reduced by 1°C per cycle over the next 9 cycles, then 36 cycles of 94°C for 30 s, 56°C for 30 s, 72°C for 1 min, with a final extension time at 72°C for 10 min (Rehner and Buckley, 2005). PCR amplicons were visualized on 1.5% agarose gel stained with SYBR Green. Positive products were cleaned using ExoSap-IT (Affymetrix, Santa Clara, CA, United States) and sequenced. Root tips from adults, saplings, and seedlings of *Oreomunnea mexicana* collected from the sampling sites (Corrales et al., 2016) that were processed using the same extraction protocols and ITS primers were included in the analysis to inform the ecology of the species.

Newly generated sequences were edited using Codon Code ALIGNER v.3.5.7 (CodonCode Corporation, Dedham, MA, United States)^1^ with generic-level identities for sequences confirmed via BLAST queries of GenBank^2^. Nucleotide datasets were assembled using a combination of new sequences and those derived from GenBank. Datasets were aligned using MUSCLE v.3.8.31 (Edgar, 2004) with default settings, followed by manual alignment using MESQUITE v.2.75 (Maddison and Maddison, 2009).

Systematic datasets were individually assembled for ITS, 28S, *RPB2*, and *EF1*α sequences using representative sequences of *Laccaria* species from all over the world in order to delimit phylogenetic species from the neotropics. Separate maximum likelihood analysis was performed on each gene region to check for systematic conflicts between the different molecular datasets. Once a lack of conflict was determined, all available sequences were put in to a supermatrix for combined analysis. All analyses were implemented on the CIPRES web portal (The CIPRES Portals 2009)^3^. Maximum likelihood bootstrap (ML) analyses were performed using RAxML v.2.2.3 (Stamatakis, 2006). Rapid bootstrapping analysis was performed for total of 1000 bootstrap replicates under default parameters set to GTR + G. This model is sufficient for the purposes of this study as it will produce a reasonable phylogeny for evaluating species relationships in our samples, and approximate their systematic relationship to other *Laccaria* species. Bootstrap support from maximum likelihood analysis ≥80% is reported on the branches of phylogenies. Maximum likelihood bootstrap support ≥90% is considered as “strong” support. Bayesian Metropolis-coupled Markov chain Monte Carlo (MCMC) analyses were performed using the GTR + G model of evolution in mrbayes v.3.1.2 (Ronquist et al., 2012). A test was performed on the combined dataset to identify the optimal model for assessing nucleotide substitution rate using jModelTest (Darriba et al., 2012). The selected model TIM2 + I + G was used in a maximum likelihood analysis using RAxML-NG (Kozlov et al., 2019). The resulting phylogenetic tree was nigh identical to the one produced using RAxML under GTR + G. All further results discussed reflect those from the latter analysis. The analyses used four chains and sampling every 100th tree for 10 million generations. The burn-in to be removed was determined using LogCombiner v.1.8.0 (Drummond and Rambaut, 2007) either by taking the first 10% of the iterations, or, if convergence around a stable average likelihood involved >10% of the iterations, by simply removing this proportion of trees from the analysis. Bayesian posterior probabilities (PP) ≥ 0.95 are reported with MLB support on the branches of the phylogenies, with PP ≥ 0.98 considered “strong” support.

## Results

**FIGURE 1:**
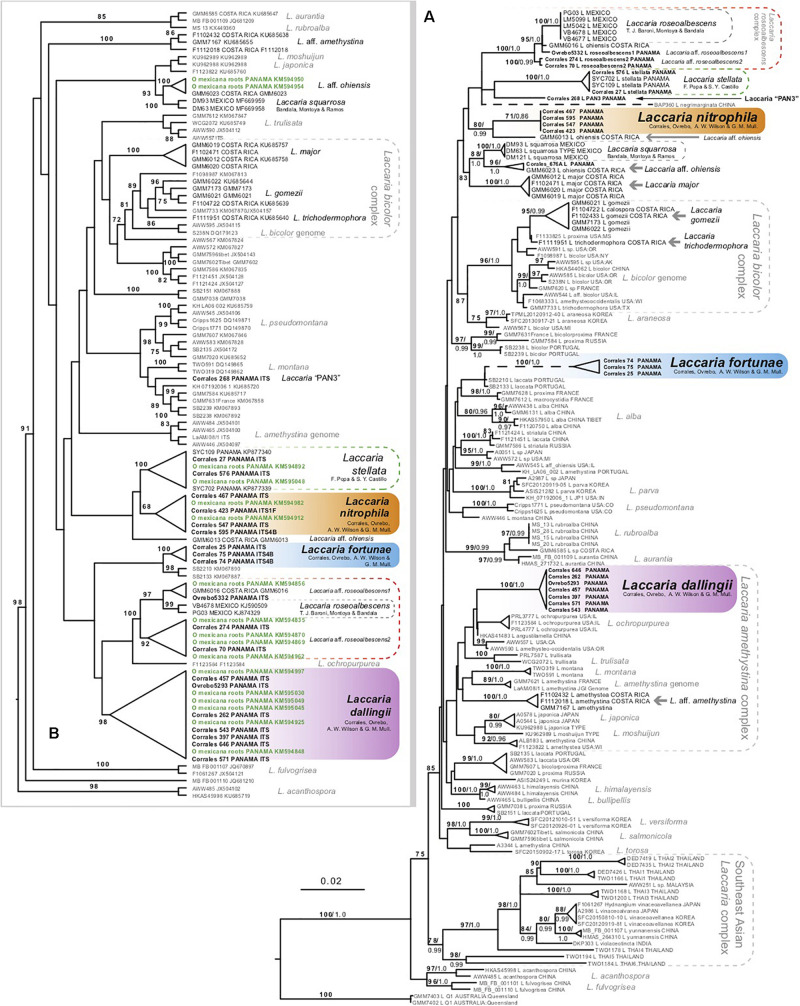
**(A)** Maximum likelihood phylogeny of northern hemisphere *Laccaria* using combined ITS, 28S, *RPB2*, and *EF1*α dataset. **(B)** Maximum likelihood phylogeny using ITS sequence data to identify species of *Laccaria* growing on roots of *Oreomunnea mexicana.* Root tip samples are in green. Numbers above branches represent maximum likelihood bootstrap percentages (in bold) followed by Bayesian posterior probabilities. Gray arrows identify additional species of neotropical *Laccaria* not fully addressed here or in previous systematic studies.

Phylogenetic analysis of combined and individual ITS, 28S, *RPB2*, and *EF1*α dataset identify and lend support for multiple species of *Laccaria* occurring in Panama, Costa Rica, and tropical Mexico. The supermatrix dataset used to phylogenetically delimit species of *Laccaria* from Panama consisted of sequence data from 152 specimens (Figure 1A). The supermatrix dataset combines ITS, 28S, *RPB2*, and *EF1*α sequences, each contributing 679, 734, 1073, and 1008 base pairs, respectively for a total of 3494 bp in the combined dataset. The number of polymorphic sites in the dataset was 1168/3494 (33%). For the Bayesian analysis, the average effective sample size (ESS) is 2143.37 using a 10% burnin. The number of specimens represented by each sequence region includes 149 for ITS, 110 for 28S, 83 for *RPB2*, and 58 for *EF1*α. The results of the separate analyses, as well as a table for all sequence data used and generated from this study can be found in the Supplementary Materials.

The phylogeny from the combined datasets is represented in Figure 1A. This figure identifies 15 putative species-rank lineages of *Laccaria* from Mexico, Costa Rica, and Panama. Up to seven *Laccaria* species from *Oreomunnea mexicana* forests of Fortuna, Panama were resolved by these analyses, with *L. stellata* as the only one previously reported from these forests. The other species include *L. nitrophila* sp. nov. (71% ML and 0.86 PP), *L. dallingii* sp. nov. (100% ML and 1.0 PP), and *L. fortunensis* sp. nov. (100% and 1.0 PP). These species are formally described within this paper. The other three potential species (*L.* aff. *roseoalbescens* 1 and 2 and *Laccaria* “PAN3”) (Figures 1A,B), are not described or proposed in this study due to the few observed specimens and limited material available to assess their morphological and ecological features.

Another ITS dataset consisting of 108 ITS sequences was created to phylogenetically identify *Laccaria* species occurring in ECM roots of *Oreomunnea mexicana.* This dataset contained ITS sequence data from 16 root tip samples. All 16 ECM sequences resolved with at least one sequence from a vouchered specimen of *Laccaria* (Figure 1B). The majority of root tips resolved with either *L. dallingii* or within the *L. roseoalbescens* complex. At least two ECM sequences resolved with *L. nitrophila, L. stellata*, or with a specimen identified as *L.* aff. *ohiensis* (Figure 1B). No ECM root tips were recovered representing *L. fortunensis*.

## Taxonomy

### Laccaria dallingii

**PLATE 1:**
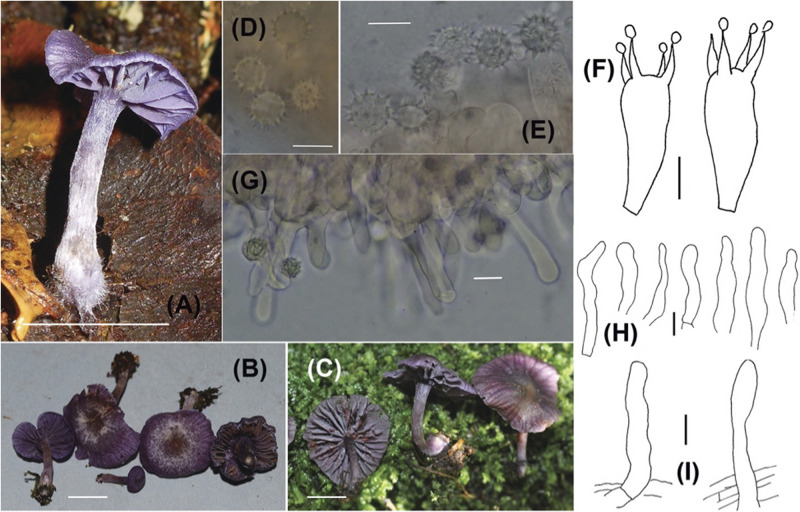
*Laccaria dallingii* **(A–C)** Sporocarps (**A** = Corrales 397, **B** = Ovrebo 5316, **C** = Corrales 571); **(D,E)** Basidiospores (**D** = Corrales 571, **E** = Corrales 489); **(F)** Basidia (**F** = Ovrebo 5293); **(G,H)** Cheilocystidia (**G** = Ovrebo 5316, **H** = Corrales 571 and 543); **(I)** Elements from pileipellis (**I** = Corrales 543 and 457). Scale bars for all macroscopic images = 1 cm. Scale bars for all microscopic images = 10 μm.

Corrales, Ovrebo, A. W. Wilson & G. M. Mueller sp. nov. Plate 1.

MycoBank 835136.

### Typification

PANAMA. Chiriquí Province: Fortuna Forest Reserve, Quebrada Honda, 3 km NE from the Fortuna Dam, 8°45.282′ N, 82°14.430′ W, 10 Dec 2013, Corrales 571 (**holotype** PMA; **isotype** DBG). GenBank: ITS MT279240; 28S MT279214.

### Etymology

This species is named in honor of ecologist Professor James W. Dalling who has dedicated a significant part of his career to study the neotropical montane forests of the Fortuna Forest Reserve. Professor Dalling was the Ph.D. advisor to lead author A. Corrales.

### Diagnosis

Distinguished from other Panamanian *Laccaria* by basidiomes that are purple overall and the presence of cheilocystidia.

#### Description

Pileus (9)12-22 mm diam., broadly convex, generally depressed at center, margin straight or incurved, glabrous or occasionally fibrillose-scaly, moist, translucent-striate, very slightly sulcate, purple (16D5, 16E8-16F8), occasionally becoming brownish at the center, hygrophanous, lightening considerably upon drying; context less than 1 mm thick, purple, no odor, taste non-descript. Lamellae 1.5-3 mm wide, adnate to subdecurrent, concolorous with pileus, entire, distant, lamellulae present. Stipe 12-30 (50) × 1-5 mm, equal, base often subbulbous, sometimes curved, glabrous, dull, purple or purplish buff, hollow; context purple; light purple strigose mycelium at base.

Basidiospores (without ornamentation) (7.5) 8-9 × (7.5) 8-9 μm (100/10, $\bar{x}$ = 8.5 × 8.3 μm, *Q* = 1-1.07, mean *Q* = 1.02), subglobose to globose in profile and face view, echinate, spines to 1.5-2 μm long, 1 μm wide at base, crowded, spores and spines hyaline, inamyloid. Basidia (28) 31-45 × (9) 11-13 μm, clavate, soon collapsing, 4-sterigmate, sterigmata to 10 μm long, to 3 μm wide at base, hyaline. Cheilocystidia scattered, sometimes clumped, most easily confirmed on face sections, 25-57 × 4-9 μm with most measurements taken from edge of hymenium, cylindric and hyphae-like, apex rounded, occasionally constricted near apex or elsewhere, smooth, thin-walled, hyaline, occasionally slightly translucent. Hyphae of lamellar trama 4-13 μm wide, parallel, pale orange-pink in mass in 3% KOH. Hyphae of subhymenium 3-4 μm wide. Hyphae of pileus surface a cutis and in places slightly interwoven, 4-12 μm wide, with scattered cystidioid end-cells that are appressed or recurved, end-cells (15) 20-50 × 5-9 μm, cylindric, clavate, smooth, hyaline. Hyphae of pileus trama 4-15 μm wide, orange-pink in mass in 3% KOH. Hyphae of stipe surface 3-5 μm wide, a cutis or in places slightly interwoven, with scattered cystidioid end-cells, appressed or recurved, end-cells (20) 30-55 (95) × 6-10 μm, cylindric, clavate, smooth, thin-walled, hyaline. Hyphae of stipe trama to 15 μm wide, cells sometimes rather short, orange-pink in mass in KOH. Clamp connections present but not at every cross wall.

### Habitat and Phenology

Solitary, scattered, gregarious or caespitose, on wood, soil or leaf litter, two collections on moss covered log. Growing in *Oreomunnea mexicana* monodominant forest and recovered from ECM root tips of *O. mexicana*. Found fruiting during January - February and July thorough December.

### Other Specimens Examined

PANAMA. Chiriquí Province: Fortuna Forest Reserve: Quebrada Honda, 3 km NE from the Fortuna Dam, 8°45.282′ N, 82°14.430′ W, 16 Feb 2012, A. Corrales 32 (ARIZ), 23 Sep 2013, A. Corrales 397 (PMA), 26 Oct 2013, A. Corrales 457 (DBG), 30 Oct 2013, A. Corrales 489 (FLAS), 23 Nov 2013, A. Corrales 543 (NY), 13 Jan 2014, A. Corrales 646 (DBG), 10 Jul 2014, C. Ovrebo 5293 (CSU), trail to Cerro Hornito from Bocas del Toro road, N 8° 40.440′, W 82° 13.038′, 7 Jul 2012, A. Corrales 262 (ARIZ), 13 Jul 2014, C. Ovrebo 5316 (CSU, DBG).

### Notes

The new species *Laccaria dallingii* is clearly the most striking of this study. As part of the *L. amethystina* complex, it has a deep purple coloration that helps it to stand apart from the other *Laccaria* species in these forests. As discussed in Vincenot et al. (2017) the *L. amethystina* complex consists of several, geographically separate but morphologically similar species and several species have been formally described within the group. Thus, it is not surprising to recover a distinct taxon in theses *Oreomunnea mexicana* monodominant forests. However, there is currently at least two other purple *Laccaria* occurring in neotropical montane forests, *L. gomezii* and a taxon awaiting formal designation recovered from Costa Rica and Colombia (Figure 1A, specimens F1102432, F1112018, and GMM7167). *Laccaria gomezii* is morphologically distinct and easily distinguished based on its close, decurrent lamellae and subglobose basidiospores. While *L. dallingii* forms a clade distinct from the aforementioned unidentified Costa Rican and Colombian taxa in the *L. amethystina* complex (see above), they are still very similar morphologically making it difficult to distinguish them from each other. Based on current distribution data for the two taxa, they can be differentiated by geography, but more survey work throughout the region is needed to confirm this. Further work on the American (North, Central, and South American) members of the complex is needed.

### Laccaria nitrophila

**PLATE 2:**
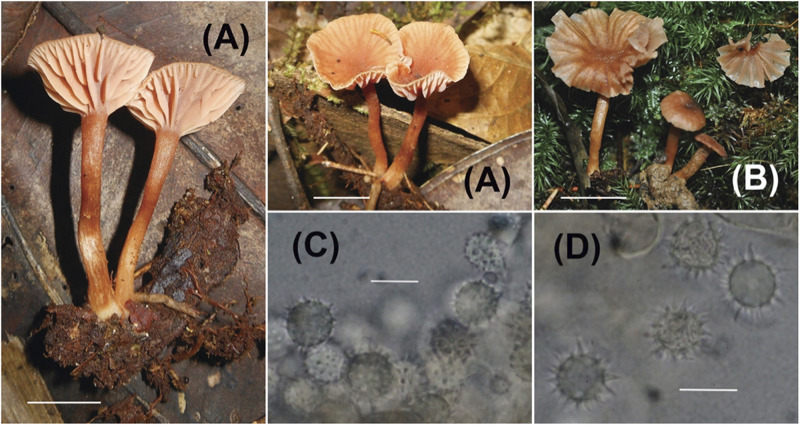
*Laccaria nitrophila* **(A,B)** Sporocarps (**A** = Corrales 423, **B** = Corrales 595); **(C,D)** Basidiospores (**C** = Corrales 595, **D** = Corrales 423). Scale bars for all macroscopic images = 1 cm. Scale bars for all microscopic images = 10 μm.

Corrales, Ovrebo, A. W. Wilson & G. M. Mueller sp. nov. Plate 2.

MycoBank 835137.

### Typification

PANAMA. Chiriquí Province, Fortuna Forest Reserve, Quebrada Honda watershed, 8°45.286′ N, 82°14.452′ W, 10 Jan 2014, A. Corrales 595 (**holotype** PMA). GenBank: ITS MT279236; 28S MT279211; rpb2 MT431186; tef1 MT436074.

### Etymology

*nitr* (L., reference to nitrogen), *phil* (Gr., reference to love), this species prefers soil habitats with high inorganic nitrogen as it was the most recovered OTU in the roots of *Oreomunnea mexicana* collected from a long-term nitrogen addition experiment at the Fortuna Forest Reserve.

### Diagnosis

Relatively small, orange brown, translucent striate pileus, distant and thick lamellae, and globose, echinulate basidiospores.

#### Description

Pileus 7-20 mm diam., convex, plano-convex to uplifted and slightly depressed, surface dry, smooth to fibrillose, striate, slightly translucent to translucent, margin undulate, orange-brown, brownish orange, hygrophanous; context to 1.5 mm thick. Lamellae adnate, subdecurrent, pinkish orange, orange-brown, edge entire, distant to subdistant; lamellulae present and sometimes anastomosing. Stipe 15-28 × 2-3 mm, equal, strict or curved, surface dry, fibrillose with white fibrils against an orange-brown background, scant white basal mycelium; context fistulose, orange-brown.

Basidiospores (without ornamentation) 8-9 (9.5) × 8-9 (9.5) μm (60/4, $\bar{x}$ = 8.6 × 8.4 μm, *Q* = 1-1.06, mean *Q* = 1.03), globose in profile and face view, echinate, spines 1.5-2 μm long, 1 μm wide at base, hyaline, inamyloid. Basidia 35-50 × 12-13 μm, mainly collapsed, 4-sterigmate, pale yellow as a layer, sterigmata to 9 × 3 μm. Hymenial cystidia not seen. Hyphae of subhymenium 3-4 μm wide, hyaline. Hyphae of pileus surface a cutis that is often tightly interwoven, hyphae 4-10 μm wide, smooth or slightly incrusted, hyaline to pale yellow. Hyphae of pileus trama 5-12 μm wide, pale yellow in mass. Hyphae of stipe surface 3-5 μm wide, appressed, smooth to slightly incrusted, hyaline. Hyphae of stipe trama 5-11 μm wide, pale yellowish in mass. Clamp connections present but not at every septum.

### Habitat and Phenology

Solitary or scattered, on soil. Ectomycorrhizal in *Oreomunnea mexicana* monodominant forest. Found fruiting during January and October - November.

### Other Specimens Examined

PANAMA. Chiriquí Province, Fortuna Forest Reserve, Quebrada Honda watershed, 8°45.286′ N, 82°,14.452′ W, 10 Oct 2013, A. Corrales 423 (ARIZ), 26 Oct 2013, A. Corrales 467 (DBG), 23 Nov 2013, A. Corrales 547 (DBG).

### Notes

*Laccaria nitrophila* has the least amount of statistical phylogenetic support from ML or Bayesian analysis. This is due in part to the over reliance on ITS data, since there are not sequence data available from other markers for more than one specimen. For these ITS sequences there is high variability in coverage among the sequences available as the quality of the ITS sequences obtained from these specimens was low. As a result, many of the sequences needed to be trimmed excessively, making them short and therefore producing inconsistent overlap between sequences for comparison. The sequence overlap in ITS data matrix provided just enough comparison to allow these specimens to be clustered in phylogenetic analysis, but was limited enough that the bootstrap analysis and Bayesian posterior probabilities turned out lower than expected for intraspecific comparisons.

Morphologically, *L. nitrophila* is part of the *L. ohiensis* complex along with *Laccaria stellata* F. Popa & S. Y. Castillo. Species in the complex are relatively small, have orange brown translucent striate pilei, distant thick lamellae, and globose basidiospores with prominent echinulae. Because of the slight variations among these characters, field identification of the different species is very difficult. *Laccaria stellata* which co-occurs with *L. nitrophila* has red and pink colorations and slightly smaller basidiospores with longer echinulae along with a reported presence of pleuro- and cheilocystidia. However, it is important to note that the descriptions of these cystidia in the original publication by Popa et al. (2016) do not effectively differentiate these cells from basidioles (1). We were able to detect cystida-like cells in the hymenium of *L. stellata* specimens collected in this study (A. Corralles 576).

*Laccaria squarrosa* Mandala, Montoya & Ramos and *L. roseoalbescens* T. J. Baroni, Montoya & Bandala are both described from neotropical forests of Mexico. The former has distinct squarrose hairs along the stipe. This species also has cheilocystida which are not observed in *Laccaria nitrophila*. *Laccaria roseoalbescens* is distinctly paler and pinkish, fading to yellow relative to the orange-brown-beige of *L. nitrophila*. There are also “cystidia-like” sterile elements within the hymenium of *L. roseoalbescens.*

### Laccaria fortunensis

**PLATE 3:**
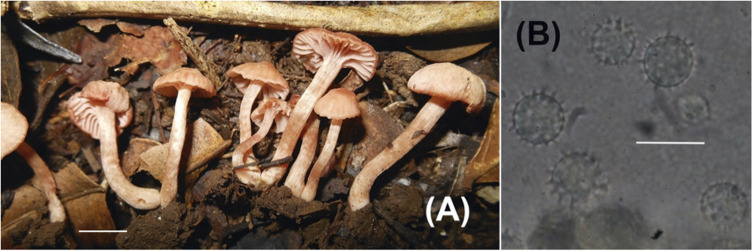
*Laccaria fortunensis* **(A)** Sporocarps (Corrales 74); **(B)** Basidiospores (Corrales 74). Scale bars for all macroscopic images = 1 cm. Scale bars for all microscopic images = 10 μm.

Corrales, Ovrebo, A. W. Wilson & G. M. Mueller sp. nov. Plate 3.

MycoBank 835138.

### Typification

PANAMA. Chiriquí Province: Fortuna Forest Reserve, Zarciadero, off of Bocas del Toro road, 2 km NW of dam, trail originating at old ANAM station, 8° 45.707′ N, 82° 15.677′ W, 10 April 2012, Corrales 74 (**holotype** PMA, **isotype** ARIZ). GenBank: ITS MT279246.

### Etymology

Named for the Fortuna Forest Reserve where this research was conducted.

### Diagnosis

Characterized by the rather small basidiomes, tan pileus color, more or less globose basidiospores with short and narrow echinulae, neotropical distribution and sequence data.

#### Description

Pileus 8-15 mm wide, convex with decurved margin, plano-convex to nearly plane, margin often becoming eroded, surface dry, glabrous, striate near edge, light orange-tan to brownish orange, context light orange-tan. Lamellae to 3 mm wide, adnate to subdecurrent, light pink, subdistant to distant, lamellulae present. Stipe 20-42 × 3-5 mm, thick, central, equal, glabrous, silky, buff to light orange-tan, lightest at apex, hollow.

Basidiospores (without ornamentation) 7-8 × 7-8 μm (60/3, $\bar{x}$ = 7.6 × 7.4, *Q* = 1-1.07 (1.14), mean *Q* = 1.04), globose or nearly so, echinulate, spines up to 1 μm long, 0.5 – rarely 1 μm wide at base, hyaline, inamyloid. Basidia 33-49 × 8-11 μm, clavate but frequently collapsed even when spores are attached, sterigmata up to 8 μm long, often bent and contorted at angles especially as the basidium is collapsed, hyaline but pale yellowish as a layer. Hymenial cystidia not seen. Hyphae of lamellar trama 3-11 μm wide, parallel, hyaline but pale yellow in mass in KOH. Hyphae of subhymenium 3 μm wide, hyaline. Pileipellis a cutis or slightly interwoven, hyphae 4-7 μm wide, smooth, thin-walled, cylindric, light golden yellow. Hyphae of pileus trama to 11 μm wide, hyaline. Hyphae of stipe surface 3-6 μm wide, a cutis, smooth, thin-walled, light golden yellow. Stipe trama hyphae up to 14 μm wide, hyaline. Clamp connections present but not at every cross wall.

### Habitat and Phenology

Gregarious to caespitose, on soil. Growing in *Oreomunnea mexicana* monodominant forest. Found fruiting during February and April.

### Other Specimens Examined

PANAMA. Chiriquí Province: Fortuna Forest Reserve: Zarciadero, off of Bocas del Toro road, 2 km NW of dam, trail originating at old ANAM station, 8° 45.707′ N, 82° 15.677′ W, 9 Feb 2012, Corrales 25 (ARIZ), 10 April 2012, Corrales 75 (ARIZ).

### Notes

*Laccaria fortunensis* is characterized by the small, thin-fleshed basidiomes and by the phylogenetic placement. In this study it is distinguished from *L. nitrophila* by having smaller, finely ornamented basidiospores.

The coloration for *Laccaria fortunensis* lies somewhere between *L. nitrophila* and *L. roseoalbescens* in being paler than the former, but darker brown than the latter. The fine ornamentation on globose basidiospores is diagnostic and not seen in other described Neotropical species of *Laccaria*.

## Discussion

Phylogenetic analysis (Figure 1) resolves up to 15 lineages of *Laccaria* within the neotropics. These include three species recently described using phylogenetic analysis: *L. stellata*, *L. roseoalbescens*, and *L. squarrosa* (Montoya et al., 2015; Popa et al., 2016; Ramos et al., 2017). Of the remaining twelve species, five of these represent species not recovered in the forests of Fortuna, Panama (gray horizontal arrows in Figure 1A). Many of these represent *Laccaria* from Costa Rica collected from montane *Quercus* forests. These species represent systematically distinct lineages. Two of them resolve within the *Laccaria bicolor* complex, one resolves within the *Laccaria amethystina* complex, and another two fall within the broadly defined *L. ohiensis* complex.

There are two sporocarp collections as well as two ECM sequences that resolve within the *L. stellata* clade (Figures 1A,B). The lack of obvious morphological differences between *L. stellata* and *L. nitrophila* makes identifying these sympatric species without molecular data challenging. Similarly, there are no clear morphological features differentiating *L. dallingii* from others in the *L. amethystina* clade. This highlights the need for using molecular data to differentiate *Laccaria* species within complexes.

One of the more interesting results of this study is the observation of a new species complex centered around *L. roseoalbescens*. This species was originally described from the cloud forests of Mexico (Montoya et al., 2015). In Panama, there are two taxa sister to *L. roseoalbescens* in the main phylogeny (Figure 1A). The specimen Ovrebo 5332 is directly sister to *L. roseoalbescens*, along with Costa Rican specimen GMM6016. These specimens form a clade and are tentatively identified as *L.* aff. *roseoalbescens* 1 in the phylogeny, and together with *L. roseoalbescens* form a clade that is supported with 95% MP and 1.0 PP. Until more specimens and sequence data are available to evaluate this potential species, we are content to leave it undescribed. Sister to this group is another putative species named *L.* aff. *roseoalbescens* 2. This is represented by two collections: Corrales 70 and Corrales 274. Unfortunately, these consist of only one and two sporocarps each. As a result, there is insufficient material to properly describe this species morphologically. Altogether this *Laccaria roseoalbescens* complex is supported with 100% ML and 1.0 PP (Figure 1A).

Based on sequences from root tips, we were able to confirm *Oreomunnea mexicana* as the host species for *L. dallingii*, *L. nitrophila*, and two species in the *L. roseoalbescens* complex. *Laccaria fortunensis* was not recovered from *O. mexicana* root tips due to the lack of root tip inventories in the locality were this species was collected. While we expect *L. fortunensis* to be associated with the same host plant, ECM associations with *Quercus* in the region is also a possibility. *Laccaria* has been reported as a nitrophilic genus due to the positive response to soil nitrogen availability by some of its species in terms of both sporocarp production and colonization of root tips in temperate and boreal ecosystems (Lilleskov et al., 2011; van der Linde et al., 2018). In tropical *Oreomunnea mexicana* monodominant forest of the Fortuna Forest Reserve, *Laccaria* has also been reported to be more abundant in sites with high natural soil fertility that in sites with low nitrogen availability (Corrales et al., 2016). Also, in a long term nitrogen addition experiment at the same study site (Corre et al., 2010), the relative abundance of *Laccaria* increased from 1% in the control plots to 10% in the nitrogen fertilized plots (1 OTU showed a negative response while 5 OTUs responded positively) in an study using high-throughput amplicon sequencing to characterize the ECM fungal communities associated with the roots of *O. mexicana* individuals (Corrales et al., 2017). *L. nitrophila*, described in this paper, is the species that was reported by Corrales et al., 2017, as the *Laccaria* OTU with the most positive response to inorganic nitrogen addition.

Neotropical forests of Central America host a number of *Laccaria* species that remain to be identified. Many of these are likely to be new, but require additional material to be adequately analyzed and described. While sufficient specimens already exist for some of the suspected species, these specimens require additional study. Altogether, this study suggests that there are as many as 15 species of *Laccaria* in the neotropical forests of Central America, and it is quite probable that the diversity is even higher. These results increase the number of suspected species in the region by 50% and doubles the number of systematically described *Laccaria* from this part of the world.

## Data Availability Statement

The data that support the findings of this study are openly available in GenBank of NCBI under the accession numbers provided in the article.

## Author Contributions

AC, CO, and AW conceived the idea, performed the research, and collected the data. AW analyzed the molecular data. AW, AC, and CO wrote the first draft of the manuscript. All the authors contributed substantially to the revisions.

## Conflict of Interest

The authors declare that the research was conducted in the absence of any commercial or financial relationships that could be construed as a potential conflict of interest.

## Footnotes

1 http://www.codoncode.com/

2 http://www.ncbi.nlm.nih.gov/

3 http://www.phylo.org/portal2/
