# Supplementary material for: Novel Laccaria Species From Juglandaceae Forest in Panama With Notes on Their Ecology
Source: Front Microbiol. 2020 Jul 17;11:1597. doi: 10.3389/fmicb.2020.01597 (PMC7380087; doi:10.3389/fmicb.2020.01597)
Supplement: Supplementary file 2 [file Data_Sheet_2.docx]

Manuscript title: *Title: Novel Laccaria species from Juglandaceae forest in Panama with notes on their ecology*

Table S1. Specimen data for molecular phylogenetic datasets. Species described as part of this paper are highlighted in a gray background.

| **SpecimenID** | **Genus Species** | **Location** | **Host** | **ITS** | **28S** | **RPB2** | **ef1a** |
| --- | --- | --- | --- | --- | --- | --- | --- |
| A0051 | *Laccaria* sp. | Japan, Narusawa | Abies sp., Tsuga diversifolia, Larix leptolepis | KU685613 | - | - | - |
| A0544 | *Laccaria japonica* | Japan, Funyu | - | JN942802 | JN939755 | JN993509 | - |
| A0578 | *Laccaria japonica* | Japan, Tsuchiyu | - | JN942783 | - | - | - |
| A2986 | *Laccaria vinaceoalvanea* | Japan, Fushimi | - | JN942810 | JN939738 | JN993520 | - |
| A2987 | *Laccaria* sp. | Japan, Fushimi | - | JN942786 | JN939739 | JN993521 | - |
| A3344 | *Laccaria amethystina* | China | - | KU685627 | - | - | - |
| ALB183 | *Laccaria amethystina* | China, Tibet | - | JX504092 | JX504176 | KU686058 | KU686161 |
| AWW251 | *Laccaria* sp. | Malaysia, Pahang Prov., Frazier's Hill | - | JX504093 | JX504177 | - | - |
| AWW438 | *Laccaria alba* | China, Yunan, Shangrila Co. | - | JX504094 | JX504178 | KU685912 | KU686072 |
| AWW446 | *Laccaria montana* | China, Xizang, Bomi Xian | - | JX504097 | JX504181 | KU686054 | KU686157 |
| AWW463 | *Laccaria himalayensis* | China, Tibet, Linzhi Co. | - | JX504098 | JX504182 | KU685913 | - |
| AWW465 | *Laccaria bullipellis* TYPE | China, Tibet, Linzhi Co. | - | JX504100 | JX504184 | KU685914 | -- |
| AWW484 | *Laccaria himalayensis* TYPE | China, Tibet, Linzhi Co. | - | JX504101 | JX504185 | KU685915 |  |
| AWW485 | *Laccaria acanthospora* TYPE | China, Tibet, Linzhi Co. | - | JX504102 | JX504186 | KU685916 | KU686073 |
| AWW544 | *Laccaria* aff. *bicolor* | USA, IL, Johnson Co. | - | KM067820 | - | **MT431173** | - |
| AWW545 | *Laccaria* aff. *ohiensis* | USA, IL, Johnson Co. | - | JX504106 | JX504190 | KU685917 | - |
| AWW557 | *Laccaria* sp. | USA, CA, Nevada Co. | - | **MT279220** | **MT279200** | **MT431174** | **MT436061** |
| AWW567 | *Laccaria bicolor* | USA, MI, Upper Peninsula | - | KM067824 | KU685765 | - | - |
| AWW572 | *Laccaria* sp. | USA, MI, Upper Peninsula | - | KM067827 | KU685767 | KU685921 | - |
| AWW583 | *Laccaria laccata* | USA, OR, Benton Co. | - | KM067828 | KU685768 | - | - |
| AWW590 | *Laccaria amethysteo-occidentalis* | USA, OR, Benton Co. | - | JX504112 | JX504195 | KU685923 | - |
| AWW591 | *Laccaria* sp. | USA, OR, Benton Co. | - | KM067830 | KU685769 | KU685924 | - |
| AWW595 | *Laccaria* sp. | USA, AK | - | JX504115 | JX504198 | - | - |
| BAP360 | *Laccaria negrimarginata* TYPE | China, Tibet | - | JX504120 | - | - | - |
| Cripps1625 | *Laccaria pseudomontana* TYPE | USA, CO | - | DQ149871 | - | - | - |
| Cripps1771 | *Laccaria pseudomontana* | USA, CO | - | DQ149870 | - | - | - |
| F1061267 | *Hydnangium vinaceoavellanea* | Japan, Tottori | *Castanopsis* | JX504121 | - | - |  |
| F1098987 | *Laccaria bicolor* | USA, NY | *Pinus* | KM067813 | **MT279201** | **MT431175** | **MT436062** |
| F1121424 | *Laccaria striatula* | China | Pinaceae | JX504127 | JX504207 | KU686064 | - |
| F1123822 | *Laccaria amethystea* | USA, WI | *Juniperus* | KU685760 | KU685911 | KU686071 | - |
| GMM7020 | *Laccaria proxima* | Russia, Caucasus | Fagaceae, Pinaceae | KU685652 | KU685795 | KU685938 | - |
| GMM7167 | *Laccaria amethystina* | - | - | KU685655 | KU685798 | - | KU686083 |
| GMM7584 | *Laccaria proxima* | Komorav Forest, Russia | - | KU685717/ KM067833 | KU685858 | KU685999 | KU686120 |
| GMM7586 | *Laccaria striatula* | Komorav Forest, Russia | - | KM067835 | KU685859 | KU686000 | - |
| GMM7596tibet | *Laccaria salmonicola* | China, Tibet | Picea, Larix | JX504143 | JX504218 | KU686045 | KU686151 |
| GMM7602Tibet | *Laccaria salmonicola* | China, Tibet | - | JX504145 | JX504220 | - | - |
| PRL7587 | *Laccaria trullisata* | - | - | KM067882 | JX504247 | KU686047 | KU686153 |
| WCG2072 | *Laccaria trullisata* | - | - | KU685749 | - | KU686044 | KU686150 |
| SB2151 | *Laccaria laccata* | Portugal | - | KM067888/ JX504173 | JX504250 | **MT431176** | **MT436063** |
| GMM7038 | *Laccaria proxima* | Russia, Caucasus | Fagaceae, Pinaceae | **MT279221** | **MT279202** | **MT431177** | **MT436064** |
| SB2135 | *Laccaria laccata* | Portugal | - | JX504172 | JX504249 | KU686028 | KU686140 |
| SB2238 | *Laccaria bicolor* | Portugal | - | KM067892 | KU685886 | KU686030 | - |
| SB2239 | *Laccaria bicolor* | Portugal | - | KM067893 | KU685887 | KU686031 | - |
| GMM6131 | *Laccaria alba* | China | - | JX504131 | JX504210 | KU685930 | KU686079 |
| F1120750 | *Laccaria alba* | China | Pinaceae, Fagaceae | JX504126 | JX504242 | - | - |
| F1133825 | *Laccaria proxima* | USA, MS | Pinaceae, Fagaceae | KU686064 | KU685786 | KU686065 | - |
| F1068333 | *Laccaria amethysteo-occidentalis* | USA, WI | - | KU685629 | KU685773 | KU686061 | - |
| SB2133 | *Laccaria laccata* | Portugal | - | KM067887 | KU685884 | KU686027 | KU686139 |
| SB2210 | *Laccaria laccata* | Portugal | - | KM067890 | KU685885 | KU686029 | KU686141 |
| F1123584 | *Laccaria ochropurpurea* | USA, IL | Quercus | **MT279222** | **MT279203** | - | - |
| F1121451 | *Laccaria laccata* | China | Pinaceae | JX504128 | JX504208 | - | - |
| AWW585 | *Laccaria bicolor* | USA, OR, Benton Co. | - | JX504111 | JX504194 | - | - |
| GMM7607 | *Laccaria bicolorproxima* | Vallon d. Arcillac, France | - | KM067846 | **MT279204** | **MT431178** | **MT436065** |
| GMM7612 | *Laccaria macrocystidia?* | Vallon d. Arcillac, France | - | KM067847 | KU685861 | KU686002 | KU686122 |
| GMM7620 | *Laccaria* sp. | Forest comaniale de Ste. Croix, France | - | KM067854/  JX504149 | JX504223 | **MT431179** | **MT436066** |
| GMM7621 | *Laccaria amethystina* | Forest comaniale de Ste. Croix, France | - | JX504150 | JX504224 | KU686046 | KU686152 |
| GMM7628 | *Laccaria proxima*? | Forest comaniale de Ste. Croix, France | - | KM067857 | KU685867 | KU686008 | KU686127 |
| GMM7631France | *Laccaria bicolor / proxima* | Forest comaniale de Ste. Croix, France | - | KM067858 | KU685869 | KU686010 | KU686129 |
| GMM7733 | *Laccaria trichodermophora* | USA, TX, Tyler Co. | - | KM067870/ JX504157 | JX504230 | KU686013 | - |
| HKAS41483 | *Laccaria angustilamella* TYPE | China, Longling County | *Quercus*, *Lithocarpus* |  | JX504233 | - | - |
| HKAS44062 | *Laccaria bicolor* | China, Yunnan | - | JX504159 | JX504235 | KU686068 | - |
| HKAS45998 | *Laccaria acanthospora* | China, Tibet | - | KU685719 | KU685870 | KU686069 | - |
| HKAS57950 | *Laccaria alba* | China, Tibet | - | JX504166 | JX504242 | - | - |
| HMAS_264310 | *Laccaria yunnanensis* | China, Yunnan | - | KX496978 | - | - | - |
| HMAS_271732 | *Laccaria aurantia* | China, Yunnan | - | KX496971 | - | - | - |
| KH_07192006_1 | *Laccaria* JP1 | USA, IN | *Quercus* | KU685720 | KU685871 | KU686014 | - |
| KH_LA06_002 | *Laccaria amethystina* | USA, LA | *Quercus, Fagus* | KU685759 | KU685910 | KU686059 | KU686162 |
| KU962988 | *Laccaria japonica* TYPE | - | - | KU962988 | - | - | - |
| KU962989 | *Laccaria moshuijun* TYPE |  | - | KU962989 | - | - | - |
| LaAM/08/1 | *Laccaria amethystina* | JGI Genome | - | JGI | JGI | JGI | JGI |
| MB_FB_001101 | *Laccaria fulvogrisea* | China, Yunnan | - | JQ670896 | - | - | - |
| MB_FB_001107 | *Laccaria yunnanensis* | China, Yunnan | - | JQ670897 | - | - | - |
| MB_FB_001109 | *Laccaria aurantia* | China, Yunnan | - | JQ681209 | - | - | - |
| MB_FB_001110 | *Laccaria fulvogrisea* | China, Yunnan | - | JQ681210 | - | - | - |
| PRL3777 | *Laccaria ochropurpurea* | USA, IL | - | KU685732 | JX504246 | KU686024 | - |
| PRL4777 | *Laccaria ochropurpurea* | USA, IL | Quercus | KU685733 | KU685883 | KU686025 | - |
| S238N | *Laccaria bicolor* | JGI Genome | - | DQ179123 | - | JGI | JGI |
| MS_13 | *Laccaria rubroalba* | China | - | KX449360 | - | - | - |
| MS_28 | *Laccaria rubroalba* | China | - | KX449359 | - | - | - |
| MS_15 | *Laccaria rubroalba* | China | - | KX449358 | - | - | - |
| MS_20 | *Laccaria rubroalba* | China | - | KX449357 | - | - | - |
| DED7419 | *Laccaria* sp. | Thailand | Dipterocarpus | KX513839/ KX513840 | KU685770 | KU685925 | KU686075 |
| DED7426 | *Laccaria* sp. | Thailand | Pinus, Dipterocarpus | KU685628 | KU685771 | KU685926 | KU686076 |
| DED7435 | *Laccaria* sp. | Thailand | Dipterocarpus | KX513841 | KU685772 | KU686060 | **MT436067** |
| TWO1166 | *Laccaria* sp. | Thailand, ChaingMai | Castanopsis, Dipterocarps | KU685744 | KU685895 | KU686041 | **MT436068** |
| TWO1168 | *Laccaria* sp. | Thailand, ChaingMai | Castanopsis, Dipterocarps | KU685745 | KU685896 | KU686042 | KU686146 |
| TWO1178 | *Laccaria* sp. | Thailand, ChaingMai | Pinus, Dipterocarpus | KU685746 | KU685897 | KU686043 | KU686147 |
| TWO1184 | *Laccaria* sp. | Thailand, ChaingMai | Castanopsis | KU685747 | KU685898 | - | KU686148 |
| TWO1194 | *Laccaria* sp. | Thailand, Prajenburi | - | KU685748 | KU685899 | - | KU686149 |
| TWO1200 | *Laccaria* sp. | Thailand | - | KU685743 | KU685894 | KU686040 | **MT436069** |
| TWO319 | *Laccaria montana* | - | - | DQ149862 | - | - | - |
| TWO591 | *Laccaria montana* | - | - | DQ149865 | - | - | - |
| DKP303 | *Laccaria violaceotincta* | India TYPE | - | MK141034 | - | - | - |
| ASIS24249 | *Laccaria murina* | Korea, Jeju-do | - | MG519552 | MG519592 | MG551625 | MG551658 |
| SFC20120919-81 | *Laccaria vinaceoavellanea* | Korea, Chungcheongnam-do | - | MG519534 | MG519577 | MG551609 | - |
| SFC20150810-10 | *Laccaria vinaceoavellanea* | Korea, Soul, Gwanak-gu | - | MG519539 | MG519580 | MG551614 | MG551646 |
| TPML20120912-40 | *Laccaria araneosa* | Korea, Gyeongsangbuk-do | - | MG519548 | MG519588 | MG551621 | MG551654 |
| SFC20130917-21 | *Laccaria araneosa* | Korea, Gyeongsangbuk-do | - | MG519549 | MG519589 | MG551622 | MG551655 |
| SFC20120919-05 | *Laccaria parva* | Korea, Soul, Gwanak-gu | - | MG519529 | MG519573 | MG551604 | MG551640 |
| ASIS21282 | *Laccaria parva* | Korea, Gyeonggi-do | - | MG519526 | MG519571 | MG551601 | MG551638 |
| SFC20150902-17 | *Laccaria torosa* | Korea, Gyeongsangbuk-do | - | MG519561 | MG519598 | MG551631 | MG551664 |
| SFC20121010-51 | *Laccaria versiforma* | Korea, Gyeongsangbuk-do | - | MG519555 | MG519593 | MG551626 | MG551659 |
| SFC20120926-01 | *Laccaria versiforma* | Korea, Gyeongsangbuk-do | - | MG519556 | MG519594 | MG551627 | MG551660 |
| F1102432 | *Laccaria amethystea* | CostaRica | Quercus | KU685638 | KU685781 | KU686062 | - |
| F1102433 | *Laccaria gomezii* | CostaRica | Quercus | - | **MT279205** | **MT431180** | **-** |
| F1102471 | *Laccaria major* | CostaRica, San Jose | Quercus | **MT279224** | **-** | - | - |
| F1104722 | *Laccaria calospora* | CostaRica | Quercus | KU685639 | KU685782 | - | - |
| F1111951 | *Laccaria trichodermophora* | CostaRica | Quercus | KU685640 | KU685784 | KU686063 | - |
| F1112018 | *Laccaria amethystina* | CostaRica | Quercus | **MT279225** | **-** | - | - |
| GMM6012 | *Laccaria major* | Costa Rica | Quercus | KU685758 | KU685909 | KU686057 | - |
| GMM6013 | *Laccaria ohiensis* | CostaRica, San Jose | Quercus | **MT279226** | **MT279206** | **MT431181** | **MT436070** |
| GMM6019 | *Laccaria major* | CostaRica, San Gerardo | Quercus | KU685757 | KU685908 | KU686056 | KU686160 |
| GMM6020 | *Laccaria major* | CostaRica, San Gerardo | Quercus | **KM067854 / JX504149** | **JX504223** | **-** | - |
| GMM7173 | *Laccaria gomezii* | - | - | **MT279227** | **MT279207** | **MT431182** | **MT436071** |
| GMM6021 | *Laccaria gomezii* | - | - | **MT279228** | **-** | - | - |
| GMM6022 | *Laccaria gomezii* | - | - | KU685644 | - | - | - |
| GMM6023 | *Laccaria ohiensis* | CostaRica, San Gerardo | Quercus | **MT279229** | **MT279208** | **MT431183** | **MT436072** |
| GMM6585 | *Laccaria* sp. | CostaRica, San Gerardo | Quercus | KU685647 | KU685791 | KU685933 | - |
| SYC109 | *Laccaria stellata* | Panama, Fortuna | *Quercus insignis* | KP877340 | - | - | - |
| SYC702 | *Laccaria stellata* | Panama, Fortuna | *Quercus insignis* | KP877339 | - | - | - |
| DM121 | *Laccaria squarrosa* | Mexico: Veracruz | *Fagus grandifolia* | MF669960 | MF669967 | - | - |
| DM63 | *Laccaria squarrosa* TYPE | Mexico: Veracruz | *Fagus grandifolia* | MF669958 | MF669965 | - | - |
| DM93 | *Laccaria squarrosa* | Mexico: Veracruz | *Fagus grandifolia* | MF669959 | MF669966 | - | - |
| VB4678 | *Laccaria roseoalbescens* | Mexico | *Quercus, Carpinus* | KJ590509 | KJ590510 | - | - |
| PG03 | *Laccaria roseoalbescens* | Mexico | *Quercus, Carpinus* | KJ874329 | KJ874332 | - | - |
| LM5042 | *Laccaria roseoalbescens* | Mexico | *Quercus, Carpinus* | KJ874327 | KJ874330 | - | - |
| LM5099 | *Laccaria roseoalbescens* | Mexico | *Quercus, Carpinus* | KJ874328 | KJ874331 | - | - |
| VB4677 | *Laccaria roseoalbescens* | Mexico | *Quercus, Carpinus* | KJ590508 | KJ590511 | - | - |
| GMM6016 | *Laccaria ohiensis* | CostaRica, San Jose | *Quercus* | **MT279230** | **MT279209** | **MT431184** | **MT436073** |
| Corrales_27 | *Laccaria stellata* | Panama | *Oreomunnea mexicana* | **MT279231** | **MT279210** | **MT431185** | - |
| Corrales_576 | *Laccaria stellata* | Panama | *Oreomunnea mexicana* | **MT279232** | **-** | - | - |
| Corrales_467 | ***Laccaria nitrophila*** | Panama | *Oreomunnea mexicana* | **MT279233** | **-** | - | - |
| Corrales_547 | ***Laccaria nitrophila*** | Panama | *Oreomunnea mexicana* | **MT279234** | **-** | - | - |
| Corrales_423 | ***Laccaria nitrophila*** | Panama | *Oreomunnea mexicana* | **MT279235** | **-** | - | - |
| Corrales_595 | ***Laccaria nitrophila*** | Panama (TYPE) | *Oreomunnea mexicana* | **MT279236** | **MT279211** | **MT431186** | **MT436074** |
| Corrales_646 | ***Laccaria dallingii*** | Panama | *Oreomunnea mexicana* | **MT279237** | **MT279212** | **-** | **MT436075** |
| Corrales_543 | ***Laccaria dallingii*** | Panama | *Oreomunnea mexicana* | **MT279238** | **MT279213** | **MT431187** | **MT436076** |
| Corrales_457 | ***Laccaria dallingii*** | Panama | *Oreomunnea mexicana* | **MT279239** | **-** | - | - |
| Corrales_571 | ***Laccaria dallingii*** | Panama (TYPE) | *Oreomunnea mexicana* | **MT279240** | **MT279214** | **-** | - |
| Corrales_397 | ***Laccaria dallingii*** | Panama | *Oreomunnea mexicana* | **MT279241** | - | - | - |
| Corrales_262 | ***Laccaria dallingii*** | Panama | *Oreomunnea mexicana* | **MT279242** | **-** | - | - |
| Ovrebo 5293 | ***Laccaria dallingii*** | Panama | *Oreomunnea mexicana* | **MT279243** | **-** | - | - |
| Corrales_268 | *Laccaria* PAN3 | Panama | *Oreomunnea mexicana* | **MT279244** | **MT279215** | **MT431188** | **MT436077** |
| Corrales_25 | ***Laccaria*** [***fortunensis***](http://www.mycobank.org/BioloMICSDetails.aspx?Rec=579956) | Panama | Oreomunnea mexicana | **MT279245** | **-** | - | - |
| Corrales_74 | ***Laccaria*** [***fortunensis***](http://www.mycobank.org/BioloMICSDetails.aspx?Rec=579956) | Panama (TYPE) | Oreomunnea mexicana | **MT279246** | **-** | - | - |
| Corrales_75 | ***Laccaria*** [***fortunensis***](http://www.mycobank.org/BioloMICSDetails.aspx?Rec=579956) | Panama | Oreomunnea mexicana | **MT279247** | **-** | - | - |
| Corrales_274 | *Laccaria* aff. *roseoalbescens2* | Panama | Oreomunnea mexicana | **MT279248** | **MT279216** | **-** | **MT436078** |
| Corrales_70 | *Laccaria* aff. *roseoalbescens2* | Panama | Oreomunnea mexicana | **MT279249** | **MT279217** | **-** | **MT436079** |
| Ovrebo5332 | *Laccaria* aff. *roseoalbescens1* | Panama | Oreomunnea mexicana | **MT279250** | **MT279218** | **-** | **MT436080** |
| Corales_676A | *Laccaria* sp. | Panama | Oreomunnea mexicana | - | **MT279219** | - | - |
| O_mexicana_roots | *Laccaria* sp. | Panama | Oreomunnea mexicana | KM595030 | - | - | - |
| O_mexicana_roots | *Laccaria* sp. | Panama | Oreomunnea mexicana | KM594869 | - | - | - |
| O_mexicana_roots | *Laccaria* sp. | Panama | Oreomunnea mexicana | KM594870 | - | - | - |
| O_mexicana_roots | *Laccaria* sp. | Panama | Oreomunnea mexicana | KM594892 | - | - | - |
| O_mexicana_roots | *Laccaria* sp. | Panama | Oreomunnea mexicana | KM594912 | - | - | - |
| O_mexicana_roots | *Laccaria* sp. | Panama | Oreomunnea mexicana | KM594925 | - | - | - |
| O_mexicana_roots | *Laccaria* sp. | Panama | Oreomunnea mexicana | KM595045 | - | - | - |
| O_mexicana_roots | *Laccaria* sp. | Panama | Oreomunnea mexicana | KM594950 | - | - | - |
| O_mexicana_roots | *Laccaria* sp. | Panama | Oreomunnea mexicana | KM594954 | - | - | - |
| O_mexicana_roots | *Laccaria* sp. | Panama | Oreomunnea mexicana | KM595048 | - | - | - |
| O_mexicana_roots | *Laccaria* sp. | Panama | Oreomunnea mexicana | KM594962 | - | - | - |
| O_mexicana_roots | *Laccaria* sp. | Panama | Oreomunnea mexicana | KM594997 | - | - | - |
| O_mexicana_roots | *Laccaria* sp. | Panama | Oreomunnea mexicana | KM595049 | - | - | - |
| O_mexicana_roots | *Laccaria* sp. | Panama | Oreomunnea mexicana | KM594982 | - | - | - |
| O_mexicana_roots | *Laccaria* sp. | Panama | Oreomunnea mexicana | KM594835 | - | - | - |
| O_mexicana_roots | *Laccaria* sp. | Panama | Oreomunnea mexicana | KM594848 | - | - | - |
| O_mexicana_roots | *Laccaria* sp. | Panama | Oreomunnea mexicana | KM594856 | - | - | - |
| GMM7402 | *Laccaria* Q1 | Queensland | Allocasuarina, Eucalyptus | KU685688 | KU685832 | KU685974 | KU686106 |
| GMM7403 | *Laccaria* Q1 | Queensland | Allocasuarina, Eucalyptus | KU685689 | KU685833 | KU685975 | KU686107 |

Figure S1.

ITS only dataset using RAxML analysis with 1000 bootstrap replicates. NTAX = 166. Colored terminals identify *Laccaria* species from Panama. Blue *L.*

[*fortunensis*](http://www.mycobank.org/BioloMICSDetails.aspx?Rec=579956); purple *L. dallingii*; orange *L. nitrophilous*; red *L. roseoalbescens*; dark green *L. stellata*; light green *Laccaria* ECM from *Oreomunnea mexicana* roots.

Figure S2.

28S only dataset using RAxML analysis with 1000 bootstrap replicates. NTAX = 110. Colored terminals identify *Laccaria* species from Panama. Blue *L. fortunensis*; purple *L. dallingii*; orange *L. nitrophilous*; red *L. roseoalbescens*; green *L. stellata*.

Figure S3.

RPB2 only dataset using RAxML analysis with 1000 bootstrap replicates. NTAX = 83. Colored terminals identify *Laccaria* species from Panama. Blue *L. fortunensis*; purple *L. dallingii*; orange *L. nitrophilous*; red *L. roseoalbescens*; green *L. stellata*.

Figure S4.

EF1a only dataset using RAxML analysis with 1000 bootstrap replicates. NTAX = 58. Colored terminals identify *Laccaria* species from Panama. Blue *L. fortunensis*; purple *L. dallingii*; orange *L. nitrophilous*; red *L. roseoalbescens*.

*Laccaria* of Panama Combined Dataset. Aligned in FASTA gapped format.

>A0051_Laccaria_sp_JAPAN_KU685613___

CCTGA-TGTGACTGTTAGCTGGC-TTTTCGAAGCATGTGCTCGT-CCATCATCTTTAATCTCT--CCA-CCTGTGCACATTTTGTAGTC-TT-GGATACCTCTCGAGGCAA-CTCGGATT----TTAG-GATCGCCG-----------------TCGGCTTTTCTTTCATTTTC-AAGACTATGTTCT----T--A-TATACA-CCAAAGTATGTTTAAAGAATGTCATC-AATAGGAAC---TTGTTTCCTA--T-AAAATTATACAACTTTCAGCAACGGATCTCTTGGCTCTCGCATCGATGAAGAACGCAGCGAAATGCGATAAGTAATGTGAATTGCAGAATTCAGTGAATCATCGAATCTTTGAACGCACCTTGCGCTCCTTGGTATTCCGAGGAGCATGCCTGTTTGAGTGTCATTAAA-TTCTCAA-CCTTCCAAC--------T-TTCATTAGCTTGG--TTAGGCTTGGA-TGT-GGGGG--TTGCGGGCTTC-AT---------CACGAAGTCGGCTCTCCTTAAATGCATTAGCGGAACTTTTGTGGACCGTCTA-TTGGTGTGAT-AATTATCTACGCCGTGGAT-GTGAAGCAG-CTTTAT---GAAGTT-CAGCTTCT-AACCGTCC---ATTGACTTGGACAA-----TTTTGACAA-TTTGA-------------------------------------------------------------------------------------------------------------------------------------------------------------------------------------------------------------------------------------------------------------------------------------------------------------------------------------------------------------------------------------------------------------------------------------------------------------------------------------------------------------------------------------------------------------------------------------------------------------------------------------------------------------------------------------------------------------------------------------------------------------------------------------------------------------------------------------------------------------------------------------------------------------------------------------------------------------------------------------------------------------------------------------------------------------------------------------------------------------------------------------------------------------------------------------------------------------------------------------------------------------------------------------------------------------------------------------------------------------------------------------------------------------------------------------------------------------------------------------------------------------------------------------------------------------------------------------------------------------------------------------------------------------------------------------------------------------------------------------------------------------------------------------------------------------------------------------------------------------------------------------------------------------------------------------------------------------------------------------------------------------------------------------------------------------------------------------------------------------------------------------------------------------------------------------------------------------------------------------------------------------------------------------------------------------------------------------------------------------------------------------------------------------------------------------------------------------------------------------------------------------------------------------------------------------------------------------------------------------------------------------------------------------------------------------------------------------------------------------------------------------------------------------------------------------------------------------------------------------------------------------------------------------------------------

>A0544_Laccaria_japonica_JAPAN_JN942802_JN939755_JN993509_

CCTGA-TGTGGCTGTTAGCTGGC-TTTTCGAAGCATGTGCTCGT-CCATCATCTTT-ATCTCT--CCA-CCTGTGCACATTTTGTAGTC-TT-GGATACCTCTCGAGGCAA-CTCGGATT----TTAG-GATCGCCG------------------TGGCTTTCCTTTCATTTCC-AAGACTATGTTTT----T--A-TATACA-CCAAAGTATGTTTAAAGAATGTTATC-AATAGGAAC---TTGTTTCCTA--T-AAAATTATACAACTTTCAGCAACGGATCTCTTGGCTCTCGCATCGATGAAGAACGCAGCGAAATGCGATAAGTAATGTGAATTGCAGAATTCAGTGAATCATCGAATCTTTGAACGCACCTTGCGCTCCTTGGTATTCCGAGGAGCATGCCTGTTTGAGTGTCATTAAA-TTCTCAA-CCTTCCAGC----TTTTT-TTTATTAGCTTGG--TTTGGCTTGGA-TGT-GGGTG--TTGCAGGCTTC-AT-----T---AATGAGGTCGGCTCTCCTTAAATGCATTAGCGGAACTTTTGTGGACCGTCTA-TTGGTGTGAT-AATTATCTACGCCGTGGAT-GTGAAGCAG-CTATAT-ATGAAGTT-CAGCTTCT-AACCGTCC---ATTGACTTGGACAA----TTTTTGACAA-TTTGAAAGCTCAAATTTAAAATCTGGCAGT-CTTTGGCTGT-CCGAGTTGTAATCTAGAGAAGTATTATCCGCGCTGGACCGTGTACAAGTCTCCTGGAATGGGGCGTCATAGAGGGTGAGAATCCCGTCTTTGACACGGACTGCCAGGGC-TTTGTGATGTGCTCTCAAAGAGTCGAGTTGTTTGGGAATGCAGCTCAAAATGGGTGGTAAATTCCATCTAAAGCTAAATATTGGCGAGAGACCGATAGCGAACAAGTACCGTGAGGGAAAGATGAAAAGAACTTTGGAAAGAGAGTTAAACAGTACGTGAAATTGCTGAAAGGGAAACGCTTGAAGTCAGTCACGTTGGCCAGGGATCAA-CCTTGC----TTTTTT---GCTTGGCTTACTTTCTGGTTGATGGG-TCAGCATCAATTTTGACTGGTGG-AAAAAGTTCAAGGGAATGTGGCAT-CTTCGGATGTGTTATAGCCTTTGTTCGCATACACTGGTTGGGATTGAGGAACTCAGCATGCCGCAAGGCCGGG---TTTTTAACCACGTACGTGCTTAGGATGCTGGCATAATGGCTTTAATCGACCCGTCTTGAAACACGGACCAAGGAGTCTAACATGCCTGCGAGTGTTTGGGTGAAAAACCCGAGCGCGTAATGAAAGTG-AAAGTTGAGATCCCTGTCGTGGGGAGCATTGACGCCCGGATCTGACCTTTTGTGACGATTCTGCGGTAGAGCATGTTTTCCGTATGCTTTTCCGGAAGTTAACCAAGGACGTTTATCGTTATCTACAAAAGGTTGTTGACCTTCGTTCAAGTGTC-----ATGAGATGTGCTGA-CCCC------TCTTCAGTGCGTCGAGACACACAAGGAATTCAACCTTGCCCTTGCAGTTAAGCACCAAACCATCACAAACGGCCTCAAATACTCTCTGGCGACAGGTAACTGGGGAGATCAGAAAAAGTCAATGTCTTCCAAGGCAGGAGTATCTCAGGTCTTGAACAGATACACATATGCATCCACCCTGTCACATCTTCGTCGGTGTAACACCCCTCTAGGGCGCGAGGGCAAGATCGCCAAACCTCGTCAGTTGCACAACACCCATTGGGGTATGGTGTGTCCTGCGGAAACTCCTGAAGGTCAAGCCTGTGGTCTTGTCAAGAACCTCGCCCTTATGGCGTGTATATCAGTCGGGTCTTACTCCGCGCCCGTTATCGAGTTTTTGGAGGAGTGGGGATTGGAGTCATTGGAAGAAAACGCACATTCATCGACACCTTGCACAAAAGTTTTCGTGAACGGTGTCTGGATGGGTGTGCATCGCGATCCTGCCAATTTGGTGAAGACGATAAAGAAATTGAGAAGGAAAGACGACATCAGCCCGGAAGTGTCAGTTGTGCGAGATATCAGGGAGAAGGAATTGAGGTTGTACACCGATGCTGGACGTGTTTGTCGACCACTCTTCATCGTCGAGAACCAACAGCTGGCACTTCAGAAGAAGCACGTTAAATGGCTCAGTAACGGCGTCAACGATGATGGCGACGAGTACAAATGGGAACATCTGGTAAAGGGTGGCATCATTGAGTTACTGGATGCTGAGGAAGAGGAAACGGTGATGATATCCATGACCCCTGAAGATCTTGAAAATTCTCGCCTACAGCAAAGTGGTGTTGACCCTCACGCGAACGACGGCGAGTTTGATCCAGCAGCTCGATTGAAGGCTGGTACTCACGCACACACATGGACACATTGCGAAATTCATCCTAGCATGATTCTTGGCATTTGTGCCAGCATTATTCC-TTCCCTGATCAT------------------------------------------------------------------------------------------------------------------------------------------------------------------------------------------------------------------------------------------------------------------------------------------------------------------------------------------------------------------------------------------------------------------------------------------------------------------------------------------------------------------------------------------------------------------------------------------------------------------------------------------------------------------------------------------------------------------------------------------------------------------------------------------------------------------------------------------------------------------------------------------------------------------------------------------------------------------------------------------------------------------------------------------------

>A0578_Laccaria_japonica_JAPAN_JN942783___

CCTGA-TGTGGCTGTTAGCTGGC-TTTTCGAAGCATGTGCTCGT-CCATCATCTTT-ATCTCT--CCA-CCTGTGCACATTTTGTAGTC-TT-GGATACCTCTCGAGGCAA-CTCGGATT----TTAG-GATCGCCG------------------TGGCTTTCCTTTCATTTCC-AAGACTATGTTTT----T--A-TATACA-CCAAAGTATGTTTAAAGAATGTTATC-AATAGGAAC---TTGTTTCCTA--T-AAAATTATACAACTTTCAGCAACGGATCTCTTGGCTCTCGCATCGATGAAGAACGCAGCGAAATGCGATAAGTAATGTGAATTGCAGAATTCAGTGAATCATCGAATCTTTGAACGCACCTTGCGCTCCTTGGTATTCCGAGGAGCATGCCTGTTTGAGTGTCATTAAA-TTCTCAA-CCTTCCAGC----TTTTT-TTTATTAGCTTGG--TTTGGCTTGGA-TGT-GGGTG--TTGCAGGCTTC-AT-----T---AATGAGGTCGGCTCTCCTTAAATGCATTAGCGGAACTTTTGTGGACCGTCTA-TTGGTGTGAT-AATTATCTACGCCGTGGAT-GTGAAGCAG-CTATAT-ATGAAGTT-CAGCTTCT-AACCGTCC---ATTGACTTGGACAA----TTTTTGACAA-TTTGA-------------------------------------------------------------------------------------------------------------------------------------------------------------------------------------------------------------------------------------------------------------------------------------------------------------------------------------------------------------------------------------------------------------------------------------------------------------------------------------------------------------------------------------------------------------------------------------------------------------------------------------------------------------------------------------------------------------------------------------------------------------------------------------------------------------------------------------------------------------------------------------------------------------------------------------------------------------------------------------------------------------------------------------------------------------------------------------------------------------------------------------------------------------------------------------------------------------------------------------------------------------------------------------------------------------------------------------------------------------------------------------------------------------------------------------------------------------------------------------------------------------------------------------------------------------------------------------------------------------------------------------------------------------------------------------------------------------------------------------------------------------------------------------------------------------------------------------------------------------------------------------------------------------------------------------------------------------------------------------------------------------------------------------------------------------------------------------------------------------------------------------------------------------------------------------------------------------------------------------------------------------------------------------------------------------------------------------------------------------------------------------------------------------------------------------------------------------------------------------------------------------------------------------------------------------------------------------------------------------------------------------------------------------------------------------------------------------------------------------------------------------------------------------------------------------------------------------------------------------------------------------------------------------------------------

>A2986_Laccaria_vinaceoalvanea_JAPAN_JN942810_JN939738_JN993520_

-CTGA-TGTGGCTGTTAGCTGGC-TTTTCGAAGCATGTGCTCGC-CCATCATCTTT-ATCTCT--CCA-CCTGTGCACCTTTTGTAGTC-TT-GAATACCTCTCGAGGCAA-CTCGGATT----TTAG-GATTGCCGTGC-TTTATACACAAAGTCGGCTTTTCTTTCATTTTC-AAGACTATGTTTT---CT--A-TACACA-CCAAAGTATGTTTAAAGAACGTCATC-AATAGGAAC---TTGTTTCCTA--TAAAAACTATACAACTTTCAGCAACGGATCTCTTGGCTCTCGCATCGATGAAGAACGCAGCGAAATGCGATAAGTAATGTGAATTGCAGAATTCAGTGAATCATCGAATCTTTGAACGCACCTTGCGCTCCTTGGTATTCCGAGGAGCATGCCTGTTTGAGTGTCATTAAA-TTCTCAACCTTTCCAGC--------T-TTTATTAGTTTGG--TCAGGCTTGGA-TGT-GGGGG--TTGCAGGCTTC-AT-----T-------TAGTCTGCTCTCCTTAAATRCATTAGCGGAACTTTTGTGGACCGTCTA-TTGGTGTGAT-AATTATCTACACCGTGGAT-GTAAAGCAGTTTTTAT-AAAAAGTT-CAGCTCCT-AACTGTCC---ATTGAC-TGGACAA-----TTTTGACAA--TTGAAAGCTCAAATTTAAAATCTGGCAGT-CTTTGGCTGT-CCGAGTTGTAATCTAGAGAAGCATTATCCGCGCTGGACCGTGTACAAGTCTCCTGGAATGGAGCGTCATAGAGGGTGAGAATCCCGTCTTTGACACGGACTACCAGGGCTTTTGTGATGTGCTCTCAAAGAGTCGAGTTGTTTGGGAATGCAGCTCAAAATGGGTGGTAAATTCCATCTAAAGCTAAATATTGGCGAGAGACCGATAGCGAACAAGTACCGTGAGGGAAAGATGAAAAGAACTTTGGAAAGAGAGTTAAACAGTACGTGAAATTGCTGAAAGGGAAACGCTTGAAGTCAGTCGCGTTGGCCAGGGATCAA-CCTTGC----TTTTTT---GCTTGGCTTATTTTCTGGTCAATGGG-TCAGCATCAATTTTGACTGGTGG-AAAAAGTTCAAGGGAATGTGGCAT-CTTTGGATGTGTTATAGCCTTTGTTCGCATACATCAGTTGGGATTGAGGAACTCAGCACGCCGAAAGGCCGGG---TTTTTAACCACGTACGTGCTTAGGATGCTGGCATAATGGCTTTAATCGACCCGTCTTGAAACACGGACCAAGGAGTCTAACATGCCTGCGAGTGTTTTGGTGAAAAACCAGAGTGCATAATGAAAGTG-AAAGTTGAGATCCCTGTCGTGGGGAGCATCGACGCCCAGATCTGACCTTTTGTGACGATTCTGCGGTAGAGCATGTTTTTCGCATGCTTTTCCGAAAGTTGACCAAGGACGTTTATCGATATCTACAAAAGGTTGCTGAGCTTTGCCCAAGTGCC-----ATGAGATGTGCTGA-CCCCTCT---TCTTCAGTGCGTCGAGACCCACAAGGAATTCAATCTTGCTCTCGCAGTGAAGCACCAAACCATCACAAACGGCCTCAAATACTCTCTGGCGACAGGTAACTGGGGGGATCAGAAAAAGTCAATGTCTTCCAAGGCAGGGGTATCTCAGGTCTTGAACAGATACACATATGCATCCACTCTGTCACATCTTCGTCGATGTAACACTCCTCTAGGACGCGAAGGCAAGATCGCCAAACCTCGTCAGTTGCACAATACTCATTGGGGTATGGTGTGTCCTGCGGAAACTCCTGAAGGTCAAGCCTGTGGTCTTGTCAAGAACCTAGCTCTTATGGCATGCATATCAGTCGGGTCTTACTCCGCACCCGTTATTGAGTTTTTGGAGGAGTGGGGGTTGGAGTCATTGGAAGAGAACGCACACTCAACGACACCTTGCACAAAAGTTTTCGTGAACGGTGTCTGGATGGGTGTGCACCGTGATCCTGCCAACTTGGTGAAGACGATAAAGAAATTGAGAAGGAAAGATGACATCAGCCCAGAAGTGTCTGTTGTGCGAGACATCAGGGAGAAGGAATTGAGGTTGTATACCGATGCTGGACGTGTTTGTCGACCACTCTTCATCGTCGAGAACCAACAGTTGGCACTTCAGAAGAAGCACGTCAAGTGGCTCAGCAACGGCCTCAATGATGATGGCGACGAGTATAAATGGGAACATCTGGTAAAGGGTGGCATCATTGAGTTACTGGATGCGGAGGAAGAGGAAACAGTGATGATATCCATGACCCCTGAAGATCTTGAAAATTCCCGCCTACAGCAAAGTGGCGTTGATCCTCACGCGAACGACGGCGAGTTTGATCCAGCAGCTCGATTGAAGGCTGGCACTCACGCACACACATGGACACATTGCGAGATTCATCCTAGCATGATTCTTGGCATTTGTGCCAGCATTATTCC-TTCCCTGATCAT------------------------------------------------------------------------------------------------------------------------------------------------------------------------------------------------------------------------------------------------------------------------------------------------------------------------------------------------------------------------------------------------------------------------------------------------------------------------------------------------------------------------------------------------------------------------------------------------------------------------------------------------------------------------------------------------------------------------------------------------------------------------------------------------------------------------------------------------------------------------------------------------------------------------------------------------------------------------------------------------------------------------------------------------

>A2987_Laccaria_sp_JAPAN_JN942786_JN939739_JN993521_

CCTGA-TGTGACTGTTAGCTGGC-TTTTCAAAGCATGTGCTCGT-CCGTCATCTTTAATCTCT-CCCA-CCTGTGCACATTTTGTAGTC-TT-GAATACCTCTCGAGGCAA-CTCGGATT----TTAG-GATCGC---------------CAAGTTGGCTTTCCTTTCACTTCC-AAGACTATGTTTT----T--A-TATACA-CCAAAGTATGTTTAAAGAATGTCATT-AATAGGAAC---TTGTTTCCTA--T-AAAATTATACAACTTTCAGCAACGGATCTCTTGGCTCTCGCATCGATGAAGAACGCAGCGAAATGCGATAAGTAATGTGAATTGCAGAATTCAGTGAATCATCGAATCTTTGAACGCACCTTGCGCTCCTTGGTATTCCGAGGAGCATGCCTGTTTGAGTGTCATTAAA-TTCTCAA-CCTTCCAGC--------T-TTTATAAGCTTGG--TTAGGCTTGGA-TGT-GGGAG--TTGCGGGCTTC-AT-----C---AATGAGGTCGGCTTTCCTTAAATGCATTAGCGGAACTTTTGTGGACCGTCTA-TTGGTGTGAT-AATTATCTACGCCGTGGAT-GTGAAACAG-CTTTAT---GAAGTT-CAGCTTCG-AACCGTCC---ATTCACTTGGACAA-----TTTTGACAA-TTTGAAAGCTCAAATTTAAAATCTGGCAGT-CTTTGGCTGT-CCGAGTTGTAATCTAGAGAAGTATTATCCGCGCTGGACCGTGTACAAGTCTCCTGGAATGGAGCGTCATAGAGGGTGAGAATCCCGTCTTTGACACGGACTGCCAGGGC-TTTGTGATGTGCTCTCAAAGAGTCGAGTTGTTTGGGAATGCAGCTCAAAATGGGTGGTAAATTCCATCTAAAGCTAAATATTGGCGAGAGACCGATAGCGAACAAGTACCGTGAGGGAAAGATGAAAAGAACTTTGGAAAGAGAGTTAAACAGTACGTGAAATTGCTGAAAGGGAAACGCTTGAAGTCAGTCACGTTGGCCAGGGATCAA-CCTTGC----TTTTTT---GCTTGGCTTACTTTCTGGTCGATGGG-TCAGCATCAATTTTGACTGGTAG-AAAAAGTTCAAGGGAATGTGGCAT-CTTCGGATGTGTTATAGCCTTTGTTCACATATATCAGTTGGGATTGAGGAACTCAGCACGCCGCAAGGCCGGG---TTTTTAACCACGTACGTGCTTAGGATGCTGGCATAATGGCTTTAATCGACCCGTCTTGAAACACGGACCAAGGAGTCTAACATGCCTGCGAGTGTTTGGGTGAAAAACCCGAGCGCGTAATGAAAGTG-AAAGTTGAGATCCCTGTCGTGGGGAGCATTGACGCCCGGATCTGACCTTTTGTGACGATTCTGCGGTAGAGCATGTTTTCCGCATGCTTTTCCGAAAGTTGACCAAGGACGTTTATCGTTACCTACAAAAGGTTGCTGACCTTCGCTCAAGTGCT-----GTGAGATGTGCTGA-CCTCTCT---TCTTCAGTGCGTCGAAACCCACAAGGAGTTCAACCTTGCCCTTGCAGTTAAGCACCAAACCATCACAAACGGCCTCAAATACTCTCTGGCGACAGGTAACTGGGGAGACCAGAAAAAATCAATGTCTTCCAAGGCAGGAGTATCTCAGGTCTTGAACAGATACACATATGCATCCACTCTGTCACATCTTCGTCGGTGTAACACCCCTCTAGGGCGCGAAGGCAAGATCGCCAAACCTCGTCAGTTGCACAACACTCATTGGGGTATGGTGTGTCCTGCGGAAACTCCTGAAGGTCAAGCCTGTGGGCTTGTCAAGAACCTCGCTCTTATGGCGTGCATATCAGTCGGGTCTTACTCCGCGCCCGTTATCGAGTTTTTGGAGGAGTGGGGATTGGAGTCATTGGAAGAGAACGCGCACTCATCAACACCTTGCACAAAAGTTTTCGTGAACGGTGTGTGGATGGGTGTGCATCGCGATCCTGCCAACTTGGTGAAGACGATAAAGAAATTGAGAAGAAAAGACGACATTAGTCCAGAAGTGTCAGTTGTGCGAGACATCAGGGAGAAAGAACTGAGGTTGTACACAGACGCTGGACGTGTTTGTCGACCACTCTTCATCGTCGAGAACCAACAGCTGGCACTTCAGAAGAAGCACGTCAAATGGCTCAGTAACGGCCTCAATGATGATGGCGATGAGTACAAATGGGAACATCTGGTAAAGGGTGGCATCATTGAGTTACTGGATGCTGAGGAAGAGGAAACAGTGATGATATCCATGACCCCTGAAGATCTTGAAAATTCTCGCTTACAACAAAGTGGTGTTGATCCTCACGCGAATGACGGCGAGTTTGATCCAGCAGCTCGTTTGAAGGCTGGCACTCACGCGCACACATGGACACATTGCGAAATTCATCCTAGCATGATTCTTGGCATTTGTGCCAGCATTATTCC-TTCCCTGATCAT------------------------------------------------------------------------------------------------------------------------------------------------------------------------------------------------------------------------------------------------------------------------------------------------------------------------------------------------------------------------------------------------------------------------------------------------------------------------------------------------------------------------------------------------------------------------------------------------------------------------------------------------------------------------------------------------------------------------------------------------------------------------------------------------------------------------------------------------------------------------------------------------------------------------------------------------------------------------------------------------------------------------------------------------

>A3344_Laccaria_amethystina_CHINA_KU685627___

CCTGA-TGTGGCTGTTAGCTGGC-TTTTCGGAGCATGTGCTCGT-CCGTCATCTTT-ATCTCT--CCA-CCTGTGCACATTTTGTAGTC-TT-GGATACCTCTCGAGGCAA-CTCGGATT----TTAG-GATCGCTGTGC---TGT---ACAAGTCAGCTTTCCTTTCATTTCC-AAGACTATGTTTT-------A-TATACACCCAAAGTATGTTTAAAGAATGTCATC-AATAGGAAC---TTGTTTCCTA--T-AAAATTATACAACTTTCAGCAACGGATCTCTTGGCTCTCGCATCGATGAAGAACGCAGCGAAATGCGATAAGTAATGTGAATTGCAGAATTCAGTGAATCATCGAATCTTTGAACGCACCTTGCGCTCCTTGGTATTCCGAGGAGCATGCCTGTTTGAGTGTCATTAAA-TTCTCAA-CCTTCCAGC--------T-TTTATTAGCTTGG--TTAGGCTTGGA-TGT-GGGAG--TTGTGGGCTTC-AT-----T---AATGAGGTCAGCTCTCCTTAAATGCATTAGCGGAACTTTTGTGGACCGTCTA-TTGGTGTGAT-AATTATCTACGCCGTGGAT-GTGAAGCAGCTTTTAT---GGAGTT-CAGCTTCT-AACTGTCC---ATTGACTTGGACAA---ATTTTTGACAA-TTTGA-------------------------------------------------------------------------------------------------------------------------------------------------------------------------------------------------------------------------------------------------------------------------------------------------------------------------------------------------------------------------------------------------------------------------------------------------------------------------------------------------------------------------------------------------------------------------------------------------------------------------------------------------------------------------------------------------------------------------------------------------------------------------------------------------------------------------------------------------------------------------------------------------------------------------------------------------------------------------------------------------------------------------------------------------------------------------------------------------------------------------------------------------------------------------------------------------------------------------------------------------------------------------------------------------------------------------------------------------------------------------------------------------------------------------------------------------------------------------------------------------------------------------------------------------------------------------------------------------------------------------------------------------------------------------------------------------------------------------------------------------------------------------------------------------------------------------------------------------------------------------------------------------------------------------------------------------------------------------------------------------------------------------------------------------------------------------------------------------------------------------------------------------------------------------------------------------------------------------------------------------------------------------------------------------------------------------------------------------------------------------------------------------------------------------------------------------------------------------------------------------------------------------------------------------------------------------------------------------------------------------------------------------------------------------------------------------------------------------------------------------------------------------------------------------------------------------------------------------------------------------------------------------------------------------------

>ALB183_Laccaria_amethystina_CHINA_TIBET_JX504092_JX504176_KU686058_KU686161

CCTGA-TGTGACTGTTAGCTGGC-TTTTCGAAGCATGTGCTCGT-CCGTCATCTTT-ATCTCT--CCA-CCTGTGCACATTTTGTAGTC-TT-GGATACCTCTCGAGGCAA-CTCGGATT----TTAG-GATCGCTGTGC---TGT---ACAAGTTGGCTTTCCTTTCATTTCC-AAGACTATGTTTT----T--A-TATACA-CCAAAGTATGTTTAAAGAATGTTATC-AATAGGAAC---TTGTTTCCTA--T-AAAATTATACAACTTTCAGCAACGGATCTCTTGGCTCTCGCATCGATGAAGAACGCAGCGAAATGCGATAAGTAATGTGAATTGCAGAATTCAGTGAATCATCGAATCTTTGAACGCACCTTGCGCTCCTTGGTATTCCGAGGAGCATGCCTGTTTGAGTGTCATTAAA-TTCTCAA-CCTTCCAGC-------TT-TTTATTAGCTTGG--TTTGGCTTGGA-TGT-GGGGG--TTGCAGGCTTC-AT-----T---AATGAGGTCGGCTCTCCTTAAATGCATTAGCGGAACTTTTGTGGACCGTCTA-TTGGTGTGAT-AATTATCTACGCCGTGGAT-GTGAAGCAG-CTTTAT-ATGAAGTT-CAGCTTCT-AACCGTCC---ATTGACTTGGACAA----TTTTTGACAA-TTTGAAAGCTCAAATTTAAAATCTGGCAGT-CTTTGGCTGT-CCGAGTTGTAATCTAGAGAAGTATTATCCGCGCTGGACCGTGTACAAGTCTCCTGGAATGGGGCGTCATAGAGGGTGAGAATCCCGTCTTTGACACGGACTGCCAGGGC-TTTGTGATGTGCTCTCAAAGAGTCGAGTTGTTTGGGAATGCAGCTCAAAATGGGTGGTAAATTCCATCTAAAGCTAAATATTGGCGAGAGACCGATAGCGAACAAGTACCGTGAGGGAAAGATGAAAAGAACTTTGGAAAGAGAGTTAAACAGTACGTGAAATTGCTGAAAGGGAAACGCTTGAAGTCAGTCGCGTTGGCCAGGGATCAA-CCTTGC-----TTTTT---GCTTGGCTTACTTTCTGGTTAATGGG-TCAGCATCAATTTTGACTGGTGG-AAAAAGTTCAAGGGAATGTGGCAT-CTTCGGATGTGTTATAGCCTTTGTTCGCATACATTGGTTGGGATTGAGGAACTCAGCACGCCGCAAGGCCGGG---TTTTTAACCACGTACGTGCTTAGGATGCTGGCATAATGGCTTTAATCGACCCGTCTTGAAACACGGACCAAGGAGTCTAACATGCCTGCGAGTGTTTGGGTGAAAAACCCGAGCGCGTAATGAAAGTG-AAAGTTGAGATCCCTGTCGTGGGGAGCATTGACGCCCGGATCTGACCTTTTGTGACGATTCTGCGGTAGAGCATGTTTTCcGCATGCTTTTCcGgAaGTTAaCcAaGGACGTTTATCGCTATTTACAAAAGGTTGCTGACCTTCGTTTAaGTGCC-----ATGAGATGTGCTGA-CCCCTTT---TTTTCAGTGCGTCGAGACCCACAAGGAGTTCAACCTTGCCcTTGCAGTTAAGCATCAAACCATCACAAATGGCCTCAAATACTCTCTGGCGACAGGTAACTGGGGTGATCAGAAAAAGTCAATGTCTTCCAAGGCAGGAGTATCTCAGGTCTTGAACAGATACACATATGCATCCACCTTGTCACATCTTCGTCGGTGTAACACTCCTCTAGGGCGCGAGGGCAAGATCGCCAAACCTCGTCAGTTGCACAACACTCATTGGGGTATGGTGTGTCCTGCGGAAACTCCTGAAGGTCAAGCCTGTGGTCTTGTCAAGAACCTCGCCCTTATGGCGTGCATATCAGTCGGGTCTTATTCCGCGCCCGTTATCGAGTTTTTGGAAGAGTGGGGATTGGAGTCATTGGAAGAAAACGCACACTCATCGACACCTTGCACAAAAGTTTTCGTGAACGGTGTCTGGATGGGTGTGCATCGCGATCCTGCCAATTTGGTCAAGACGATAAAGAAATTGAGAAGGAAAGACGACATCAGCCCAGAAGTATCAGTTGTGCGAGACATTAGGGAGAAGGAACTGAGGTTGTACACCGATGCTGGACGTGTTTGTCGACCACTCTTCATCGTCGAGAACCAACAGCTGGCACTTCAGAAGAAGCACGTCAARTGGCTCAGTAACGGTCTCAACGATGATGGCGACGAGTACAAATGGGAGCACCTGGTAAAGGGTGGCATCATTGAGTTACTGGATGCTGAGGAAGAGGAAACGGTGATGATATCCATGACCCCTGAAGATCTTGAAAATTCTCGCCTACAGCAAAGTGGTGTTGATCCTCACGCGAACGACGGCGAGTTTGATCCAGCAGCTCGATTGAAGGCTGGCACTCACGCACACACATGGACACATTGCGAAATTC--------------------------------------------------------------------------------------------------------------------------------------------------------------------------------------------------------------------------------------------------------ATCTCA-TGCAGTGGAGCGAGGATCGTTTCAACGAAATCGTCAAGGAGACGTCAaCCTTCATCAAGAAGGTTGGTTACAACCCCAAGGCCGTTGCCTTTGTCCCTATTTCCGGCTGGCACGGTGACAACATGTTGGAGGAGTCCGCTAAGTGAGCATTC--TTGTATCGTGATGGTTACCCCGTCGCTCATGTTGCACTT-AGCATGCCTTGGTACAAGGGCTGGACCAAGGAAACCAAGGCTGGTGTCGTCAAGGGCAAGACCCTCCTCGATGCTATTGATGCCATTGAGCCCCCCGTCCGACCTTCCGACAAACCCCTCCGTCTGCCCCTCCAGGATGTCTACAAAATTGGCGGTATTGGAACTGTGCCCGTCGGTCGTGTTGAGACTGGTATCATCAAGGCCGGAATGGTCGTCACTTTCGCGCCCTCCAACGTCACCACTGAAGTTAAGTCCGTCGAAATGCATCACGAACAGCTCGAGCAGGGTAACCCTGGTGACAATGTCGGTTTCAACGTCAAAAACGTGTCAGTGAAGGATATTCGTCGTGGCAACGTGGCCTCCGACTCCAAGAACGATCCCGCCAAGGAAGCGGCCTCTTTCAACGCACAGGTCATCGTCCTCAACCACCCTGGTCAAATTGGTGCTGGTTACGCCCCAGTTCTCGACTGTCACACCGCCCACATCGCTTGCAAGTTCGCCGAGCTCATCGAGAAGATTGATCGTCGTACGGGTAAATCTATCGAAAACTCACCTAAATTCGTCAAGTCGGGTGACGCCTGCATCGTCAAGCTCGTTCCCAGCAAGCCTATG

>AWW251_Laccaria__MALAYSIA_JX504093_JX504177__

CCTGA-TGTGGCTGTTAGCTGGC-TTTTCGAGGCATGTGCTCGC-CCATCATCGTT-ATCTCT--CCA-CCTGTGCAC-TTTTGTAGTCTTT-GAATACCTCTCGAGGCAA-CTCGGATT----TTAG-GATTGCCGTGC-----TGTACAAAGTCGGCTTTTCTATCATTTTC-AAGACTATGTTTT----TCTA-TACACA-CCAATGTATGTTTAAAGAATGTCGTC-AATAGGAAC---TTGTTTCCTA--T-AAAATTATACAACTTTCAGCAACGGATCTCTTGGCTCTCGCATCGATGAAGAACGCAGCGAAATGCGATAAGTAATGTGAATTGCAGAATTCAGTGAATCATCGAATCTTTGAACGCACCTTGCGCTCCTTGGTATTCCGAGGAGCATGCCTGTTTGAGTGTCATTAAA-TTCTCAACCTTTCCAGA--------TCTTTATTAGTCTGG--TCAGGCTTGGA-TGT-GGGGG-TTTGCAGGTTTT------------TATGAGATCTGCTCTCCTTAAATGCATTAGTGGAACTTTTGTGGACCGTCTA-TTGGTGTGAT-AATTATCTACACCGTAGAT-GTAAAACAG-TTTTAT---AAAGTT-TAGCTTCT-AACTGTCC---ATTGACTTGGACAA-----TTTTGACAA-TTTGAAAGCTCAAATTTAAAATCTGACGGT--CTTGTCTGT-CCGAGTTGTAATCTAGAGAAGCATTATCCGCGTTGGACCGTGTACAAGTCTCCTGGAATGGAGCGTCATAGAGGGTGAGAATCCCGTCTTTAACACGGACTGCCAGGGCTTTTGTGATGTGCTCTCAAAGAGTCGAGTTGTTTGGGAATGCAGCTCAAAATGGGTGGTAAATTCCATCTAAAGCTAAATATTGGCGAGAGACCGATAGCGAACAAGTACCGTGAGGGAAAGATGAAAAGAACTTTGGAAAGAGAGTTAAACAGTACGTGAAATTGCTGAAAGGGAAACGCTTGAAGTCAGTCGCGTTGGCCAGTGATCAA-CCTTGC---TTTTTTT---GCTTGGCTTATTTTCTGGTCAATGTG-TCAGCATCAATTTTGACTGGTGG-AAAAAGTTCAAAGGAATGTGGCAT-CCTTGGATGTGTTATAGCCTTTGTTCGCATACATCAGTTGGGATTGAGGAACTCAGCACGCTGAAAGGCCGGGTTTTTTTAAACCACGTACGTGCTTAGGATGCTGGCATAATGGCTTTAATCGACCCGTCTTGAAACACGGACCAAGGAGTCTAACATGCCTGCAAGTGTTTTGGTGAAAAACCAGAGCGCATAATGAAAGTG-AAAGTTGAGATCCCTGTCGTGGGGAGCATCGACGCCCAGATCTGACCTTTTGTGACGATTCTGCGGTAGAGCATGT-----------------------------------------------------------------------------------------------------------------------------------------------------------------------------------------------------------------------------------------------------------------------------------------------------------------------------------------------------------------------------------------------------------------------------------------------------------------------------------------------------------------------------------------------------------------------------------------------------------------------------------------------------------------------------------------------------------------------------------------------------------------------------------------------------------------------------------------------------------------------------------------------------------------------------------------------------------------------------------------------------------------------------------------------------------------------------------------------------------------------------------------------------------------------------------------------------------------------------------------------------------------------------------------------------------------------------------------------------------------------------------------------------------------------------------------------------------------------------------------------------------------------------------------------------------------------------------------------------------------------------------------------------------------------------------------------------------------------------------------------------------------------------------------------------------------------------------------------------------------------------------------------------------------------------------------------------------------------------------------------------------------------------------------------------------------------------------------------------------------------------------------------------------------

>AWW438_Laccaria_alba_CHINA_JX504094_JX504178_KU685912_KU686072

CCTGA-TGTGACTGTTAGCTGGC-TTTTCGAAGCATGTGCTCGT-CCATCATCTTTAATCTCT--CCA-CCTGTGCACATTTTGTAGTC-TT-GGATACCTGTCGAGGCAA-CTCGGATT---TTTAG-GATCGCCGTGC---TGT---ACAAGTCGGCTTTTCTTTCATTTCC-AAGACTATGTTTT----T--A-TATACA-CCAAAGTATGTTTAAAGAATGTCATT-AATAGGAAC---TTGTTTCCTA--T-AAAATTATACAACTTTCAGCAACGGATCTCTTGGCTCTCGCATCGATGAAGAACGCAGCGAAATGCGATAAGTAATGTGAATTGCAGAATTCAGTGAATCATCGAATCTTTGAACGCACCTTGCGCTCCTTGGTATTCCGAGGAGCATGCCTGTTTGAGTGTCATTAAA-TTCTCAA-CCTTCCAAC--------T-TTTATTAGCTTGG--ATAGGCTTGGA-TGT-GGGGG--TTGCAGGCTTC-AT-----C---AATGAGGTCAGCTCTTCTTAAATGCATTAGCGGAACTTTTGTGGACCGTCTA-TTGGTGTGAT-AATTATCTACGCCGTGGAT-GAGAAACAG-CTTTAT---CAAGTT-CAGCTTCT-AACCGTCC---ATTGACTTGGACAA-----TTTTGACAA-TTTGAAAGCTCAAATTTAAAATCTGGCAGT-CTTTGGCTGT-CCGAGTTGTAATCTAGAGAAGTATTATCCGCGCTGGACCGTGTACAAGTCTCCTGGAATGGAGCGTCATAGAGGGTGAGAATCCCGTCTTTGACACGGACTGCCAGGGC-TTTGTGATGTGCTCTCGAAGAGTCGAGTTGTTTGGGAATGCAGCTCAAAATGGGTGGTAAATTCCATCTAAAGCTAAATATTGGCGAGAGACCGATAGCGAACAAGTACCGTGAGGGAAAGATGAAAAGAACTTTGGAAAGAGAGTTAAACAGTACGTGAAATTGCTGAAAGGGAAACGCTTGAAGTCAGTCGCATTGGCCAGGGATCAA-CCTTGC----TTTTTT---GCTTGGCTTACTTTCTGGTTGATGGG-TCAGCATCAATTTTGACTGGTAG-AAAAAGTTCAAGGGAATGTGGCAT-CTTCGGATGTGTTATAGCCTTTGTTCACATATATCAGTTGGGATTGAGGAACTCAGCACGCCGCAAGGCCGGG---TTTTTAACCACGTACGTGCTTAGGATGCTGGCATAATGGCTTTAATCGACCCGTCTTGAAACACGGACCAAGGAGTCTAACATGCCTGCGAGTGTTTGGGTGAAAAACCCGAGCGCGTAATGAAAGTG-AAAGTTGAGATCCCTGTCATGGGGAGCATTGACGCCCGGATCTGACCTTTTGTGACGATTCTGCGGTAGAGCATGTTTTCCGCATGCTTTTCCGAAAGTTGACCAAGGACGTTTATCGTTATCTACAAAAGgttgctgaccttcgctcaagtYtc-----atgagatatgctga-cccctct---tcttcagTGCGTCGAGACCCACAAGGAGTTCAACCTTGCCCTTGCAGTTAAGCATCAAACCATYACAAACGGCCTCAAATACTCTCTGGCGACAGGTAACTGGGGAGATCAGAAAAAGTCAATGTCTTCCAAGGCAGGAGTATCTCAGGTYTTGAACAGATACACATATGCATCCACTCTGTCACATCTTCGTCGGTGTAACACTCCTCTAGGGCGCGAAGGCAAGATCGCCAAACCTCGTCAGTTGCACAACACTCATTGGGGTATGGTGTGTCCTGCGGAAACTCCTGAAGGTCAAGCCTGTGGTCTTGTCAAGAACCTCGCCCTTATGGCGTGCATATCAGTCGGGTCTTACTCCGCGCCCGTTATCGAGTTTTTGGAGGAGTGGGGATTGGAGTCATTGGAAGAGAACGCGCACTCATCAACACCTTGCACAAAAGTTTTCGTGAACGGTGTCTGGATGGGTGTGCATCGTGATCCTGCCAATCTGGTGAAGACGATAAAGAAATTGAGAAGGAAAGACGACATTAGCCCAGAAGTGTCAGTTGTGCGAGACATCAGGGAGAAGGAATTGAGGTTGTACACCGATGCTGGACGTGTTTGTCGACCACTCTTCATTGTCGAGAACCAACAGCTGGCACTTCAGAAGAAGCACGTCAAATGGCTCAGTAACGGCCTCAACGATGATGGCGACGAGTACAAATGGGAACATCTAGTAAAGGGTGGCATCATCGAGTTACTGGATGCTGAGGAAGAAGAAACAGTGATGATATCCATGACCCCTGAAGATCTTGAAAATTCTCGCCTACAGCAGAGTGGTGTTGATCCTCACGCGAACGACGGCGAGTTTGATCCAGCAGCTCGATTGAAGGCTGGCACTCACGCACACACATGGACACATTGCGAAATTCATCCTAGCATGATTCTTGGCATTTGTGCCAGCATTATTCCTTTCCCTGATCATCTGCGCCATTCTCATCATTGCTGGTGGCACTGGTGAGTTCGAGGCTGGTATCTCCAAGGATGGCCAGACCCGCGAGCACGCCCTCCTCGCTTTCACCCTCGGTGTGCGGCAACTCATCGTTGCCGTCAACAAGATGGACACCACTAAGGTAAGAAACGTTCCAC-CTATAGTTAGGCTTCTTACACATTCTCATCATCTCA-TGTAGTGGAGCGAGGACCGTTTCAACGAAATTATCAAGGAGACGTCAACCTTCATCAAGAAGGTTGGTTACAACCCAAAGGCTGTTGCCTTTGTCCCTATTTCCGGCTGGCACGGCGACAACATGTTGGAGGAGTCCGCTAAGTAAGCATTC--TCGTATCATCATGATCGCCCCGTCGCTCATGTTGCACACAAGCATGCCTTGGTACAAGGGCTGGACCAGGGAGACCAAGGCTGGTGTCGTCAAGGGCAAGACTCTCCTCGATGCTATTGATGCCATTGAACCCCCCGTCCGACCTTCCGACAAACCCCTCCGTCTCCCCCTCCAGGATGTCTACAAAATTGGCGGTATCGGAACTGTGCCCGTCGGTCGTGTTGAGACTGGTATCATCAAGGCCGGAATGGTCGTCACTTTCGCTCCCTCCAACGTCACCACTGAAGTCAAGTCCGTCGAAATGCATCACGAACAGCTCGAGCAGGGTAATCCTGGTGACAATGTCGGTTTCAATGTCAAGAACGTGTCAGTAAAGGATATTCGTCGTGGCAACGTGGCCTCCGACTCCAAGAACGATCCGGCCAAGGAAGCAGCCTCTTTCAAYGCACAGGTCATTGTCCTCAACCACCCTGGTCAAATTGGTGCTGGTTACGCCCCAGTTCTCGACTGTCACACTGCCCACATCGCCTGCAAGTTCGCCGAGCTCATCGAGAAGATCGATCGTCGTACGGGTAAATCCATCGAAAACTCGCCTAAATTTGTCAAGTCGGGTGACGCCTGCATCGTCAAACTCGTTCCCAGCAAGCCTATG

>AWW446_Laccaria_montana_CHINA_JX504097_JX504181_KU686054_KU686157

CCTGA-TGTGACTGTTAGCTGGC-TTTTCGAAGCATGTGCTCGT-CCGTCATCTTTAATCTCT--CCA-CCTGTGCACATTTTGTAGTC-TT-GGATACCTCTCGAGGCAA-CTCGGATT----TTAG-GATCGCCGTGC---TGT---AAAAGTCAGCTTTCCTCTCATTTCC-AAGACTATGTTTT----T--A-TATACA-CCAAAGTATGTTTAAAGAATGTCATC-AATAGGAAC---TTGTTTCCTA--T-AAAATTATACAACTTTCAGCAACGGATCTCTTGGCTCTCGCATCGATGAAGAACGCAGCGAAATGCGATAAGTAATGTGAATTGCAGAATTCAGTGAATCATCGAATCTTTGAACGCACCTTGCGCTCCTTGGTATTCCGAGGAGCATGCCTGTTTGAGTGTCATTAAA-TTCTCAA-CCTTCCAGC--------T-TTTATTAGCTTGG--TTAGGCTTGGA-TGT-GGGGG--TTGTGGGCTTC-AT-----T---AATGAGGTCAGCTCTCCTTAAATGCATTAGCGGAACTTTTGTGGACCGTCTA-TTGGTGTGAT-AATTATCTACGCCGTGGAT-GTGAAGCAG-CTTTAT---GAAGTT-CAGCTTCT-AATCGTCC---ATTGACTTGGACAA----TTTTTGACAA-TTTGAAAGCTCAAATTTAAAATCTGGCAGT-CTTTGGCTGT-CCGAGTTGTAATCTAGAGAAGTATTATCCGCGCTGGACCGTGTACAAGTCCCCTGGAATGGGGCGTCATAGAGGGTGAGAATCCCGTCTTTGACACGGACTGCCAGGGC-TTTGTGATGTGCTCTCAAAGAGTCGAGTTGTTTGGGAATGCAGCTCAAAATGGGTGGTAAATTCCATCTAAAGCTAAATATTGGCGAGAGACCGATAGCGAACAAGTACCGTGAGGGAAAGATGAAAAGAACTTTGGAAAGAGAGTTAAACAGTACGTGAAATTGCTGAAAGGGAAACGCTTGAAGTCAGTCGCGTTGGCCAGGGATCAA-CCTTGC----TTTTTT---GCTTGGCTTACTTTCTGGTCGATGGG-TCAGCATCAATTTTGACTGGTGG-AAAAAGTTCAAGGGAATGTGGCAT-CTTCGGATGTGTTATAGCCTTTGTTCACATACATCAGTTGGGATTGAGGAACTCAGCACGCCGCAAGGCCGGG---TTTTTAACCACGTACGTGCTTAGGATGCTGGCATAATGGCTTTAATCGACCCGTCTTGAAACACGGACCAAGGAGTCTAACATGCCTGCGAGTGTTTGGGTGAAAAACCCGAGCGCGTAATGAAAGTG-AAAGTTGAGATCCCTGTCGTGGGGAGCATTGACGCCCGGATCTGACCTTTTGTGACGATTCTGCGGTAGAGCATGTTTTCCGCATGCTTTTCCGAAAGTTGACCAAGGACGTTTATCGTTATCTACAAAAGgttgctgaccttcgctcaagtgcc-----atgagatgtgctgaccccctct---tcttcagTGCGTCGARACCCACAAGGAGTTCAACCTTGCCCTTGCAGTTAAGCATCAAACCATCACAAACGGCCTCAAATACTCTCTGGCGACAGGTAACTGGGGAGATCAGAAAAAGTCAATGTCTTCCAAGGCAGGAGTATCTCAGGTCTTGAACAGATACACATATGCATCCACTCTGTCRCATCTTCGTCGGTGTAACACTCCTCTGGGGCGCGAAGGCAAGATCGCCAAACCTCGTCAGTTGCACAACACTCATTGGGGTATGGTGTGTCCTGCGGAAACTCCTGAAGGTCAAGCCTGTGGTCTTGTCAAGAAYCTCGCCCTTATGGCGTGCATATCAGTCGGGTCTTACTCTGCGCCCGTTATCGAGTTTTTGGAGGAGTGGGGATTGGAGTCGTTGGAAGAGAAYGCGCACTCATCAACACCTTGCACMAAAGTTTTCGTGAACGGTGTCTGGATGGGTGTGCATCGCGATCCTGCCAATTTGGTGAAGACGATAAAGAAATTGAGAAGGAAAGACGACATTAGCCCAGAAGTGTCAGTCGTGCGAGACATCAGGGAGAAGGAATTGAGGTTGTACACCGATGCTGGACGTGTTTGTCGACCACTCTTCATCGTCGATAACCAACAGCTGGCACTTCAGAAGAAGCAYGTCAAATGGCTCAGTAACGGCCTCAACGATGATGGCGACGAGTACAAATGGGAGCATCTGGTAAAGGGTGGCATCATTGAGTTACTGGATGCTGAGGAAGAGGAAACAGTGATGATATCCATGACCCCTGAAGATCTTGAAAATTCTCGCYTACAGCAAAGTGGTGTTGATCCTCACGCGAACGACGGCGAGTTTGATCCAGCAGCTCGATTGAAGGCTGGCACTCACGCACACACATGGACACATTGCGAAATTCATCCTAGCATGATTCTTGGCATYTGTGCCAGCATTATTCCTTTCCCTGATCATTTGCGCCATTCTCATCATTGCTGCTGGTACTGGTGAGTTCGAGGCTGGTATCTCCAAAGATGGCCAGACCCGCGAGCACGCTCTCCTCGCTTTCACCCTCGGTGTGCGGCAACTCATCGTTGCCGTCAACAAGATGGACACCACTAAGGTAAGAAACGTTTCAC-TTATAGTTAGGCTTCTTGCACATTCTCATCATCTCA-TGTAGTGGAGCGAGGACCGTTTCAACGAAATCATCAAGGAGACGTCAACCTTCATCAAGAAGGTTGGTTACAACCCCAAGGCCGTTGCCTTTGTCCCTATTTCCGGCTGGCACGGTGACAACATGTTGGAGGAGTCCCCTAAGTAAGCATTC--TCGTATCGTCATGATCGCCCCGTCGCTCATGTTGCACTCAAGCATGTCTTGGTACAAGGGCTGGACCAAGGAGACCAAGGCTGGTGTCGTCAAGGGCAAGACCCTCCTCGATGCTATTGATGCCATTGAGCCCCCCGTCCGACCTTCCGACAAACCCCTCCGTCTCCCTCTTCAGGATGTCTACAAAATTGGCGGTATCGGAACTGTGCCCGTCGGTCGTGTTGAGACTGGCATCATCAAGGCCGGAATGGTCGTCAATTTCGCTCCCTCCAACGTTACCACTGAAGTCAAGTCCGTCGAAATGCATCACGAACAGCTCGAGCAGGGTAACCCTGGTGACAATGTCGGTTTCAACGTCAAAAACGTGTCAGTGAAGGATATCCGTCGTGGCAACGTGGCCTCCGACTCCAAGAACGATCCCGCCAAGGAAGCGGCCTCTTTCAACGCACAGGTCATCGTCCTCAACCACCCTGGTCAAATTGGTGCTGGTTACGCCCCCGTTCTCGATTGTCACACCGCCCACATCGCCTGCAAGTTCGCCGAGCTCATCGAGAAGATCGATCGTCGTACGGGTAAATCCATTGAAAACTCGCCTAAATTCGTCAAGTCGGGTGACGCCTGCATCGTCAAGCTCGTTCCCAGCAAGCCTATG

>AWW463_Laccaria_himalayensis_CHINA_JX504098_JX504182_KU685913_

CCTGA-TGTGGCTGTTAGCTGGCTTTTTTGAAGCATGTGCTCGT-CCGTCATCTTTAATCTCT--CCA-CCTGTGCACATTTTGTAGTC-TT-GGATACCTCTCGAGGAAA-CTCGGATT----TTAG-AATCGCCGTGC---TGT---ACAAGTCGGCTTTCCTTTCATTTCC-AAGACTATGTTTT----T--A-TATACA-CCAATGTATGTTTAAAGAATGTCATC-AATAGGAAC---TTGTTTCCTA--T-AAAATTATACAACTTTCAGCAACGGATCTCTTGGCTCTCGCATCGATGAAGAACGCAGCGAAATGCGATAAGTAATGTGAATTGCAGAATTCAGTGAATCATCGAATCTTTGAACGCACCTTGCGCTCCTTGGTATTCCGAGGAGCATGCCTGTTTGAGTGTCATTAAA-TTCTCAA-CCTTCCAGC--------T-TTTATTAGCTTGG--TTAGGCTTGGA-TGT-GGGGG--TTGTGGGCTTC-AT-----T---ATTGAGGTCGACTCTCCTTAAATGCATTAGCGGAACTTTTGTGGACCGTCTA-TTGGTGTGAT-AATTATCTACGCTGTGGAT-GTGAAGCAG--------------TT-CAGCTTCT-AACCGTCC---ATTGACTTGGACAA-----TTTTGACAA-TTTGAAAGCTCAAATTTAAAATCTGGCAGT-CTTTGGCTGT-CCGAGTTGTAATCTAGAGAAGTATTATCCGCGCTGGACCGTGTACAAGTCTCCTGGAATGGAGCGTCATAGAGGGTGAGAATCCCGTCTTTGACACGGACTGCCAGGGC-TTTGTGATGTGCTCTCAAAGAGTCGAGTTGTTTGGGAATGCAGCTCAAAATGGGTGGTAAATTCCATCTAAAGCTAAATATTGGCGAGAGACCGATAGCGAACAAGTACCGTGAGGGAAAGATGAAAAGAACTTTGGAAAGAGAGTTAAACAGTACGTGAAATTGCTGAAAGGGAAACGCTTGAAGTCAGTCACGTTGGCCAGGGATCAA-CCTTGC----TTTTTT---GCTTGGCTTACTTTCTGGTTGATGGG-TCAGCATCAATTTTGACTGGTGG-AAAAAGTTCAAGGGAATGTGGCAT-CTTCGGATGTGTTATAGCCTTTGTTCGCATACATCAATTGGGATTGAGGAACTCAGCACGCCGCAAGGCCGGG---TTTTTAACCACGTACGTGCTTAGGATGCTGGCATAATGGCTTTAATCGACCCGTCTTGAAACACGGACCAAGGAGTCTAACATGCCTGCGAGTGTTTGGGTGAAAAACCCGAGCGCGTAATGAAAGTG-AAAGTTGAGATCCCTATCGTGGGGAGCATTGACGCCCGGATCTGACCTTTTGTGACGATTCTGCGGTAGAGCATGTTTTCCGCATGCTTTTCCGAAAGTTGACCAAGGACGTTTATCGTTACCTACAAAAGgttgctgaccttcgttcaagtgcc-----atgagatgtgctca-cccctct---ttttcagTGTGTCGAGGCCCACAAGGAGTTCAACCTTGCCCTTGCAGTTAAGCATCAAACCATCACGAACGGCCTCAAATACTCTCTGGCGACAGGCAACTGGGGAGATCAGAAAAAGTCAATGTCTTCCAAGGCAGGAGTATCTCAGGTCTTGAACAGATACACATATGCGTCCACTCTGTCACATCTTCGTCGGTGTAACACTCCTCTAGGGCGCGAAGGCAAGATCGCCAAACCTCGTCAGTTGCACAACACTCATTGGGGTATGGTGTGTCCTGCGGAAACTCCTGAAGGTCAAGCCTGTGGTCTTGTCAAGAACCTCGCCCTTATGGCGTGCATATCAGTCGGGTCTTACTCCGCGCCCGTTATCGAGTTTTTGGAGGAGTGGGGATTGGAGTCTTTGGAAGAGAACGCACACTCATCGACACCYTGCACAAAAGTTTTCGTGAACGGTGTCTGGATGGGTGTGCATCGCGATCCTGCCAATTTGGTGAAGACGATAAAGAAATTGAGAAGGAAAGACGACATCAGCCCAGAAGTGTCAGTTGTGCGAGACATCAGGGAGAAGGAATTGAGGTTGTACACCGATGCTGGACGCGTTTGTCGACCACTCTTCATCGTCGAGAACCAACAGCTGGCACTTCAGAAGAAGCACGTCAAATGGCTCAGTAACGGCCTCAACGATGATGGCGACGAGTACAAATGGGAACATCTGGTAAAGGGTGGAATCATTGAGTTACTGGATGCTGAGGAAGAGGAAACAGTGATGATATCCATGACCCCTGAAGATCTTGAAAATTCTCGCCTACAGCAAAGTGGTGTTGATCCTCACGCGAACGACGGCGAGTTTGATCCAGCAGCTCGATTGAAGGCTGGCACTCACGCACACACATGGACACATTGCGAAATTCATCCTAGCATGATTCTTGGCATTTGTGCCAGCATCATTCCTTTCCCTGATCAT------------------------------------------------------------------------------------------------------------------------------------------------------------------------------------------------------------------------------------------------------------------------------------------------------------------------------------------------------------------------------------------------------------------------------------------------------------------------------------------------------------------------------------------------------------------------------------------------------------------------------------------------------------------------------------------------------------------------------------------------------------------------------------------------------------------------------------------------------------------------------------------------------------------------------------------------------------------------------------------------------------------------------------------------

>AWW465_Laccaria_bullipellis TYPE_CHINA_JX504100_JX504184_KU685914_

CCTGA-TGTGGCTGTTAGCTGGCTTTTTCGAAGCATGTGCTCGT-CCGTCATCTTTAATCTCT--CCA-CCTGTGCACATTTTGTAGTC-TT-GGATACCTCTCGAGGAAA-CTCGGATT----TTAG-AATCGCCGTGC---TGT---ACAAGTCGGCTTTCCTTTCATTTCC-AAGACTATGTTTT----T--A-TATACA-CCAAAGTATGTTTAAAGAATGTCATC-AATAGGAAC---TTGTTTCCTA--T-AAAATTATACAACTTTCAGCAACGGATCTCTTGGCTCTCGCATCGATGAAGAACGCAGCGAAATGCGATAAGTAATGTGAATTGCAGAATTCAGTGAATCATCGAATCTTTGAACGCACCTTGTGCTCCTTGGTATTCCGAGGAGCATGCCTGTTTGAGTGTCATTAAA-TTCTCAA-CCTTCCAGC--------T-TTTATTAGCTTGG--TTAGGCTTGGA-TGT-GGGGG--TTGTGGGCTTC-AT-----T---AATGAGGTCGACTCTCCTTAAATGCATTAGCGGAACTTTTGTGGACCGTCTA-TTGGTGTGAT-AATTATCTACGCTGTGGAT-GTAAAGCAG--------------TT-CAGCTTCT-AACCGTCC---ATTGACTTGGACAA-----TTTTGACAA-TTTGAAAGCTCAAATTTAAAATCTGGCAGT-CTTTGGCTGT-CCGAGTTGTAATCTAGAGAAGTATTATCCGCGCTGGACCGTGTACAAGTCTCCTGGAATGGAGCGTCATAGAGGGTGAGAATCCCGTCTTTGACACGGACTGCCAGGGC-TTTGTGATGTGCTCTCAAAGAGTCGAGTTGTTTGGGAATGCAGCTCAAAATGGGTGGTAAATTCCATCTAAAGCTAAATATTGGCGAGAGACCGATAGCGAACAAGTACCGTGAGGGAAAGATGAAAAGAACTTTGGAAAGAGAGTTAAACAGTACGTGAAATTGCTGAAAGGGAAACGCTTGAAGTCAGTCACGTTGGCCAGGGATCAA-CCTTGC----TTTTTT---GCTTGGCTTACTTTCTGGTTGATGGG-TCAGCATCAATTTTGACTGGTGGAAAAAAGTTCAAGGGAATGTGGCAT-CTTCGGATGTGTTATAGCCTTTGTTCGCATACATCAATTGGGATTGAGGAACTCAGCACGCCGCAAGGCCGGG---TTTTTAACCACGTACGTGCTTAGGATGCTGGCATAATGGCTTTAATCGACCCGTCTTGAAACACGGACCAAGGAGTCTAACATGCCTGCGAGTGTTTGGGTGAAAAACCCGAGCGCGTAATGAAAGTG-AAAGTTGAGATCCCTATCGTGGGGAGCATTGACGCCCGGATCTGACCTTTTGTGACGATTCTGCGGTAGAGCATGTTTTCCGCATGCTTTTCCGAAAGTTGACCAAGGACGTTTATCGTTATCTACAAAAGgttgctgaccttcgttcaagtgcc-----atgagatgtgctga-cccctct---ttttcagTGTGTCGAGGCCCACAAGGAGTTCAACCTTGCCCTTGCAGTTAAGCATCAAACCATCACGAACGGCCTCAAATACTCTCTGGCGACAGGCAACTGGGGAGATCAGAAAAAGTCAATGTCTTCCAAGGCAGGAGTATCTCAGGTCTTGAACAGATACACATATGCGTCCACTCTGTCACATCTTCGTCGGTGTAACACTCCTCTAGGGCGCGAAGGCAAGATCGCCAAACCTCGTCAGTTGCACAACACTCATTGGGGTATGGTGTGTCCTGCGGAAACTCCTGAAGGTCAAGCCTGTGGTCTTGTCAAGAACCTCGCCCTTATGGCGTGCATATCAGTCGGGTCTTACTCCGCGCCCGTTATCGAGTTTTTGGAGGAGTGGGGATTGGAGTCTTTGGAAGAAAACGCACACTCATCGACACCTTGCACAAAAGTTTTCGTGAACGGTGTCTGGATGGGTGTGCATCGCGATCCTGCCAATTTGGTGAAGACGATAAAGAAATTGAGAAGGAAAGACGACATCAGCCCAGAAGTGTCAGTTGTGCGAGACATCAGGGAGAAGGAATTGAGGTTGTACACCGATGCTGGACGCGTTTGTCGACCACTCTTCATCGTCGAGAACCAACAGCTGGCACTTCAGAAGAAGCACGTCAAATGGCTCAGTAACGGCCTCAACGATGATGGCGACGAGTACAAATGGGAACATCTGGTAAAGGGTGGAATCATTGAGTTACTGGATGCTGARGAAGAGGAAACAGTGATGATATCCATGACCCCTGAAGATCTTGAAAATTCTCGCCTACAGCAAAGTGGTGTTGATCCTCACGCGAACGACGGCGAGTTTGATCCAGCAGCTCGATTGAAGGCTGGCACTCACGCACACACATGGACACATTGCGAAATTCATCCTAGCATGATTCTTGGCATTTGTGCCAGCATCATTCCTTTCCCTGATCAT------------------------------------------------------------------------------------------------------------------------------------------------------------------------------------------------------------------------------------------------------------------------------------------------------------------------------------------------------------------------------------------------------------------------------------------------------------------------------------------------------------------------------------------------------------------------------------------------------------------------------------------------------------------------------------------------------------------------------------------------------------------------------------------------------------------------------------------------------------------------------------------------------------------------------------------------------------------------------------------------------------------------------------------------

>AWW484_Laccaria_himalayensis TYPE_CHINA_JX504101_JX504185_KU685915_

CCTGA-TGTGGCTGTTAGCTGGCTTTTTTGAAGCATGTGCTCGT-CCGTCATCTTTAATCTCT--CCA-CCTGTGCACATTTTGTAGTC-TT-GGATACCTCTCGAGGAAA-CTCGGATT----TTAG-AATCGCCGTGC---TGT---ACAAGTCGGCTTTCCTTTCATTTCC-AAGACTATGTTTT----T--A-TATACA-CCAATGTATGTTTAAAGAATGTCATC-AATAGGAAC---TTGTTTCCTA--T-AAAATTATACAACTTTCAGCAACGGATCTCTTGGCTCTCGCATCGATGAAGAACGCAGCGAAATGCGATAAGTAATGTGAATTGCAGAATTCAGTGAATCATCGAATCTTTGAACGCACCTTGCGCTCCTTGGTATTCCGAGGAGCATGCCTGTTTGAGTGTCATTAAA-TTCTCAA-CCTTCCAGC--------T-TTTATTAGCTTGG--TTAGGCTTGGA-TGT-GGGGG--TTGTGGGCTTC-AT-----T---ATTGAGGTCGACTCTCCTTAAATGCATTAGCGGAACTTTTGTGGACCGTCTA-TTGGTGTGAT-AATTATCTACGCTGTGGAT-GTGAAGCAG--------------TT-CAGCTTCT-AACCGTCC---ATTGACTTGGACAA-----TTTTGACAA-TTTGAAAGCTCAAATTTAAAATCTGGCAGT-AACGGGCTGT-CCGAGTTGTAATCTAGAGAAGTATTATCCGCGCTGGACCGTGTACAAGTCTCCTGGAATGGAGCGTCATAGAGGGTGAGAATCCCGTCTTTGACACGGACTGCCAGGGC-TTTGTGATGTGCTCTCAAAGAGTCGAGTTGTTTGGGAATGCAGCTCAAAATGGGTGGTAAATTCCATCTAAAGCTAAATATTGGCGAGAGACCGATAGCGAACAAGTACCGTGAGGGAAAGATGAAAAGAACTTTGGAAAGAGAGTTAAACAGTACGTGAAATTGCTGAAAGGGAAACGCTTGAAGTCAGTCACGTTGGCCAGGGATCAA-CCTTGC----TTTTTT---GCTTGGCTTACTTTCTGGTTGATGGG-TCAGCATCAATTTTGACTGGTGG-AAAAAGTTCAAGGGAATGTGGCAT-CTTCGGATGTGTTATAGCCTTTGTTCGCATACATCAATTGGGATTGAGGAACTCAGCACGCCGCAAGGCCGGG---TTTTTAACCACGTACGTGCTTAGGATGCTGGCATAATGGCTTTAATCGACCCGTCTTGAAACACGGACCAAGGAGTCTAACATGCCTGCGAGTGTTTGGGTGAAAAACCCGAGCGCGTAATGAAAGTG-AAAGTTGAGATCCCTATCGTGGGGAGCATTGACGCCCGGATCTGACCTTTTGTGACGATTCTGCGGTAGAGCATGTTTTCCGCATGCTTTTCCGAAAGTTGACCAAGGACGTTTATCGTTATCTACAAAAGgttgctgaccttcgttcaagtgcc-----atgagatgtgctca-cccctct---ttttcagTGTGTCGAGGCCCACAAGGAGTTCAACCTTGCCCTTGCAGTTAAGCATCAAACCATCACGAACGGCCTCAAATACTCTCTGGCGACAGGCAACTGGGGAGATCAGAAAAAGTCAATGTCTTCCAAGGCAGGAGTATCTCAGGTCTTGAACAGATACACATATGCGTCCACTCTGTCACATCTTCGTCGGTGTAACACTCCTCTAGGGCGCGAAGGCAAGATCGCCAAACCTCGTCAGTTGCACAACACTCATTGGGGTATGGTGTGTCCTGCGGAAACTCCTGAAGGTCAAGCCTGTGGTCTTGTCAAGAACCTCGCCCTTATGGCGTGCATATCAGTCGGGTCTTACTCCGCGCCCGTTATCGAGTTTTTGGAGGAGTGGGGATTGGAGTCTTTGGAAGAGAACGCACACTCATCGACACCTTGCACAAAAGTTTTCGTGAACGGTGTCTGGATGGGTGTGCATCGCGATCCTGCCAATTTGGTGAAGACGATAAAGAAATTGAGAAGGAAAGACGACATCAGCCCAGAAGTGTCAGTTGTGCGAGACATCAGGGAGAAGGAATTGAGGTTGTACACCGATGCTGGACGCGTTTGTCGACCACTCTTCATCGTCGAGAACCAACAGCTGGCACTTCAGAAGAAGCACGTCAAATGGCTCAGTAACGGCCTCAACGATGATGGCGACGAGTACAAATGGGAACATCTGGTAAAGGGTGGAATCATTGAGTTACTGGATGCAGAGGAAGAGGAAACAGTGATGATATCCATGACCCCTGAAGATCTTGAAAATTCKCGCCTACAGCAAAGTGGTGTTGATCCTCACGCGAACGACGGCGAGTTTGATCCAGCAGCTCGATTGAAGGCTGGCACTCACGCACACACATGGACACATTGCGAAATTCATCCTAGCATGATTCTTGGCATTTGTGCCAGCATCATTCCTTTCCCTGATCAT------------------------------------------------------------------------------------------------------------------------------------------------------------------------------------------------------------------------------------------------------------------------------------------------------------------------------------------------------------------------------------------------------------------------------------------------------------------------------------------------------------------------------------------------------------------------------------------------------------------------------------------------------------------------------------------------------------------------------------------------------------------------------------------------------------------------------------------------------------------------------------------------------------------------------------------------------------------------------------------------------------------------------------------------

>AWW485_Laccaria_acanthospora TYPE_CHINA_JX504102_JX504186_KU685916_KU686073

TCTGA-TGTGGCTGTTAGCTGGC-TTTTTGAAGCATGTGCTCGC-CTGTCATCTTT-ATCTCT--CCA-CCTGTGCACCTTTTGTAGTC-TT-GGATACCTCTCGAGGCAA-CTCGGATT----TTGG-GATCGC------------------GTTAGCTTTCCTTGCATTTCC-AAGACTATGTTTT----T--A-TATACA-CCAAAGAATGTTTAAAGAATGTCATC-AATAGGAAC---TTGTTTCCTA--TAAAAACTATACAACTTTCAGCAACGGATCTCTTGGCTCTCGCATCGATGAAGAACGCAGCGAAATGCGATAAGTAATGTGAATTGCAGAATTCAGTGAATCATCGAATCTTTGAACGCACCTTGCGCTCCTTGGTATTCCGAGGAGCATGCCTGTTTGAGTGTCATTAAA-TTCTCAA-CCTTCCAGC--------T-TTTATTAGCTTGG--TTAGGCTTGGA-TGT-GGGGG--TTGCGGGCTTC-AC-----T---TATGAGGTCGGCTCTCCTTAAATGCATTAGCGGAACTTTTGTGGACCGTCTA-TTGGTGTGAT-AATTATCTACGCTGTGGAT-GTGAAGCAG-CTTTAT---GAAGTT-CAGCTTCT-AACAGTCC---ATTGACTTGGACAA----TTTTTGACAA-TTTGAAAGCTCAAATTTAAAATCTGGCAGT-CTTTGGCTGT-CCGAGTTGTAATCTAGAGAAGCATTATCCGCGCTGGACCGTGTACAAGTCTCCTGGAATGGAGCGTCATAGAGGGTGAGAATCCCGTCTTTGACACGGACTGCCAGGGC-TTTGTGATATGCTCTCAAAGAGTCGAGTTGTTTGGGAATGCAGCTCAAAATGGGTGGTAAATTCCATCTAAAGCTAAATATTGGCGAGAGACCGATAGCGAACAAGTACCGTGAGGGAAAGATGAAAAGAACTTTGGAAAGAGAGTTAAACAGTACGTGAAATTGCTGAAAGGGAAACGCTTGAAGTCAGTCGCGTTGGCCAGGGATCAA-CCTTGC----ATTTTT---GCTTGGCTTATTTCCTGTTTGACGGG-TCAGCATCAATTTTGACTGGTGG-AAAAAGTTCAAGGGAATGTGGCAT-CTTTGGATGTGTTATAGCCTTTGTTCGCATACATCGGTTGGGATTGAGGAACTCAGCACGCCGAAAGGCCGGG---TTTTTAACCACGTACGTGCTTAGGATGCTGGCATAATGGCTTTAATCGACCCGTCTTGAAACACGGACCAAGGAGTCTAACATGCCTGCGAGTGTTTGGGTGGAAAACTCGAGCGCGTAATGAAAGTG-AAAGTTGAGATCCCTGTCATGGGGAGCATCGACGCCCGGATCTGACCTTTTGTGACGATTCCGCGGTAGAGCATGTTTTTCGCATGCTTTTCCGAAAGTTGACCAAGGACGTTTATCGTTATCTTCAAAAGgttgctgagcttcgctcaagttcc-----atgagatgtgctga-cccctct---tcttcagTGCGTCGAGACCCACAAGGAGTTCAACCTTGCCCTTGCAGTTAAGCATCAAACCATCACAAACGGCCTCAAATACTCTCTGGCTACAGGTAACTGGGGGGATCAGAAGAAGTCAATGTCTTCCAAGGCAGGAGTATCCCAGGTCTTGAACAGATACACATATGCCTCCACTCTGTCACATCTTCGTCGGTGTAACACTCCTCTAGGGCGCGAAGGCAAGATCGCCAAACCTCGTCAGTTGCACAACACTCATTGGGGTATGGTGTGTCCTGCGGAAACTCCTGAAGGTCAAGCCTGTGGTCTTGTCAAGAACCTCGCCCTTATGGCGTGCATATCAGTCGGGTCTTACTCCGCGCCCGTTATYGAGTTTTTGGAGGAGTGGGGATTAGAGTCATTGGAAGAGAACGCGCACTCATCGACACCCTGCACAAAAGTTTTCGTGAACGGCGTCTGGATGGGTGTACATCGCGATCCCGCCAATTTGGTGAAGACGATAAAGAAGTTGAGAAGGAAAGACGACATCAGCCCAGAGGTGTCAGTTGTGCGAGACATCAGGGAGAAGGAATTGAGGTTGTACACCGATGCTGGACGTGTTTGTCGACCCCTCTTCATCGTCGAGAACCAGCAGYTGGCACTTCAGAAGAAGCACGTCAAATGGCTCAGTAACGGCCTCAACGACGATGGCGACGAGTACAAATGGGAACATCTGGTAAAGGGTGGCATCATTGAGTTATTGGACGCTGAGGAAGAGGAAACAGTTATGATATCCATGACCCCTGAAGATCTTGAAAATTCTCGCCTACAGCAAAGTGGTGTTGATCCTCACGCGAACGACGGCGAGTTTGATCCAGCAGCTCGGTTGAAGGCCGGCACTCACGCACACACATGGACACATTGCGAAATTCATCCTAGCATGATACTTGGCATTTGTGCCAGCATTATCCCTTTCCCTGATCATCTGCGCCGTTCTCATCATTGCTGGTGGTACTGGTGAGTTCGAGGCTGGAWTCTCCAAGGATGGCCAGACCCGCGAGCACGCTCTCCTCGCTTTCACCCTCGGTGTGCGGCAACTCATCGTTGCCGTCAACAAGATGGACACCACTAAGGTAACAAACGTTTCAT-TCATAGTTAGGCTTTTTGCACATTCTCATCATCCCA-TGTAGTGGAGCGAGGACCGTTTCAACGAAATCATCAAGGAGACGTCAACCTTCATCAAGAAGGTTGGTTACAACCCAAAGGCCGTTGCCTTTGTCCCTATTTCCGGCTGGCACGGTGACAACATGTTGGAGGAGTCCCCTAAGTAAGCATTC--TCGTGTCGTCATGATCGCCCCGTCGCTCATTTTCCACTC-AGCATGCCTTGGTATAAGGGCTGGACCAGGGAGACCAAGGCTGGCGTCGTCAAGGGCAAGACCCTCCTCGATGCTATTGATGCCATTGAGCCCCCCGTCCGTCCTTCCGACAAACCCCTCCGTCTCCCCCTCCAGGATGTCTACAAAATTGGCGGTATCGGAACTGTGCCCGTCGGTCGTGTTGAGACTGGTATCATCAAGGCCGGAATGGTCGTCACTTTCGCTCCCTCTAACGTCACCACTGAAGTCAAGTCCGTCGAAATGCATCACGAACAGCTCGAGCAGGGTAACCCTGGTGACAATGTCGGTTTCAACGTCAAGAACGTGTCAGTGAAGGATATTCGTCGTGGCAACGTGGCCTCCGACTCCAAGAACGATCCCGCCAAGGAAGCGGCCTCTTTCAACGCACAGGTCATCGTCCTCAACCACCCTGGCCAAATTGGTGCTGGTTACGCCCCGGTTCTCGACTGTCACACCGCACATATCGCCTGCAAGTTCGCCGAGCTCATCGAGAAGATCGATCGTCGTACGGGTAAATCCATTGAAAACTCGCCCAAATTCGTTAAGTCGGGTGATGCCTGCATCGTCAAGCTCGTTCCTAGCAAGCCTATG

>AWW544_Laccaria_aff bicolor_USA_IL_KM067820__RPB2_

TCTGA-TGTGACTGTTAGCTGGC-TTTTCGAAGCATGTGCTCGT-CCATCATCTTT-ATCTCT--CCA-CCTGTGCACATTTTGTAGTC-TT-GGATACCTCTCGAGGAAA-CTTGGATT----TTAG-GATCGCCGTGC---TGT---ACAAGTCGGCTTTTCTTTCATTTCC-AAGACTATGTTTT----T--A-TATACA-CCAAAGTATGTTTATAGAATGTYATC-AATGGGAAC---TTGTTTCCTA--T-AAAATTATACAACTTTCAGCAACGGATCTCTTGGCTCTCGCATCGATGAAGAACGCAGCGAAATGCGATAAGTAATGTGAATTGCAGAATTCAGTGAATCATCGAATCTTTGAACGCACCTTGCGCTCCTTGGTATTCYGAGGAGCATGCCTGTTTGAGTGTCATTAAA-TTCTCAA-CCTTCCAAC--------T-TTTATTAGCTTGG--TTAGGCTTGGA-TGT-GGGGG--TTGCAGGCTTC-AT-----C---ACTGAGGTCGGCTCTCCTTAAATGCATTAGYGGAACTTTTGTGGACCGTCTA-TTGGTGTGAT-AATTATCTACGCCGTGGAT-GTGAAGCAG-CTTTAT---GAAGTT-CTGCTTCT-AACCGTCC---ATTGACTTGGACAA-----TTTTGACAA-TTTGA--------------------------------------------------------------------------------------------------------------------------------------------------------------------------------------------------------------------------------------------------------------------------------------------------------------------------------------------------------------------------------------------------------------------------------------------------------------------------------------------------------------------------------------------------------------------------------------------------------------------------------------------------------------------------------------------------------------------------------------------------------------TTT-CGCATGCTTTTCCGAAAGTTGACCAAGGACGTTTATCGTTATCTACAAAAAgttgctgactttcactcaagtgcc-----atgagacgtattga-tctctct---tcttcagTGCGTCGAGACCCACAAGGAGTTCAACCTTGCCCTTGCAGTTAAGCATCAAACCATCACAAACGGCCTCAAATACTCTCTGGCGACAGGTAACTGGGGGGATCAGAAAAAGTCAATGTCTTCCAAGGCAGGAGTATCTCAGGTCTTGAACAGATACACATATGCATCCACTCTGTCACATCTTCGTCGGTGTAACACTCCTCTGGGGCGCGAAGGCAAGATCGCCAAACCTCGTCAGTTGCACAACACCCATTGGGGTATGGTGTGCCCTGCGGAAACTCCTGAAGGTCAAGCCTGTGGTCTTGTCAAGAACCTCGCCCTTATGGCGTGCATATCAGTCGGGTCTTACTCCGCCCCCGTTATCGAGTTTTTGGAGGAATGGGGATTGGAATCATTGGAAGAGAACGCRCACTCATCAACTCCCTGCACAAAAGTTTTCGTGAACGGTGTATGGATGGGTGTGCATCGCGATCCTGCCAACTTGGTGAAGACGATAAAGAAATTGAGAAGGAAAGACGACATTAGCCCTGAAGTGTCAGTTGTGCGAGACATCCGAGAGAAGGAATTGAGGTTGTACACCGATGCTGGACGTGTTTGTCGACCACTTTTCATCGTCGAGAACCAACAGTTGGCACTTCAGAAGAAGCACGTCAAATGGCTTAGTAACGGCCTCAACGATGATGGCGACGAATACAAATGGGAACATTTGGTAAAGGGTGGCATCATTGAGTTACTGGATGCTGAGGAAGAGGAAACGGTGATGATATCCATGACCCCTGAAGATCTTGAAAATTCTCGCCTACAGCAAAGTGGCGTTGATCCTCACGCGAACGACGGCGAGTTTGATCCAGCAGCTCGATTAAAGGCTGGCACTCACGCACACACATGGACACATTGCGAAATTCACCCTAGCATGATTCTTGGCATTTGTGCCAGCATCATTCCTTTCCCCGATCAT------------------------------------------------------------------------------------------------------------------------------------------------------------------------------------------------------------------------------------------------------------------------------------------------------------------------------------------------------------------------------------------------------------------------------------------------------------------------------------------------------------------------------------------------------------------------------------------------------------------------------------------------------------------------------------------------------------------------------------------------------------------------------------------------------------------------------------------------------------------------------------------------------------------------------------------------------------------------------------------------------------------------------------------------

>AWW545_Laccaria_aff ohiensis_USA_IL_JX504106_JX504190_KU685917_

CCTGA-TGTGGCTGTTAGCTGGC-TTTTCGAAGCATGTGCTCGT-CCGTCATCTTTAATCTCT--CCA-CCTGTGCACATTTTGTAGTCTTT-GGATACCTCTCGAGGCAA-CTCGGATA----TTAG-GATTGCCGTGC---TGT---ACAAGTCGGCTTTTCTTTCATTTCC-AAGACTATGTTTT----T--A-TATACC-CCAAAGTATGTTTAAAGAATGTCATC-AATGGGAAC---TTGTTTCCTA--T-AAAATTATACAACTTTCAGCAACGGATCTCTTGGCTCTCGCATCGATGAAGAACGCAGCGAAATGCGATAAGTAATGTGAATTGCAGAATTCAGTGAATCATCGAATCTTTGAACGCACCTTGCGCTCCTTGGTATTCCGAGGAGCATGCCTGTTTGAGTGTCATTAAA-TTCTCAA-CCTTCCAGC--------T-TTTATTAGCTTGG--TTAGGCTTGGA-TGT-GGGGG--TTGCGGGCTTC-AT-----C---AATGAGGTCGGCTCTCCTTAAATGCATTAGCGGAACTTTTGTGGACCGTCTA-TTGGTGTGAT-AATTATCTACGCCGTGGAT-GTAAAGCAG-CTTTAT---AAAGTT-CAGCTTCT-AACCGTCC---ATTGACTTGGACAA-----TTTTGACAA-TTTGAAAGCTCAAATTTAAAATCTGGCAGT-CTTTGGCTGT-CCGAGTTGTAATCTAGAGAAGTATTATCCGCGCTGGACCGTGTACAAGTCTCCTGGAATGGAGCGTCATAGAGGGTGAGAATCCCGTCTTTGACACGGACTGCCAGGGC-TTTGTGATGTGCTCTCGAAGAGTCGAGTTGTTTGGGAATGCAGCTCAAAATGGGTGGTAAATTCCATCTAAAGCTAAATATTGGCGAGAGACCGATAGCGAACAAGTACCGTGAGGGAAAGATGAAAAGAACTTTGGAAAGAGAGTTAAACAGTACGTGAAATTGCTGAAAGGGAAACGCTTGAAGTCAGTCGCGTTGGCCAGGGATCAA-CCTTGC----TTTTTT---GCTTGGCTTACTTTCTGGTCGATGGG-TCAGCATCAATTTTGACTGGTGG-AAAAAGTTCAAGGGAATGTGGCAT-CTTCGGATGTGTTATAGCCTTTGTTCACATATATCAGTTGGGATTGAGGAACTCAGCACGCCGCAAGGCCGGG---TTTTTAACCACGTACGTGCTTAGGATGCTGGCATAATGGCTTTAATCGACCCGTCTTGAAACACGGACCAAGGAGTCTAACATGCCTGCGAGTGTTTGGGTGAAAAACCCGAGCGCGTAATGAAAGTG-AAAGTTGAGATCCCTGTCGTGGGGAGCATTGACGCCCGGATCTGACCTTTTGTGACGATTCTGCGGTAAAGCATGTTTTTCGCATGCTTTTCCGAAAGTTGACCAAGGACGTTTATCGTTATCTACAAAAGgttgctgaccttcgctcaagtgccatgagatgagatgtgctga-cccctct---tcttcagTGCGTCGAGACCCACAAGGAGTTCAACCTTGCCCTTGCAGTTAAGCATCAAACCATCACAAACGGCCTCAAATACTCTCTGGCGACAGGTAACTGGGGAGATCAGAAAAAGTCAATGTCTTCCAAAGCAGGAGTATCTCAGGTCTTGAACAGATRCACATATGCATCCACTCTATCACATCTTCGTCGGTGTAACACTCCTCTAGGGCGCGAAGGCAAGATCGCCAAACCTCGTCAGTTGCACAACACTCATTGGGGTATGGTGTGTCCTGCCGAAACTCCTGAAGGTCAAGCCTGTGGTCTTGTCAAGAACCTCGCCCTTATGGCGTGCATATCAGTCGGGTCTTACTCTGCGCCCGTTATCGAGTTTTTGGAGGAGTGGGGATTGGAGTCATTGGAAGAGAACGCGCACTCATCAACACCTTGCACAAAAGTTTTCGTGAACGGTGTCTGGATGGGTGTGCATCGCGATCCTGCCAATTTGGTGAAGACGATAAAGAAATTGAGAAGGAAAGACGATATTAGCCCAGAAGTGTCAGTTGTGAGAGACATCAGGGAGAAGGAATTGAGGTTGTATACCGATGCTGGACGTGTTTGTCGACCACTCTTCATCGTCGAGAACCAACAGCTGGCACTTCAGAAGAAGCATGTCAAATGG------------------------------------------------------------------------------------------------------------------------------------------------------------------------------------------------------------------------------------------------------------------------------------------------------------------------------------------------------------------------------------------------------------------------------------------------------------------------------------------------------------------------------------------------------------------------------------------------------------------------------------------------------------------------------------------------------------------------------------------------------------------------------------------------------------------------------------------------------------------------------------------------------------------------------------------------------------------------------------------------------------------------------------------------------------------------------------------------------------------------------------------------------------------------------------------------------------------------------------------------------------------------------------------------------------------------------------------------------------

>AWW557_Laccaria__USA_CA_ITS_28S_RPB2_EF1a

CCTGA-TGTGACTGTTAGCTGGC-TTTT-ATGGCAC-TGCTCGT-CCGTCATCTTT-ATCTCT--CC--CCTGTGAAA--TTGGTAGTC-TT-GGATACCT--------------------------G-GATCGCTGTGC---TGT---ACAAGTCGGCTTTCTTTTCATTTCC-AAGACTATGTTTT----T--A-TATACA-CCAAAGTATGTTTAAAGAATGTCATC-AATAGGAAA---TTGTTTCCTA--T-AAAATTATACAACTTTCAGCAACGGATCTCTTGGCTCTCGCATCGATGAAGAACGCAGCGAAATGCGATAAGTAATGTGAATTGCAGAATTCAGTGAATCATCGAATCTTTGAACGCACCTTGCGCTCCTTGGTATTCCGAGGAGCATGCCTGTTTGAGTGTCATTAAA-TTCTCAA-CCTTCCAGC-------TT-TTTATTAGCTTGG--TTAGGCTTGGA-TGT-GGGGG--TTGCGGGCTTC-AT-----T---AATGAGGTCGGCTCTCCTTAAATGCATTAGCGGAACTTTTGTGGACTGTCTATTTGGTGTGATAAATTATCTACGCCGTGGAT-GTGAAGCAGATTTTAT---GAAGTT-CAGCTTCT-AACCGTCC---ATTGACTTGGACAA-----TTTTGACAA-TTTGAAAGCTCAAATTTAAAATCTGGGAGG--GCCGCCTGT-CCGAGTTGTAATCTAGAGAAGTATTATCCGCGCTGGACCGTGTACAAGTCTCCTGGAATGGGGCGTCATAGAGGGTGAGAATCCCGTCTTTGACACGGACTGCCAGGGC-TTTGTGATGTGCTCTCAAAGAGTCGAGTTGTTTGGGAATGCAGCTCAAAATGGGTGGTAAATTCCATCTAAAGCTAAATATTGGCGAGAGACCGATAGCGAACAAGTACCGTGAGGGAAAGATGAAAAGAACTTTGGAAAGAGAGTTAAACAGTACGTGAAATTGCTGAAAGGGAAACGCTTGAAGTCAGTCGCGTTGGCTAGGGATCAA-CCTTGC----TTTTTT---GCTTGGCTTACTTTCTGGTCGATGGG-TCAGCATCAATTTTGACTGATGG-AAAAAGTTCAAGGGAATGTGGCAT-CTTCGGATGTGTTATAGCCTTTGTTCGCATACATTGGTTGGGATTGAGGAACTCAGCACGCCGCAAGGCCGGG-TTTTTTTAACCACGTACGTGCTTAGGATGCTGGCATAATGGCTTTAATCGACCCGTCTTGAAACACGGACCAAGGAGTCTAACATGCCTGCGAGTGTTTGGGTGAAAAACCCGAGCGCGTAATGAAAGTG-AAAGTTGAGATCCCTGTCGTGGGGAGCATTGACGCCCGGATCTGACCTTTTGTGACGATTCTGCGGTAGAGCATGTTTTCCGCATGCTTTTCCGAAAGTTGAGCAAGGATGTTTATCGTTATCTACAAAAGgttgctgaccttcgctcaagtgcc-----atgagatgtgctga-cccctct---tcttcagTGCGTTGAGACCCACAAGGAGTTCAACCTGGCCCTTGCAGTTAAGCATCAAACCATCACAAACGGCCTCAAATACTCTCTGGCGACAGGTAACTGGGGAGATCAGAAAAAGTCAATGTCTTCCAAGGCAGGAGTATCTCAGGTCTTGAACAGATACACATATGCATCCACTCTATCACATCTTCGTCGGTGTAATACTCCTCTAGGGCGCGAAGGCAAGATCGCCAAACCCCGTCAGTTGCACAACACTCATTGGGGTATGGTGTGTCCTGCGGAAACTCCTGAAGGTCAAGCCTGTGGTCTTGTCAAGAACCTCGCCCTTATGGCGTGCATATCAGTCGGGTCTTACTCCGCGCCCGTTATCGAGTTTTTGGAGGAGTGGGGATTGGAGTCATTGGAAGAGAACGCACATTCATCGACACCTTGCACAAAAGTTTTCGTGAACGGTGTCTGGATGGGTGTGCATCGCGATCCTGCCAATTTGGTGAAGACGATAAAGAAATTGAGAAGGAAAGACGACATCAGCCCAGAAGTGTCAGTTGTGCGAGACATCAGRGAGAAGGAATTGAGGTTGTACACCGATGCTGGACGTGTTTGTCGACCACTCTTCATCGTCGAGAACCAACAGCTGGCACTTCAGAAGAAGCACGTCAAATGGCTCAGTAACGGCCTCAACGATGACGGCGACGAGTACAAATGGGAACATCTGGTAAAGGGTGGCATCATCGAGTTACTGGATGCTGAGGAAGAGGAAACGGTGATGATATCCATGACCCCTGAAGATCTTGAAAATTCTCGCCTACAGCAAAGTGGTGTTGATCCTCATGCGAACGACGGCGAGTTTGATCCAGCAGCTCGATTGAAGGCTGGCACTCACGCWCACACATGGACACATTGCGAAATTCATCCTAGCATGATTCTTGGCATTTGCGCCAGCATTATTCCTTTCCCTGATCATCTGCGCCATTCTCATCATTGCTGGTGGTACTGGTGAGTTCGAGGCTGGTATCTCCAAGGATGGCCAGACCCGCGAGCACGCTCTCCTCGCTTTCACCCTCGGTGTGCGGCAACTCATCGTTGCCGTCAACAAGATGGACACCACCAAGGTAAGAAACTTTTGAC-TTTTAGTTAGGCTTCTTTCACATTCTCATCATCTCA-TGTAGTGGAGTGAGGACCGTTTCAACGAAATCATCAAGGAGACGTCAACCTTCATCAAGAAGGTTGGTTACAACCCCAAGGCCGTTGCCTTTGTCCCTATTTCCGGCTGGCACGGTGACAACATGTTGGAGGAGTCCCCTAAGTGAGCATTC--TCGTATCGACATGGTCGCCCCGTCGCTCATGTTGTACTC-AGCATGCCTTGGTACAAGGGCTGGACCAAGGAGACCAAGGCTGGTGGCGTCAAGGGCAAGACCCTCCTCGATGCTATTGATGCCATTGAGCCCCCCGTCCGACCTTCCGACAAACCCCTCCGTCTCCCCCTCCAGGATGTCTACAAAATTGGCGGTATTGGAACTGTGCCCGTCGGTCGTGTTGAGACTGGTATCATCAAGGCCGGAATGGTCGTCACTTTCGCTCCCTCCAACGTCACCACTGAAGTCAAGTCCGTCGAAATGCATCACGAACAGCTCGAGCAGGGTAACCCTGGTGACAATGTCGGTTTCAACGTCAAGAACGTGTCAGTGAAGGATATTCGTCGTGGCAACGTGGCCTCCGACTCCAAGAACGATCCCGCCAAGGAAGCGGCCGCTTTCAACGCACAGGTCATCGTCCTCAACCACCCTGGTCAAATTGGTGCTGGTTACGCCCCAGTTCTCGACTGTCACACCGCCCACATCGCCTGCAAGTTCGCCGAGCTCATCGAGAAGATCGATCGTCGTACGGGTAAATCCATTGAAAACTCGCCTAAATTCGTCAAGTCGGGTGACGCCTGCATCGTCAAGCTCGTTCCCAGCAAGCCTATG

>AWW567_Laccaria_bicolor_USA_MI_KM067824_KU685765__

CCTGA-TG-GACTGTTAGCTGGC-TTTTCAAAGCATGTGCTCGT-CTGTCATCTTT-ATCTCT--CCA-CCTGTGCACATTTTGTAGTC-TT-GGATACCTCTCGAGGCAA-CTCGGATT----TTAG-GATCGCCGTGC---TGT---ACAAGTYGGCTTTTCTTTCATTTCC-AAGACTATGTTTT----T--A-TATACA-CCAAAGTATGTTTAAAGAATGTCATC-AATGGGAAC---TTGTTTCCTA--T-AAAATTATACAACTTTCAGCAACGGATCTCTTGGCTCTCGCATCGATGAAGAACGCAGCGAAATGCGATAAGTAATGTGAATTGCAGAATTCAGTGAATCATCGAATCTTTGAACGCACCTTGCGCTCCTTGGTATTCCGAGGAGCATGCCTGTTTGAGTGTCATTAAA-TTCTCAA-CCTTCCAAC--------T-TTTATTAGCTTGG--TTAGGCTTGGA-TGT-GGGGG--TTGCGGGCTTC-AT-----T---AATGAGGTCGGCTCTCCTTAAATGCATTAGCGGAACTTTTGTGGACCGTCTA-TTGGTGTGAT-AATTATCTACGCCGTGGAT-GTGAARCAG-CTTTAT---GAAGTT-CTGCTTCT-AATCGTCC---ATTGACTTGGACAA-----TTTTGACAA-TTTGAAAGCTCAAATTTAAAATCTGGCAGTCCTTTGGCTGT-CCGAGTTGTAATCTAGAGAAGTATTATCCGCGCTGGACCGTGTACAAGTCTCCTGGAATGGGGCGTCATAGAGGGTGAGAATCCCGTCTTTGACACGGACTGCCAGGGC-TTTGTGATATGCTCTCAAAGAGTCGAGTTGTTTGGGAATGCAGCTCAAAATGGGTGGTAAATTCCATCTAAAGCTAAATATTGGCGAGAGACCGATAGCGAACAAGTACCGTGAGGGAAAGATGAAAAGAACTTTGGAAAGAGAGTTAAACAGTACGTGAAATTGCTGAAAGGGAAACGCTTGAAGTCAGTCGCGTTGGCCAGGGATCAA-CCTTGC----TTTTTT---GCTTGGCTTACTTTCTGGTTGATGGG-TCAGCATCAATTTTGACTGGTGG-AAAAAGTTCAAGGGAATGTGGCAT-CTTCGGATGTGTTATAGCCTTTGTTCACATACATTGGTTGGGATTGAGGAACTCAGCACGCCGCAAGGCCGGG---TTTTTAACCACGTACGTGCTTAGGATGCTGGCATAATGGCTTTAATCGACCCGTCTTGAAACACGGACCAAGGAGTCTAACATGCCTGCGAGTATTTGGGTGAAAAACCCGAGTGCRTAATGAAAGTG-AAAGTTGAGATCCCTGTCGTGGGGAGCATTGACGCCYGGATCTGACCTTTTGTGACGATTCTGCGGTAGAGCATGT-----------------------------------------------------------------------------------------------------------------------------------------------------------------------------------------------------------------------------------------------------------------------------------------------------------------------------------------------------------------------------------------------------------------------------------------------------------------------------------------------------------------------------------------------------------------------------------------------------------------------------------------------------------------------------------------------------------------------------------------------------------------------------------------------------------------------------------------------------------------------------------------------------------------------------------------------------------------------------------------------------------------------------------------------------------------------------------------------------------------------------------------------------------------------------------------------------------------------------------------------------------------------------------------------------------------------------------------------------------------------------------------------------------------------------------------------------------------------------------------------------------------------------------------------------------------------------------------------------------------------------------------------------------------------------------------------------------------------------------------------------------------------------------------------------------------------------------------------------------------------------------------------------------------------------------------------------------------------------------------------------------------------------------------------------------------------------------------------------------------------------------------------------------------

>AWW572_Laccaria_sp_USA_MI_KM067827_KU685767_KU685921_

CCTGA-TGTGACTGTTAGCTGGC-TTTTCGAAGCATGTGCTCGT-CCATCATCTTTAATCTCT--CCA-CCTGTGCACATTTTGTAGTC-TT-GGATACCTCTCGAGGCAA-CTCGGATT----TTAG-GATCGCCG-----------------TTGGCTTTTCTTTCATTTTC-AAGACTATGTTCT----T--A-TATACA-CCAAAGTATGTTTAAAGAATGTCATC-AATAGGAAC---TTGTTTCCTA--T-AAAATTATACAACTTTCAGCAACGGATCTCTTGGCTCTCGCATCGATGAAGAACGCAGCGAAATGCGATAAGTAATGTGAATTGCAGAATTCAGTGAATCATCGAATCTTTGAACGCACCTTGCGCTCCTTGGTATTCCGAGGAGCATGCCTGTTTGAGTGTCATTAAA-TTCTCAA-CCTTCCAAC--------T-TTTATTAGCTTGG--TTAGGCTTGGA-TGT-GGGGG--TTGCGGGCTTC-AT---------CACGAAGTCGGCTCTCCTTAAATGCATTAGCGGAACTTTTGTGGACCGTCTA-TTGGTGTGAT-AATTATCTACGCCGTGGAT-GTGAAGCAG-CTTTAT---GAAGTT-CAGCTTCT-AACCGTCC---ATTGACTTGGACAA-----TTTTGACAA-TTTGAAAGCTCAAATTTAAAATCTGGCAGT-CTTTGGCTGT-CCGAGTTGTAATCTAGAGAAGTATTATCCGCGCTGGACCGTGTACAAGTCTCCTGGAATGGAGCGTCATAGAGGGTGAGAATCCCGTCTTTGACACGGATTGCCAGGGC-TTTGTGATGTGCTCTCGAAGAGTCGAGTTGTTTGGGAATGCAGCTCAAAATGGGTGGTAAATTCCATCTAAAGCTAAATATTGGCGAGAGACCGATAGCGAACAAGTACCGTGAGGGAAAGATGAAAAGAACTTTGGAAAGAGAGTTAAACAGTACGTGAAATTGCTGAAAGGGAAACGCTTGAAGTCAGTCGCGTTGGCCAGGGATCAA-CCTTGC----TTTTTT---GCTTGGCTTACTTTCTGGTCGATGGG-TCAGCATCAATTTTGACTGGTGG-AAAAAGTTCAAGGGAATGTGGCAT-CTTCGGATGTGTTATAGCCTTTGTTCATATATATCAGTTGGGATTGAGGAACTCAGCACGCCGCAAGGCCGGG---TTTTTAACCACGTACGTGCTTAGGATGCTGGCATAATGGCTTTAATCGACCCGTCTTGAAACACGGACCAAGGAGTCTAACATGCCTGCRAGTGTTTGGGTGAAAAACCCGAGCGCGTAATGAAAGTG-AAAGTTGAGATCCCTGTCGTGGGGAGCATTGACGCCCGGATCTGACCTTTTGTGACGATTCTGCGGTAGAGCATGTTTTCCGTATGCTTTTCCGAAAGTTGACCAAGGACGTTTATCGTTATCTACAAAAGgttgctgaccttcgctcaagtgcc-----atgagatgtgctga-cctctct---tcttcagTGCGTCGAGACCCACAAGGAGTTCAACCTTGCCCTTGCAGTTAAGCATCAAACCATAACAAACGGCCTCAAATACTCTCTGGCGACAGGTAACTGGGGAGATCAGAAAAAGTCAATGTCTTCCAAGGCAGGAGTATCTCAGGTCTTGAACAGATACACATATGCATCAACTCTGTCACATCTTCGTCGGTGTAACACTCCTCTAGGGCGCGAAGGCAAGATCGCCAAACCTCGTCAGTTGCACAACACTCATTGGGGTATGGTGTGTCCTGCGGAAACTCCTGAAGGTCAAGCCTGTGGTCTCGTCAAGAACCTCGCCCTTATGGCGTGCATATCAGTCGGGTCTTACTCCGCGCCCGTTATCGAGTTTTTAGAGGAGTGGGGATTGGAGTCATTGGAAGAGAACGCGCACTCATCAACACCTTGCACAAAAGTTTTCGTGAACGGTGTCTGGATGGGTGTACATCGCGATCCTGCCAATTTGGTGAAGACGATAAAGAAATTGAGAAGGAAAGACGACATTAGCCCAGAAGTGTCAGTCGTGCGAGACATCAGGGAGAAGGAATTGAGGTTGTACACCGATGCTGGACGTGTTTGTCGACCACTCTTCATCGTCGAGAACCAACAGCTGGCACTTCAGAAGAAGCACGTCAAATGGCTCAGTAACGGCCTCAACGATGATGGCGACGAGTACAAATGGGAACATCTGGTAAAGGGTGGCATCATTGAATTACTGGATGCTGAGGAAGAGGAAACAGTGATGATATCCATGACCCCTGAAGATCTTGAAAATTCTCGCCTGCAGCAAAGTGGTGTTGATCCTCACGCGAACGACGGCGAGTTTGATCCAGCAGCTCGATTGAAGGCTGGCACTCACGCACACACATGGACACATTGCGAAATTCATCCTAGCATGATTCTTGGCATTTGTGCCAGCATTATTCCTTTCCCTGATCAC------------------------------------------------------------------------------------------------------------------------------------------------------------------------------------------------------------------------------------------------------------------------------------------------------------------------------------------------------------------------------------------------------------------------------------------------------------------------------------------------------------------------------------------------------------------------------------------------------------------------------------------------------------------------------------------------------------------------------------------------------------------------------------------------------------------------------------------------------------------------------------------------------------------------------------------------------------------------------------------------------------------------------------------------

>AWW583_Laccaria_laccata_USA_OR_KM067828_KU685768__

CCTGA-TGTGGCTGTTAGCTGGC-TTTTCGAAGCATGTGCTCGT-CCATCATCTTT-ATCT----CCA-CCTGTGCACATTTTGTAGTC-TT-GGATACCTCTCGAGGCAA-CTCGGATT----TTAG-GATCGCTGTGC---TGT---ACAAGTCGGCTTTCCTTTCATTTCC-AAGACTATGTTTT---TT--A-TATACA-CCAAAGTATGTTTAAARAAKGTCATC-AATGGGA-------------TA--T-AAAATTATACAACTTTCAGCAATGGATCTCTTGGCTCTCGCATCGATGAAGAACGCAGCGAAATGCGATAAGTAATGTGAATTGCAGAATTCAGTGAATCATCGAATCTTTGAACGCACCTTGCGCTCCTTGGTATTCCGAGGAGCATGCCTGTTTGAGTGTCATTAAA-TTCTCAA-CCTTCCAGC--------T-TTTATTAGAGTGG--ATAGGCTTGGA-TGT-GGGGG--TTGCGGGCTTC-AT-----C---AATGAGGTTGGCTCTCCTTAAAAGCATTAGCGGAACTTTTGTGGACCGTCTA-TTGGTGTGAT-AATTATCTACGCCGTGGAT-GTGAAGCAG-CTTTAT---AAAGTT-CAGCTTCT-AACTGTCC---ATTAATTTGGACAA-----TTTTGACAA--TTGGAAGCTCAAATTTAAAATCTGGCAGT-CTTTGGCTGT-CCGAGTTGTAATCTAGAGAAGTATTATCCGCGCTGGACCGTGTACAAGTCTCCTGGAATGGAGCGTCATAGAGGGTGAGAATCCCGTCTTTGACACGGACTGCCAGGGC-TTTGTGATGTGCTCTCAAAGAGTCGAGTTGTTTGGGAATGCAGCTCAAAATGGGTGGTAAATTCCATCTAAAGCTAAATATTGGCGAGAGACCGATAGCGAACAAGTACCGTGAGGGAAAGATGAAAAGAACTTTGGAAAGAGAGTTAAACAGTACGTGAAATTGCTGAAAGGGAAACGCTTGAAGTCAGTCGCGTTGGCCAGGGATCAA-CCTTGC----TTTTTT---GCTTGGCTTACTTTCTGGTTGATGGG-TCAGCATCAATTTTGACTGGTGG-AAAAAGTTCAAGGGAATGTGGCAT-CTTCTGATGTGTTATAGCCTTTGTTCGCATACATCGGTTGGGATTGAGGAACTCAGCACGCCGCAAGGCCGGG---TTTTTAACCACGTACGTGCTTAGGATGCTGGCATAATGGCTTTAATCGACCCGTCTTGAAACACGGACCAAGGAGTCTAACATGCCTGCGAGTGTTTGGGTGAAAAACTCGAGCGCGTAATGAAAGTG-AAAGTTGAGATCCCTGTCATGGGGAGCATTGACGCCCGGATCTGACCTTTTGTGACGATTCTGCGGTAGAGCATGT-----------------------------------------------------------------------------------------------------------------------------------------------------------------------------------------------------------------------------------------------------------------------------------------------------------------------------------------------------------------------------------------------------------------------------------------------------------------------------------------------------------------------------------------------------------------------------------------------------------------------------------------------------------------------------------------------------------------------------------------------------------------------------------------------------------------------------------------------------------------------------------------------------------------------------------------------------------------------------------------------------------------------------------------------------------------------------------------------------------------------------------------------------------------------------------------------------------------------------------------------------------------------------------------------------------------------------------------------------------------------------------------------------------------------------------------------------------------------------------------------------------------------------------------------------------------------------------------------------------------------------------------------------------------------------------------------------------------------------------------------------------------------------------------------------------------------------------------------------------------------------------------------------------------------------------------------------------------------------------------------------------------------------------------------------------------------------------------------------------------------------------------------------------------

>AWW590_Laccaria_amethysteo-occidentalis_USA_OR_JX504112_JX504195_KU685923_

CCTGA-TGTGACTGTTAGCTGGC-TTTTCGAAGCATGTGCTCGT-CCGTCATCTTT-ATCTCT--CCA-CCTGTGCACATTTTGTAGTC-TT-GGATACCT--------------------------G-GATCGCTGTGC---TGT---ACAAGTCGGCTTTCTTTTCATTTCC-AAGACTATGTTTT----T--A-TATACA-CCAAAGTATGTTTAAAGAATGTCATC-AATAGGAAA---TTGTTTCCTA--T-AAAATTATACAACTTTCAGCAACGGATCTCTTGGCTCTCGCATCGATGAAGAACGCAGCGAAATGCGATAAGTAATGTGAATTGCAGAATTCAGTGAATCATCGAATCTTTGAACGCACCTTGCGCTCCTTGGTATTCCGAGGAGCATGCCTGTTTGAGTGTCATTAAA-TTCTCAA-CCTTCCAGC-------TT-TTTATTAGCTTGG--TTAGGCTTGGA-TGT-GGGGG--TTGCGGGCTTC-AT-----T---AATGAGGTCGGCTCTCCTTAAATGCATTAGCGGAACTTTTGTGGACTGTCTATTTGGTGTGATAAATTATCTACGCCGTGGAT-GTGAAGCAGATTTTAT---GAAGTT-CAGCTTCT-AACCGTCC---ATTGACTTGGACAA-----TTTTGACAA-TTTGAAAGCTCAAATTTAAAATCTGGCAGT-CTTTGGCTGT-CCGAGTTGTAATCTAGAGAAGTATTATCCGCGCTGGACCGTGTACAAGTCTCCTGGAATGGGGCGTCATAGAGGGTGAGAATCCCGTCTTTGACACGGACTGCCAGGGC-TTTGTGATGTGCTCTCAAAGAGTCGAGTTGTTTGGGAATGCAGCTCAAAATGGGTGGTAAATTCCATCTAAAGCTAAATATTGGCGAGAGACCGATAGCGAACAAGTACCGTGAGGGAAAGATGAAAAGAACTTTGGAAAGAGAGTTAAACAGTACGTGAAATTGCTGAAAGGGAAACGCTTGAAGTCAGTCGCGTTGGCTAGGGATCAA-CCTTGC----TTTTTT---GCTTGGCTTACTTTCTGGTCGATGGG-TCAGCATCAATTTTGACTGATGG-AAAAAGTTCAAGGGAATGTGGCAT-CTTCGGATGTGTTATAGCCTTTGTTCGCATACATTGGTTGGGATTGAGGAACTCAGCACGCCGCAAGGCCGGG-TTTTTTTAACCACGTACGTGCTTAGGATGCTGGCATAATGGCTTTAATCGACCCGTCTTGAAACACGGACCAAGGAGTCTAACATGCCTGCGAGTGTTTGGGTGAAAAACCCGAGCGCGTAATGAAAGTG-AAAGTTGAGATCCCTGTCGTGGGGAGCATTGACGCCCGGATCTGACCTTTTGTGACGATTCTGCGGTAGAGCATGTTTTCCGCATGCTTTTCCGAAAGTTGAGCAAGGATGTTTATCGTTATCTACAAAAGgttgctgaccttcgctcaagtgcc-----atgagatgtgctga-cccctct---tcttcagTGCGTTGAGACCCACAAGGAGTTCAACCTGGCCCTTGCAGTTAAGCATCAAACCATCACAAACGGCCTCAAATACTCTCTGGCGACAGGTAACTGGGGAGATCAGAAAAAGTCAATGTCTTCCAAGGCAGGAGTATCTCAGGTCTTGAACAGATACACATATGCATCCACTCTRTCACATCTTCGTCGGTGTAATACTCCTCTAGGGCGCGAAGGCAAGATCGCCAAACCCCGTCAGTTGCACAACACTCATTGGGGTATGGTGTGTCCTGCGGAAACTCCTGAAGGTCAAGCCTGTGGTCTTGTCAAGAACCTCGCCCTTATGGCGTGCATATCAGTCGGGTCTTACTCCGCGCCCGTTATCGAGTTTTTGGAGGAGTGGGGATTGGAGTCATTGGAAGAGAACGCACATTCATCGACACCTTGCACAAAAGTTTTCGTGAACGGTGTCTGGATGGGTGTGCATCGCGATCCWGCCAATTTGGTGAAGACGATAAAGAAATTGAGAAGGAAAGACGACATCAGCCCAGAAGTGTCAGTTGTGCGGGAYATCAGRGAGAAGGAATTGAGGTTGTACACCGATGCTGGACGTGTTTGTCGACCACTCTTCATCGTCGAGAACCAACAGCTGGCACTTCAGAAGAAGCACGTCAAATGGCTCAGTAACGGCCTCAACGATGACGGCGACGAGTACAAATGGGAACATCTGGTAAAGGGTGGCATCATCGAGTTACTGGATGCTGAGGAAGAGGAAACGGTGATGATATCCATGACCCCTGAAGATCTTGAAAATTCTCGCCTACAGCAAAGTGGTGTTGATCCTCATGCGAACGACGGCGAGTTTGATCCAGCAGCTCGATTGAAGGCTGGCACTCACGCACACACATGGACACATTGCGAAATTCATCCTAGCATGATTCTTGGCATTTGCGCCAGCATTATTCCTTTCCCTGATCAT------------------------------------------------------------------------------------------------------------------------------------------------------------------------------------------------------------------------------------------------------------------------------------------------------------------------------------------------------------------------------------------------------------------------------------------------------------------------------------------------------------------------------------------------------------------------------------------------------------------------------------------------------------------------------------------------------------------------------------------------------------------------------------------------------------------------------------------------------------------------------------------------------------------------------------------------------------------------------------------------------------------------------------------------

>AWW591_Laccaria_sp_USA_OR_KM067830_28S_RPB2_

CCTGA-TGTGACTGTTAGCTGGC-TTTTCGAAGCATGTGCTCGT-CCATCATCTTT-ATCTCT--CCA-CCTGTGCACATTTTGTAGTC-TT-GCATACCTCTCGAGGAAA-CTCGGATT----TGAG-GATTGCCGTGC---TGT---ACAAGTCGGCTTTTCTTTCATTTCC-AAGACTATGTTTT----T--A-TATACA-CCAAAGTATGTTTATAGAATGTCATC-AATGGGAAC---TTGTTTCCTA--T-AAAATTATACAACTTTCAGCAACGGATCTCTTGGCTCTCGCATCGATGAAGAACGCAGCGAAATGCGATAAGTAATGTGAATTGCAGAATTCAGTGAATCATCGAATCTTTGAACGCACCTTGCGCTCCTTGGTATTCCGAGGAGCATGCCTGTTTGAGTGTCATTAAA-TTCTCAA-CCTTCCAAC--------T-TTTATTAGCTTGG--TTAGGCTTGGA-TGT-GGGGG--TTGCGGGCTTC-AT-----C---ACTGAGGTCGGCTCTCCTTAAATGCATTAGTGGAACTTTTGTGGACCATCTA-TTGGTGTGAT-AATTATCTACGCCGTGGGT-GTGAAGCAG-TTTTAT---GAAGTT-CTGCTTCT-AACCGTCC---ATTGACTTGGACAA-----TTTTGACAA-TTTGAAAGCTCAAATTTAAAATCTGGCAGT-CTTTGGCTGT-CCGAGTTGTAATCTAGAGAAGTATTATCCGCGCTGGACCGTGTACAAGTCTCCTGGAATGGAGCGTCATAGAGGGTGAGAATCCCGTCTTTGACACGGACTGCCAGGGC-TTTGTGATATGCTCTCAAAGAGTCGAGTTGTTTGGGAATGCAGCTCAAAATGGGTGGTAAATTCCATCTAAAGCTAAATATTGGCGAGAGACCGATAGCGAACAAGTACCGTGAGGGAAAGATGAAAAGAACTTTGGAAAGAGAGTTAAACAGTACGTGAAATTGCTGAAAGGGAAACGCTTGAAGTCAGTCGCGTTGGCCAGGGATCAA-CCTTGC------TTTT---GCTTGGCTTACTTTCTGGTCGATGGG-TCAGCATCAATTTTGACTGGTGG-AAAAAGTTCAAGGGAATGTGGCAT-CTTCGGATGTGTTATAGCCTTTGTTCACATACATTGGTTGGGATTGAGGAACTCAGCACGCCGCAAGGCCGGG---TTTTTAACCACGTACGTGCTTAGGATGCTGGCATAATGGCTTTAATCGACCCGTCTTGAAACACGGACCAAGGAGTCTAACATGCCTGCGAGTATTTGGGTGAAAAACCCGAGTGCGTAATGAAAGTG-AAAGTTGAGATCCCTGTCGTGGGGAGCATTGACGCCCGGATCTGACCTTTTGTGACGATTCTGCGGTAGAGCATGTTTTCCGCATGCTTTTCCGAAAGTTGACCAAGGACGTTTATCGTTATCTACAAAAGgttgctgactttcgctcaagtgcc-----atgagacgtgttga-cctctct---tcttcagTGCGTCGAGACCCACAAGGAGTTCAACCTTGCCCTTGCAGTTAAGCATCAAACCATTACAAACGGCCTCAAATACTCTCTGGCGACAGGTAACTGGGGGGATCAGAAAAAGTCAATGTCTTCCAAGGCAGGAGTATCTCAGGTCTTGAACAGATACACATATGCATCCACTCTGTCACATCTTCGTCGGTGTAACACTCCTCTAGGACGCGAAGGCAAGATCGCCAAACCTCGTCAGTTGCACAACACCCATTGGGGTATGGTGTGCCCGGCGGAAACTCCTGAAGGTCAAGCCTGTGGTCTTGTCAAGAACCTCGCCCTTATGGCGTGCATATCAGTCGGGTCTTATTCCGCGCCCGTTATCGAGTTTTTGGAGGAATGGGGATTGGAGTCATTGGAAGAGAACGCACACTCATCAACGCCCTGCACCAAAGTTTTCGTGAAYGGTGTATGGATGGGTGTGCATCGCGATCCTGCCAACTTGGTGAAGACGATAAAGAAATTGAGAAGGAAAGACGACATTAGCCCAGAAGTGTCAGTTGTGCGAGACATCCGGGAGAAGGAGTTGAGGTTGTACACCGATGCTGGACGTGTTTGTCGACCACTCTTCATCGTCGAGAACCAACAGCTGGCACTTCAGAAGAAGCACGTCAAATGGCTCAGTAACGGCCTCAACGATGATGGCGACGAATACAAATGGGAACATTTGGTAAAGGGTGGCATCATTGAGTTACTGGATGCTGAGGAAGAGGAAACGGTGATGATATCCATGACCCCTGAAGATCTTGAAAATTCTCGCCTACAGCAAAGTGGCGTTGACCCTCACGCGAACGACGGCGAGTTTGATCCAGCAGCTCGATTAAAGGCTGGCACTCACGCACACACATGGACACATTGCGAAATTCATCCTAGCATGATTCTTGGCATTTGTGCCAGCATTATTCCTTTCCCCGATCAT------------------------------------------------------------------------------------------------------------------------------------------------------------------------------------------------------------------------------------------------------------------------------------------------------------------------------------------------------------------------------------------------------------------------------------------------------------------------------------------------------------------------------------------------------------------------------------------------------------------------------------------------------------------------------------------------------------------------------------------------------------------------------------------------------------------------------------------------------------------------------------------------------------------------------------------------------------------------------------------------------------------------------------------------

>AWW595_Laccaria_sp_USA_AK_JX504115_JX504198__

CCTGA-TGTGATTGTTAGCTGGC-TTTTCGAAGCATGTGCTCGT-CTGTCATATTT-ATCTCT--CCA-CCTGTGCACATTTTGTAGTC-TT-GGATACCTCTCGAGGAAA-CTCGGATT----TTAG-GATCGCTGTGC---TGT---ACAAGTCGGCTTTTCTTTCATTTCC-AAGACTATGTTTT----T--A-TATACA-CCAAAGTATGTTTATAGAATGTCATC-AATGGGAAC---TTGTTTCCTA--T-AAAATTATACAACTTTCAGCAACGGATCTCTTGGCTCTCGCATCGATGAAGAACGCAGCGAAATGCGATAAGTAATGTGAATTGCAGAATTCAGTGAATCATCGAATCTTTGAACGCACCTTGCGCTCCTTGGTATTCCGAGGAGCATGCCTGTTTGAGTGTCATTAAA-TTCTCAA-CCTTCCAAC--------T-TTTATTAGCTTGG--TTAGGCTTGGA-TGT-GGGGG--TTGCGGGCTTC-AA-----T---AATGAGGTCGGCTCTCCTTAAATGCATTAGTGGAACTTTTGTGGACCGTCTA-TTGGTGTGAT-AATTATCTACGCCGTGGAT-GTGAAGCAG-CTTTAT---GAAGTT-CTGCTTCT-AACCGTCC---ATTGACTTGGACAA----TTTTTGACAA-TTTGAAAGCTCAAATTTAAAATCTGGCGGT-CTTTGGCTGT-CCGAGTTGTAATCTAGAGAAGTATTATCCGCGCTGGACCGTGTACAAGTCTCCTGGAATGGAGCGTCATAGAGGGTGAGAATCCCGTCTTTGACACGGACTGCCAGGGC-TTTGTGATATGCTCTCAAAGAGTCGAGTTGTTTGGGAATGCAGCTCAAAATGGGTGGTAAATTCCATCTAAAGCTAAATATTGGCGAGAGACCGATAGCGAACAAGTACCGTGAGGGAAAGATGAAAAGAACTTTGGAAAGAGAGTTAAACAGTACGTGAAATTGCTGAAAGGGAAACGCTTGAAGTCAGTCGCGTTGGCCAGGGATCAA-CCTTGC----TTTTTT---GCTTGGCTTACTTTCTGGTCGATGGG-TCAGCATCAATTTTGACCGGTGG-AAAAAGTYCAAAGGAATGTGGCAT-CTTCGGATGTGTTATAGCCTTTGTTCACATACATTGGTTGGGATTGAGGAACTCAGCACGCCGCAAGGCCGGG---TTTTTAACCACGTACGTGCTTAGGATGCTGGCATAATGGCTTTAATCGACCCGTCTTGAAACACGGACCAAGGAGTCTAACATGCCTGCGAGTATTTGGGTGAAAAACCCGAGTGCGTAATGAAAGTG-AAAGTTGAGATCCCTGTCGTGGGGAGCATTGACGCCCGGATCTGACCTTTTGTGACGATTCTGCGGTAGAGCATGT-----------------------------------------------------------------------------------------------------------------------------------------------------------------------------------------------------------------------------------------------------------------------------------------------------------------------------------------------------------------------------------------------------------------------------------------------------------------------------------------------------------------------------------------------------------------------------------------------------------------------------------------------------------------------------------------------------------------------------------------------------------------------------------------------------------------------------------------------------------------------------------------------------------------------------------------------------------------------------------------------------------------------------------------------------------------------------------------------------------------------------------------------------------------------------------------------------------------------------------------------------------------------------------------------------------------------------------------------------------------------------------------------------------------------------------------------------------------------------------------------------------------------------------------------------------------------------------------------------------------------------------------------------------------------------------------------------------------------------------------------------------------------------------------------------------------------------------------------------------------------------------------------------------------------------------------------------------------------------------------------------------------------------------------------------------------------------------------------------------------------------------------------------------------

>BAP360_Laccaria_negrimarginata TYPE_CHINA_TIBET_JX504120___

CCTGA-TGTGGCTGTCAGCTGGC-TTCT----GCATGTGCTCGT-TCATCATCTTT-ATCTCT--CCA-CCTGTGCACATTTTGTAGAC-TT-GGATA--------------------------ATGG---------------------------------CTCTGTCATTTAC-AAGACTATGTTAT----C--AATATAC--CCAAAGCATGTTTAAAGAATGTCATC-AATAGGAACT--TGGTTTCCTAT-TAGAAACTATACAACTTTCAGCAACGGATCTCTTGGCTCTCGCATCGATGAAGAACGCAGCGAAATGCGTTAAGTAATGTGAATTGCAGAATTCAGTGAATCATCGAATCTTTGAACGCACCTTGCGCTCCTTGGTATTCCGAGGAGCATGCCTGTTTGAGTGTCATTAAA-TTCTCAA-CCTT---------------TCCATTAATTTGK--TTTGGCTTGGA-TGT-GGGGG--TTGCAGGCTTC-ATTTGACT----ATGAGGTCAGCTCTCCTTAAATGCATTAGTGGAACTTTTGTTGACCATCTA-TTGGTGTGAT-ATCTATCTACACTGTGGAT-GTGAAGCAG------------TGTT-CAGCTTCT-AACCGTCTATTATTGACTTT-AC---------TTGACCA-TTTGA-------------------------------------------------------------------------------------------------------------------------------------------------------------------------------------------------------------------------------------------------------------------------------------------------------------------------------------------------------------------------------------------------------------------------------------------------------------------------------------------------------------------------------------------------------------------------------------------------------------------------------------------------------------------------------------------------------------------------------------------------------------------------------------------------------------------------------------------------------------------------------------------------------------------------------------------------------------------------------------------------------------------------------------------------------------------------------------------------------------------------------------------------------------------------------------------------------------------------------------------------------------------------------------------------------------------------------------------------------------------------------------------------------------------------------------------------------------------------------------------------------------------------------------------------------------------------------------------------------------------------------------------------------------------------------------------------------------------------------------------------------------------------------------------------------------------------------------------------------------------------------------------------------------------------------------------------------------------------------------------------------------------------------------------------------------------------------------------------------------------------------------------------------------------------------------------------------------------------------------------------------------------------------------------------------------------------------------------------------------------------------------------------------------------------------------------------------------------------------------------------------------------------------------------------------------------------------------------------------------------------------------------------------------------------------------------------------------------------------------------------------------------------------------------------------------------------------------------------------------------------------------------------------------------------------

>Cripps1625_Laccaria_pseudomontana_TYPE_USA_CO_DQ149871___

CCTGA-TGTGGCTGTTAGCTGGC-TTTTCAAAGCATGTGCTCGT-CCGTCATCTTTAATTTCT--CCA-CCTGTGCACATTTTGTAGTC-TT-GGATACCTCTCGAGGCAA-CTCGGATT----TTAG-GATCGCCGTGC---TGT---AAAAGTCAGCTTTCCTCTCATTTCC-AAGACTATGTTTT----C--A-TATACA-CCAAAGTATGTTTAAAGAATGTCATC-AATGGGAAC---TTGTTTCCTA--T-AAAATTATACAACTTTCAGCAACGGATCTCTTGGCTCTCGCATCGATGAAGAACGCAGCGAAATGCGATAAGTAATGTGAATTGCAGAATTCAGTGAATCATCGAATCTTTGAACGCACCTTGCGCTCCTTGGTATTCCGAGGAGCATGCCTGTTTGAGTGTCATTAAA-TTCTCAA-CCTTCCAAC--------T-TTTATTAGCTTGG--TTAGGCTTGGA-TGT-GGGGG--TTGCGGGCTTC-AT-----C---AATGAGGTCGGCTCTCCTTAAATGCATTAGCGGAACTTTTGTGGACCGTCTA-TTGGTGTGAT-AATTATCTACGCCGTGGAT-TTGAAGCAG-CTTTAT---GAAGTT-CAGCCTCT-AACCGTCC---ATTGACTTGGACAA-----TTTTGACAA-TTTGA-------------------------------------------------------------------------------------------------------------------------------------------------------------------------------------------------------------------------------------------------------------------------------------------------------------------------------------------------------------------------------------------------------------------------------------------------------------------------------------------------------------------------------------------------------------------------------------------------------------------------------------------------------------------------------------------------------------------------------------------------------------------------------------------------------------------------------------------------------------------------------------------------------------------------------------------------------------------------------------------------------------------------------------------------------------------------------------------------------------------------------------------------------------------------------------------------------------------------------------------------------------------------------------------------------------------------------------------------------------------------------------------------------------------------------------------------------------------------------------------------------------------------------------------------------------------------------------------------------------------------------------------------------------------------------------------------------------------------------------------------------------------------------------------------------------------------------------------------------------------------------------------------------------------------------------------------------------------------------------------------------------------------------------------------------------------------------------------------------------------------------------------------------------------------------------------------------------------------------------------------------------------------------------------------------------------------------------------------------------------------------------------------------------------------------------------------------------------------------------------------------------------------------------------------------------------------------------------------------------------------------------------------------------------------------------------------------------------------------------------------------------------------------------------------------------------------------------------------------------------------------------------------------------------------------

>Cripps1771_Laccaria_pseudomontana_USA_CO_DQ149870___

CCTGA-TGTGGCTGTTAGCTGGC-TTTTCAAAGCATGTGCTCGT-CCGTCATCTTTAATTTCT--CCA-CCTGTGCACATTTTGTAGTC-TT-GGATACCTCTCGAGGCAA-CTCGGATT----TTAG-GATCGCCGTGC---TGT---AAAAGTCAGCTTTCCTCTCATTTCC-AAGACTATGTTTT----C--A-TATACA-CCAAAGTATGTTTAAAGAATGTCATC-AATGGGAAC---TTGTTTCCTA--T-AAAATTATACAACTTTCAGCAACGGATCTCTTGGCTCTCGCATCGATGAAGAACGCACCGAAATGCGATAAGTAATGTGAATTGCAGAATTCAGTGAATCATCGAATCTTTGAACGCACCTTGCGCTCCTTGGTATTCCGAGGAGCATGCCTGTTTGAGTGTCATTAAA-TTCTCAA-CCTTCCAGC--------T-TTTATTAGCTTGG--TTAGGCTTGGA-TGT-GGGGG--TTGCGGGCTTC-AT-----C---AATGAGGTCGGCTCTCCTTAAATGCATTAGCGGAACTTTTGTGGACCGTCTA-TTGGTGTGAT-AATTATCTACGCCGTGGAT-TTGAAGCAG-CTTTAT---GAAGTT-CAGCCTCT-AACCGTCC---ATTGACTTGGACAA-----TTTTGACAA-TTTGA-------------------------------------------------------------------------------------------------------------------------------------------------------------------------------------------------------------------------------------------------------------------------------------------------------------------------------------------------------------------------------------------------------------------------------------------------------------------------------------------------------------------------------------------------------------------------------------------------------------------------------------------------------------------------------------------------------------------------------------------------------------------------------------------------------------------------------------------------------------------------------------------------------------------------------------------------------------------------------------------------------------------------------------------------------------------------------------------------------------------------------------------------------------------------------------------------------------------------------------------------------------------------------------------------------------------------------------------------------------------------------------------------------------------------------------------------------------------------------------------------------------------------------------------------------------------------------------------------------------------------------------------------------------------------------------------------------------------------------------------------------------------------------------------------------------------------------------------------------------------------------------------------------------------------------------------------------------------------------------------------------------------------------------------------------------------------------------------------------------------------------------------------------------------------------------------------------------------------------------------------------------------------------------------------------------------------------------------------------------------------------------------------------------------------------------------------------------------------------------------------------------------------------------------------------------------------------------------------------------------------------------------------------------------------------------------------------------------------------------------------------------------------------------------------------------------------------------------------------------------------------------------------------------------------------

>F1061267_Hydnangium_vinaceoavellanea_JAPAN_JX504121___

-------GTGGCTGTTAGCTGGC-TTTTCGAAGCATGTGCTCGC-CCATCATCTTT-ATCTCT--CCA-CCTGTGCACCTTTTGTAGTC-TT-GAATACCTCTCGAGGCAA-CTCGGATT----TTAG-GATTGCCGTGC-TTTATACACAAAGTCGGCTTTTCTTTCATTTTC-AAGACTATGTTTT---CT--A-TACACA-CCAAAGTATGTTTAAAGAACGTCATC-AATAGGAAC---TTGTTTCCTA--TAAAAACTATACAACTTTCAGCAACGGATCTCTTGGCTCTCGCATCGATGAAGAACGCAGCGAAATGCGATAAGTAATGTGAATTGCAGAATTCAGTGAATCATCGAATCTTTGAACGCACCTTGCGCTCCTTGGTATTCCGAGGAGCATGCCTGTTTGAGTGTCATTAAA-TTCTCAACCTTTCCAGC--------T-TTTATTAGTTTGG--TCAGGCTTGGA-TGT-GGGGG--TTGCAGGCTTC-AT-----T-------TAGTCTGCTCTCCTTAAATGCATTAGCGGAACTTTTGTGGACCGTCTA-TTGGTGTGAT-AATTATCTACACCGTGGAT-GTAAAGCAGTTTTTAT-AAAAAGTT-CAGCTCCT-AACTGTCC---ATTGACTTGGACAA-----TTTTGACAA-TTTGA-------------------------------------------------------------------------------------------------------------------------------------------------------------------------------------------------------------------------------------------------------------------------------------------------------------------------------------------------------------------------------------------------------------------------------------------------------------------------------------------------------------------------------------------------------------------------------------------------------------------------------------------------------------------------------------------------------------------------------------------------------------------------------------------------------------------------------------------------------------------------------------------------------------------------------------------------------------------------------------------------------------------------------------------------------------------------------------------------------------------------------------------------------------------------------------------------------------------------------------------------------------------------------------------------------------------------------------------------------------------------------------------------------------------------------------------------------------------------------------------------------------------------------------------------------------------------------------------------------------------------------------------------------------------------------------------------------------------------------------------------------------------------------------------------------------------------------------------------------------------------------------------------------------------------------------------------------------------------------------------------------------------------------------------------------------------------------------------------------------------------------------------------------------------------------------------------------------------------------------------------------------------------------------------------------------------------------------------------------------------------------------------------------------------------------------------------------------------------------------------------------------------------------------------------------------------------------------------------------------------------------------------------------------------------------------------------------------------------------------------------------------------------------------------------------------------------------------------------------------------------------------------------------------------------------

>F1098987_Laccaria_bicolor_USA_NY_KM067813_F1098987_RPB2_F1098987

-CTGA-TGTGACTGTTAGCTGGC-TTTTCGAAGCATGTGCTCGT-CCATCATCTTT-ATCTCT--CCA-CCTGTGCACATTTTGTAGTC-TTAGGATACCTCTCGAGGAAA-CTCGGATT----TGAG-GATCGCCGTGC---TGT---ACAAGTCGGCTTTTCTTTCATTTCC-AAGACTATGTTTT----T--A-TATACA-CCAAAGTATGTTTATAGAATGTCATC-AATGGGAAC---TTGTTTCCTA--T-AAAATTATACAACTTTCAGCAACGGATCTCTTGGCTCTCGCATCGATGAAGAACGCAGCGAAATGCGATAAGTAATGTGAATTGCAGAATTCAGTGAATCATCGAATCTTTGAACGCACCTTGCGCTCCTTGGTATTCCGAGGAGCATGCCTGTTTGAGTGTCATTAAA-TTCTCAA-CCTTCCAAC--------T-TTTATTAGCTTGG--TTAGGCTTGGA-TGT-GGGGG--TTGCGGGCTTC-AT-----C---ACTGAGGTCGGCTCTCCTTAAATGCATTAGTGGAACTTTTGTGGACCATCTA-TTGGTGTGAT-AATTATCTACGCCGTGGGT-GTGAAGCAG-CTTTAT---GAAGTT-CTGCTTCT-AACCGTCC---ATTGACTTGGACAA-----TTTTGACAA-TTTGAAAGCTCAAATTTAAAATCTGGCAGT-CTTTGGCTGT-CCGAGTTGTAATCTAGAGAAGTATTATCCGCGCTGGACCGTGTACAAGTCTCCTGGAATGGAGCGTCATAGAGGGTGAGAATCCCGTCTTTGACACGGACTGCCAGGGC-TTTGTGATATGCTCTCAAAGAGTCGAGTTGTTTGGGAATGCAGCTCAAAATGGGTGGTAAATTCCATCTAAAGCTAAATATTGGCGAGAGACCGATAGCGAACAAGTACCGTGAGGGAAAGATGAAAAGAACTTTGGAAAGAGAGTTAAACAGTACGTGAAATTGCTGAAAGGGAAACGCTTGAAGTCAGTCGCGTTGGCCAGGGATCAA-CCTTGC------TTTT---GCTTGGCTTACTTTCTGGTCGATGGG-TCAGCATCAATTTTGACTGGTGG-AAAAAGTTCAAGGGAATGTGGCAT-CTTCGGATGTGTTATAGCCTTTGTTCACATACATTGGTTGGGATTGAGGAACTCAGCACGCCGCAAGGCCGGG---TTTTTAACCACGTACGTGCTTAGGATGCTGGCATAATGGCTTTAATCGACCCGTCTTGAAACACGGACCAAGGAGTCTAACATGCCTGCGAGTATTTGGGTGAAAAACCCGAGTGCGTAATGAAAGTG-AAAGTTGAGATCCCTGTCGTGGGGAGCATTGACGCCCGGATCTGACCTTTTGTGACGATTCTGCGGTAGAGCATGTTTTCCGCATGCTTTTCCGAAAGTTGACCAAGGACGTTTATCGTTATCTACAAAAGGTTGCTGACTTTCGCTCAAGTGCC-----ATGAGACGTGTTGA-CCTCT-----TCTTCAGTGCGTCGAGACCCACAAGGAGTTTAACCTTGCCCTTGCAGTTAAGCATCAAACCATCACAAACGGCCTCAAATACTCTCTGGCGACAGGTAACTGGGGGGATCAGAAAAAGTCAATGTCTTCCAAGGCAGGAGTATCTCAGGTCTTGAACAGATACACATATGCATCCACCCTGTCACATCTTCGTCGGTGTAACACTCCTCTAGGGCGCGAAGGCAAGATCGCCAAACCTC----------------------------------------------------------------------------------------------------------------------------------------------------------------------------------------------------------------------------------------------------------------------------------------------------------------------------------------------------------------------------------------------------------------------------------------------------------------------------------------------------------------------------------------------------------------------------------------------------------------------------------------------------------------------------------------------------------------------------------------------------------------------------------------------------------------------------------------------------------------------------------------------------------TAAGAAAAGTTTCAC-TTATAGTTAGGCTTCTTYCACATGCTCATCACCTCA-TGCAGTGGAGCGAGGACCGTTTCAACGAAATTATCAAGGAGACGTCAAGCTTCATCAAGAAGGTTGGTTACAACCCCAAGGCCGTTGCCTTTGTTCCTATTTCCGGCTGGCACGGTGACAACATGTTGGAGGAGTCCCCTAAGTAAGTATTC--TCGTATCGTCATTATTGCGGGGTCGCTCATGTCACACTC-AGCATGCCTTGGTTCAAGGGCTGGACCAAGGAGACCAAGGCCGGTGTCGTCAAGGGCAAGACCCTCCTCGATGCTATTGATGCCATTGAGCCCCCCGTCCGACCTTCCGACAAGCCCCTCCGTCTTCCCCTCCAGGAYGTCTACAAAATTGGCGGTATCGGAACTGTGCCCGTCGGTCGTGTTGAGACTGGTATCATCAAGGCCGGAATGGTCGTCAATTTCGCTCCCTCCAACGTCACCACTGAAGTCAAGTCCGTCGAAATGCATCACGAACAGCTCGAGCAGGGTAACCCTGGTGACAATGTCGGTTTCAACGTCAAGAACGTGTCAGTGAAGGATATTCGTCGTGGCAACGTGGCCTCCGACTCCAAGAACGATCCCGCCAAGGAAGCGGCCTCTTTCAACGCACAGGTCATCGTCCTCAACCACCCTGGTCAAATTGGTGCTGGTTATGCCCCGGTTCTCGACTGTCACACCGCCCACATCGCCTGCAAGTTCGCCGAGCTCATCGAAAAGATCGATCGTCGTACGG--------------------------------------------------------------------------------

>F1121424_Laccaria_striatula_CHINA_JX504127_JX504207_KU686064_

-CTGA-TGTGACTGTTAGCTGGC-TTTTCGAAGCATGTGCTCGT-CCGTCATCTTTAATCTCT--CCA-CCTGTGCACATTTTGTAGTC-TTCGGATACCTCTCGAGGCAA-CTCGGATT----TTAG-GATCGCCGTGC---TGT---ACAAGTCGACTTTTCTTTCATTTCC-AAGACTATGTTTT----T--A-TATACA-CCAAAGTATGTTTAAAGAATGTCATC-AATAGGAAC---TTGTTTCCTA--T-AAAATTATACAACTTTCAGCAACGGATCTCTTGGCTCTCGCATCGATGAAGAACGCAGCGAAATGCGATAAGTAATGTGAATTGCAGAATTCAGTGAATCATCGAATCTTTGAACGCACCTTGCGCTCCTTGGTATTCCGAGGAGCATGCCTGTTTGAGTGTCATTAAA-TTCTCAA-CCTTCYATG--------T-TTTATTAGCATGG--TTAGGCTTGGA-TGT-GGGGG--TTGCGGGCTTC-AT-----C---AATGATGTCAGCTCTCCTTAAATGCATTAGCGGAACTTTTGTGGACCGTCTA-TTGGTGTGAT-AGTTATCTACGCCGTGGAT-GTGATGCAG-CTTTAT---GAAGTT-CAGCTTCT-AACCGTCC---ATTGACTTGGACAA-----TTTTGACAA-TTTGAAAGCTCAAATTTAAAATCTGGCAGT-CTTTGGCTGT-CCGAGTTGTAATCTAGAGAAGTATTATCCGCGCTGGACCGTGTACAAGTCTCCTGGAATGGAGCGTCATAGAGGGTGAGAATCCCGTCTTTGACACGGACTGCCAGGGC-TTTGTGATGTGCTCTCGAAGAGTCGAGTTGTTTGGGAATGCAGCTCAAAATGGGTGGTAAATTCCATCTAAAGCTAAATATTGGCGAGAGACCGATAGCGAACAAGTACCGTGAGGGAAAGATGAAAAGAACTTTGGAAAGAGAGTTAAACAGTACGTGAAATTGCTGAAAGGGAAACGCTTGAAGTCAGTCGCGTTGGCCAGGGATCAA-CCTTGC----TTTTTT---GCTTGGCTTACTTTCTGGTCGATGGG-TCAGCATCAATTTTGACTGGTAG-AAAAAGTTCAAGGGAATGTGGCAT-CTTCGGATGTGTTATAGCCCTTGTTCACATATATCAGTTGGAATTGAGGAACTCAGCACGCCGCAAGGCCGGG---TTTTTAACCACGTACGTGCTTAGGATGCTGGCATAATGGCTTTAATCGACCCGTCTTGAAACACGGACCAAGGAGTCTAACATGCCTGCGAGTGTTTGGGTGAAAAACCCGAGCGCATAATGAAAGTG-AAAGTTGAGATCCCTGTCATGGGGAGCATTGACGCCCGGATCTGACCTTTTGTGACGATTCTGCGGTAGAGCATGTTTTCCGTATGCTTTTCCGAAAGTTGACCAAGGACGTTTATCGTTATCTACAAAAGGTTGCTGACCTTCGCTCAAGTGCC-----ATGAGATGTGCTGA-CCTCTCT---TCTTCAGTGCGTCGAGACCCACAAGGAGTTCAACCTTGCCCTTGCGGTTAAGCATCAAACCATCACAAACGGCCTCAAATACTCTCTGGCGACAGGTAACTGGGGAGATCAGAAAAAGTCAATGTCTTCCAAGGCAGGAGTATCTCAGGTCTTGAACAGATACACATATGCATCCACTCTGTCACATCTTCGTCGGTGTAACACTCCTCTAGGGCGCGAAGGCAAGATCGCCAAACCTCGTCAGTTGCACAACACCMATTGGGG----------------------------------------------------------------------------------------------------------------------------------------------------------------------------------------------------------------------------------------------------------------------------------------------------------------------------------------------------------------------------------------------------------------------------------------------------------------------------------------------------------------------------------------------------------------------------------------------------------------------------------------------------------------------------------------------------------------------------------------------------------------------------------------------------------------------------------------------------------------------------------------------------------------------------------------------------------------------------------------------------------------------------------------------------------------------------------------------------------------------------------------------------------------------------------------------------------------------------------------------------------------------------------------------------------------------------------------------------------------------------------------------------------------------------------------------------------------------------------------------------------------------------------------------------------------------------------------------------------------------------------------------------------------------------------------------------------------------------------------------------------------------------

>F1123822_Laccaria_amethystea_USA_WI_KU685760_KU685911_KU686071_

-CTGA-TGTGACTGTTAGCTGGC-TTTTCGAAGCATGTGCTCGT-CCGTCATCTTT-ATCCCT--CCA-CCTGTGCACATTTTGTAGTC-TT-GGATACCTCTCGAGGCAA-CTCGGATT----TTAG-GATCGCTCTGC---TGT---ACAAGTTGGCTTTCCTTTCATTTCC-AAGACTATGTTTT----T--A-TATACA-CCAAAGTATGTTTAAAGAATGTTATC-AATAGGAAC---TTGTTTCCTA--T-AAAATTATACAACTTTCAGCAACGGATCTCTTGGCTCTCGCATCGATGAAGAACGCAGCGAAATGCGATAAGTAATGTGAATTGCAGAATTCAGTGAATCATCGAATCTTTGAACGCACCTTGCGCTCCTTGGTATTCCGAGGAGCATGCCTGTTTGAGTGTCATTAAA-TTCTCAA-CCTTCCAGC--------TTTTTATTAGCTTGG--TTTGGCTTGGA-TGT-GGGGG--TTGCAGGCTTC-AT-----T---AATGAGGTCGGCTCTCCTTAAATGCATTAGCGGAACTTTTGTGGACCGTCTA-TTGGTGTGAT-AATTATCTACGCCGTGGAT-GTGAAGCAG-CTTTAT-------------------------------------------A--------------------AAGCTCAAATTTAAAATCTGGCAGT-CTTTGGCTGT-CCGAGTTGTAATCTAGAGAAGTATTATCCGCGCTGGACCGTGTACAAGTCTCCTGGAATGGGGCGTCATAGAGGGTGAGAATCCCGTCTTTGACACGGACTGCCAGGGC-TTTGTGATGTGCTCTCAAAGAGTCGAGTTGTTTGGGAATGCAGCTCAAAATGGGTGGTAAATTCCATCTAAAGCTAAATATTGGCGAGAGACCGATAGCGAACAAGTACCGTGAGGGAAAGATGAAAAGAACTTTGGAAAGAGAGTTAAACAGTACGTGAAATTGCTGAAAGGGAAACGCTTGAAGTCAGTCGCGTTGGCCAGGGATCAA-CCTTGC-----TTTTT---GCTTGGCTTACTTTCTGGTTAATGGG-TCAGCATCAATTTTGACTGGTGG-AAAAAGTTCAAGGGAATGTGGCAT-CTTCGGATGTGTTATAGCCTTTGTTCGCATACATTGGTTGGGATTGAGGAACTCAGCACGCCGCAAGGCCGGG---TTTTTAACCACGTACGTGCTTAGGATGCTGGCATAATGGCTTTAATCGACCCGTCTTGAAACACGGACCAAGGAGTCTAACATGCCTGCGAGTGTTTGGGTGAAAAACCCGAGCGCGTAATGAAAGTG-AAAGTTGAGATCCCTGTCGTGGGGAGCATTGACGCCCGGATCTGACCTTTTGTGACGATTCTGCGGTAGAGCATGTTTTCCGCATGCTTTTCCGGAAGTTAACCAAGGACGTTTATCGYTATCTACAAAAGGTTGCTGACCTTCGTTTAAGTGCC-----ATGAGATGTGCTGACCCCTTTT---TTTTCAGTGCGTCGAGACCCACAAGGAGTTCAACCTTGCCCTTGCAGTTAAGCATCAAACCATCACAAATGGCCTCAAATACTCTCTGGCGACAGGTAACTGGGGTGATCAGAAAAAGTCAATGTCTTCCAAGGCAGGAGTATCTCAGGTCTTGAACAGATACACATATGCATCCACCTTGTCACATCTTCGTCGGTGTAACACTCCTCTAGGGCGCGAGGGCAAGATCGCCAAACCTCGTCAGTTGCACAACACTMATTGGGG----------------------------------------------------------------------------------------------------------------------------------------------------------------------------------------------------------------------------------------------------------------------------------------------------------------------------------------------------------------------------------------------------------------------------------------------------------------------------------------------------------------------------------------------------------------------------------------------------------------------------------------------------------------------------------------------------------------------------------------------------------------------------------------------------------------------------------------------------------------------------------------------------------------------------------------------------------------------------------------------------------------------------------------------------------------------------------------------------------------------------------------------------------------------------------------------------------------------------------------------------------------------------------------------------------------------------------------------------------------------------------------------------------------------------------------------------------------------------------------------------------------------------------------------------------------------------------------------------------------------------------------------------------------------------------------------------------------------------------------------------------------------------

>GMM7020_Laccaria_proxima_RUSSIA_KU685652_KU685795_KU685938_

CCTGA-TGTGGCTGTTAGCTGGC-TTTTCGAAGCATGTGCTCGT-YCATCATCTTT-ATCT----CCA-CCTGTGCACATTTTGTAGTC-TT-GGATACCTCTCGAGGCAA-CTCGGATT----TTAG-GATCGCTGTGC---TGT---ACAAGTCGGCTTTCCTTTCATTTCC-AAGACTATGTTTT--TTT--A-TATACA-CCAAAGTATGTTTAAAGAATGTCATC-AATGGGA-------------TA--T-AAAATTATACAACTTTCAGCAATGGATCTCTTGGCTCTCGCATCGATGAAGAACGCAGCGAAATGCGATAAGTAATGTGAATTGCAGAATTCAGTGAATCATCGAATCTTTGAACGCACCTTGCGCTCCTTGGTATTCCGAGGAGCATGCCTGTTTGAGTGTCATTAAA-TTCTCAA-CCTTCCAGC--------T-TTTATTAGCTTGG--ATAGGCTTGGA-TGT-GGGGG--TTGCGGGCTTC-AT-----C---AAYGAGGTTGGCTCTCCTTAAAAGCATTAGCGGAACTTTTGTGGACCGTCTA-TTGGTGTGAT-AATTATCTACGCCGTGGAT-GTGAAGCAA-CTTTAT---AAAGTT-CAGCTTCT-AACTGTCC---ATTAACTTGGACAA-----TTTT-----------AAGCTCAAATTTAAAATCTGGCAGT-CTTTGGCTGT-CCGAGTTGTAATCTAGAGAAGTATTATCCGCGCTGGACCGTGTACAAGTCTCCTGGAATGGAGCGTCATAGAGGGTGAGAATCCCGTCTTTGACACGGACTGCCAGGGC-TTTGTGATGTGCTCTCAAAGAGTCGAGTTGTTTGGGAATGCAGCTCAAAATGGGTGGTAAATTCCATCTAAAGCTAAATATTGGCGAGAGACCGATAGCGAACAAGTACCGTGAGGGAAAGATGAAAAGAACTTTGGAAAGAGAGTTAAACAGTACGTGAAATTGCTGAAAGGGAAACGCTTGAAGTCAGTCGCGTTGGCCAGGGATCAA-CCTTGC----TTTTTT---GCTTGGCTTACTTTCTGGTTGATGGG-TCAGCATCAATTTTGACTGGTGG-AAAAAGTTCAAGGGAATGTGGCAT-CTTCTGATGTGTTATAGCCTTTGTTCGCATACATCGGTTGGGATTGAGGAACTCAGCACGCCGCAAGGCCGGG---TTTTTAACCACGTACGTGCTTAGGATGCTGGCATAATGGCTTTAATCGACCCGTCTTGAAACACGGACCAAGGAGTCTAACATGCCTGCGAGTGTTTGGGTGAAAAACTCGAGCGCGTAATGAAAGTG-AAAGTTGAGATCCCTGTCATGGGGAGCATTGACGCCCGGATCTGACCTTTTGTGACGATTCTGCGGTAGAGCATGTTTTCCGCATGCTTTTCCGAAAGTTGACCAAGGACGTTTATCGTTATCTACAAAAGGTTGCTGACCTTCGTTCAAGCGCC-----ATGAGATCTGCTGA-CCCTTCT---TTTTCAGTGCGTCGAGGCCCACAAGGAGTTCAACCTTGCCCTTGCAGTTAAGCATCAAACCATCACAAACGGCCTCAAATACTCTCTGGCGACAGGTAACTGGGGAGATCAGAAAAAGTCAATGTCTTCCAAAGCAGGAGTATCTCAGGTTTTGAACAGATACACATATGCATCCACTCTGTCACATCTTCGTCGGTGTAACACTCCTCTAGGGCGCGAAGGCAAGATCGCCAAACCTCGTCAGTTGCACAACACTCATTGGGGTATGGTGTGTCCTGCGGAAACCCCTGAAGGTCAAGCCTGTGGTCTTGTCAAGAACCTCGCCCTTATGGCATGCATATCAGTCGGGTCTTACTCCGCGCCCGTTATCGAGTTTTTGGAGGAGTGGGGATTGGAGTCTTTGGAAGAGAACGCACACTCATCGACACCTTGCACAAAAGTTTTCGTGAATGGTGTCTGGATGGGTGTGCATCGCGATCCTGCCAATTTGGTGAAGACGATAAAGAAATTGAGAAGGAAAGACGACATCAGCCCAGAAGTGTCAGTTGTGCGAGACATCAGGGAGAAGGAATTGAGGTTGTACACCGATGCTGGACGTGTTTGTCGACCACTCTTCATCGTCGAGAACCAACAGCTGGCACTTCAGAAGAAGCACGTCAAATGGCTCAGTAACGGCCTCAACGATGATGGCGACGAGTACAAATGGGAACATCTGGTAAAGGGTGGCATCATTGAGTTACTGGATGCTGAGGAAGAGGAAACAGTGATGATATCCATGACCCCTGAAGATCTTGAAAATTCTCGCCTACAGCAAAGTGGTGTTGATCCTCACGCGAACGACGGCGAGTTTGATCCAGCAGCTCGATTGAAGGCTGGCACTCACGCACACACGTGGACACATTGCGAAATTCATCCTAGCATGATTCTTGGCATTTGTGCAAGCATTATTCCTTTCCCTGATCAT------------------------------------------------------------------------------------------------------------------------------------------------------------------------------------------------------------------------------------------------------------------------------------------------------------------------------------------------------------------------------------------------------------------------------------------------------------------------------------------------------------------------------------------------------------------------------------------------------------------------------------------------------------------------------------------------------------------------------------------------------------------------------------------------------------------------------------------------------------------------------------------------------------------------------------------------------------------------------------------------------------------------------------------------

>GMM7167_Laccaria_amethystina__KU685655_KU685798__KU686083

CCTGA-TGTGGCTGTTAGCTGGC-TTTTCGAAGCATGTGCTCGTCCCGTCATCTTT-ATCTCT--CCA-CCTGTGAACATTTTGTAGTC-TT-GGATACCTCTCGAGTCAA-CTCGGATT--T-TAAG-GATTGCCGTGC---TTGT--ACAAGTTGGCTTTCCTTTCACTTCC-AAGACTATGTTTT-------A-TACACA-CCCAAGTATGTTTAAAGAATGTCATC-AATAGGAAC---TTGTTTCCTA--T-AAAATTATACAACTTTCAGCAACGGATCTCTTGGCTCTCGCATCGATGAAGAACGCAGCGAAATGCGATAAGTAATGTGAATTGCAGAATTCAGTGAATCATCGAATCTTTGAACGCACCTTGCGCTCCTTGGTATTCCGAGGAGCATGCCTGTTTGAGTGTCATTAAA-TTCTCAA-CCTTCCAGC--------T-TTTATTAGCTTGG--TTAGGATTGGA-TGT-GGGAG--TTGCAGGCTTC-AT-----T---AATGAGGTCAGCTCTCCTTAAATGCATTAGCGGAACTTTTGTGGACCGTCTA-TTGGTGTGAT-AATTATCTACGCCGTGGAT-GTGAAGCAG-ATCTAT---GAAGTT-CAGCTTCT-AACCGTCC---ATTGACTTGGACAA-----TTTTGACAA-TTTGAAAGCTCAAATTTAAAATCTGGCGGT-CTTTGGCTGT-CCGAGTTGTAATCTAGAGAAGTATTATCCGCGCTGGACCGTGTACAAGTCTCCTGGAATGGGGCGTCATAGAGGGTGAGAATCCCGTCTTTGACACGGACTGCCAGGGC-TTTGTGATGTGCTCTCAAAGAGTCGAGTTGTTTGGGAATGCAGCTCAAAATGGGTGGTAAATTCCATCTAAAGCTAAATATTGGCGAGAGACCGATAGcGAACAAGTACCGTGAGGGAAAGATGAAAAGAACTTTGGAAAGAGAGTTAAACAGTACGTGAAATTGCTGAAAGGGAAACGCTTGAAGTCAGTCGCATTGGCCAGGGATCAA-CCTTGC----TTTTTT---GCTTGGCTTACTTTCTGGTCAATGGG-TCAGCATCAATTTTGACTGGTGG-AAAAAGTTCAAGGGAATGTGGCAT-CTTTGGATGTGTTATAGCCTTTGTTCACATACATTGGTCGGGATTGAGGAACTCAGCACGCCGCAAGGCCGGG---TTTTTAACCACGTACGTGCTTAGGATGCTGGCATAATGGCTTTAATCGACCCGTCTTGAAACACGGACCAAGGAGTCTAACATGCCTGCGAGTGTTTGGGTGAAAAACCCGAGCGCGTAATGAAAGTG-AAAGTTGAGATCCCTGTCATGGGGAGCATTGACGCCCGGATCTGACCTTTTGTGACGATTCTGCGGTAGAGCATGT-------------------------------------------------------------------------------------------------------------------------------------------------------------------------------------------------------------------------------------------------------------------------------------------------------------------------------------------------------------------------------------------------------------------------------------------------------------------------------------------------------------------------------------------------------------------------------------------------------------------------------------------------------------------------------------------------------------------------------------------------------------------------------------------------------------------------------------------------------------------------------------------------------------------------------------------------------------------------------------------------------------------------------------------------------------------------------------------------------------------------------------------------------------------------------------------AGCACGCTCTCCTCGCTTTCACCCTCGGTGTGCGGCAACTCATCGTTGCCGTCAACAAGATGGACACCACCAAGGTAAGAAACTTTTGAC-TTATAGCCAGGCTTGTTTCACATCCTCATCATCTCA-TGTAGTGGAGCGAGGACCGTTTCAACGAAATCATCAAGGAGACGTCAACCTTCATCAAGAAGGTTGGTTACAACCCCAAGGCCGTTGCCTTTGTCCCTATTTCCGGCTGGCACGGTGACAACATGTTGGAGGAGTCCGCTAAGTGAGCATTC--TCGCATCGTCATGGTCGCCCCGCCGCTCATGTTGTACTC-AGCATGCCTTGGTACAAGGGCTGGACCAGGGAGACCAAGGCTGGTGCCGTCAAGGGCAAGACCCTACTCGATGCTATTGATGCCATTGAGCCCCCCGTTCGACCTTCCGACAAACCCCTCCGTCTCCCCCTCCAGGATGTCTACAAAATTGGTGGTATTGGAACTGTGCCCGTCGGTCGTGTTGAGACTGGTATCATCAAGTCCGGAATGGTCGTCACTTTCGCTCCCTCCAACGTCACCACTGAAGTCAAGTCCGTCGAAATGCATCACGAACAGCTCGAGCAGGGTAACCCTGGCGACAATGTCGGTTTCAACGTCAAGAACGTGTCAGTGAAGGATATTCGTCGTGgCAACGTGGCCTCCGACTCCAAGAACGATCCTGCCAAGGAAGCGGCCTCTTTCAACGCACAGGTCATCGTCCTCAACCACCCTGGTCAAATTGGTGCTGGTTACGCCCCAGTTCTCGACTGTCACACCGCCCACATCGCCTGCAAGTTCGCCGAGCTTATCGAGAAGATCGATCGTCGTACTTTTAAATCCATTGAAAACTCACCTAAATTTGTCAAGTCGGGTGACGCCTGCATCGTCAAGCTCGTTCCCAGCAAGCCTATG

>GMM7584_Laccaria_proxima_RUSSIA_KU685717_KU685858_KU685999_KU686120

CCTGA-TGTGGCTGTTAGCTGGC-TTTTCGAAGCATGTGCTCGT-CCGTCATCTTT-ATCTCT--CCA-CCTGTGCACATTTTGTAGTC-TT-GGATAACTCTCGAGGCAA-CTCGGATT----TTAG-AATCGCCGTGC---TGT---ACAAGTCGGCTTTCCTTTCATTTCC-AAGACTATGTTTT----T--A-TATACA-CCAAAGTATGTTCAAAGAATGTCATC-AATGGGAAC---TTGTTTCCTA--T-AAAATTATACAACTTTCAGCAACGGATCTCTTGGCTCTCGCATCGATGAAGAACGCAGCGAAATGCGATAAGTAATGTGAATTGCAGAATTCAGTGAATCATCGAATCTTTGAACGCACCTTGCGCTCCTTGGTATTCCGAGGAGCATGCCTGTTTGAGTGTCATTAAA-TTCTCAA-CCTTCCAAC--------T-TTTATTAGCTTGG--TTAGGCTTGGA-TGT-GGGGG--TTGCGGGCTTC-AT-----T---AACGAGGTCGGCTCTCCTTAAATGCATTAGCGGAACTTTTGTGGACCGTCTA-TTGGTGTGAT-AATTATCTACGCCGTGGAT-GTGAAGCAG-CTTTAT---GAAGTT-CAGCTTCT-AACCGTCC---ATTTATTTGGACAA----TTTTTGACAA-TTTGAAAGCTCAAATTTAAAATCTGGCAGT-CTTTGGCTGT-CCGAGTTGTAATCTAGAGAAGTATTATCCGCGCTGGACCGTGTACAAGTCTCCTGGAATGGGGCGTCATAGAGGGTGAGAATCCCGTCTTTGACACGGACTGCCAGGGC-TTTGTGATGTGCTCTCAAAGAGTCGAGTTGTTTGGGAATGCAGCTCAAAATGGGTGGTAAATTCCATCTAAAGCTAAATATTGGCGAGAGACCGATAGCGAACAAGTACCGTGAGGGAAAGATGAAAAGAACTTTGGAAAGAGAGTTAAACAGTACGTGAAATTGCTGAAAGGGAAACGCTTGAAGTCAGTCGCGTTGGCCAGGGATCAA-CCTTGC----TTTTTT---GCTTGGCTTACTTTCTGGTCGATGGG-TCAGCATCAATTTTGACTGGTGG-AAAAAGTTCAAGGGAATGTGGCAT-CTTCGGATGTGTTATAGCCTTTGTTCGCATACATCAGTTGGGATTGAGGAACTCAGCACGCCGCAAGGCCGGG---TTTTTAACCACGTACGTGCTTAGGATGCTGGCATAATGGCTTTAATCGACCCGTCTTGAAACACGGACCAAGGAGTCTAACATGCCTGCGAGTGTTTGGGTGAAAAACCCGAGCGCGTAATGAAAGTG-AAAGTTGAGATCCCTGTCGCGGGGAGCATTGACGCCCGGATCTGACCTTTTGTGACGATTCTGCGGTAGAGCATGTTTTCCGCATGCTTTTCCGAAAGTTGACCAAAGACGTTTATCGTTATCTACAAAAGgttgctgatcttcgctcaagtgcc-----atgagatgtgctga-cctctct---ttttcagTGCGTCGAGACCCACAAGGAGTTCAACCTTGCCCTTGCAGTTAAGCATCAAACCATCACAAACGGCCTCAAATACTCTCTGGCGACAGGTAACTGGGGAGATCAAAAAAAGTCAATGTCTTCYAAGGCAGGAGTATCTCAGGTCTTGAACAGATACACATATGCATCCACTCTGTCACATCTTCGTCGGTGTAACACTCCTCTAGGGCGCGAAGGCAAGATCGCCAAACCTCGTCAGTTGCACAACACTCATTGGGGTATGGTGTGTCCTGCGGAAACTCCTGAAGGTCAAGCCTGTGGTCTTGTCAAGAACCTCGCCCTTATGGCGTGCATATCAGTCGGGTCTTACTCCGCGCCCGTTATCGARTTTTTGGAGGAATGGGGATTGGAGTCGTTGGAAGAGAACGCGCACTCATCAACACCTTGCACAAAAGTTTTCGTGAACGGTGTCTGGATGGGTGTGCATCGCGATCCTGCCAATTTGGTGAAGACGATAAAGAAATTGAGRAGGAAAGATGACATTAGCCCAGAAGTGTCAGTTGTGCGAGACATCAGGGAGAAGGAATTGAGGTTGTACACCGATGCTGGACGTGTTTGTCGACCACTCTTCATCGTCGAGAACCAACAGCTGGCACTTCAGAAGAAGCACGTCAAATGGCTCAGTAACGGCCTCAACGATGATGGTGACGAATACAAATGGGAACATCTGGTAAAGGGTGGCATCATTGAGCTACTGGATGCTGAGGAAGAGGAAACAGTGATGATATCCATGACCCCTGAAGATCTTGAAAATTCTCGCCTACAGCAAAGCGGTGTCGATCCTCACGCGAACGACGGCGAGTTTGATCCAGCAGCTCGATTGAAGGCTGGCACTCACGCACACACATGGACACATTGCGAAATTCATCCTAGCATGATTCTTGGCATTTGTGCCAGCATTATTCCTTTCCCCGATCATCTGCGCCCTTCTCATCATTGCTGGTGGTACTGGTGAGTTCGAGGCTGGTATCTCCAAGGATGGCCAGACCCGCGAGCACGCTCTCCTCGCTTTCACCCTCGGTGTGCGGCAACTCATCGTTGCCGTCAACAAGATGGACACCACCAAGGTAAGAAACCTTTGAC-TTATAGTTAGGCTTCTTGCGCATTCTCATCGTCTCA-TGTAGTGGAGCGAGGACCGTTTCAACGAAATCATCAAGGAGACGTCAACTTTCATCAAGAAGGTTGGTTACAACCCCAAGGCTGTTGCCTTTGTCCCTATTTCCGGCTGGCACGGTGACAACATGTTGGAGGAGTCTCCCAAGTAAGCATTC--TCGTATTGTCATGATCGCCCCGTCGTTCATGTTGCACTC-AGCATGCCTTGGTACAAGGGCTGGACCAAGGAGACCAAGGCYGGTGTCGTCAAGGGCRAGATCCTCATCGATRCTATCGTTGCCATTGAGCCCCCCGCCCGACCCTCCSTCCACCYTCTCGGTGTCCCCCAACATGATGTCTACAAAATTGGCGGCATCGGAACTGTGCCCGTCGGTCGTGTTGAGACTGGTATCATCAAGGCCGGAATGGTCGTCACCTTCACCACTTCCAACGTCACCATGGAAATGCAGTCCGTGGAAATGCACCACGAGCAGCTTGAGCAGGGTAACCCTGGTGACAATGTCGGTTTCAACGTCAAGAACGTGTCAGTGAAGGATATTCGTCGTGGCAACGTGGCCTATGCCTCCAAGAACGATCCCGCCAAGGAAGCCGCCTCTTTCAACGCACAGGTCATCGTCCTCAACCACCCCGGTCAAATTGGTGCTGGTTACGCCCCGGTTCTCGACTGTCACACCGCCCACATCGCCTGCAAGTTCGCCGAGCTCATTGAGAAGATCGATCGTCGTACGGGTAAATCCATTGAAAACTCGCCTAAATTCGTCAAGTCTGGTGACGCCTGCATCGTCAAGCTCGTTCCCAGCAAGCCTATG

>GMM7586_Laccaria_striatula_RUSSIA_KM067835_KU685859_KU686000_

CCTGA-TGTGACTGTTAGCTGGC-TTTTCGAAGAA-GTGCTCGT-CCGTCATCTTTAATCTCT--CCA-CCTGTGCACATTTTGTAGTC-TT-GGATACCTCTCGAGGCAA-CTCGGATT----TTAG-GATCGCCGTGC---TGTTGTACAAGTCGACTTTTCTTTCATTTCC-AAGACTATGTTTT----T--A-TATACA-CCAAAGTATGTTTAAAGAATGTCATC-AATAGGAAC---TTGTTTCCTA--T-AAAATTATACAACTTTCAGCAACGGATCTCTTGGCTCTCGCATCGATGAAGAACGCAGCGAAATGCGATAAGTAATGTGAATTGCAGAATTCAGTGAATCATCGAATCTTTGAACGCACCTTGCGCTCCTTGGTATTCCGAGGAGCATGCCTGTTTGAGTGTCATTAAA-TTCTCAA-CCTTCCATG--------T-TTTATTAGCATGG--TTAGGCTTGGA-TGT-GGGGG--TTGCGGGCTTC-AT-----C---AATGATGTCGGCTCTCCTTAAATGCATTAGCGGAACTTTTGTGGACCGTCTA-TTGGTGTGAT-AGTTATCTACGCCGTGGAT-GTGATGCAG-CTTTAT---GAAGTT-CAGCTTCT-AACCGTCC---ATTGACTTGGACAA-----TTTTGACAA--TTGAAAGCTCAAATTTAAAATCTGGCAGT-CTTTGGCTGT-CCGAGTTGTAATCTAGAGAAGTATTATCCGCGCTGGACCGTGTACAAGTCTCCTGGAATGGAGCGTCATAGAGGGTGAGAATCCCGTCTTTGACACGGACTGCCAGGGC-TTTGTGATGTGCTCTCGAAGAGTCGAGTTGTTTGGGAATGCAGCTCAAAATGGGTGGTAAATTCCATCTAAAGCTAAATATTGGCGAGAGACCGATAGCGAACAAGTACCGTGAGGGAAAGATGAAAAGAACTTTGGAAAGAGAGTTAAACAGTACGTGAAATTGCTGAAAGGGAAACGCTTGAAGTCAGTCGCGTTGGCCAGGGATCAA-CCTTGC----TTTTTT---GCTTGGCTTACTTTCTGGTCGATGGG-TCAGCATCAATTTTGACTGGTAG-AAAAAGTTCAAGGGAATGTGGCAT-CTTCGGATGTGTTATAGCCCTTGTTCACATATATCAGTTGGAATTGAGGAACTCAGCACGCCGCAAGGCCGGG---TTTTTAACCACGTACGTGCTTAGGATGCTGGCATAATGGCTTTAATCGACCCGTCTTGAAACACGGACCAAGGAGTCTAACATGCCTGCGAGTGTTTGGGTGAAAAACCCGAGCGCATAATGAAAGTG-AAAGTTGAGATCCCTGTCATGGGGAGCATTGACGCCCGGATCTGACCTTTTGTGACGATTCTGCGGTAGAGCATGTTTTCCGTATGCTTTTCCGAAAGTTGACCAAGGACGTTTATCGTTATTTACAAAAGgttgctgaccttcgctcaagtgcc-----atgagatgtgctga-cctctct---tcttcagTGCGTCGAGACCCACAAGGAGTTCAACCTTGCCCTTGCGGTTAAGCATCAAACCATCACAAACGGCCTCAAATACTCTCTGGCGACAGGTAACTGGGGAGATCAGAAAAAGTCAATGTCTTCCAAGGCAGGAGTATCTCAGGTCTTGAACAGATACACATATGCATCCACTCTGTCACATCTTCGTCGGTGTAACACTCCTCTAGGGCGCGAAGGCAAGATCGCCAAACCTCGTCAGTTGCACAACACCCATTGGGGTATGGTGTGTCCTGCGGAAACTCCTGAAGGTCAAGCCTGTGGTCTTGTCAAGAACCTCGCCCTTATGGCGTGCATATCAGTCGGGTCTTACTCCGCGCCCGTTATCGAGTTTTTGGAGGAGTGGGGATTGGAGTCATTGGAAGAGAACGCGCACTCATCAACACCTTGCACAAAAGTTTTCGTGAACGGTGTCTGGATGGGTGTGCATCGCGATCCTGCCAATTTGGTGAAGACGATAAAGAAATTGAGAAGGAAGGACGACATTAGCCCAGAAGTGTCAGTCGTGCGAGACATCAGGGAGAAGGAATTGAGGTTGTACACCGATGCTGGACGTGTTTGTCGACCACTCTTCATCGTCGAGAACCAACAGCTGGCACTTCAGAAGAAGCACGTCAAATGGCTCAGTAACGGCCTCAACGATGATGGYGACGAGTACAAATGGGAACATCTGGTAAAGGGTGGCATCATTGAGTTACTGGATGCTGAGGAAGAGGAAACAGTGATGATATCCATGACGCCTGAAGATCTTGAAAATTCTCGCCTACAGCAAAGTGGTGTTGATCCTCACGCGAACGACGGCGAGTTTGATCCAGCAGCTCGATTGAAGGCTGGCACTCACGCACACACATGGACACATTGCGAAATTCATCCTAGCATGATTCTTGGCATTTGTGCCAGCATTATTCCTTTCCCTGATCAT------------------------------------------------------------------------------------------------------------------------------------------------------------------------------------------------------------------------------------------------------------------------------------------------------------------------------------------------------------------------------------------------------------------------------------------------------------------------------------------------------------------------------------------------------------------------------------------------------------------------------------------------------------------------------------------------------------------------------------------------------------------------------------------------------------------------------------------------------------------------------------------------------------------------------------------------------------------------------------------------------------------------------------------------

>GMM7596tibet_Laccaria_salmonicola_CHINA_TIBET_JX504143_JX504218_KU686045_KU686151

CCTGA-TGTGGCTGTTAGCTGGC-TTTTCGAAGCATGTGCTCGT-CCGTCATCTTT-ATCTCT--CCA-CCTGTGCACATTTTGTAGTC--------------------------------------------------------------------------------TTTCC-AAGACTATGTTTT----T--A-TATACA-CCAATGTATGTTTAAAGAATGTCATC-AATAGAAAC---TTGTTTTCTA--TAAAAATTATACAACTTTCAGCAACGGATCTCTTGGCTCTCGCATCGATGAAGAACGCAGCGAAATGCGATAAGTAATGTGAATTGCAGAATTCAGTGAATCATCGAATCTTTGAACGCACCTTGCGCTCCTTGGTATTCCGAGGAGCATGCCTGTTTGAGTGTCATTAAA-TTCTCAA-CCTTCCAAC--------T-TTTATTAGCTTGG--TTTGGCTTGGA-TGT-GGGGG--TTGCGGGCTTCTAT-----T---AATGAAGTCGGCTCTCCTTAAATGCATTAGCGGAACTTTTGTGGACCGTCTA-TTGGTGTGAT-AATTATCTATGCCGTGGAT-GTAAAGCAG-CTTTAT---GAAGTT-CAGCTTCT-AATCGTCC---ATTGACTTGGACAATTTTTTTTTGACAA-TTTGAAAGCTCAAATTTAAAATCTGGCAGT-CTTTGGCTGT-CCGAGTTGTAATCTAGAGAAGTATTATCCGCGCTGGACCGTGTACAAGTCTCCTGGAATGGAGCGTCATAGAGGGTGAGAATCCCGTCTTTGACACGGACTGCCAGGGC-TTTGTGATATGCTCTCAAAGAGTCGAGTTGTTTGGGAATGCAGCTCAAAATGGGTGGTAAATTCCATCTAAAGCTAAATATTGGCGAGAGACCGATAGCGAACAAGTACCGTGAGGGAAAGATGAAAAGAACTTTGGAAAGAGAGTTAAACAGTACGTGAAATTGCTGAAAGGGAAACGCTTGAAGTCAGTCGCGTTGGCCAGGGATCAA-CCTTGC----TTTTTT---GCTTGGCTTACTTTCTGGTCAATGGG-TCAGCATCAATTTTGACTGGTGG-AAAAAGTTCAAGGGAATGTGGCAT-CTTCGGATGTGTTATAGCCTTTGTTCATATGCATTGGTTGGGATTGAGGAACTCAGCACGCCGAAAGGCCGGG--TTTTTTAACCACGTACGTGCTTAGGATGCTGGCATAATGGCTTTAATCGACCCGTCTTGAAACACGGACCAAGGAGTCTAACATGCCTGCGAGTGTTTGGGTGAAAAACCCGAGTGCGTAATGAAAGTG-AAAGTTGAGATCCCTGTCGTGGGGAGCATTGACGCCCGGATCTGACCTTTTGTGACGATTCTGCGGTAGAGCATGTTTTCCGCATGCTTTTCCGAAAGTTGACCAAGGACGTTTATCGTTATTTACAAAAGGTTTCTGACCTTCGCTGAAGTGCC-----ATGAGATGTGCTGA-CCCCTCT---TCTTTAGTGCGTTGAGACCCACAAGGAGTTCAACCTTGCCCTTGCAGTTAAGCATCAAACCATCACAAACGGCCTCAAATACTCTCTGGCGACAGGTAATTGGGGAGATCAGAAAAAGTCAATGTCTTCCAAGGCAGGAGTATCTCAGGTCTTGAACAGATACACATATGCGTCCACTCTGTCACATCTTCGTCGGTGTAACACTCCTCTAGGGCGCGAAGGCAAGATCGCCAAACCTCGTCAGTTGCACAACACTCATTGGGGTATGGTGTGTCCTGCGGAAACTCCTGAAGGTCAAGCCTGTGGTCTTGTCAAGAACCTCGCCCTTATGGCGTGCATATCAGTCGGGTCTTACTCTGCGCCCGTTATCGAGTTTTTGGAGGAGTGGGGGTTGGAGTCATTGGAAGAGAACGCGCACTCATCAACACCTTGCACAAAAGTTTTCGTGAACGGTGTCTGGATGGGTGTGCATCGCGATCCTGCCAATTTGGTGAAAACGATAAAGAAATTGAGAAGGAAAGACGACATCAGTCCAGAAGTGTCAGTCGTGCGAGACATCAGGGAGAAGGAATTGAGGTTGTACACAGATGCTGGACGTGTTTGTCGACCACTCTTCATCGTTGAGAACCAACAGCTAGCACTTCAGAAGAAGCACGTCAAATGGCTCAGTAACGGCCTCAACGATGATGGCGACGAGTACAAATGGGAACACCTGGTAAAAGGTGGCATCATTGAGTTACTGGATGCTGAGGAAGAGGAAACGGTGATGATATCCATGACCCCTGAAGATCTTGAAAATTCTCGCCTACAGCAAAGTGGTGTTGACCCTCATGCGAACGACGGCGAGTTTGATCCAGCAGCTCGGTTGAAGGCGGGCACTCACGCACACACGTGGACACATTGCGAAATTCATCCTAGCATGATTCTTGGCATTTGTGCCAGCATTATTCCTTTCCCTGATCAT----------------------------------------------------------------------CGCGAGCACGCTCTCCTCGCTTTCACCCTTGGTGTACGGCAACTCATCGTTGCCGTCAACAAGATGGACACCACTAAGGTAAGAAACGTTTCAC-TTATAGTTAGGCTTCTTGCACATTCTCATCATCTCA-TGTAGTGGAGCGAGGACCGTTTCAACGAAATCATCAAGGAGACGTCAACCTTCATCAAGAAGGTTGGTTACAACCCCAAGGCCGTTGCCTTTGTCCCTATTTCCGGCTGGCACGGTGACAACATGTTGGAGGAGTCCGCTAAGTAAGCATTC--TCGTATCGTCATGATCGCCCCGTCGCTCATGTTGCACTC-AGCATGTCTTGGTACAAGGGCTGGACCAAGGAGACCAAGGCTGGTGTCGTCAAGGGCAAGACCCTCCTCGATGCTATTGATGCCATTGAGCCCCCCGTCCGACCTTCCGACAAACCCCTCCGTCTCCCCCTCCAGGATGTCTACAAAATTGGCGGTATCGGAACTGTGCCCGTCGGTCGTGTTGAGACTGGTATCATCAAGGCCGGAATGGTCGTCAATTTTGCTCCCTCCAACGTCACCACTGAAGTCAAGTCCGTCGAAATGCATCACGAACAGCTTGAGCAGGGTAACCCTGGTGACAATGTCGGTTTCAACGTCAAGAACGTGTCGGTGAAGGATATTCGTCGTGGCAACGTGGCCTCCGACTCCAAGAACGATCCYGCCAAGGAAGCGGCCTCTTTCAACGCACAGGTCATCGTCCTTAAYCACCCTGGTCAAATTGGTGCTGGTTACGCCCCGGTTCTCGATTGTCACACCGCCCACATCGCCTGCAAGTTCGCCGAGCTCATCGAGAAGATCGATCGTCGTACGGGTAAATCTATTGAAAACTCGCCCAAATTCGTCAAGTCGGGTGATGCCTGCATYGTCAAGCTCGTTCCCAGCAAGCCTATG

>GMM7602Tibet_Laccaria_salmonicola_CHINA_TIBET_GMM7602_28S__

CCTGA-TGTGGCTGTTAGCTGGC-TTTTCGAAGCATGTGCTCGT-CCGTCATCTTT-ATCTCT--CCA-CCTGTGCACATTTTGTAGTC--------------------------------------------------------------------------------TTTCC-AAGACTATGTTTT----T--A-TATACC-CCAATGTATGTTTAAAGAATGTCATC-AATAGAAAC---TTGTTTTCTA--TAAAAATTATACAACTTTCAGCAACGGATCTCTTGGCTCTCGCATCGATGAAGAACGCAGCGAAATGCGATAAGTAATGTGAATTGCAGAATTCAGTGAATCATCGAATCTTTGAACGCACCTTGCGCTCCTTGGTATTYTGAGGAGCATGCCTGTTTGAGTGTCATTAAA-TTCTCAA-CCTTCCAAC--------T-TTTATTAGCTTGG--TTTGGCTTGGA-TGT-GGGGG--TTGCGGGCTTCTAT-----T---AATGAAGTCGGCTCTCCTTAAATGCATTAGCGGAACTTTTGTGGACCGTCTA-TTGGTGTGAT-AATTATCTATGCCGTGGAT-GTAAAGCAG-CTTTAT---GAAGTT-CAGCTTCT-AATCGTCC---ATTGACTTGGACAT-TTTTTTTTGACAA--TTGAAAGCTCAAATTTAAAATCTGGCAGT-CTTTGGCTGT-CCGAGTTGTAATCTAGAGAAGTATTATCCGCGCTGGACCGTGTACAAGTCTCCTGGAATGGAGCGTCATAGAGGGTGAGAATCCCGTCTTTGACACGGACTGCCAGGGC-TTTGTGATATGCTCTCAAAGAGTCGAGTTGTTTGGGAATGCAGCTCAAAATGGGTGGTAAATTCCATCTAAAGCTAAATATTGGCGAGAGACCGATAGCGAACAAGTACCGTGAGGGAAAGATGAAAAGAACTTTGGAAAGAGAGTTAAACAGTACGTGAAATTGCTGAAAGGGAAACGCTTGAAGTCAGTCGCGTTGGCCAGGGATCAA-CCTTGC----TTTTTT---GCTTGGCTTACTTTCTGGTCAATGGG-TCAGCATCAATTTTGACTGGTGG-AAAAAGTTCAAGGGAATGTGGCAT-CTTCGGATGTGTTATAGCCTTTGTTCATATGCATTGGTTGGGATTGAGGAACTCAGCACGCCGAAAGGCCGGG---TTTTTAACCACGTACGTGCTTAGGATGCTGGCATAATGGCTTTAATCGACCCGTCTTGAAACACGGACCAAGGAGTCTAACATGCCTGCGAGTGTTTGGGTGAAAAACCCGAGCGCGTAATGAAAGTG-AAAGTTGAGATCCCTGTCGTGGGGAGCATTGACGCCCGGATCTGACCTTTTGTGACGATTCTGCGGTAGAGCATGT-----------------------------------------------------------------------------------------------------------------------------------------------------------------------------------------------------------------------------------------------------------------------------------------------------------------------------------------------------------------------------------------------------------------------------------------------------------------------------------------------------------------------------------------------------------------------------------------------------------------------------------------------------------------------------------------------------------------------------------------------------------------------------------------------------------------------------------------------------------------------------------------------------------------------------------------------------------------------------------------------------------------------------------------------------------------------------------------------------------------------------------------------------------------------------------------------------------------------------------------------------------------------------------------------------------------------------------------------------------------------------------------------------------------------------------------------------------------------------------------------------------------------------------------------------------------------------------------------------------------------------------------------------------------------------------------------------------------------------------------------------------------------------------------------------------------------------------------------------------------------------------------------------------------------------------------------------------------------------------------------------------------------------------------------------------------------------------------------------------------------------------------------------------------

>PRL7587_Laccaria_trullisata__KM067882_JX504247_KU686047_KU686153

CCTGA-TGTGACTGTTAGCTGGC-TTTTTGAAGCATGTGCTCGT-CCGTCATTTTT-ATCTCT--CCACCCTGTGCACATTTTGTAGTC-TT-GGATACCTCTCGAGGCAA-CTCGGATT----TTGGAGATCGCTGTGC---TGT---ACAAGTCAGCTTTCTTTTCATTTCC-AAGACTATGTTTTAATAT--A-TACACA-CCAAAGTATGTTTAAAGAATGTCATC-AATAGGAAC---TTGTTTCCTA--TAAAATTTATACAACTTTCAGCAACGGATCTCTTGGCTCTCGCATCGATGAAGAACGCAGCGAAATGCGATAAGTAATGTGAATTGCAGAATTCAGTGAATCATCGAATCTTTGAACGCACCTTGCGCTCCTTGGTATTCCGAGGAGCATGCCTGTTTGAGTGTCATTAAA-TTCTCAA-CCTTCCAGC--------TTTTTATTGGCTTGG--TTAGGCTTGGA-TGT-GGGGG-TTTGTGGGCTTC-AT-----TTA-ATTGAGGTCGACTCTCCTTAAATGCATTAGTGGAACTTTTGTGGACCGTCTA-TTGGTGTGAT-AATTATCTACGCCGTGGAT-GTGAAGCATAATTTAT---GAAGTT-CAGCTTCT-AACCGTCC---ATTGACTTGGACAA-----TTTTGACAA-TTTGAAAGCTCAAATTTAAAATCTGGCAGT-CTTTGGCTGT-CCGAGTTGTAATCTAGAGAAGTATTATCCGCGCTGGACCGTGTACAAGTCTCCTGGAATGGGGCGTCATAGAGGGTGAGAATCCCGTCTTTGACACGGACTGCCAGGGCTTT-GTGATGTGCTCTCAAAGAGTCGAGTTGTTTGGGAATGCAGCTCAAAATGGGTGGTAAATTCCATCTAAAGCTAAATATTGGCGAGAGACCGATAGCGAACAAGTACCGTGAGGGAAAGATGAAAAGAACTTTGGAAAGAGAGTTAAACAGTACGTGAAATTGCTGAAAGGGAAACGCTTGAAGTCAGTCGCGTTGGCCAGGGATCAA-CCTTGC----TTTTTT---GCTTGGCTTACTTTCTGGTCGATGGGGTCAGCATCAATTTTGACTGGTGG-AAAAAGTTCAAGGGAATGTGGCAT-CTTCGGATGTGTTATAGCCTTTGTTCGCATACATTGGTTGGGATTGAGGAACTCAGCACGCCGCAAGGCCGGG---TTTTTAACCACGTACGTGCTTAGGATGCTGGCATAATGGCTTTAATCGACCCGTCTTGAAACACGGACCAAGGAGTCTAACATGCCTGCGAGTGTTTGGGTGAAAAACCCGAGTGCGTAATGAAAGTG-AAAGTTGAGATCCCTGTCGTGGGGAGCATCGACGCCCGGATCTGACCTTTTGTGACGATTCTGCGGTAGAGCATGTTTTCCGTATGCTTTTCCGAAAGTTGAGCAAGGACGTTTATCGTTATCTACAAAAGgttgttgacctttg-tcaagtgcc-----atgagatgtgctga-tccctct---tcttcagTGCGTCGAGACCCACAAGGAGTTCAACCTTGCCCTTGCAGTTAAGCATCAAACCATCACAAACGGCCTCAAATACTCTCTGGCGACAGGTAACTGGGGAGATCAGAAAAAGTCAATGTCTTCCAAGGCAGGAGTATCTCAGGTCTTGAACAGATACACATATGCATCCACTCTGTCACATCTTCGTCGGTGTAACACTCCTCTAGGGCGCGAAGGCAAAATCGCCAAACCTCGTCAGTTGCACAACACTCATTGGGGTATGGTGTGTCCTGCGGAAACTCCTGAAGGTCAAGCTTGTGGTCTTGTCAAGAACCTTGCCCTTATGGCGTGCATATCAGTCGGGTCTTACTCCGCGCCCGTTATCGAGTTTTTGGAGGAGTGGGGATTGGAGTCATTGGAAGAGAACGCACACTCATCGACACCTTGCACAAAAGTTTTCGTGAACGGTGTTTGGATGGGTGTGCATCGCGATCCTGCCAATTTGGTGAAGACGATAAAGAAATTGAGAAGGAAAGATGACATCAGCCCAGAAGTGTCAGTTGTGCGAGACATCAGGGAGAAGGAATTGAGGTTGTACACCGATGCTGGACGTGTTTGTCGACCACTCTTCATCGTCGAGAACCAACAGCTGGCACTTCAGAAGAAGCACGTCAAATGGCTCAGTAACGGCCTCAACGATGATGGCGACGAGTACAAATGGGAACATCTGGTAAAGGGTGGCATCATCGAGCTACTGGATGCTGAGGAAGAGGAAACGGTGATGATATCTATGACCCCTGAAGATCTTGAAAATTCTCGCCTACAGCAAAGTGGTGTTGATCCTCATGCGAACGACGGCGAGTTTGATCCAGCAGCTCGATTGAAGGCTGGCACTCATGCACACACATGGACACATTGCGAAATTCATCCTAGCATGATTCTTGGCATTTGCGCCAGCATTATTCCTTTCCCTGATCATTTGCGCCATTCTCATCATTGCTGCTGGTACTGGTGAGTTCGAGGCTGGTATCTCCAAGGATGGCCAGACCCGCGAGCACGCTCTCCTGGCTTTCACCCTCGGTGTGCGGCAACTCATCGTTGCCGTCAACAAGATGGACACCACTAAGGTAAGAAACGTTTCAC-TTATAGTTAGGCTTCTTGCACATTCTCATCATCTCA-TGTAGTGGAGCGAGGACCGTTTCAACGAAATCATCAAGGAGACGTCAACCTTCATCAAGAAGGTTGGTTACAACCCCAAGGCCGTTGCCTTTGTCCCTATTTCCGGCTGGCACGGTGACAACATGTTGGAGGAGTCCCCTAAGTAAGCATTC--TCGTATCGTCATGATCGCCCCGTCGCTCATGTTGCACTCAAGCATGTCTTGGTACAAGGGCTGGACCAAGGAGACCAAGGCTGGTGTCGTCAAGGGCAAGACCCTCCTCGATGCTATTGATGCCATTGAGCCCCCCGTCCGACCTTCCGACAAACCCCTCCGTCTYCCTCTTCAGGATGTCTACAAAATTGGCGGTATCGGAACTGTGCCCGTCGGTCGTGTTGAGACTGGCATCATCAAGGCCGGAATGGTCGTCAATTTCGCTCCCTCCAACGTCACGACTGAAGTCAAGTCCGTCGAAATGCATCACGAACAGCTCGAGCAGGGTAACCCTGGTGACAATGTCGGTTTCAACGTCAAGAACGTGTCGGTGAAGGATATTCGTCGTGGCAACGTGGCCTCCGACTCCAAGAACGATCCCGCCAAGGAAGCGGCCTCTTTCAACGCACAGGTCATCGTCCTCAACCACCCTGGTCAAATTGGTGCTGGTTACGCTCCGGTTCTCGACTGTCACACTGCCCACATCGCCTGCAAGTTCGCCGAGCTCATTGAGAAGATCGATCGTCGTACGGGTAAATCCATTGAAAACTCGCCTAAATTCGTCAAGTCGGGTGACGCCTGCATCGTCAAGCTCGTTCCCAGCAAGCCTATG

>WCG2072_Laccaria_trullisata__KU685749__KU686044_KU686150

CCTGA-TGTGACTGTTAGCTGGC-TTTTTGAAGCATGTGCTCGT-CCGTCATTTTT-ATCTCT--CCACCCTGTGCACATTTTGTAGTC-TT-GGATACCTCTCGAGGCAA-CTCGGATT----TTGGAGATCGCTGTGC---TGT---ACAAGTCAGCTTTCTTTTCATTTCC-AAGACTATGTTTTAATAT--A-TACACA-CCAAAGTATGTTTAAAGAATGTCATC-AATAGGAAC---TTGTTTCCTA--TAAAATTTATACAACTTTCAGCAACGGATCTCTTGGCTCTCGCATCGATGAAGAACGCAGCGAAATGCGATAAGTAATGTGAATTGCAGAATTCAGTGAATCATCGAATCTTTGAACGCACCTTGCGCTCCTTGGTATTCCGAGGAGCATGCCTGTTTGAGTGTCATTAAA-TTCTCAA-CCTTCCAGC--------TTTTTATTGGCTTGG--TTAGGCTTGGA-TGT-GGGGG-TTTGTGGGCTTC-AT-----TTA-ATTGAGGTCGACTCTCCTTAAATGCATTAGTGGAACTTTTGTGGACCGTCTA-TTGGTGTGAT-AATTATCTACGCCGTGGAT-GTGAAGCATAATATAT---GAAGTT-CAGCTTCT-AACCGTCC---ATTGACTTGGACAA-----TTTTGACAA-TTTGA--------------------------------------------------------------------------------------------------------------------------------------------------------------------------------------------------------------------------------------------------------------------------------------------------------------------------------------------------------------------------------------------------------------------------------------------------------------------------------------------------------------------------------------------------------------------------------------------------------------------------------------------------------------------------------------------------------------------------------------------------------------TTTCCGTATGCTTTTCCGAAAGTTGAGCAAGGACGTTTATCGTTATCTACAAAAGgttgttgacctttg-tcaagtgcc-----atgagatgtgctga-tccctct---tcttcagTGCGTCGAGACCCACAAGGAGTTCAACCTTGCCCTTGCAGTTAAGCATCAAACCATCACAAACGGCCTCAAATACTCTCTGGCGACAGGTAACTGGGGAGATCAGAAAAAGTCAATGTCTTCCAAGGCAGGAGTATCTCAGGTCTTGAACAGATACACATATGCATCCACTCTGTCACATCTTCGTCGGTGTAACACTCCTCTAGGGCGCGAAGGCAAAATCGCCAAACCTCGTCAGTTGCACAACACTCATTGGGGTATGGTGTGTCCTGCGGAAACTCCTGAAGGTCAAGCTTGTGGTCTTGTCAAGAACCTTGCCCTTATGGCGTGCATATCAGTCGGGTCTTACTCCGCGCCCGTTATCGAGTTTTTGGAGGAGTGGGGATTGGAGTCATTGGAAGAGAACGCACACTCATCGACACCTTGCACAAAAGTTTTCGTGAACGGTGTTTGGATGGGTGTGCATCGCGATCCTGCCAATTTGGTGAAGACGATAAAGAAATTGAGAAGGAAAGATGACATCAGCCCAGAAGTGTCAGTTGTGCGAGACATCAGGGAGAAGGAATTGAGGTTGTACACCGATGCTGGACGTGTTTGTCGACCACTCTTCATCGTCGAGAACCAACAGCTGGCACTTCAGAAGAAGCACGTCAAATGGCTCAGTAACGGCCTCAACGATGATGGCGACGAGTACAAATGGGAACATCTGGTAAAGGGTGGCATCATCGAGCTACTGGATGCTGAGGAAGAGGAAACGGTGATGATATCTATGACCCCTGAAGATCTTGAAAATTCTCGCCTACAGCAAAGTGGTGTTGATCCTCATGCGAACGACGGCGAGTTTGATCCAGCAGCTCGATTGAAGGCTGGCACTCATGCACACACATGGACACATTGCGAAATTCATCCTAGCATGATTCTTGGCATTTGCGCCAGCATTATTCCTTTCCCTGATCATCTGCGCCATTCTCATCATTGCTGGTGGTACTGGTGAGTTCGAGGCTGGTATCTCCAAGGATGGCCAGACCCGCGAGCACGCTCTCCTCGCTTTCACCCTCGGTGTACGGCAACTCATCGTTGCCGTCAACAAGATGGACACCACCAAGGTAAGAAAC-TTTTAC-TTATAGTTAGGCTTCTTTCACATCCTCATCATCTCA-TGTAGTGGAGCGAGGACCGTTTCAACGAAATCGTCAAGGAGACGTCAACCTTCATCAAGAAGGTTGGTTACAACCCCAAGGCCGTTGCCTTTGTCCCTATTTCCGGCTGGCACGGTGACAACATGTTGGAGGAGTCCGCTAAGTGAGTATTC--TCGCATCGTCGTGGTCGCCTCGTCGCTCATGTTGTACTC-AGCATGCCTTGGTACAAGGGCTGGACTAAGGAGACCAAGGCTGGTGTCGTCAAGGGCAAGACCCTCCTCGATGCTATTGATGCCATTGAGCCCCCCGTTCGACCTTCCGACAAACCTCTCCGTCTCCCCCTCCAGGATGTCTACAAAATTGGTGGTATTGGGACTGTGCCCGTCGGTCGTGTTGAGACTGGTATCATCAAGGCCGGAATGGTCGTCACTTTCGCTCCCTCCAACGTCACCACCGAAGTCAAGTCCGTCGAAATGCATCACGAACAGCTCGAGCAGGGTAACCCTGGTGACAATGTCGGTTTCAACGTCAAAAACGTGTCAGTGAAGGATATTCGTCGTGGCAACGTGGCCTCCGATTCCAAGAACGATCCAGCCAAGGAAGCGGCCTCTTTCAACGCACAGGTCATCGTCCTCAACCACCCTGGTCAAATTGGTGCTGGTTACGCCCCAGTGCTCGACTGTCACACCGCCCACATCGCCTGCAAGTTCGCCGAGCTCATCGAGAAGATCGATCGTCGTACGGGTAAATCCATTGAAAACTCACCTAAATTCGTCAAGTCGGGTGACGCCTGCATTGTCAAGCTCATTCCCAGCAAGCCTATG

>SB2151_Laccaria_laccata_PORTUGAL_KM067888_28S_RPB2_EF1a

CCTGA-TGTGGCTGTTAGCTGGCTTTTTTGAAGCATGTGCTCGT-CCGTCATTTTT-ATCT----CCA-CCTGTGCACATTTTGTAGTC-TT-GGATACCTCTCGAGGCAA-CTCGGATT----TTAG-GATTGCCGTGC---TGT---TCAAGTCGGCTTTCCTTTCATTTCC-AAGACTATGTTTT----T--A-TATACA-CCAAAGCATGTTTATAGAATGTCATC-AATAGGAAC---TTGTTTCCTA--T-AAAATTATACAACTTTCAGCAACGGATCTCTTGGCTCTCGCATCGATGAAGAACGCAGCGAAATGCGATAAGTAATGTGAATTGCAGAATTCAGTGAATCATCGAATCTTTGAACGCACCTTGCGCTCCTTGGTATTCCGAGGAGCATGCCTGTTTGAGTGTCATTAAA-TTCTCAA-CCTTCCAGC--------T-TTTATTGGCTTGG--TTAGGCTTGGATTGT-GGGGG--TTGCGGGCTTC-AT-----T---AATGAGGTCGGCTCTCCTTAAATGCATTAGCGGAACTTTTGTGGACCGTCTA-TTGGTGTGAT-AATTATCTACGCCGTGGAT-GTGAAGCAG-CTTTAT---GAAGTT-CAGCTTCT-AATCGTTC---ATTGACTTGGACAA-----TTTTGACAA-TTTGAAAGCTCAAATTTAAAATCTGGCAGT-CTTTGGCTGT-CCGAGTTGTAATCTAGAGAAGTATTATCCGCGCTGGACCGTGTACAAGTCTCCTGAAATGGGGCATCATAGAGGGTGAGAATCCCGTCTTTGACACGGACTGCCAGGGC-TTTGTGATGTGCTCTCAAAGAGTCGAGTTGTTTGGGAATGCAGCTCAAAATGGGTGGTAAATTCCATCTAAAGCTAAATATTGGCGAGAGACCGATAGCGAACAAGTACCGTGAGGGAAAGATGAAAAGAACTTTGGAAAGAGAGTTAAACAGTACGTGAAATTGCTGAAAGGGAAACGCTTGAAGTCAGTCGCGTTGGCCAGGGATCAA-CCTTGC-TTTTTTTTT---GCTTGGCTTACTTTCTGGTCGACGGG-TCAGCATCAATTTTGACCGGTGG-AAAAAGTTCAAGGGAATGTGGCAT-CTTCGGATGTGTTATAGCCTTTGTTCGCATACATCGGTTGGGATTGAGGAACTCAGCACGCCGAAAGGCCGGG---TTTTTAACCACGTACGTGCTTAGGATGCTGGCATAATGGCTTTAATCGACCCGTCTTGAAACACGGACCAAGGAGTCTAACATGCCTGCGAGTGTTTGGGTGAAAAACCCGAGCGCGTAATGAAAGTG-AAAGTTGAGATCCCTGTCATGGGGAGCATCGACGCCCGGATCTGACCTTTTGTGACGATTCTGCGGTAGAGCATGTTTTCCGCATGCTTTTCCGAAAGTTGACCAAGGACGTTTATCGTTATCTACAAAAGgttgctgaccttcgctcaagtgcc-----atgagatgtgctga-cccctct---tcttcagTGCGTCGAGACCCACAAGGAGTTCAACCTTGCCCTTGCAGTTAAGCATCAAACCATCACAAACGGCCTCAAATACTCTCTAGCGACAGGTAACTGGGGAGATCAGAAAAAATCAATGTCTTCCAAGGCAGGAGTATCTCAGGTCTTGAACAGATACACATATGCATCTACTCTGTCACATCTTCGTCGGTGTAACACTCCTCTAGGGCGCGAAGGCAAGATAGCCAAACCCCGTCAGTTGCACAACACTCATTGGGGTATGGTGTGTCCTGCGGAAACTCCTGAAGGTCAAGCCTGTGGTCTTGTCAAGAACCTCGCCCTTATGGCGTGCATATCAGTGGGGTCTTACTCCGCGCCCGTTATCGAGTTTTTAGAGGAGTGGGGATTGGAGTCATTGGAAGAGAACGCACACTCATCGACACCTTGCACAAAAGTTTTCGTGAACGGTGTCTGGATGGGTGTGCATCGCGATCCTGCCAACTTGGTGAAAACGATAAAGAAGCTGAGAAGGAAAGACGACATCAGCCCAGAAGTGTCAGTTGTGCGAGACATCAGGGAGAAGGAATTGAGGTTGTACACTGATGCTGGACGTGTTTGTCGACCACTCTTCATCGTCGAGAACCAACAGCTGGCACTTCAGAAGAAGCACGTCAAATGGCTCAGTAACGGCCTCAACGATGATGGCGACGAGTACAAATGGGAACATCTGGTAAAGGGTGGCATCATTGAGTTACTGGATGCTGAGGAAGAGGAAACKGTGATGATCTCCATGACCCCTGAAGATCTTGAAAATTCTCGCCTACAGCAAAGTGGTGTTGATCCTCACGCGAACGATGGCGAGTTTGATCCAGCAGCTCGATTGAAGGCTGGCACTCACGCACACACATGGACACATTGTGAAATTCATCCTAGCATGATTCTCGGCATTTGCGCCAGCATTATTCCTTTCCCTGATCATCTGCGCCATTCTCATCATTGCTGGTGGTACTGGTGAGTTCGAGGCTGGTATCTCCAAGGATGGCCAGACCCGCGAGCACGCTCTCCTCGCTTTCACCCTCGGTGTGCGGCAACTCATCGTTGCCGTCAACAAGATGGACACGACTAAGGTAAGAAACATTTCAA-TTATAGTTAGGCTTCTTGCACATTCTCATCATCYCA-TGTAGTGGAGCGAGGACCGTTTCAACGAAATCATCAAGGAGACGTCAACCTTCATCAAGAAGGTTGGTTACAACCCCAAGGCCGTTGCATTTGTCCCTATTTCCGGTTGGCACGGTGACAACATGTTGGAGGAGTCCCCTAAGTAAGCATTC--TCGTGTCGTCATGATCGCCCCGTCGCTCAYGTCGCACTC-AGCATGCCTTGGTACAAGGGCTGGACCAAGGAGACCAAGGCTGGTGTCGTCAAGGGCAAGACCCTCCTCGATGCTATTGATGCCATTGAGCCCCCCGTCCGACCTTCCGATAAACCCCTCCGTCTCCCCCTCCAGGATGTCTACAAAATTGGCGGTATCGGAACTGTGCCCGTCGGTCGTGTTGAGACTGGTATCATCAAGGCCGGAATGGTCGTCACTTTCGCTCCCTCTAACGTCACCACTGAAGTCAAGTCCGTCGAAATGCATCACGAACAGCTCGAGCAGGGTAACCCAGGTGACAATGTCGGTTTCAACGTCAAGAACGTGTCAGTGAAGGATATTCGTCGTGGCAACGTGGCCTCCGACTCCAAGAATGATCCCGCCAAGGAAGCGGCCTCTTTCAAYGCACAGGTCATCGTCCTCAACCACCCTGGTCAAATTGGTGCTGGTTACGCCCCGGTTCTCGACTGCCACACCGCCCACATCGCCTGCAAGTTCGCCGAGCTCATCGAGAAGATCGATCGTCGTACGGGTAAATCCATTGAAAACTCGCCKAAATTCGTCAAGTCGGGTGACGCCTGCATCGTCAAGCTCGTTCCCAGCAAGCCTATG

>GMM7038_Laccaria_proxima_RUSSIA_GMM7038_GMM7038_RPB2_GMM7038

CCTGA-TGTGGCTGTTAGCTGGCTTTTTTGAAGCATGTGCTCGT-CCGTCATTTTT-ATCT----CCA-CCTGTGCACATTTTGTAGTC-TT-TGATACCTCTCGAGGCAA-CTCGGATT----TTAG-GATTGCCGTGC---TGT---TCAAGTCGGCTTTCCTTTCATTTCC-AAGACTATGTTTT----T--A-TATACA-CCAAAGCATGTTTATAGAATGTCATC-AATAGGAAC---TTGTTTCCTA--T-AAAATTATACAACTTTCAGCAACGGATCTCTTGGCTCTCGCATCGATGAAGAACGCAGCGAAATGCGATAAGTAATGTGAATTGCAGAATTCAGTGAATCATCGAATCTTTGAACGCACCTTGCGCTCCTTGGTATTCCGAGGAGCATGCCTGTTTGAGTGTCATTAAA-TTCTCAA-CCTTCCAGC--------T-TTTATTGGCTTGG--TTAGGCTTGGATTGT-GGGGG--TTGCGGGCTTC-AT-----T---AATGAGGTCGGCTCTCCTTAAATGCATTAGCGGAACTTTTGTGGACCGTCTA-TTGGTGTGAT-AATTATCTACGCCGTGGAT-GTGAAGCAG-CTTTAT---GAAGTT-CAGCTTCT-AATCGTTC---ATTGACTTGGACAA-----TTTTGACAA-TTTGAAAGCTCAAATTTAAAATCTGGCAGT-CTTTGGCTGT-CCGAGTTGTAATCTAGAGAAGTATTATCCGCGCTGGACCGTGTACAAGTCTCCTGAAATGGGGCATCATAGAGGGTGAGAATCCCGTCTTTGACACGGACTGCCAGGGC-TTTGTGATGTGCTCTCAAAGAGTCGAGTTGTTTGGGAATGCAGCTCAAAATGGGTGGTAAATTCCATCTAAAGCTAAATATTGGCGAGAGACCGATAGCGAACAAGTACCGTGAGGGAAAGATGAAAAGAACTTTGGAAAGAGAGTTAAACAGTACGTGAAATTGCTGAAAGGGAAACGCTTGAAGTCAGTCGCGTTGGCCAGGGATCAA-CCTTGC----TTTTTT---GCTTGGCTTACTTTCTGGTCGACGGG-TCAGCATCAATTTTGACCGGTGG-AAAAAGTTCAAGGGAATGTGGCAT-CTTCGGATGTGTTATAGCCTTTGTTCGCATACATCGGTTGGGATTGAGGAACTCAGCATGCCGAAAGGCCGGG---TTTTTAACCACGTACGTGCTTAGGATGCTGGCATAATGGCTTTAATCGACCCGTCTTGAAACACGGACCAAGGAGTCTAACATGCCTGCGAGTGTTTGGGTGAAAAACCCGAGCGCGTAATGAAAGTG-AAAGTTGAGATCCCTGTCATGGGGAGCATCGACGCCCGGATCTGACCTTTTGTGACGATTCTGCGGTAGAGCATGTTTTCCGCATGCTTTTCCGAAAGTTGACCAAGGACGTTTATCGTTATCTACAAAAGGTTGCTGACCTTCGCTCAAGTGCC-----ATGAGATGTGCTGA-CCCCTCT---TCTTCAGTGCGTCGAGACCCACAAGGAGTTCAACCTTGCCCTTGCAGTTAAGCATCAAACCATCACAAACGGCCTCAAATACTCTCTAGCGACAGGTAACTGGGGAGACCAGAAAAAATCAATGTCTTCCAAGGCAGGAGTATCTCAGGTCTTGAACAGATACACATATGCATCTACTCTGTCACATCTTCGTCGGTGTAACACTCCTCTAGGGCGCGAAGGCAAGATAGCCAAACCCCGTCAGTTGCACAACACTCATTGGGGTATGGTGTGTCCTGCGGAAACTCCTGAAGGTCAAGCCTGTGGTCTTGTCAAGAACCTCGCCCTTATGGCGTGCATATCAGTGGGGTCTTACTCCGCGCCCGTTATCGAGTTTTTAGAGGAGTGGGGATTGGAGTCATTGGAAGAGAACGCACACTCATCGACACCTTGCACAAAAGTTTTCGTGAACGGTGTCTGGATGGGTGTGCATCGCGATCCTGCCAACTTGGTGAAAACGATAAAGAAGCTGAGAAGGAAAGACGACATCAGCCCAGAAGTGTCAGTTGTGCGAGACATCAGGGAGAAGGAATTGAGGTTGTACACTGATGCTGGACGTGTTTGTCGACCACTCTTCATCGTCGAGAACCAACAGCTGGCACTTCAGAAGAAGCACGTCAAATGGCTCAGCAACGGCCTCAACGATGATGGCGACGAGTACAAATGGGAACATCTGGTAAAGGGTGGCATCATTGAGTTACTGGATGCTGAGGAAGAGGAAACGGTGATGATCTCCATGACCCCTGAAGATCTTGAAAATTCTCGCCTACAGCAAAGTGGTGTTGATCCTCACGCGAACGATGGCGAGTTTGATCCAGCAGCTCGATTGAAGGCTGGCACTCACGCACACACATGGACACATTGTGAAATTCATCCTAGCATGATTCTCGGCATTTGCGCCAGCATTATTCCTTTCCCTGATCAT----------------------------------------------------------------------CGCGAGCACGCTCTCCTCGCTTTCACCCTCGgTGTGCGGCAACTCATCGTTGCCGTCAaCAAGATGGACACGACTAAGGTAAGAAACATTTCAA-TTATAGTTAGGCTTCTTGCACATTCTCATCATCTCA-TGTAGTGGAGTGAGGACCGTTTCAACGAAATCATCAAGGAGACGTCAACCTTCATCAAGAAGGTTGGTTACAACCCCAAGGCCGTTGCCTTTGTCCCTATTTCCGGCTGGCACGGTGACAACATGTTGGAGGAGTCCCCTAAGTAAGCATTC--TCGTGTCGTCATGATCGCCCCGTCGCTCATGTCGCACTC-AGCATGCCTTGGTACAAGGGCTGGACCAAGGAGACCAAGGCTGGTGTCGTCAAGGGCAAGACCCTCCTCGATGCTATTGATGCCATTGAGCCCCCCGTCCGACCTTCCGACAAACCCCTCCGTCTCCCCCTCCAGGATGTCTACAAAATTGGCGGTATCGGAACTGTGCCCGTCGGTCGTGTTGAGACTGGTATCATCAAGGCCGGAATGGTCGTTACTTTCGCTCCCTCTAACGTCACCACTGAAGTCAAGTCCGTCGAAATGCATCACGAACAGCTCGAGCAGGGTAACCCTGGTGACAATGTCGGTTTCAACGTCAAGAACGTGTCAGTGAAAGATATTCGTCGTGGCAACGTGGCCTCCGACTCCAAGAATGATCCCGCCAAGGAAGCGGCCTCTTTCAACGCACAGGTCATCGTCCTCAACCACCCTGGTCAAATTGGTGCTGGTTACGCCCCGGTTCTCGACTGTCACACCGCCCACATCGCCTGCAAGTTCGCCGAGCTCATCGAGAAGATCGATCGTCGTACGGGTAAATCCATTGAAAaCTCGCCTAAATTCGTCAaGTCGGgTGACGCCTGCATCGTCAaGCTCGTtCCCAGCAaGCCTATG

>SB2135_Laccaria_laccata_PORTUGAL_JX504172_JX504249_KU686028_KU686140

CCTGA-TGTGGCTGTTAGCTGGC-TTTTCGAAGCATGTGCTCGT-CCATCATCTTT-ATCT----CCA-CCTGTGCACATTTTGTAGTC-TT-GGATACCTCTCGAGGCAA-CTCGGATT----TTAG-GATCGCTGTGC---TGT---ACAAGTCGGCTTTCCTTTCATTTCC-AAGACTATGTTTT---TT--A-TATACA-CCAAAGTATGTTTAAAGAATGTCATC-AATGGGA-------------TA--T-AAAATTATACAACTTTCAGCAATGGATCTCTTGGCTCTCGCATCGATGAAGAACGCAGCGAAATGCGATAAGTAATGTGAATTGCAGAATTCAGTGAATCATCGAATCTTTGAACGCACCTTGCGCTCCTTGGTATTCCGAGGAGCATGCCTGTTTGAGTGTCATTAAA-TTCTCAA-CCTTCCAGC--------T-TTTATTAGCTTGG--ATAGGCTTGGA-TGT-GGGGG--TTGCGGGCTTC-AT-----C---AATGAGGTTGGCTCTCCTTAAAAGCATTAGCGGAACTTTTGTGGACCGTCTA-TTGGTGTGAT-AATTATCTACGCCGTGGAT-GTGAAGCAA-CTTTAT---AAAGTT-CAGCTTCT-AACTGTCC---ATTAATTTGGACAA-----TTTTGACAA-TTTGGAAGCTCAAATTTAAAATCTGGCAGT-ATTTGGCTGT-CCGAGTTGTAATCTAGAGAAGTATTATCCGCGCTGGACCGTGTACAAGTCTCCTGGAATGGAGCGTCATAGAGGGTGAGAATCCCGTCTTTGACACGGACTGCCAGGGC-TTTGTGATGTGCTCTCAAAGAGTCGAGTTGTTTGGGAATGCAGCTCAAAATGGGTGGTAAATTCCATCTAAAGCTAAATATTGGCGAGAGACCGATAGCGAACAAGTACCGTGAGGGAAAGATGAAAAGAACTTTGGAAAGAGAGTTAAACAGTACGTGAAATTGCTGAAAGGGAAACGCTTGAAGTCAGTCGCGTTGGCCAGGGATCAA-CCTTGC----TTTTTT---GCTTGGCTTACTTTCTGGTTGATGGG-TCAGCATCAATTTTGACTGGTGG-AAAAAGTTCAAGGGAATGTGGCAT-CTTCTGATGTGTTATAGCCTTTGTTCGCATACATCGGTTGGGATTGAGGAACTCAGCACGCCGCAAGGCCGGG---TTTTTAACCACGTACGTGCTTAGGATGCTGGCATAATGGCTTTAATCGACCCGTCTTGAAACACGGACCAAGGAGTCTAACATGCCTGCGAGTGTTTGGGTGAAAAACTCGAGCGCGTAATGAAAGTG-AAAGTTGAGATCCCTGTCATGGGGAGCATTGACGCCCGGATCTGACCTTTTGTGACGATTCTGCGGTAGAGCATGTTTTCCGCATGCTTTTCCGAAAGTTGACCAAGGACGTTTATCGTTATCTACAAAAGgttgctgaccttcgttcaagtgcc-----atgagatctgctga-cccttct---ttttcagTGCGTCGAGGCCCACAAGGAGTTCAACCTTGCCCTTGCAGTTAAGCATCAAACCATCACAAACGGCCTCAAATACTCTCTGGCGACAGGTAACTGGGGAGATCAGAAAAAGTCAATGTCTTCCAAAGCAGGAGTATCTCAGGTTTTGAACAGATACACATATGCATCCACTCTGTCACATCTTCGTCGGTGTAACACTCCTCTAGGGCGCGAAGGCAAGATCGCCAAACCTCGTCAGTTGCACAACACTCATTGGGGTATGGTGTGTCCTGCGGAAACCCCTGAAGGTCAAGCCTGTGGTCTTGTCAAGAACCTCGCCCTTATGGCATGCATATCAGTCGGGTCTTACTCCGCGCCCGTTATCGAGTTTTTGGAGGAGTGGGGATTGGAGTCTTTGGAAGAGAACGCACACTCATCGACACCTTGCACAAAAGTTTTCGTGAATGGTGTCTGGATGGGTGTGCATCGCGATCCTGCCAATTTGGTGAAGACGATAAAGAAATTGAGAAGGAAAGACGACATCAGCCCAGAAGTGTCAGTTGTGCGAGACATCAGGGAGAAGGAATTGAGGTTGTACACTGATGCTGGACGTGTTTGTCGACCACTCTTCATCGTCGAGAACCAACAGCTGGCACTTCAGAAGAAGCACGTCAAATGGCTCAGTAACGGCCTCAACGATGATGGCGACGAGTACAAATGGGAACATCTGGTAAAGGGTGGCATCATTGAGTTACTGGATGCTGAGGAAGAGGAAACAGTGATGATATCCATGACCCCTGAAGATCTTGAAAATTCTCGCCTACAGCAAAGTGGTGTTGATCCTCACGCGAACGACGGCGAGTTTGATCCAGCAGCTCGATTGAAGGCTGGCACTCACGCACACACGTGGACACATTGCGAAATTCATCCTAGCATGATTCTTGGCATTTGTGCAAGCATTATTCCTTTCCCTGATCATCTGCGCCATTCTCATCATTGCTGGTGGTACTGGTGAGTTCGAGGCTGGTATATCTAAGGATGGCCAGACCCGCGAACACGCTCTCCTCGCTTTCACCCTTGGTGTGCGGCAACTCATCGTTGCCGTCAACAAGATGGACACCACTAAGGTAAGAAACGTTTCAC-CTATAGTTAGGCTCCTTCCAGATTCTCATCATCTCA-TGTAGTGGAGCGAGGACCGTTTCAACGAAATCATCAAGGAGACGTCAACCTTCATCAAGAAGGTTGGTTACAACCCCAAGGCCGTTGCCTTTGTCCCTATTTCCGGCTGGCACGGTGACAACATGTTGGAGGAGTCCTCTAAGTAAGCATTC--TCGTATTGTCATGATTGCCCCGTCACTCATGTTGCACCC-AGCATGCCTTGGTACAAGGGCTGGACCAAGGAGACCAAGGCTGGTGTCGTCAAGGGCAAGACCCTCCTCGATGCTATTGATGCCATTGAACCCCCCGTCCGACCTTCCGACAAACCCCTCCGTCTCCCCCTCCAGGATGTCTACAAAATTGGCGGTATCGGAACTGTGCCCGTCGGTCGTGTTGAGACTGGTATCATCAAGGCCGGAATGGTCGTCACTTTCGCTCCCTCCAACGTCACCACTGAAGTCAAGTCCGTCGAAATGCATCACGAACAGCTTGAGCAGGGTAACCCTGGTGACAATGTCGGTTTCAACGTCAAGAACGTGTCAGTGAAGGATATTCGTCGTGGCAACGTGGCCTCCGACTCCAAGAACGATCCCGCCAAGGAAGCGGCCTCTTTCAACGCACAGGTCATCGTCCTCAACCACCCTGGTCAAATTGGTGCTGGTTACGCCCCGGTTCTCGACTGTCACACCGCCCACATCGCCTGCAAGTTCGCCGAGCTCATCGAGAAGATCGATCGTCGTACGGGTAAATCCATTGAAAACTCGCCTAAATTCGTCAAGTCGGGTGACGCCTGCATCGTCAAGCTCGTTCCCAGCAAGCCTATG

>SB2238_Laccaria_bicolor_PORTUGAL_KM067892_KU685886_KU686030_

TCTGA-TGTGGCTGTTAGCTGGC-TTTTCGAAGCATGTGCTCGT-CCATCATCTTT-ATCTCT--CCA-CCTGTGCACATTTTGTAGTC-TT-GGATAACTCTCGGGGCAACCTCGGATT----TTAG-GATCGCCGTGC---TGT---ACAAGTCGGCTTTCCTTTCATTTCC-AAGACTATGTTTT----T--A-TATACA-CCAAAGTATGTTTAAAGAATGTCATC-AATGGGAAC---TTGTTTCCTA--T-AAAATTATACAACTTTCAGCAACGGATCTCTTGGCTCTCGCATCGATGAAGAACGCAGCGAAATGCGATAAGTAATGTGAATTGCAGAATTCAGTGAATCATCGAATCTTTGAACGCACCTTGCGCTCCTTGGTATTCCGAGGAGCATGCCTGTTTGAGTGTCATTAAA-TTCTCAA-CCTTCCATC--------T-TTTATTAGCTTGG--TTAGGCTTGGA-TGT-GGGGG--TTGCGGGCTTC-AT-----A---AACGAGGTCGGCTCTCCTTAAATGCATTAGCGGAACTTTTGTGGACCGTCTA-TTGGTGTGAT-AATTATCTACGCCGTGGAT-GTAAAGCAG-CTTTAT---GAAGTT-CAGCTTCT-AACCGTCC---ATTCATTTGGACAA----TTTTTGACAA-TTTGAAAGCTCAAATTTAAAATCTGGCAGT-CTTTGGCTGT-CCGAGTTGTAATCTAGAGAAGTATTATCCGCGCTGGACCGTGTACAAGTCTCCTGGAATGGGGCGTCATAGAGGGTGAGAATCCCGTCTTTGACACGGACTGCCAGGGC-TTTGTGATGTGCTCTCAAAGAGTCGAGTTGTTTGGGAATGCAGCTCAAAATGGGTGGTAAATTCCATCTAAAGCTAAATATTGGCGAGAGACCGATAGCGAACAAGTACCGTGAGGGAAAGATGAAAAGAACTTTGGAAAGAGAGTTAAACAGTACGTGAAATTGCTGAAAGGGAAACGCTTGAAGTCAGTCGCGTTGGCCAGGGATCAACCCTTGC----TCTTTT---GCTTGGCTTACTTTCTGGTCGATGGG-TCAGCATCAATTTTGACTGGTGG-AAAAAGTTCAAGGGAATGTGGCAT-CTTCGGATGTGTTATAGCCTTTGTTCGCATACATCAGTTGGGATTGAGGAACTCAGCACGCCGCAAGGCCGGG---TTTTTAACCACGTACGTGCTTAGGATGCTGGCATAATGGCTTTAATCGACCCGTCTTGAAACACGGACCAAGGAGTCTAACATGCCTGCGAGTGTTTGGGTGAAAAACCCGAGCGCGTAATGAAAGTG-AAAGTTGAGATCCCTGTCGCGGGGAGCATTGACGCCCGGATCTGACCTTTTGTGACGATTCTGCGGTAGAGCATGTTTTCCGCATGCTTTTCCGAAAGTTGACCAAAGACGTTTATCGTTATCTACAAAAGgttggtgacctccgctcaagtgcc-----atgaaatgtgttga-cctctct---ccttcagTGCGTCGAGACCCACAAGGAGTTCAACCTTGCCCTTGCAGTTAAGCATCAAACCATCACAAACGGCCTCAAATACTCTCTGGCGACAGGTAACTGGGGAGATCAGAAAAAGTCAATGTCTTCCAAGGCAGGAGTATCTCAGGTCTTGAACAGATACACATATGCATCCACTCTGTCACATCTTCGTAGGTGTAACACTCCTCTAGGGCGCGAAGGCAAGATCGCCAAACCTCGTCAGTTGCACAACACTCATTGGGGTATGGTGTGTCCTGCGGAAACTCCTGAAGGTCAAGCCTGTGGCCTTGTCAAGAACCTCGCCCTTATGGCGTGCATATCAGTCGGGTCTTACTCCGCGCCCGTCATCGAGTTTTTGGAGGAATGGGGATTGGAGTCATTGGAAGAGAACGCGCACTCATCCACACCTTGCACAAAAGTTTTCGTGAACGGTGTCTGGATGGGTGTGCATCGCGATCCTGCCAATTTGGTGAAGACGATAAAGAAATTGAGAAGGAAAGACGACATTAGCCCAGAAGTGTCAGTTGTGCGAGACATCAGGGAGAAGGAATTGAGGTTGTACACCGATGCTGGACGTGTTTGTCGACCACTCTTCATCGTCGAGAACCAACAGCTGGCACTTCAGAAGAAGCACGTCAAATGGCTCAGTAACGGCCTCAACGATGATGGCGACGAATACAAATGGGAACATCTGGTAAAGGGTGGCATCATTGAGTTACTGGATGCTGAGGAAGAGGAAACAGTGATGATATCTATGACCCCTGAAGATCTTGAAAATTCTCGCCTACAGCAAAGCGGTGTTGATCCTCACGCGAACGACGGCGAGTTTGATCCAGCAGCTCGATTGAAGGCTGGCACTCACGCACACACATGGACACATTGCGAAATTCATCCTAGCATGATTCTTGGCATTTGTGCCAGCATTATTCCTTTCCCTGACCAT------------------------------------------------------------------------------------------------------------------------------------------------------------------------------------------------------------------------------------------------------------------------------------------------------------------------------------------------------------------------------------------------------------------------------------------------------------------------------------------------------------------------------------------------------------------------------------------------------------------------------------------------------------------------------------------------------------------------------------------------------------------------------------------------------------------------------------------------------------------------------------------------------------------------------------------------------------------------------------------------------------------------------------------------

>SB2239_Laccaria_bicolor_PORTUGAL_KM067893_KU685887_KU686031_

TCTGA-TGTGGCTGTTAGCTGGC-TTTTCGAAGCATGTGCTCGT-CCATCATCTTT-ATCTCT--CCA-CCTGTGCACATTTTGTAGTC-TT-GGATAACTCTCGGGGCAACCTCGGATT----TTAG-GATCGCCGTGC---TGT---ACAAGTCGGCTTTCCTTTCATTTCC-AAGACTATGTTTT----T--A-TATACA-CCAAAGTATGTTTAAAGAATGTCATC-AATGGGAAC---TTGTTTCCTA--T-AAAATTATACAACTTTCAGCAACGGATCTCTTGGCTCTCGCATCGATGAAGAACGCAGCGAAATGCGATAAGTAATGTGAATTGCAGAATTCAGTGAATCATCGAATCTTTGAACGCACCTTGCGCTCCTTGGTATTCCGAGGAGCATGCCTGTTTGAGTGTCATTAAA-TTCTCAA-CCTTCCATC--------T-TTTATTAGCTTGG--TTAGGCTTGGA-TGT-GGGGG--TTGCGGGCTTC-AT-----A---AACGAGGTCGGCTCTCCTTAAATGCATTAGCGGAACTTTTGTGGACCGTCTA-TTGGTGTGAT-AATTATCTACGCCGTGGAT-GTGAAGCAG-CTTTAT---GAAGTT-CAGCTTCT-AACCGTCC---ATTCATTTGGACAA----TTTTTGACAA-TTTGAAAGCTCAAATTTAAAATCTGGCAGT-CTTTGGCTGT-CCGAGTTGTAATCTAGAGAAGTATTATCCGCGCTGGACCGTGTACAAGTCTCCTGGAATGGGGCGTCATAGAGGGTGAGAATCCCGTCTTTGACACGGACTGCCAGGGC-TTTGTGATGTGCTCTCAAAGAGTCGAGTTGTTTGGGAATGCAGCTCAAAATGGGTGGTAAATTCCATCTAAAGCTAAATATTGGCGAGAGACCGATAGCGAACAAGTACCGTGAGGGAAAGATGAAAAGAACTTTGGAAAGAGAGTTAAACAGTACGTGAAATTGCTGAAAGGGAAACGCTTGAAGTCAGTCGCGTTGGCCAGGGATCAACCCTTGC----TCTTTT---GCTTGGCTTACTTTCTGGTCGATGGG-TCAGCATCAATTTTGACTGGTGG-AAAAAGTTCAAGGGAATGTGGCAT-CTTCGGATGTGTTATAGCCTTTGTTCGCATACATCAGTTGGGATTGAGGAACTCAGCACGCCGCAAGGCCGGG---TTTTTAACCACGTACGTGCTTAGGATGCTGGCATAATGGCTTTAATCGACCCGTCTTGAAACACGGACCAAGGAGTCTAACATGCCTGCGAGTGTTTGGGTGAAAAACCCGAGCGCGTAATGAAAGTG-AAAGTTGAGATCCCTGTCGCGGGGAGCATTGACGCCCGGATCTGACCTTTTGTGACGATTCTGCGGTAGAGCATGTTTTCCGCATGCTTTTCCGAAAGTTGACCAAAGACGTTTATCGTTATCTACAAAAGgttggtgacctccgctcaagtgcc-----atgaaatgtgttga-cctctct---ccttcagTGCGTCGAGACCCACAAGGAGTTCAACCTTGCCCTTGCAGTTAAGCATCAAACCATCACAAACGGCCTCAAATACTCTCTGGCGACAGGTAACTGGGGAGATCAGAAAAAGTCAATGTCTTCCAAGGCAGGAGTATCTCAGGTCTTGAACAGATACACATATGCATCCACTCTGTCACATCTTCGTAGGTGTAACACTCCTCTAGGGCGCGAAGGCAAGATCGCCAAACCTCGTCAGTTGCACAACACTCATTGGGGTATGGTGTGTCCTGCGGAAACTCCTGAAGGTCAAGCCTGTGGCCTTGTCAAGAACCTCGCCCTTATGGCGTGCATATCAGTCGGGTCTTACTCCGCGCCCGTCATCGAGTTTTTGGAGGAATGGGGATTGGAGTCATTGGAAGAGAACGCGCACTCATCCACACCTTGCACAAAAGTTTTCGTGAACGGTGTCTGGATGGGTGTGCATCGCGATCCTGCCAATTTGGTGAAGACGATAAAGAAATTGAGAAGGAAAGACGACATTAGCCCAGAAGTGTCAGTTGTGCGAGACATCAGGGAGAAGGAATTGAGGTTGTACACCGATGCTGGACGTGTTTGTCGACCACTCTTCATCGTCGAGAACCAACAGCTGGCACTTCAGAAGAAGCACGTCAAATGGCTCAGTAACGGCCTCAACGATGATGGCGACGAATACAAATGGGAACATCTGGTAAAGGGTGGCATCATTGAGTTACTGGATGCTGAGGAAGAGGAAACAGTGATGATATCTATGACCCCTGAAGATCTTGAAAATTCTCGCCTACAGCAAAGCGGTGTTGATCCTCACGCGAACGACGGCGAGTTTGATCCAGCAGCTCGATTGAAGGCTGGCACTCACGCACACACATGGACACATTGCGAAATTCATCCTAGCATGATTCTTGGCATTTGTGCCAGCATTATTCCTTTCCCTGACCAT------------------------------------------------------------------------------------------------------------------------------------------------------------------------------------------------------------------------------------------------------------------------------------------------------------------------------------------------------------------------------------------------------------------------------------------------------------------------------------------------------------------------------------------------------------------------------------------------------------------------------------------------------------------------------------------------------------------------------------------------------------------------------------------------------------------------------------------------------------------------------------------------------------------------------------------------------------------------------------------------------------------------------------------------

>GMM6131_Laccaria_alba_CHINA_JX504131_JX504210_KU685930_KU686079

CCTGA-TGTGACTGTTAGCTGGC-TTTTCGAAGCATGTGCTCGT-CCATCATCTTTAATCTCT--CCA-CCTGTGCACATTTTGTAGTC-TT-GGATACCTCTCGAGGCAA-CTCGGATT---TTTAG-GATCGCCGTGC---TGT---ACAAGTCGGCTTTTCTTTCATTTCC-AAGACTATGTTTT----T--A-TATACA-CCAAAGTATGTTTAAAGAATGTCATT-AATAGGAAC---TTGTTTCCTA--T-AAAATTATACAACTTTCAGCAACGGATCTCTTGGCTCTCGCATCGATGAAGAACGCAGCGAAATGCGATAAGTAATGTGAATTGCAGAATTCAGTGAATCATCGAATCTTTGAACGCACCTTGCGCTCCTTGGTATTCCGAGGAGCATGCCTGTTTGAGTGTCATTAAA-TTCTCAA-CCTTCCAAC--------T-TTTATTAGCTTGG--ATAGGCTTGGA-TGT-GGGGG--TTGCAGGCTTC-AT-----C---AATGAGGTCAGCTCTTCTTAAATGCATTAGCGGAACTTTTGTGGACCGTCTA-TTGGTGTGAT-AATTATCTACGCCGTGGAT-GAAAAACAG-CTTTAT---CAAGTT-CAGCTTCT-AACCGTCC---ATTGACTTGGACAA-----TTTTGACAA-TTTGAAAGCTCAAATTTAAAATCTGGCAGT-CTTTGGCTGT-CCGAGTTGTAATCTAGAGAAGTATTATCCGCGCTGGACCGTGTACAAGTCTCCTGGAATGGAGCGTCATAGAGGGTGAGAATCCCGTCTTTGACACGGACTGCCAGGGC-TTTGTGATGTGCTCTCGAAGAGTCGAGTTGTTTGGGAATGCAGCTCAAAATGGGTGGTAAATTCCATCTAAAGCTAAATATTGGCGAGAGACCGATAGCGAACAAGTACCGTGAGGGAAAGATGAAAAGAACTTTGGAAAGAGAGTTAAACAGTACGTGAAATTGCTGAAAGGGAAACGCTTGAAGTCAGTCGCATTGGCCAGGGATCAA-CCTTGC----TTTTTT---GCTTGGCTTACTTTCTGGTTGATGGG-TCAGCATCAATTTTGACTGGTAG-AAAAAGTTCAAGGGAATGTGGCAT-CTTCGGATGTGTTATAGCCTTTGTTCACATATATCAGTTGGGATTGAGGAACTCAGCACGCCGCAAGGCCGGG---TTTTTAACCACGTACGTGCTTAGGATGCTGGCATAATGGCTTTAATCGACCCGTCTTGAAACACGGACCAAGGAGTCTAACATGCCTGCAAGTGTTTGGGTGAAAAACCCGAGCGCGTAATGAAAGTG-AAAGTTGAGATCCCTGTCATGGGGAGCATTGACGCCCGGATCTGACCTTTTGTGACGATTCTGCGGTAGAGCATGT-------------TTCCGAAAGTTGACCAAGGACGTTTTTCGtTATCTACAAAAGGTTGCTGACCTTCGCTCAAGTTTC-----ATGAGATATGCTGA-CCCCTCT---TCTTCAGTGCGTCGAGACCCACAAGGAGTTCAACCTTGCCCTTGCAGTTAAGCATCAAACCATCACAAACGGCCTCAAATACTCTCTGGCGACAGGTAACTGGGGAGATCAGAAAAAGTCAATGTCTTCCAAGGCAGGAGTATCTCAGGTCTTGAACAGATACACATATGCATCCACTCTGTCACATCTTCGTCGGTGTAACACTCCTCTAGGGCGCGAAGGCAAGATCGCCAAACCTCGTCAGTTGCACAACACTCATTGGGGTATGGTGTGTCCTGCGGAAACTCCTGAAGGTCAAGCCTGTGGTCTTGTCAAGAACCTCGCCCTTATGGCGTGCATATCAGTCGGGTCTTACTCYGCGCCCGTTATCGAGTTTTTGGAGGAGTGGGGATTGGAGTCATTGGAAGAGAACGCGCACTCATCAACACCTTGCACAAAAGTTTTCGTGAACGGTGTCTGGATGGGTGTGCATCGTGATCCTGCCAATCTGGTGAAGACGATAAAGAAATTGAGAAGGAAAGACGACATTAGCCCAGAAGTGTCAGTTGTGCGAGACATCAGGGAGAAGGAATTGAGGTTGTACACCGATGCTGGACGTGTTTGTCGACCACTCTTCATTGTCGAGAACCAACAGCTGGCACTTCAGAAGAAGCACGTCAAATGGCTCAGTAACGGCCTCAACGATGATGGCGACGAGTACAAATGGGAACATCTAGTAAAGGGTGGCATCATCGAGTTACTGGATGCTGAGGAAGAAGAAACAGTGATGATATCCATGACCCCTGAAGATCTTGAAAATTCTCGCCTACAGCAGAGTGGTGTTGATCCTCACGCGAACGACGGCGAGTTTGATCCAGCAGCTCGATTGAAGGCTGGCACTCACGCACACACATGGACACATTGCGAAATTCATCCTAGCATGATTCTTGGCATTTGTGCCAGCATTGTTCCTTTCCCT----------------------------------------------------------------------------CGCGAGCACGCCCTCCTCGCTTTCACCCTCGGTGTGCGGCAACTCATCGTTGCCGTCAACAAGATGGACACCACTAAGGTAAGAAACGTTCCAC-CTATAGTTAGGCTTCTTACACATTCTCATCATCTCA-TGTAGTGGAGCGAGGACCGTTTCAACGAAATTATCAAGGAGACGTCAACCTTCATCAAGAAGGTTGGTTACAACCCAAAGGCTGTTGCCTTTGTCCCTATTTCCGGCTGGCACGGCGACAACATGTTGGAGGAGTCCGCTAAGTAAGCATTC--TCGTATCATCATGATCGCCCCGTCGCTCATGTTGCACACAAGCATGCCTTGGTACAAGGGCTGGACCAGGGAGACCAAGGCTGGTGTCGTCAAGGGCAAGACTCTCCTCGATGCTATTGATGCCATTGAACCCCCCGTCCGACCTTCCGACAAACCCCTCCGTCTCCCCCTCCAGGATGTCTACAAAATTGGCGGTATCGGAACTGTGCCCGTCGGTCGTGTTGAGACTGGTATCATCAAGGCCGGAATGGTCGTCACTTTCGCTCCCTCCAACGTCACCACTGAAGTCAAGTCCGTCGAAATGCATCACGAACAGCTCGAGCAGGGTAATCCTGGTGACAATGTCGGTTTCAATGTCAAGAACGTGTCAGTAAAGGATATTCGTCGTGGCAACGTGGCCTCCGACTCCAAGAACGATCCAGCCAAGGAAGCAGCCTCTTTCAACGCACAGGTCATTGTCCTCAACCACCCTGGTCAAATTGGTGCTGGTTACGCCCCAGTTCTCGACTGTCACACTGCCCACATCGCCTGCAAGTTCGCCGAGCTCATCGAGAAGATCGATCGTCGTACGGGTAAATCCATCGAAAACTCGCCTAAATTTGTCAAGTCGGGTGACGCCTGCATCGTCAAACTCGTTCCCAGCAAGCCTATG

>F1120750_Laccaria_alba_CHINA_JX504126_JX504242__

----A-TGTGACTGTTAGCTGGC-TTTTCGAAGCATGTGCTCGT-CCATCATCTTTAATCTCT--CCA-CCTGTGCACATTTTGTAGTC-TT-GGATACCTCTCGAGGCAA-CTCGGATT---TTTAG-GATCGCCGTGC---TGT---ACAAGTCGGCTTTTCTTTCATTTCC-AAGACTATGTTTT----T--A-TATACA-CCAAAGTATGTTTAAAGAATGTCATT-AATAGGAAC---TTGTTTCCTA--T-AAAATTATACAACTTTCAGCAACGGATCTCTTGGCTCTCGCATCGATGAAGAACGCAGCGAAATGCGATAAGTAATGTGAATTGCAGAATTCAGTGAATCATCGAATCTTTGAACGCACCTTGCGCTCCTTGGTATTCCGAGGAGCATGCCTGTTTGAGTGTCATTAAA-TTCTCAA-CCTTCCAAC--------T-TTTATTAGCTTGG--ATAGGCTTGGA-TGT-GGGGG--TTGCAGGCTTC-AT-----C---AATGAGGTCAGCTCTTCTTAAATGCATTAGCGGAACTTTTGTGGACCGTCTA-TTGGTGTGAT-AATTATCTACGCCGTGGAT-GAAAAACAG-CTTTAT---CAAGTT-CAGCTTCT-AACCGTCC---ATTGACTTGGACAA-----TTTTGACAA-TTTGAAAGCTCAAATTTAAAATCTGGCAGT-CTTTGGCTGT-CCGAGTTGTAATCTAGAGAAGTATTATCCGCGCTGGACCGTGTACAAGTCTCCTGGAATGGGGCGTCATAGAGGGTGAGAATCCCGTCTTTGACACGGACTGCCAGGGC-TTTGTGATGTGCTCTCAAAGAGTCGAGTTGTTTGGGAATGCAGCTCAAAATGGGTGGTAAATTCCATCTAAAGCTAAATATTGGCGAGAGACCGATAGCGAACAAGTACCGTGAGGGAAAGATGAAAAGAACTTTGGAAAGAGAGTTAAACAGTACGTGAAATTGCTGAAAGGGAAACGCTTGAAGTCAGTCGCGTTGGCCAGGGATCAA-CCTTGC----TTTTTT---GCTTGGCTTACTTTCTGGTCGATGGG-TCAGCATCAATTTTGACTGGTGG-AAAAAGTTCAAGGGAATGTGGCAT-CTTCGGATGTGTTATAGCCTTTGTTCGCATACATTGGTTGGGATTGAGGAACTCAGCACGCCGCAAGGCCGGG---TTTTTAACCACGTACGTGCTTAGGATGCTGGCATAATGGCTTTAATCGACCCGTCTTGAAACACGGACCAAGGAGTCTAACATGCCTGCGAGTGTTTGGGTGAAAAACCCGAGCGCGTAATGAAAGTG-AAAGTTGAGATCCCTGTCGTGGGGAGCATTGACGCCCGGATCTGACCTTTTGTGACGATTCTGCGGTAGAGCATGT-----------------------------------------------------------------------------------------------------------------------------------------------------------------------------------------------------------------------------------------------------------------------------------------------------------------------------------------------------------------------------------------------------------------------------------------------------------------------------------------------------------------------------------------------------------------------------------------------------------------------------------------------------------------------------------------------------------------------------------------------------------------------------------------------------------------------------------------------------------------------------------------------------------------------------------------------------------------------------------------------------------------------------------------------------------------------------------------------------------------------------------------------------------------------------------------------------------------------------------------------------------------------------------------------------------------------------------------------------------------------------------------------------------------------------------------------------------------------------------------------------------------------------------------------------------------------------------------------------------------------------------------------------------------------------------------------------------------------------------------------------------------------------------------------------------------------------------------------------------------------------------------------------------------------------------------------------------------------------------------------------------------------------------------------------------------------------------------------------------------------------------------------------------------

>F1133825_Laccaria_proxima_USA_MS_KU686064_KU685786_KU686065_

-CTGA-TGTGACTGTTAGCTGGC-TTTTCGAAGCATGTGCTCGT-CCATCATCTTT-ATCTCT--CCA-CCTGTGCACATTTTGTAGTC-TT-GGATACCTCTCGAGGAAA-CTYGGATT----TTAG-GATCGCCGTGC---TGT---ACAAGTCGGCTTTTCTTTCATTTCC-AAGACTATGTTTT----T--A-TATACA-CCAAAGTATGTTTATAGAATGTCATC-AATGGGAAC---TTGTTTCCTA--T-AAAATTATACAACTTTCAGCAACGGATCTCTTGGCTCTCGCATCGATGAAGAACGCAGCGAAATGCGATAAGTAATGTGAATTGCAGAATTCAGTGAATCATCGAATCTTTGAACGCACCTTGCGCTCCTTGGTATTCYGAGGAGCATGCCTGTTTGAGTGTCATTAAW-TTCTCAA-CCTTCCAAC--------T-TTTATTAGCTTGG--TTAGGCTTGGA-TGT-GGGGG--TTGCAGGCTTC-AT-----C---ACTGAGGTCGGCTCTCCTTAAATGCATTAGCGGAACTTTTGTGGACCGTCTA-TTGGTGTGAT-AATTATCTACGCCGTGGAT-GTGAAGCAG-CTTTAT---GAAGTT-CTGCTTCT-AACCGTCC---ATTGACTTGGACAA-----TTTTGACAA-TTTGAAAGCTCAAATTTAAAATCTGGCAGT-CTTTGGCTGT-CCGAGTTGTAATCTAGAGAAGTATTATCCGCGCTGGACCGTGTACAAGTCTCCTGGAATGGAGCGTCATAGAGGGTGAGAATCCCGTCTTTGACACGGACTGCCAGGGC-TTTGTGATATGCTCTCAAAGAGTCGAGTTGTTTGGGAATGCAGCTCAAAATGGGTGGTAAATTCCATCTAAAGCTAAATATTGGCGAGAGACCGATAGCGAACAAGTACCGTGAGGGAAAGATGAAAAGAACTTTGGAAAGAGAGTTAAACAGTACGTGAAATTGCTGAAAGGGAAACGCTTGAAGTCAGTCGCGTTGGCCAGGGATCAA-CCTTGC------TTTT---GCTTGGCTTACTTTCTGGTCGATGGG-TCAGCATCAATTTTGACTGGTGG-AAAAAGTTCAAGGGAATGTGGCAT-CTTCGGATGTGTTATAGCCTTTGTTCACATACATTGGTTGGGATTGAGGAACTCAGCACGCCGCAAGGCCGGG---TTTTTAACCACGTACGTGCTTAGGATGCTGGCATAATGGCTTTAATCGACCCGTCTTGAAACACGGACCAAGGAGTCTAACATGCCTGCGAGTATTTGGGTGAAAAACCCGAGTGCGTAATGAAAGTG-AAAGTTGAGATCCCTGTCGTGGGGAGCATTGACGCCCGGATCTGACCTTTTGTGACGATTCTGCGGTAGAGCATGTTTTCCGCATGCTTTTCCGAAAGTTGACCAAGGACGTTTATCGTTATCTACAAAAGGTTGCTGACTTTCRCTCAAGTGCC-----ATGAGACGTRTTGA-YCTCTCTNNNNNNNNNNNNNNNNNNNNNNNNNNNNNNNNNNNNNNNNNNNNNNNNNNNNNNNNNNNNNNNNNNNNNNNNNNNNNNNNNNNNNNNNNNNNNNNNNNNNNNNNNNNNNNNNNNNNNNAAGTCAATGTCTTCCAAGGCAGGAGTATCTCAGGTCTTGAACAGATACACATATGCATCCACTCTGTCACATCTYCGTCGGTGTAACACTCCTCTRGGACGCGAAGGCAAGATCGCCAARCCTCGTCAGTTGCACAACACCCATTGGGG----------------------------------------------------------------------------------------------------------------------------------------------------------------------------------------------------------------------------------------------------------------------------------------------------------------------------------------------------------------------------------------------------------------------------------------------------------------------------------------------------------------------------------------------------------------------------------------------------------------------------------------------------------------------------------------------------------------------------------------------------------------------------------------------------------------------------------------------------------------------------------------------------------------------------------------------------------------------------------------------------------------------------------------------------------------------------------------------------------------------------------------------------------------------------------------------------------------------------------------------------------------------------------------------------------------------------------------------------------------------------------------------------------------------------------------------------------------------------------------------------------------------------------------------------------------------------------------------------------------------------------------------------------------------------------------------------------------------------------------------------------------------------

>F1068333_Laccaria_amethysteooccidentalis_USA_WI_KU685629_KU685773_KU686061_

-CTGA-TGTGACTGTTAGCTGGC-TTTTCGAAGCATGTGCTCGT-CCATCATCTTT-ATCTCT--CCA-CCTGTGCACATTTTGTAGTC-TT-GGATACCTCTCGAGGAAA-CTYGGATT----TTAG-GATCGCCGTGC---TGT---ACAAGTCGGCTTTTCTTTCATTTCC-AAGACTATGTTTT----T--A-TATACA-CCAAAGTATGTTTATAGAATGTCATC-AATGGGAAC---TTGTTTCCTA--T-AAAATTATACAACTTTCAGCAACGGATCTCTTGGCTCTCGCATCGATGAAGAACGCAGCGAAATGCGATAAGTAATGTGAATTGCAGAATTCAGTGAATCATCGAATCTTTGAACGCACCTTGCGCTCCTTGGTATTCCGAGGAGCATGCCTGTTTGAGTGTCATTAAA-TTCTCAA-CCTTCCAAC--------T-TTTATTAGCTTGG--TTAGGCTTGGA-TGT-GGGGG--TTGCAGGCTTC-AT-----C---ACTGAGGTCGGCTCTCCTTAAATGCATTAGCGGAACTTTTGTGGACCGTCTA-TTGGTGTGAT-AATTATCTACGCCGTGGAT-GTGAAGCAG-CTTTAT---GAAGTT-CTGCTTCT-AACCGTCC---ATTGACTTGGACAA-----TTTTGACAA-TTTGAAAGCTCAAATTTAAAATCTGGCAGT-CTTTGGCTGT-CCGAGTTGTAATCTAGAGAAGTATTATCCGCGCTGGACCGTGTACAAGTCTCCTGGAATGGAGCGTCATAGAGGGTGAGAATCCCGTCTTTGACACGGACTGCCAGGGC-TTTGTGATATGCTCTCAAAGAGTCGAGTTGTTTGGGAATGCAGCTCAAAATGGGTGGTAAATTCCATCTAAAGCTAAATATTGGCGAGAGACCGATAGCGAACAAGTACCGTGAGGGAAAGATGAAAAGAACTTTGGAAAGAGAGTTAAACAGTACGTGAAATTGCTGAAAGGGAAACGCTTGAAGTCAGTCGCGTTGGCCAGGGATCAA-CCTTGC------TTTT---GCTTGGCTTACTTTCTGGTCGATGGG-TCAGCATCAATTTTGACTGGTGG-AAAAAGTTCAAGGGAATGTGGCAT-CTTCGGATGTGTTATAGCCTTTGTTCACATACATTGGTTGGGATTGAGGAACTCAGCACGCCGCAAGGCCGGG---TTTTTAACCACGTACGTGCTTAGGATGCTGGCATAATGGCTTTAATCGACCCGTCTTGAAACACGGACCAAGGAGTCTAACATGCCTGCGAGTATTTGGGTGAAAAACCCGAGTGCGTAATGAAAGTG-AAAGTTGAGATCCCTGTCGTGGGGAGCATTGACGCCCGGATCTGACCTTTTGTGACGATTCTGCGGTAGAGCATGTTTTCCGCATGCTTTTCCGAAAGTTGACCAAGGACGTTTATCGTTATCTACAAAAGGTTGCTGACTTTCACTCAAGTGCC-----ATGAGACGTRTTGA-CCTCTCW---TCTTCAGTGCGTCGAGACCCACAAGGAGTTYAACCTTGCCCTTGCAGTTAAGCATCAAACCATCACAAACGGCCTCAAATACTCTCTGGCGACAGGTAACTGGGGGGATCAGAAAAAGTCAATGTCTTCCAAGGCAGGAGTATCTCAGGTCTTGAACAGATACACATATGCATCCACTCTGTCACATCTTCGTCGGTGTAACACTCCTCTRGGGCGCGAAGGCAAGATCGCCAAACCTCGTCAGTTGCACAACACCMAYTGGGG----------------------------------------------------------------------------------------------------------------------------------------------------------------------------------------------------------------------------------------------------------------------------------------------------------------------------------------------------------------------------------------------------------------------------------------------------------------------------------------------------------------------------------------------------------------------------------------------------------------------------------------------------------------------------------------------------------------------------------------------------------------------------------------------------------------------------------------------------------------------------------------------------------------------------------------------------------------------------------------------------------------------------------------------------------------------------------------------------------------------------------------------------------------------------------------------------------------------------------------------------------------------------------------------------------------------------------------------------------------------------------------------------------------------------------------------------------------------------------------------------------------------------------------------------------------------------------------------------------------------------------------------------------------------------------------------------------------------------------------------------------------------------

>SB2133_Laccaria_laccata_PORTUGAL_KM067887_KU685884_KU686027_KU686139

CCTGA-TGTGACTGTTAGCTGGC-TTTTCGAAGCATGTGCTCGT-CCGTCATCTTTAATCTCT--CCA-CCTGTGCACATTTTGTAGTC-TT-GGATACCTCTCGAGGAAA-CTCGGATT----TTAG-GATCGCCGTGC---TGT---ACAAGTCGGCTTTTCTTTCATTTCC-AAGACTATGTTTT-------A-TATACA-CCAAAGTATGTTTAAAGAATGTCATC-AATAGGAAC---TTGTTTCCTA--T-AAAATTATACAACTTTCAGCAACGGATCTCTTGGCTCTCGCATCGATGAAGAACACAGCGAAATGCGATAAGTAATGTGAATTGCAGAATTCAGTGAATCATCGAATCTTTGAACGCACCTTGCGCTCCTTGGTATTCCGAGGAGCATGCCTGTTTGAGTGTCATTAAA-TTCTCAA-CCTTCCAGC--------T-TTTATTGGCTTGG--TTAGGCTTGGA-TGT-GGGGG--TTGCAGGCTTC-AT-----C---AATGAGGTCAGCTCTCCTTAAATGCATTAGCAGAACTTTTGTGGACTGTCTA-TTGGTGTGAT-AATTATCTACGCCGTGGAT-GTGACGCAA-CTTTAW---GAAGTT-CAGCTTCT-AACTGTCC---ATTGACTTGGACAA-----TTTTGACAA-TTTGAAAGCTCAAATTTAAAATCTGGCAGT-CTTTGGCTGT-CCGAGTTGTAATCTAGAGAAGTATTATCCGCGCTGGACCGTGTACAAGTCTCCTGGAATGGAGCGTCATAGAGGGTGAGAATCCCGTCTTTGACACGGACTGCCAGGGC-TTTGTGATGTGCTCTCGAAGAGTCGAGTTGTTTGGGAATGCAGCTCAAAATGGGTGGTAAATTCCATCTAAAGCTAAATATTGGCGAGAGACCGATAGCGAACAAGTACCGTGAGGGAAAGATGAAAAGAACTTTGGAAAGAGAGTTAAACAGTACGTGAAATTGCTGAAAGGGAAACGCTTGAAGTCAGTCGCGTTGGCCAGGGATCAA-CCTTGC--TATTTTTT---GCTTGGCTTACTTTCTGGTCGATGGG-TCAGCATCAATTTTGACTGGTGG-AAAAAGTTCAAGGGAATGTGGCAT-CTTCGGATGTGTTATAGCCTTTGTTCACATATATCAGTTGGGATTGAGGAACTCAGCACGCCGCAAGGCCGGG---TTTTTAACCACGTACGTGCTTAGGATGCTGGCATAATGGCTTTAATCGACCCGTCTTGAAACACGGACCAAGGAGTCTAACATGCCTGCGAGTGTTTGGGTGAAAAACCCGAGCGCGTAATGAAAGTG-AAAGTTGAGATCCCTGTCGTGGGGAGCATTGACGCCCGGATCTGACCTTTTGTGACGATTCTGCGGTAGAGCATGTATTCCGCATGCTTTTCCGAAAGTTGACTAAGGACGTTTATCGTTATCTGCAAAAGgttgccgaccttcgctcaagtgcc-----atgagatatgctga-cccctct---tcttcagTGCGTCGAGACCCACAAGGAGTTCAACCTTGCCCTTGCAGTTAAGCATCAAACCATCACAAACGGCCTCAAATACTCTCTGGCGACAGGTAACTGGGGAGATCAGAAAAAGTCAATGTCTTCCAAGGCAGGAGTATCTCAGGTCTTGAACAGATACACATATGCATCCACTCTGTCACATCTTCGTCGGTGTAACACTCCTCTAGGGCGCGAAGGCAAGATCGCCAAACCTCGTCAGTTGCATAACACTCATTGGGGTATGGTGTGTCCTGCGGAAACTCCTGAAGGTCAAGCCTGTGGTCTTGTCAAGAACCTCGCCCTTATGGCGTGCATATCAGTCGGGTCTTACTCCGCGCCCGTTATCGAGTTTTTGGAGGAGTGGGGATTGGAGTCATTGGAAGAGAACGCGCACTCATCAACACCTTGCACAAAAGTTTTCGTGAACGGTGTCTGGATGGGTGTACATCGCGATCCTGCCAATTTGGTGAAGACGATAAAGAAATTGAGAAGGAAAGACGACATTAGCCCAGAAGTGTCAGTTGTGCGAGACATCAGGGAGAAGGAATTGAGGTTGTACACCGATGCTGGACGTGTTTGTCGACCACTCTTCATCGTCGAGAACCAACAGCTGGCACTTCAGAAGAAGCACGTCAAATGGCTCAGTAACGGCCTCAACGATGATGGCGACGATTACAAATGGGAACATCTGGTAAAGGGTGGCATCATTGAGTTACTGGATGCCGAGGAAGAGGAAACAGTGATGATTTCCATGACCCCTGAAGATCTTGAAAATTCTCGCCTACAGCAAAGTGGTGTTGATCCTCACGCGAACGACGGCGAGTTTGATCCAGCAGCTCGATTGAAAGCTGGCACTCACGCACACACATGGACACATTGCGAAATTCATCCTAGCATGATTCTTGGCATTTGTGCCAGCATTATTCCTTTCCCTGATCATCTGCGCCATTCTCATCATTGCTGGTGGTACTGGTGAGTTCGAGGCTGGTATCTCCAAGGATGGCCAGACCCGCGAGCACGCTCTCCTCGCTTTCACCCTCGGCGTGCGGCAACTCATCGTTGCCGTCAACAAGATGGACACCACTAAGGTAAGAAACGTTTCAC-TTATAGTTAGGCTTCTTGCACATTCTCATCATCTCA-TGTAGTGGAGCGAGGACCGTTTCAACGAAATCATCAAGGAGACGTCAACCTTCATCAAGAAGGTTGGTTACAACCCCAAGGCCGTTGCCTTTGTTCCTATTTCCGGCTGGCACGGTGACAACATGTTGGAGGAGTCGGCTAAGTAAGGATTC--TCGTATCGTCATGATCGCCCCGTCGCTTATGTTGCACTCAAGCATGCCTTGGTACAAGGGCTGGACCAGGGAGACCAAGGCTGGTCCCGTCAAGGGCAAGACCCTCCTCGATGCTATTGATGCCATTGAACCCCCCGTCCGACCTTCCGACAAACCCCTCCGTCTCCCCCTCCAGGATGTCTACAAAATTGGCGGTATCGGAACTGTGCCCGTCGGTCGTGTTGAGACTGGTATCATCAAGGCCGGAATGGTCGTCACTTTCGCTCCCTCCAACGTCACCACTGAAGTCAAGTCTGTCGAAATGCATCACGAACAGCTCGAGCAGGGTAACCCTGGTGACAATGTCGGTTTCAACGTCAAGAACGTGTCAGTGAAGGATATTCGTCGTGGCAACGTGGCCTCCGACTCCAAGAACGATCCCGCCAAGGAAGCGGCCTCTTTCAACGCACAGGTCATCGTCCTCAACCACCCTGGTCAAATTGGTGCTGGTTACGCCCCAGTTCTCGACTGTCACACCGCCCACATCGCCTGCAAGTTCGCCGAGCTCATCGAGAAGATCGATCGTCGTACGGGTAAATCCATTGAAAACTCGCCTAAATTCGTCAAGTCGGGTGACGCCTGCATCGTCAAGCTCGTTCCCAGCAAGCCTATG

>SB2210_Laccaria_laccata_PORTUGAL_KM067890_KU685885_KU686029_KU686141

CCTGA-TGTGACTGTTAGCTGGC-TTTTCGAAGCATGTGCTCGT-CCGTCATCTTTAATCTCT--CCA-CCTGTGCACATTTTGTAGTC-TT-GGATACCTCTCGAGGAAA-CTCGGATT----TTAG-GATCGCCGTGC---TGT---ACAAGTCGGCTTTTCTTTCATTTCC-AAGACTATGTTTT-------A-TATACA-CCAAAGTATGTTTAAAGAATGTCATC-AATAGGAAC---TTGTTTCCTA--T-AAAATTATACAACTTTCAGCAACGGATCTCTTGGCTCTCGCATCGATGAAGAACGCAGCGAAATGCGATAAGTAATGTGAATTGCAGAATTCAGTGAATCATCGAATCTTTGAACGCACCTTGCGCTCCTTGGTATTCCGAGGAGCATGCCTGTTTGAGTGTCATTAAA-TTCTCAA-CCTTCCAGC--------T-TTTATTGGCTTGG--TTAGGCTTGGA-TGT-GGGGG--TTGCAGGCTTC-AT-----C---AATGAGGTCAGCTCTCCTTAAATGCATTAGCAGAACTTTTGTGGACTGTCTA-TTGGTGTGAT-AATTATCTACGCCGTGGAT-GTGACGCAG-CTTTAT---GAAGTT-CAGCTTCT-AACTGTCC---ATTGACTTGGACAA-----TTTTGACAA-TTTGAAAGCTCAAATTTAAAATCTGGCAGT-CTTTGGCTGT-CCGAGTTGTAATCTAGAGAAGTATTATCCGCGTTGGACCGTGTACAAGTCTCCTGGAATGGAGCGTCATAGAGGGTGAGAATCCCGTCTTTGACACGGACTGCCAGGGC-TTTGTGATGTGCTCTCGAAGAGTCGAGTTGTTTGGGAATGCAGCTCAAAATGGGTGGTAAATTCCATCTAAAGCTAAATATTGGCGAGAGACCGATAGCGAACAAGTACCGTGAGGGAAAGATGAAAAGAACTTTGGAAAGAGAGTTAAACAGTACGTGAAATTGCTGAAAGGGAAACGCTTGAAGTCAGTCGCGTTGGCCAGGGATCAA-CCTTGC--TATTTTTT---GCTTGGCTTACTTTCTGGTCGATGGG-TCAGCATCAATTTTGACTGGTGG-AAAAAGTTCAAGGGAATGTGGCAT-CTTCGGATGTGTTATAGCCTTTGTTCACATATATCAGTTGGGATTGAGGAACTCAGCACGCCGCAAGGCCGGG---TTTTTAACCACGTACGTGCTTAGGATGCTGGCATAATGGCTTTAATCGACCCGTCTTGAAACACGGACCAAGGAGTCTAACATGCCTGCGAGTGTTTGGGTGAAAAACCCGAGCGCGTAATGAAAGTG-AAAGTTGAGATCCCTGTCGTGGGGAGCATTGACGCCCGGATCTGACCTTTTGTGACGATTCTGCGGTAGAGCATGTATTCCGCATGCTTTTCCGAAAGTTGACTAAGGACGTTTATCGTTATCTGCAAAAGgttgccgaccttcgctcaagtgcc-----atgagatatgctga-cccctct---tcttcagTGCGTCGAGACCCACAAGGAGTTCAACCTTGCCCTTGCAGTTAAGCATCAAACCATCACAAACGGCCTCAAATACTCTCTGGCGACAGGTAACTGGGGAGATCAGAAAAAGTCAATGTCTTCCAAGGCAGGAGTATCTCAGGTCTTGAACAGATACACATATGCATCCACTCTGTCACATCTTCGTCGGTGTAACACTCCTCTAGGGCGCGAAGGCAAGATCGCCAAACCTCGTCAGTTGCACAACACTCATTGGGGTATGGTGTGTCCTGCGGAAACTCCTGAAGGTCAAGCCTGTGGTCTTGTCAAGAACCTCGCCCTTATGGCGTGCATATCAGTCGGGTCTTACTCCGCGCCCGTTATCGAGTTTTTGGAGGAGTGGGGATTGGAGTCATTGGAAGAGAACGCGCACTCATCAACACCTTGCACAAAAGTTTTCGTGAACGGTGTCTGGATGGGTGTACATCGCGATCCTGCCAATTTGGTGAAGACGATAAAGAAATTGAGAAGGAAAGACGACATTAGCCCAGAAGTGTCAGTTGTGCGAGACATCAGGGAGAAGGAATTGAGGTTGTACACCGATGCTGGACGTGTTTGTCGACCACTCTTCATCGTCGAGAACCAACAGCTGGCACTTCAGAAGAAGCACGTCAAATGGCTCAGTAACGGCCTCAACGATGATGGCGACGATTACAAATGGGAACATCTGGTAAAGGGTGGCATCATTGAGTTACTGGATGCCGAGGAAGAGGAAACAGTGATGATTTCCATGACCCCTGAAGATCTTGAAAATTCTCGCCTACAGCAAAGTGGTGTTGATCCTCACGCGAACGACGGCGAGTTTGATCCAGCAGCTCGATTGAAAGCTGGCACTCACGCACACACATGGACACATTGCGAAATTCATCCTAGCATGATTCTTGGCATTTGTGCCAGCATTATTCCTTTCCCTGATCATCTGCGCCATTCTCATCATTGCTGGTGGTACTGGTGAGTTCGAGGCTGGTA-CTCCAAGGATGGCCAGACCCGCGAGCACGCTCTCCTCGCTTTCACCCTCGGCGTGCGGCAACTCATCGTTGCCGTCAACAAGATGGACACCACTAAGGTAAGAAACGTTTCAC-TTATAGTTAGGCTTCTTGCACATTCTCATCATCTCA-TGTAGTGGAGCGAGGACCGTTTCAACGAAATCATCAAGGAGACGTCAACCTTCATCAAGAAGGTTGGTTACAACCCCAAGGCCGTTGCCTTTGTTCCTATTTCCGGCTGGCACGGTGACAACATGTTGGAGGAGTCGGCTAAGTAAGGATTC--TCGTATCGTCATGATCGCCCCGTCGCTTATGTTGCACTCAAGCATGCCTTGGTACAAGGGCTGGACCAGGGAGACCAAGGCTGGTCCCGTCAAGGGCAAGACCCTCCTCGATGCTATTGATGCCATTGAACCCCCCGTCCGACCTTCCGACAAACCCCTCCGTCTCCCCCTCCAAGATGTCTACAAAATTGGCGGTATCGGAACTGTGCCCGTCGGTCGTGTTGAGACTGGTATCATCAAGGCCGGAATGGTCGTCACTTTCGCTCCCTCCAACGTCACCACTGAAGTCAAGTCTGTCGAAATGCATCACGAACAGCTCGAGCAGGGTAACCCTGGTGACAATGTCGGTTTCAACGTCAAGAACGTGTCAGTGAAGGATATTCGTCGTGGCAACGTGGCCTCCGACTCCAAGAACGATCCCGCCAAGGAAGCGGCCTCTTTCAACGCACAGGTCATCGTCCTCAACCACCCTGGTCAAATTGGTGCTGGTTACGCCCCAGTTCTCGACTGTCACACCGCCCACATCGCCTGCAAGTTCGCCGAGCTCATCGAGAAGATCGATCGTCGTACGGGTAAATCCATTGAAAACTCGCCTAAATTCGTCAAGTCGGGTGACGCCTGCATCGTCAAGCTCGTTCCCAGCAAGCCTATG

>F1123584_Laccaria_ochropurpurea_USA_IL_F1123584_F1123584__

CCTGA-TGTGGCTGTTAGCTGGC-TTTTTGAAGCATGTGCTCGT-CCGTCGTCTTT-ATCTCT--CCA-CCTGTGCACATTTTGTAGTC-TT-GGATACCTCTCGAGGCAA-CTCGGATT--TTTAGG-GATCGCCGTGC---TGT---ACAAGTCGGCTTTCTTTTCATTTCC-AAGACTATGTTTT----T--A-TATACA-CCAAAGTATGTTTAAAGAATGTCATA-AAAAGGAAC---TTGATTCCTA--T-AAAATTATACAACTTTCAGCAACGGATCTCTTGGCTCTCGCATCGATGAAGAACGCAGCGAAATGCGATAAGTAATGTGAATTGCAGAATTCAGTGAATCATCGAATCTTTGAACGCACCTTGCGCTCCTTGGTATTCCGAGGAGCATGCCTGTTTGAGTGTCATTAAA-TTCTCAA-CCTTCCAGC--------T-TTTATTAGCTTGGTTTTAGGCTTGGA-TGT-GGGGG--TTGCAGGCTTC-AT-----T---AATGAGGTTAGCTCTCCTTAAATGCATTAGCAGAACTTTTGTGGACTGTCTA-TTGGTGTGAT-AATTATCTACGCCGTGGAT-GTGAAGCAG-ATTTAT---GAAGTT-CAGCTTCT-AACTGTCC---ATTGACTTGGACAA-----TTTTGACAA-TTTGAAAGCTCAAATTTAAAATCTGGCAGT-CTTTGGCTGT-CCGAGTTGTAATCTAGAGAAGTATTATCCGCGCTGGACCGTGTACAAGTCTCCTGGAATGGGGCGTCATAGAGGGTGAGAATCCCGTCTTTGACACGGACTGCCAGGGC-TTTGTGATGTGCTCTCAAAGAGTCGAGTTGTTTGGGAATGCAGCTCAAAATGGGTGGTAAATTCCATCTAAAGCTAAATATTGGCGAGAGACCGATAGCGAACAAGTACCGTGAGGGAAAGATGAAAAGAACTTTGGAAAGAGAGTTAAACAGTACGTGAAATTGCTGAAAGGGAAACGCTTGAAGTCAGTCGCGTTGGCCAGGGATCAA-CCTTGC----TTTTTT---GCTTGGCTTACTTTCTGGTCGATGGG-TCAGCATCAATTTTGACTGGTGG-AAAAAGTTCAAGGGAATGTGGCAT-CTTCGGATGTGTTATAGCCTTTGTTCGCATACATTGGTTGGGATTGAGGAACTCAGCACGCCGCAAGGCCGGG---TTTTTAACCACGTACGTGCTTAGGATGCTGGCATAATGGCTTTAATCGACCCGTCTTGAAACACGGACCAAGGAGTCTAACATGCCTGCGAGTGTTTGGGTGAAAAACCCGAGCGCGTAATGAAAGTG-AAAGTTGAGATCCCTGTCGTGGGGAGCATTGACGCCCGGATCTGACCTTTTGTGACGATTCTGCGGTAGAGCATGT-----------------------------------------------------------------------------------------------------------------------------------------------------------------------------------------------------------------------------------------------------------------------------------------------------------------------------------------------------------------------------------------------------------------------------------------------------------------------------------------------------------------------------------------------------------------------------------------------------------------------------------------------------------------------------------------------------------------------------------------------------------------------------------------------------------------------------------------------------------------------------------------------------------------------------------------------------------------------------------------------------------------------------------------------------------------------------------------------------------------------------------------------------------------------------------------------------------------------------------------------------------------------------------------------------------------------------------------------------------------------------------------------------------------------------------------------------------------------------------------------------------------------------------------------------------------------------------------------------------------------------------------------------------------------------------------------------------------------------------------------------------------------------------------------------------------------------------------------------------------------------------------------------------------------------------------------------------------------------------------------------------------------------------------------------------------------------------------------------------------------------------------------------------------

>F1121451_Laccaria_laccata_CHINA_JX504128_JX504208__

-CTGA-TGTGACTGTTAGCTGGC-TTTTCGAAGCATGTGCTCGT-CCGTCATCTTTAATCTCT--CCA-CCTGTGCACATTTTGTAGTC-TT-GGATACCTCTCGAGGCAA-CTCGGATT----TTAG-GATCGCCGTGC---TGT---ACAAGTCGACTTTTCTTTCATTTCC-AAGACTATGTTTT----T--A-TATACA-CCAAAGTATGTTTAAAGAATGTCATC-AATAGGAAC---TTGTTTCCTA--T-AAAATTATACAACTTTCAGCAACGGATCTCTTGGCTCTCGCATCGATGAAGAACGCAGCGAAATGCGATAAGTAATGTGAATTGCAGAATTCAGTGAATCATCGAATCTTTGAACGCACCTTGCGCTCCTTGGTATTCCGAGGAGCATGCCTGTTTGAGTGTCATTAAA-TTCTCAA-CCTTCCATS--------T-TTTATTAGCATGG--TTAGGCTTGGA-TGT-GGGGG--TTGCGGGCTTC-AT-----C---AATGATGTCAGCTCTCCTTAAATGCATTAGCGGAACTTTTGTGGACCGTCTA-TTGGTGTGAT-AGTTATCTACGCCGTGGAT-GTGATGCAG-CTTTAT---GAAGTT-CAGCTTCT-AACCGTCC---ATTGACTTGGACAA-----TTTTGACAA-TTTGAAAGCTCAAATTTAAAATCTGGCAGT-CTTTGGCTGT-CCGAGTTGTAATCTAGAGAAGTATTATCCGCGCTGGACCGTGTACAAGTCTCCTGGAATGGAGCGTCATAGAGGGTGAGAATCCCGTCTTTGACACGGACTGCCAGGGC-TTTGTGATGTGCTCTCGAAGAGTCGAGTTGTTTGGGAATGCAGCTCAAAATGGGTGGTAAATTCCATCTAAAGCTAAATATTGGCGAGAGACCGATAGCGAACAAGTACCGTGAGGGAAAGATGAAAAGAACTTTGGAAAGAGAGTTAAACAGTACGTGAAATTGCTGAAAGGGAAACGCTTGAAGTCAGTCGCGTTGGCCAGGGATCAA-CCTTGC----TTTTTT---GCTTGGCTTACTTTCTGGTCGATGGG-TCAGCATCAATTTTGACTGGTAG-AAAAAGTTCAAGGGAATGTGGCAT-CTTCGGATGTGTTATAGCCCTTGTTCACATATATCAGTTGGAATTGAGGAACTCAGCACGCCGCAAGGCCGGG---TTTTTAACCACGTACGTGCTTAGGATGCTGGCATAATGGCTTTAATCGACCCGTCTTGAAACACGGACCAAGGAGTCTAACATGCCTGCGAGTGTTTGAGTGAAAAACCCGAGCGCATAATGAAAGTG-AAAGTTGAGATCCCTGTCATGGGGAGCATTGACGCCCGGATCTGACCTTTTGTGACGATTCTGCGGTAGGGCATGT-----------------------------------------------------------------------------------------------------------------------------------------------------------------------------------------------------------------------------------------------------------------------------------------------------------------------------------------------------------------------------------------------------------------------------------------------------------------------------------------------------------------------------------------------------------------------------------------------------------------------------------------------------------------------------------------------------------------------------------------------------------------------------------------------------------------------------------------------------------------------------------------------------------------------------------------------------------------------------------------------------------------------------------------------------------------------------------------------------------------------------------------------------------------------------------------------------------------------------------------------------------------------------------------------------------------------------------------------------------------------------------------------------------------------------------------------------------------------------------------------------------------------------------------------------------------------------------------------------------------------------------------------------------------------------------------------------------------------------------------------------------------------------------------------------------------------------------------------------------------------------------------------------------------------------------------------------------------------------------------------------------------------------------------------------------------------------------------------------------------------------------------------------------------

>AWW585_Laccaria_bicolor_USA_OR_JX504111_JX504194__

CCTGA-TGTGATTGTTAGCTGGC-TTTTCGAAGCATGTGCTCAT-CCGTCATCTTT-ATCTCT--CCA-CCTGTGCACATTTTGTAGTC-TT-GGATACCTCTCGAGGAAA-CTCGGATT----TTAG-AATCGCTGTGC---TGT---ACAAGTCGGCTTTTCTTTCATTTCC-AAGACTATGTTTT----T--A-TATACA-CCAAAGTATGTTTATAGAATGTCATC-AATGGGAAC---TTGTTTCCTA--T-AAAATTATACAACTTTCAGCAACGGATCTCTTGGCTCTCGCATCGATGAAGAACGCAGCGAAATGCGATAAGTAATGTGAATTGCAGAATTCAGTGAATCATCGAATCTTTGAACGCACCTTGCGCTCCTTGGTATTCCGAGGAGCATGCCTGTTTGAGTGTCATTAAA-TTCTCAA-CCTTCCAAC--------T-TTTATTAGCTTGG--TTAGGCTTGGA-TGT-GGGGG--TTGCGGGCTTG-AT-----T---AATGAGGTCGGCTCTCCTTAAATGCATTAGCGGAACTTTTGTGGACCGTCTA-TTGGTGTGAT-AATTATCTACGCCGTGGAT-GTGAAGCAG-CTTTAT---GAAGTT-CTGCTTCT-AACCGTCC---ATTGACTTGGACAA-----TTTTGATAA--TTGAAAGCTCAAATTTAAAATCTGGCAGT-CTTTGGCTGT-CCGAGTTGTAATCTAGAGAAGTATTATCCGCGCTGGACCGTGTACAAGTCTCCTGGAATGGAGCGTCATAGAGGGTGAGAATCCCGTCTTTGACACGGACTGCCAGGGC-TTTGTGATATGCTCTCRAAGAGTCGAGTTGTTTGGGAATGCAGCTCAAAATGGGTGGTAAATTCCATCTAAAGCTAAATATTGGCGAGAGACCGATAGCGAACAAGTACCGTGAGGGAAAGATGAAAAGAACTTTGGAAAGAGAGTTAAACAGTACGTGAAATTGCTGAAAGGGAAACGCTTGAAGTCAGTCGCGTTGGCCAGGGATCAA-CCTTGC----TTTTTT---GCTTGGCTTACTTTCTGGTTGATGGG-TCAGCATCAATTTTGACCGGTGG-AAAAAGTTCAAAGGAATGTGGCAT-CTTCGGATGTGTTATAGCCTTTGTTCACATACATTGGTTGGGATTGAGGAACTCAGCACGCCGCAAGGCCGGG---TTTTTAACCACGTCCGTGCTTAGGATGCTGGCATAATGGCTTTAATCGACCCGTCTTGAAACACGGACCAAGGAGTCTAACATGCCTGCGAGTATTTGGGTGAAAAACCCGAGTGCGTAATGAAAGTG-AAAGTTGAGATCCCTGTCGTGGGGAGCATTGACGCCCGGATCTGACCTTTTGTGACGATTCTGCGGTAGAGCATGT-----------------------------------------------------------------------------------------------------------------------------------------------------------------------------------------------------------------------------------------------------------------------------------------------------------------------------------------------------------------------------------------------------------------------------------------------------------------------------------------------------------------------------------------------------------------------------------------------------------------------------------------------------------------------------------------------------------------------------------------------------------------------------------------------------------------------------------------------------------------------------------------------------------------------------------------------------------------------------------------------------------------------------------------------------------------------------------------------------------------------------------------------------------------------------------------------------------------------------------------------------------------------------------------------------------------------------------------------------------------------------------------------------------------------------------------------------------------------------------------------------------------------------------------------------------------------------------------------------------------------------------------------------------------------------------------------------------------------------------------------------------------------------------------------------------------------------------------------------------------------------------------------------------------------------------------------------------------------------------------------------------------------------------------------------------------------------------------------------------------------------------------------------------------

>GMM7607_Laccaria_bicolorproxima_FRANCE_KM067846_28S_RPB2_EF1a

CCTGA-TGTGGCTGTTAGCTGGC-TTTTCGAAGCATGTGCTCGT-CCATCATCTTT-ATCT----CCA-CCTGTGCACATTTTGTAGTC-TT-GGATACCTCTCGAGGCAA-CTCGGATT----TTAG-GATCGCTGTGC---TGT---ACAAGTCGGCTTTCCTTTCATTTCC-AAGACTACGTTTT----T--A-TACACA-CTAAAGTATGTTTAAAGAATGTCATC-AATGGGAAC---TTGTTTCCTA--T-AAAATTATACAACTTTCAGCAACGGATCTCTTGGCTCTCGCATCGATGAAGAACGCAGCGAAATGCGATAAGTAATGTGAATTGCAGAATTCAGTGAATCATCGAATCTTTGAACGCACCTTGCGCTCCTTGGTATTCCGAGGAGCATGCCTGTTTGAGTGTCATTAAA-TTCTCAA-CCTTCCAGC--------T-TTTATTAGCTTGG--ATAGGCTTGGA-TGT-GGGGG--TTGCGGGCTTC-AT-----C---AATGAGGTTGGCTCTCCTTAAAAGCATTAGCGGAACTTTTGTGGACCGTCTA-TTGGTGTGAT-AATTATCTACGCCGTGGAT-GTGAAGCAA-CTTTAT---AAAGTT-CAGCTTCT-AACTGTCC---ATTAACTTGGACAA-----TTTTGACAA-TTTGGAAGCTCAAATTTAAAATCTGGCAGT-CTTTGGCTGT-CCGAGTTGTAATCTAGAGAAGTATTATCCGCGCTGGACCGTGTACAAGTCTCCTGGAATGGAGCGTCATAGAGGGTGAGAATCCCGTCTTTGACACGGACTGCCAGGGC-TTTGTGATGTGCTCTCAAAGAGTCGAGTTGTTTGGGAATGCAGCTCAAAATGGGTGGTAAATTCCATCTAAAGCTAAATATTGGCGAGAGACCGATAGCGAACAAGTACCGTGAGGGAAAGATGAAAAGAACTTTGGAAAGAGAGTTAAACAGTACGTGAAATTGCTGAAAGGGAAACGCTTGAAGTCAGTCGCGTTGGCCAGGGATCAA-CCTTGC----TTTTTT---GCTTGGCTTACTTTCTGGTTGATGGG-TCAGCATCAATTTTGACTGGTGG-AAAAAGTTCAAGGGAATGTGGCAT-CTTCTGATGTGTTATAGCCTTTGTTCGCATACATCGGTTGGGATTGAGGAACTCAGCACGCCGCAAGGCCGGG---TTTTTAACCACGTACGTGCTTAGGATGCTGGCATAATGGCTTTAATCGACCCGTCTTGAAACACGGACCAAGGAGTCTAACATGCCTGCGAGTGTTTGGGTGAAAAACTCGAGCGCGTAATGAAAGTG-AAAGTTGAGATCCCTGTCATGGGGAGCATTGACGCCCGGATCTGACCTTTTGTGACGATTCTGCGGTAGAGCATGTTTTCCGCATGCTTTTCCGAAAGTTGACCAAGGACGTTTATCGTTATCTACAAAAGgttgctgaccttcgttcaagtgcc-----atgagatctgctga-cccttct---ttttcagTGCGTCGAGGCCCACAAGGAGTTCAACCTTGCCCTTGCAGTTAAGCATCAAACCATCACAAACGGCCTCAAATACTCTCTGGCGACAGGTAACTGGGGAGATCAGAAAAAGTCAATGTCTTCCAAAGCAGGAGTATCTCAGGTTTTGAACAGATACACATATGCATCCACTCTGTCACATCTTCGTCGGTGTAACACTCCTCTAGGGCGYGAAGGCAAGATCGCCAAACCTCGTCAGTTGCACAACACTCATTGGGGTATGGTGTGTCCTGCGGAAACCCCTGAAGGTCAAGCCTGTGGTCTTGTCAAGAACCTCGCCCTTATGGCATGCATATCAGTCGGGTCTTACTCCGCGCCCGTTATCGAGTTTTTGGAGGAGTGGGGATTGGAGTCTTTGGAAGAGAACGCACACTCATCGACACCTTGCACAAAAGTTTTCGTGAATGGTGTCTGGATGGGTGTGCATCGCGATCCTGCCAATTTGGTGAAGACGATAAAGAAATTGAGAAGGAAAGACGACATCAGCCCAGAAGTGTCAGTTGTGCGAGACATCAGGGAGAAGGAATTGAGGTTGTACACTGATGCTGGACGTGTTTGTCGACCACTCTTCATCGTCGAGAACCAACAGCTGGCACTTCAGAAGAAGCACGTCAAATGGCTCAGTAACGGCCTCAACGATGATGGCGACGAGTACAAATGGGAACATCTGGTAAAGGGTGGCATCATTGAGTTACTGGATGCTGAGGAAGAGGAAACAGTGATGATATCCATGACCCCTGAAGATCTTGAAAATTCTCGCCTACAGCAAAGTGGTGTTGATCCTCACGCGAACGACGGCGAGTTTGATCCAGCAGCTCGATTGAAGGCTGGCACTCACGCACACACGTGGACACATTGCGAAATTCATCCTAGCATGATTCTTGGCATTTGTGCAAGCATTATTCC-TTCCCTGATCATCTGCGCCATTCTCATCATTGCTGGTGGTACTGGTGAGTTCGAGGCTGGTATATCTAAGGATGGCCAGACCCGCGAACACGCTCTCCTCGCTTTCACCCTTGGTGTGCGGCAACTCATCGTTGCCGTCAACAAGATGGACACCACTAAGGTAAGAAACGTTTCAC-CTGTAGTTAGGCTCCTTCCAGATTCTCATCATCTCA-TGTAGTGGAGCGAGGACCGTTTCAACGAAATCATCAAGGAGACGTCAACCTTCATCAAGAAGGTTGGTTACAACCCCAAGGCCGTTGCCTTTGTCCCTATTTCCGGCTGGCACGGTGACAACATGTTGGAGGAGTCCTCTAAGTAAGCATTC--TCGTATTGTCATGATTGCCCCGTCACTCATGTTGCACCC-AGCATGCCTTGGTACAAGGGCTGGACCAAGGAGACCAAGGCTGGTGTCGTCAAGGGCAAGACCCTCCTCGATGCTATTGATGCCATTGAACCCCCCGTCCGACCTTCCGACAAACCCCTCCGTCTCCCCCTCCAGGATGTCTACAAAATTGGCGGTATCGGAACTGTGCCCGTCGGTCGTGTTGAGACTGGTATCATCAAGGCCGGAATGGTCGTCACTTTTGCTCCCTCCAACGTCACCACTGAAGTCAAGTCCGTCGAAATGCATCACGAACAGCTTGAGCAGGGTAACCCTGGTGACAATGTCGGTTTCAACGTCAAGAACGTGTCAGTGAAGGATATTCGTCGTGGCAACGTGGCCTCCGACTCCAAGAACGATCCCGCCAAGGAAGCGGCCTCTTTCAACGCACAGGTCATCGTCCTCAACCACCCTGGTCAAATTGGTGCTGGTTACGCCCCGGTTCTCGACTGTCACACCGCCCACATCGCCTGCAAGTTCGCCGAGCTCATCGAGAAGATCGATCGTCGTACGGGTAAATCCATTGAAAACTCGCCTAAATTCGTCAAGTCGGGTGACGCCTGCATCGTCAAGCTCGTTCCCAGCAAGCCTATG

>GMM7612_Laccaria_macrocystidia_FRANCE_KM067847_KU685861_KU686002_KU686122

CCTGA-TGTGACTGTTAGCTGGC-TTTTCGAAGCATGTGCTCGT-CCGTCATCTTTAATCTCT--CCA-CCTGTGCACATTTTGTAGTC-TT-GGATACCTCTCGAGGCAA-CTCGGATT----TTAG-GATCGCCGTGC---TGC---ACAAGTCGGCTTTCCTTTCATTTCC-AAGACTATGTTTT----T--A-TATACA-CCAAAGTATGTTTAAAGAATGTCATC-AATAGGAAC---TTGTTTCCTA--T-AAAATTATACAACTTTCAGCAACGGATCTCTTGGCTCTCGCATCGATGAAGAACGCAGCGAAATGCGATAAGTAATGTGAATTGCAGAATTCAGTGAATCATCGAATCTTTGAACGCACCTTGCGCTCCTTGGTATTCCGAGGAGCATGCCTGTTTGAGTGTCATTAAA-TTCTCAA-CCTTCCAGC--------T-TTTATTAGCATGG--TTAGGCTTGGA-TGT-GGGGG--TTGTGGGCTTC-AT-----C---AATGAGGTCGGCTCTCCTTAAATGCATTAGCGGAACTTTTGTGGACCGTCTA-TTGGTGTGAT-AATTATCTACGCTGTGGAT-GTGAAGCAG-ATTTAT---GAAGTT-CAGCTTCT-AACTGTCC---ATTGACTTGGACAA-----TTTTGACAA-TTTGAAAGCTCAAATTTAAAATCTGGCAGT-CTTTGGCTGT-CCGAGTTGTAATCTAGAGAAGTATTATCCGCGCTGGACCGTGTACAAGTCTCCTGGAATGGAGCGTCATAGAGGGTGAGAATCCCGTCTTTGACACGGACTGCCAGGGCTTT-GTGATGTGCTCTCGAAGAGTCGAGTTGTTTGGGAATGCAGCTCAAAATGGGTGGTAAATTCCATCTAAAGCTAAATATTGGCGAGAGACCGATAGCGAACAAGTACCGTGAGGGAAAGATGAAAAGAACTTTGGAAAGAGAGTTAAACAGTACGTGAAATTGCTGAAAGGGAAACGCTTGAAGTCAGTCGCGTTGGCCAGGGATCAA-CCTTGC----TTTTTT---GCTTGGCTTACTTTCTGGTTGATGGG-TCAGCATCAATTTTGACTGGTGG-AAAAAGTTCAAGGGAATGTGGCAT-CTTCGGATGTGTTATAGCCTTTGTTCACATATATCAGTTGGGATTGAGGAACTCAGCACGCCGCAAGGCCGGG---TTTTTAACCACGTACGTGCTTAGGATGCTGGCATAATGGCTTTAATCGACCCGTCTTGAAACACGGACCAAGGAGTCTAACATGCCTGCGAGTGTTTGGGTGAAAAACCCGAGCGCGTAATGAAAGTG-AAAGTTGAGATCCCTGTCATGGGGAGCATTGACGCCCGGATCTGACCTTTTGTGACGATTCTGCGGTAGAGCATGTTTTCCGCATGCTTTTCCGAAAGTTGACTAAGGACGTTTATCGTTATCTACAAAAGgttgctgaccttcgctcgagtgcc-----ttgagatatgctga-cccctct---tctttagTGCGTCGAGACCCACAAGGAGTTCAACCTTGCCCTTGCAGTCAAGCATCAAACCATCACAAATGGTCTCAAATACTCTCTGGCGACAGGTAACTGGGGAGATCAGAAAAAGTCAATGTCTTCCAAGGCAGGAGTATCTCAGGTCTTGAACAGATACACATATGCATCCACTCTGTCACATCTTCGTCGGTGTAACACTCCTCTAGGGCGCGAAGGCAAGATCGCCAAACCTCGTCAGTTGCACAACACTCATTGGGGTATGGTGTGTCCTGCGGAAACTCCTGAAGGTCAAGCCTGTGGTCTTGTCAAGAACCTCGCCCTTATGGCGTGCATATCAGTCGGGTCTTACTCCGCGCCCGTTATCGAGTTTTTGGAGGAGTGGGGATTGGAGTCATTGGAAGAGAACGCGCACTCATCAACACCTTGCACAAAAGTTTTCGTGAACGGTGTCTGGATGGGTGTGCATCGCGATCCTGCCAATTTGGTGAAGACGATAAAGAAATTGAGAAGGAAAGACGACATTAGCCCAGAAGTGTCAGTCGTGCGAGACATTAGGGAGAAGGAATTGAGGTTGTACACCGATGCTGGACGTGTTTGTCGACCACTCTTCATCGTCGAGAACCAACAGCTGGCACTTCAGAAGAAGCACGTCAAATGGCTCAGTAACGGCCTCAACGATGATGGCGATGAGTACAAATGGGAGCATCTGGTAAAGGGTGGCATCATTGAGTTACTGGATGCTGAGGAAGAGGAAACAGTGATGATATCCATGACCCCTGAAGATCTTGAAAATTCTCGCCTACAGCAAAGTGGTGTTGATCCTCACGCGAACGACGGCGAGTTTGATCCAGCAGCTCGATTGAAGGCTGGCACTCACGCACACACATGGACACATTGCGAAATTCATCCTAGCATGATTCTTGGCATTTGTGCCAGCATTATTCCTTTCCCTGATCATCTGCGCCATTCTCATCATTGCTGGTGGTACTGGTGAGTTCGAGGCTGGCATCTCCAAGGATGGCCAGACCCGCGAGCACGCTCTCCTCGCTTTCACCCTTGGTGTGCGGCAACTCATCGTTGCCGTCAACAAGATGGACACCACTAAGGKAAGAAACGTTTCAC-TTATAGTTAGGCTTCTTGCACATTCTCATCATCTCA-TGTAGTGGAGCGAGGACCGTTTCAACGAAATCATCAAGGAGACGTCAACCTTCATCAAGAAGGTTGGTTACAACCCCAAGGCCGTTGCCTTTGTCCCTATTTCCGGCTGGCACGGTGACAACGTGTTGGAGGAGTCCGCTAAGTGAGCATTC--TCGTATCGTCATGACCGCCCCGTCGCTCATGTTGCACTCAAGCATGCCTTGGTACAAGGGCTGGACCAGGGAGACCAAGGCTGGTCCCGTCAAGGGCAAGACCCTCCTCGATGCTATCGATGCCATTGAACCCCCTGTCCGACCTTCCGACAAACCCCTCCGTCTCCCCCTTCAGGATGTCTACAAAATTGGTGGTATCGGAACTGTGCCCGTCGGTCGTGTTGAGACTGGTATCATCAAGTCCGGAATGGTCGTCACTTTCGCCCCCTCCAACGTCACCACTGAAGTTAAGTCCGTCGAAATGCATCACGAACAGCTCGAGCAGGGTAACCCCGGTGACAATGTCGGTTTCAACGTCAAGAACGTGTCAGTGAAGGATATTCGTCG-GGCAACGTGGCCTCCGACTCCAAGAACGATCCCGCCAAGGAAGCGGCCTCTTTCAACGCACAGGTCATCGTCCTCAACCACCCTGGTCAAATTGGTGCTGGTTACGCCCCGGTTCTCGACTGTCACACCGCCCACATCGCCTGCAAGTTCGCCGAGCTCATCGAGAAGATCGATCGTCGTACGGGTAAATCCATCGAAAATTCGCCTAAATTTGTCAAGTCGGGTGACGCCTGCATCGTCAAGCTTGTCCCCAGCAAGCCTATG

>GMM7620_Laccaria_sp_FRANCE_KM067854_28S_RPB2_EF1a

CCTGA-TGTGATTGTTAGCTGGC-TTTTCGAAGCATGTGCTCGT-CCGTCATCTTT-ATCTCT--CCA-CCTGTGCACATTTTGTAGTC-TT-GGATACCTCTCGAGGAAA-CTCGGATT----TTAG-GATCGCTGTGC---TGT---ACAAGTCGGCTTTTCTTTCATTTCC-AAGACTATGTTTT--TAT--A-TATACA-CCAAAGTATGTTTATAGAATGTCATC-AATGGGAAC---TTGTTTCCTA--T-AAAATTATACAACTTTCAGCAACGGATCTCTTGGCTCTCGCATCGATGAAGAACGCAGCGAAATGCGATAAGTAATGTGAATTGCAGAATTCAGTGAATCATCGAATCTTTGAACGCACCTTGCGCTCCTTGGTATTCCGAGGAGCATGCCTGTTTGAGTGTCATTAAA-TTCTCAA-CCTTCCAAC--------T-TTTATTAGCTTGG--TTAGGCTTGGA-TGT-GGGGG--TTGCGGGCTTC-AT-----T---AATGAGGTCGGCTCTCCTTAAATGCATTAGCGGAACTTTTGTGGACCGTCTA-TTGGTGTGAT-AATTATCTACGCCGTGGAT-GTGAAGCAG-CTTTAT---GAAGTT-CTGCTTCT-AACCGTCC---ATTGACTTGGACAA----TTTTTGACAA-TTTGAAAGCTCAAATTTAAAATCTGGCAGT-CTTTGGCTGT-CCGAGTTGTAATCTAGAGAAGTATTATCCGCGCTGGACCGTGTACAAGTCTCCTGGAATGGAGCGTCATAGAGGGTGAGAATCCCGTCTTTGACACGGACTGCCAGGGC-TTTGTGATATGCTCTCAAAGAGTCGAGTTGTTTGGGAATGCAGCTCAAAATGGGTGGTAAATTCCATCTAAAGCTAAATATTGGCGAGAGACCGATAGCGAACAAGTACCGTGAGGGAAAGATGAAAAGAACTTTGGAAAGAGAGTTAAACAGTACGTGAAATTGCTGAAAGGGAAACGCTTGAAGTCAGTCGCGTTGGCCAGGGATCAA-CCTTGC----TTTTTT---GCTTGGCTTACTTTCTGGTCGATGGG-TCAGCATCAATTTTGACCGGTGG-AAAAAGTTCAAAGGAATGTGGCAT-CTTCGGATGTGTTATAGCCTTTGTTCACATACATTGGTTGGGATTGAGGAACTCAGCACGCCGCAAGGCCGGG---TTTTTAACCACGTACGTGCTTAGGATGCTGGCATAATGGCTTTAATCGACCCGTCTTGAAACACGGACCAAGGAGTCTAACATGCCTGCGAGTATTTGGGTGAAAAACCCGAGTGCGTAATGAAAGTG-AAAGTTGAGATCCCTGTCGTGGGGAGCATTGACGCCCGGATCTGACCTTTTGTGACGATTCTGCGGTAGAGCATGTTTTCCGCATGCTTTTCCGAAAGTTGACCAAGGACGTTTATCGTTATCTACAAAAGgtttctgactttcgtttaagtgct-----atgagacgtattga-cctctct---tcttcagTGCGTCGAGACCCATAAGGAGTTCAACCTTGCCCTTGCAGTTAAGCATCAAACCATCACAAACGGCCTCAAATACTCTCTGGCGACAGGTAACTGGGGGGATCAGAAAAAGTCAATGTCTTCCAAGGCAGGAGTATCTCAGGTCTTGAACAGATACACATATGCATCCACTCTGTCACATCTTCGTCGGTGTAACACTCCTCTAGGGCGCGAAGGCAAGATCGCCAAACCTCGTCAGTTGCACAACACCCATTGGGGTATGGTGTGCCCTGCGGAAACTCCTGAAGGTCAAGCCTGTGGTCTTGTCAAGAACCTCGCCCTTATGGCGTGCATATCAGTCGGGTCTTACTCCGCGCCCGTCATCGAGTTTTTGGAGGAATGGGGATTGGAGTCATTGGAAGAGAACGCGCACTCATCAACTCCCTGCACAAAAGTTTTCGTGAACGGTGTATGGATGGGTGTGCATCGCGATCCTGCCAACTTGGTGAAGACGATAAAGAAATTGAGAAGGAAAGACGACATTAGCCCTGAAGTGTCAGTTGTGCGAGACATCCGAGAGAAGGAATTGAGGTTGTACACCGATGCTGGACGTGTTTGTCGACCACTTTTCATCGTCGAGAACCAACAGCTGGCACTTCAGAAGAAGCACGTCAAATGGCTCAGTAACGGCCTCAACGATGATGGCGACGAATACAAATGGGAGCATCTGGTAAAGGGTGGCATCATTGAGTTACTGGATGCTGAGGAAGAGGAAACGGTGATGATATCCATGACTCCTGAAGATCTTGAAAATTCTCGCCTACAGCAAAGTGGCGTTGATCCTCACGCGAACGACGGCGAGTTTGATCCAGCAGCTCGATTAAAGGCTGGCACTCACGCACACACATGGACACATTGCGAAATTCATCCTAGCATGATTCTTGGCATTTGTGCCAGCATTATTCCTTTCCCCGATCATCTGCGCCATTCTCATCATTGCTGGTGGTACTGGTGAGTTTGAGGCTGGTATCTCCAAGGATGGCCAGACCCGCGAGCACGCTCTCCTCGCTTTCACCCTCGGTGTGCGGCAACTCATCGTTGCCGTCAACAAGATGGACACCACCAAGGTAAGAAAAGTTTCAC-TTATAGTCAGGCTTCTTCCACATGCTCATCACCTCAT-GCAGTGGAGCGAGGACCGTTTCAACGAAATTATCAAGGAGACATCAAGCTTCATCAAGAAGGTTGGTTACAACCCCAAGGCCGTTGCCTTTGTTCCTATTTCCGGCTGGCACGGTGACAACATGTTGGAGGAGTCCCCTAAGTAAGTATTC--TCGTATCGTCATTATCGCCCCGTCGCTCATGTCACACTC-AGCATGCCTTGGTTCAAGGGCTGGACCAAGGAGACCAAGGCTGGTGTCGTCAAGGGCAAGACCCTCCTCGATGCTATTGATGCCATTGAGCCCCCCGTCCGACCTTCCGACAAGCCCCTCCGTCTTCCCCTCCAGGACGTTTACAAAATTGGCGGTATCGGAACTGTGCCCGTCGGTCGTGTTGAGACTGGTATCATCAAGGCCGGAATGGTCGTCAATTTCGCTCCCTCCAACGTCACCACTGAAGTCAAGTCCGTCGAAATGCATCACGAACAGCTCGAGCAGGGTAACCCTGGTGACAATGTCGGTTTCAACGTCAAGAACGTGTCAGTGAAGGATATTCGTCGTGGCAACGTGGCCTCCGACTCCAAGAACGATCCCGCCAAGGAAGCGGCCTCTTTCAACGCACAGGTCATCGTCCTCAACCACCCTGGTCAAATTGGTGCTGGTTATGCCCCGGTTCTCGACTGTCACACCGCCCACATCGCCTGCAAGTTCGCCGAGCTCATCGAAAAGATCGATCGTCGTACGGGTAAATCCATTGAAAACTCGCCCAAATTCGTCAAGTCGGGTGACGCCTGCATCGTCAAGCTCGTTCCCAGCAAGCCTATG

>GMM7621_Laccaria_amethystina_FRANCE_JX504150_JX504224_KU686046_KU686152

CCTGA-TGTGGCTGTTAGCTGGC-TTTTCGAAGCATGTGCTCGT-CCGTCATCTTT-ATCTCT--CCA-CCTGTGCACATTTTGTAGTC-TT-GGATACCTCTCGAGGCAA-CTCGGATT----TTAG-GATCGCTGTGC---TGT---ACAAGTCGGCTTTCCTTTCATTTCC-AAGACTATGTTTT----T--A-TATACA-CCAAAGTATGTTTAAAGAATGTCATC-AATAGGAAC---TTGTTTCCTA--T-ATAATTATACAACTTTCAGCAACGGATCTCTTGGCTCTCGCATCGATGAAGAACGCAGCGAAATGCGATAAGTAATGTGAATTGCAGAATTCAGTGAATCATCGAATCTTTGAACGCACCTTGCGCTCCTTGGTATTCCGAGGAGCATGCCTGTTTGAGTGTCATTAAA-TTCTCAA-CCTTCCAGC-------TT-TTTATAAGCTTGG--TTAGGCTTGGA-TGT-GGGGG--TTGTGGGCTTC-AT-----T---AATGAGGTCAGCTCTCCTTAAATGCATTAGCGGAACTTTTGTGGACCGTCTA-TTGGTGTGAT-AATTATCTACGCCGTGGAT-GTGAAGCAG-ATTTAT---GAAGTT-CAGCTTCT-AACCGTCC---ATTGACTTGGACAA-----TTTTGACAA-TTTGAAAGCTCAAATTTAAAATCTGGCAGT-CTTTGGCTGT-CCGAGTTGTAATCTAGAGAAGTATTATCCGCGCTGGACCGTGTACAAGTCTCCTGGAATGGGGCGTCATAGAGGGTGAGAATCCCGTCTTTGACACGGACTGCCAGGGCTTT-GTGATGTGCTCTCAAAGAGTCGAGTTGTTTGGGAATGCAGCTCAAAATGGGTGGTAAATTCCATCTAAAGCTAAATATTGGCGAGAGACCGATAGCGAACAAGTACCGTGAGGGAAAGATGAAAAGAACTTTGGAAAGAGAGTTAAACAGTACGTGAAATTGCTGAAAGGGAAACGCTTGAAGTCAGTCGCGTTGGCCAGGGATCAA-CCTTGC----TTTTTT---GCTTGGCTTACTTTCTGGTCGATGGG-TCAGCATCAATTTTGACCGGTGG-AAAAAGTTCAAGGGAATGTGGCAT-CTTCGGATGTGTTATAGCCTTTGTTCGCATACATTGGTTGGGATTGAGGAACTCAGCACGCCGCAAGGCCGGG---TTTTTAACCACGTACGTGCTTAGGATGCTGGCATAATGGCTTTAATCGACCCGTCTTGAAACACGGACCAAGGAGTCTAACATGCCTGCGAGTGTTTGGGTGAAAAACCCGAGCGCGTAATGAAAGTG-AAAGTTGAGATCCCTGTCGTAGGGAGCATTGACGCCCGGATCTGACCTTTTGTGACGATTCTGCGGTAGAGCATGTTTTCCGCATGCTTTTCCGAAAGCTGTCCAAGGACGTTTATCGTTATCTACAAAAGgttgctgaccttcgtttaagtgcc-----atgagatgtgctga-cgcctaa---tcttcagTGTGTCGAGACCCACAAAGAGTTCAACCTTGCCCTTGCAGTTAAGCATCAAACCATCACAAACGGTCTCAAATACTCTCTGGCGACAGGTAACTGGGGAGATCAGAAAAAGTCAATGTCTTCCAAGGCAGGAGTATCTCAGGTCTTGAACAGATACACATATGCGTCCACTCTGTCACATCTTCGTCGGTGTAACACTCCTTTAGGGCGCGAAGGCAAGATCGCCAAACCTCGTCAGCTGCACAACACTCATTGGGGTATGGTGTGTCCTGCGGAAACTCCTGAAGGTCAAGCCTGTGGTCTTGTCAAGAACCTCGCCCTTATGGCGTGCATATCAGTCGGGTCTTACTCTGCGCCTGTTATCGAGTTTTTGGAGGAGTGGGGATTGGAGTCATTGGAAGAGAACGCACACTCATCGACACCTTGCACAAAAGTTTTCGTGAACGGTGTCTGGATGGGTGTGCATCGCGATCCTGCCAATTTGGTCAAGACGATAAAGAAACTGAGAAGGAAAGATGACATCAGCCCAGAAGTGTCAGTTGTGCGAGACATTAGGGAGAAGGAACTCAGGTTGTACACCGATGCTGGACGTGTTTGTCGACCACTCTTCATCGTCGAGAACCAACAGCTGGCACTTCAGAAGAAGCACGTCAAATGGCTCAGTAACGGCCTCAACGATGATGGCGACGAGTACAAATGGGAACATCTGGTAAAGGGTGGCATCATTGAGTTACTGGATGCTGAGGAAGAGGAAACGGTGATGATATCCATGACCCCTGAAGATCTTGAAAATTCTCGCCTACAGCAAAGTGGTGTTGACCCCCACGCGAACGACGGCGAGTTTGATCCAGCAGCTCGATTGAAGGCTGGCACTCACGCACACACATGGACTCATTGCGAAATTCATCCTAGCATGATTCTTGGCATTTGTGCCAGCATTATTCCTTTCCCTGATCATCTGCGCCATTCTCATCATTGCTGGTGGTACTGGTGAGTTCGAGGCTGGTATCTCCAAGGATGGCCAGACCCGCGAGCACGCTCTCCTCGCTTTCACCCTCGGTGTGCGGCAACTCATCGTGGCCGTCAACAAGATGGACACCACCAAGGTAAGAAACGTTTGAC-TTATAGTTAGGCTTCTTTCACATTTTCATCATCTCA-TGTAGTGGAGCGAGGACCGTTTCAACGAAATCATCAAGGAGACGTCAACCTTCATCAAGAAGGTTGGTTACAACCCCAAGGCCGTTGCCTTTGTCCCTATTTCCGGCTGGCACGGTGACAACATGTTGGAGGAGTCCGCTAAGTGAGCATTC--TCGTATCGTTATGGTCGCCCCGTSGCTCATGTTACATTC-AGCATGCCTTGGTACAAGGGCTGGACCAGGGAGACCAAGGCTGGTCCCGTCAAGGGCAAGACCCTCCTCGATGCTATTGATGCCATTGARCCCCCCGTCCGACCTTCCGACAAACCCCTCCGTCTCCCCCTCCAGGATGTCTACAAAATTGGCGGTATTGGAACTGTGCCCGTCGGTCGTGTTGAGACTGGTATCATCAAGGCCGGAATGGTCGTCACTTTCGCGCCCTCCAACGTCACCACTGAAGTCAAGTCCGTCGAAATGCATCACGAACAGCTCGAGCAGGGTAACCCTGGTGACAATGTCGGTTTCAACGTCAAGAACGTGTCAGTGAAGGATATTCGTCGTGGCAACGTGGCCTCCGACTCCAAGAACGATCCGGCCAAGGAAGCGGCCTCTTTCAACGCACAGGTCATCGTCCTCAACCACCCTGGTCAAATTGGTGCTGGTTACGCCCCAGTTCTCGACTGTCACACCGCCCACATCGCCTGCAAGTTYGCCGAGCTTATCGAGAAGATCGATCGTCGTACGGGTAAATCTATTGAAAATTCGCCTAAATTCGTCAAATCGGGTGACGCCTGCATCGTCAAGCTCGTTCCCAGCAAGCCTATG

>GMM7628_Laccaria_proxima?_FRANCE_KM067857_KU685867_KU686008_KU686127

CCTGA-TGTGACTGTTAGCTGGC-TTTTCGAAGCATGTGCTCGT-CCGTCATCTTTAATCTCT--CCA-CCTGTGCACATTTTGTAGTC-TT-GGATACCTCTCGAGGCAA-CTCGGATT----TTAG-GATCGCCGTGC---TGC---ACAAGTCGGCTTTCCTTTCATTTCC-AAGACTATGTTTT----T--A-TATACA-CCAAAGTATGTTTAAAGAATGTCATC-AATAGGAAC---TTGTTTCCTA--T-AAAATTATACAACTTTCAGCAACGGATCTCTTGGCTCTCGCATCGATGAAGAACGCAGCGAAATGCGATAAGTAATGTGAATTGCAGAATTCAGTGAATCATCGAATCTTTGAACGCACCTTGCGCTCCTTGGTATTCCGAGGAGCATGCCTGTTTGAGTGTCATTAAA-TTCTCAA-CCTTCCAGC--------T-TTTATTAGCATGG--TTAGGCTTGGA-TGT-GGGGG--TTGTGGGCTTC-AT-----C---AATGAGGTCGGCTCTCCTTAAATGCATTAGCGGAACTTTTGTGGACCGTCTA-TTGGTGTGAT-AATTATCTACGCTGTGGAT-GTGAAGCAG-ATTTAT---GAAGTT-CAGCTTCT-AACTGTCC---ATTGACTTGGACAA-----TTTTGACAA-TTTGAAAGCTCAAATTTAAAATCTGGCAGT-CTTTGGCTGT-CCGAGTTGTAATCTAGAGAAGTATTATCCGCGCTGGACCGTGTACAAGTCTCCTGGAATGGAGCGTCATAGAGGGTGAGAATCCCGTCTTTGACACGGACTGCCAGGGCTTT-GTGATGTGCTCTCGAAGAGTCGAGTTGTTTGGGAATGCAGCTCAAAATGGGTGGTAAATTCCATCTAAAGCTAAATATTGGCGAGAGACCGATAGCGAACAAGTACCGTGAGGGAAAGATGAAAAGAACTTTGGAAAGAGAGTTAAACAGTACGTGAAATTGCTGAAAGGGAAACGCTTGAAGTCAGTCGCGTTGGCCAGGGATCAA-CCTTGC----TTTTTT---GCTTGGCTTACTTTCTGGTTGATGGG-TCAGCATCAATTTTGACTGGTGG-AAAAAGTTCAAGGGAATGTGGCAT-CTTCGGATGTGTTATAGCCTTTGTTCACATATATCAGTTGGGATTGAGGAACTCAGCACGCCGCAAGGCCGGG---TTTTTAACCACGTACGTGCTTAGGATGCTGGCATAATGGCTTTAATCGACCCGTCTTGAAACACGGACCAAGGAGTCTAACATGCCTGCGAGTGTTTGGGTGAAAAACCCGAGCGCGTAATGAAAGTG-AAAGTTGAGATCCCTGTCATGGGGAGCATTGACGCCCGGATCTGACCTTTTGTGACGATTCTGCGGTAGAGCATGTTTTCGGCATGCTTTTCCGAAAGTTGACTAAGGACGTTTATCGTTATCTACAAAAAgttgctgaccttcgctcgagtgcc-----ttgagatatgctga-cccctct---tctttagTGCGTCGAGACCCACAAGGAGTTCAACCTTGCCCTTGCAGTCAAGCATCAAACCATCACAAATGGCCTCAAATACTCTCTGGCGACAGGTAACTGGGGAGATCAGAAAAAGTCAATGTCTTCCAAGGCAGGAGTATCTCAGGTCTTGAACAGATACACTTATGCATCCACTCTGTCACATCTTCGTCGGTGTAACACTCCTCTAGGGCGCGAAGGCAAGATCGCCAAACCTCGTCAGTTGCACAACACTCATTGGGGTATGGTGTGTCCTGCGGAAACTCCTGAAGGTCAAGCCTGTGGTCTTGTCAAGAACCTCGCCCTTATGGCGTGCATATCAGTCGGGTCTTACTCCGCGCCCGTTATCGAGTTTTTGGAGGAGTGGGGATTGGAGTCATTGGAAGAGAACGCGCACTCATCAACACCTTGCACAAAAGTTTTCGTGAACGGTGTCTGGATGGGTGTGCATCGCGATCCTGCCAATTTGGTGAAGACGATAAAGAAATTGAGAAGGAAAGACGACATTAGCCCAGAAGTGTCAGTCGTGCGAGACATTAGGGAGAAGGAATTGAGGTTGTACACCGATGCTGGACGTGTTTGTCGACCACTCTTCATCGTCGAGAACCAACAGCTGGCACTTCAGAAGAAGCACGTCAAATGGCTCAGTAACGGCCTCAACGATGATGGCGATGAGTACAAATGGGAGCATCTGGTAAAGGGTGGCATCATTGAGTTACTGGATGCTGAGGAAGAGGAAACAGTGATGATATCCATGACCCCTGAAGATCTTGAAAATTCTCGCCTACAGCAAAGTGGTGTTGATCCTCACGCGAACGACGGCGAGTTTGATCCAGCAGCTCGATTGAAGGCTGGCACTCACGCACACACATGGACACATTGCGAAATTCATCCTAGCATGATTCTTGGCATTTGTGCCAGCATTATTCCTTTCCCTGATCATCTGCGCCATTCTCATCATTGCTGGTGGTACTGGTGAGTTCGAGGCTGGCATCTCCAAGGATGGCCAGACCCGCGAGCACGCTCTCCTCGCTTTCACCCTYGGTGTGCGGCAACTCATCGTTGCCGTCAACAAGATGGACACCACTAAGGTAAGAAACGTTTCAC-TTATAGTTAGGCTTCTTGCACATTCTCATCATCTCA-TGTAGTGGAGCGAGGACCGTTTCAACGAAATCATCAAGGAGACGTCAACCTTCATCAAGAAGGTTGGTTACAACCCCAAGGCCGTTGCCTTTGTCCCTATTTCCGGCTGGCACGGTGACAACATGTTGGAGGAGTCCGCTAAGTGAGCATTC--TCGTATCGTCATGACCGCCCCGTCGCTCATGTTGCACTCAAGCATGCCTTGGTACAAGGGCTGGACCAGGGAGACCAAGGCTGGTCCCGTCAAGGGCAAGACCCTCCTCGATGCTATCGATGCCATTGAACCCCCTGTCCGACCTTCCGACAAACCCCTCCGTCTCCCCCTTCAGGATGTCTACAAAATTGGTGGTATCGGAACTGTGCCCGTCGGTCGTGTTGAGACTGGTATCATCAAGTCCGGAATGGTCGTCACTTTCGCCCCCTCCAACGTCACCACTGAAGTTAAGTCCGTCGAAATGCATCACGAACAGCTCGAGCAGGGTAACCCCGGTGACAATGTCGGTTTCAACGTCAAGAACGTGTCAGTGAAGGATATTCGTCGTGGCAACGTGGCCTCCGACTCCAAGAACGATCCCGCCAAGGAAGCGGCCTCTTTCAACGCACAGGTCATCGTCCTCAACCACCCTGGTCAAATTGGTGCTGGTTACGCCCCGGTTCTCGACTGTCACACCGCCCACATCGCCTGCAAGTTCGCCGAGCTCATCGAGAAGATCGATCGTCGTACGGGTAAATCCATCGAAAATTCGCCTAAATTTGTCAAGTCGGGTGACGCCTGCATCGTCAAGCTTGTCCCCAGCAAGCCTATG

>GMM7631France_Laccaria_bicolorproxima_FRANCE_KM067858_KU685869_KU686010_KU686129

CCTGA-TGTGGCTGTTAGCTGGC-TTTTCGAAGCATGTGCTCGT-CCGTCATCTTT-ATCTCT--CCA-CCTGTGCACATTTTGTAGTC-TT-GGATAACTCTCGAGGCAA-CTCGGATT----TTAG-AATCGCCGTGC---TGC---ACAAGTCGGCTTTCCTTTCATTTCC-AAGACTATGTTTT----T--A-TATACA-CCAAAGTATGTTCAAAGAATGTCATC-AATGGGAAC---TTGTTTCCTA--T-AAAATTATACAACTTTCAGCAACGGATCTCTTGGCTCTCGCATCGATGAAGAACGCAGCGAAATGCGATAAGTAATGTGAATTGCAGAATTCAGTGAATCATCGAATCTTTGAACGCACCTTGCGCTCCTTGGTATTCCGAGGAGCATGCCTGTTTGAGTGTCATTAAA-TTCTCAA-CCTTCCAAC--------T-TTTATTAGCTTGG--TTAGGCTTGGA-TGT-GGGGG--TTGCGGGCTTC-AT-----T---AACGAGGTCGGCTCTCCTTAAATGCATTAGCGGAACTTTTGTGGACCGTCTA-TTGGTGTGAT-AATTATCTACGCCGTGGAT-GTGAAGCAG-CTTTAT---GAAGTT-CAGCTTCT-AACCGTCC---ATTTATTTGGACAA----TTTTTGACAA-TTTGAAAGCTCAAATTTAAAATCTGGCAGT-CTTTGGCTGT-CCGAGTTGTAATCTAGAGAAGTATTATCCGCGCTGGACCGTGTACAAGTCTCCTGGAATGGGGCGTCATAGAGGGTGAGAATCCCGTCTTTGACACGGACTGCCAGGGC-TTTGTGATGTGCTCTCAAAGAGTCGAGTTGTTTGGGAATGCAGCTCAAAATGGGTGGTAAATTCCATCTAAAGCTAAATATTGGCGAGAGACCGATAGCGAACAAGTACCGTGAGGGAAAGATGAAAAGAACTTTGGAAAGAGAGTTAAACAGTACGTGAAATTGCTGAAAGGGAAACGCTTGAAGTCAGTCGCGTTGGCCAGGGATCAA-CCTTGC----TTTTTT---GCTTGGCTTACTTTCTGGTCGATGGG-TCAGCATCAATTTTGACTGGTGG-AAAAAGTTCAAGGGAATGTGGCAT-CTTCGGATGTGTTATAGCCTTTGTTCGCATACATCAGTTGGGATTGAGGAACTCAGCACGCCGCAAGGCCGGG---TTTTTAACCACGTACGTGCTTAGGATGCTGGCATAATGGCTTTAATCGACCCGTCTTGAAACACGGACCAAGGAGTCTAACATGCCTGCGAGTGTTTGGGTGAAAAACCCGAGCGCGTAATGAAAGTG-AAAGTTGAGATCCCTGTCGCGGGGAGCATTGACGCCCGGATCTGACCTTTTGTGACGATTCTGCGGTAGAGCATGTTTTCCGCATGCTTTTCCGAAAGTTGACCAAAGACGTTTATCGTTATCTACAAAAGgttgctgatcttcgctcaagtgcc-----atgagatgtgctga-cctctct---ttttcagTGCGTCGAGACCCACAAGGAGTTCAACCTTGCCCTTGCAGTTAAGCATCAAACCATCACAAACGGCCTCAAATACTCTCTGGCGACAGGTAACTGGGGAGATCAAAAAAAGTCAATGTCTTCTAAGGCAGGAGTATCTCAGGTCTTGAACAGATACACATATGCATCCACTCTGTCACATCTTCGTCGGTGTAACACTCCTCTAGGGCGCGAAGGCAAGATCGCCAAACCTCGTCAGTTGCACAACACTCATTGGGGTATGGTGTGTCCTGCGGAAACTCCTGAAGGTCAAGCCTGTGGTCTTGTCAAGAACCTCGCCCTTATGGCGTGCATATCAGTCGGGTCTTACTCCGCGCCCGTTATCGAGTTTTTGGAGGAATGGGGATTGGAGTCGTTGGAAGAGAACGCGCACTCATCAACACCTTGCACAAAAGTTTTCGTGAACGGTGTCTGGATGGGTGTGCATCGCGATCCTGCCAATTTGGTGAAGACGATAAAGAAATTGAGAAGGAAAGATGACATTAGCCCAGAAGTGTCAGTTGTGCGAGACATCAGGGAGAAGGAATTGAGGTTGTACACCGATGCTGGACGTGTTTGTCGACCACTCTTCATCGTCGAGAACCAACAGCTGGCACTTCAGAAGAAGCACGTCAAATGGCTCAGTAACGGCCTCAACGATGATGGTGACGAATACAAATGGGAACATCTGGTAAAGGGTGGCATCATTGAGCTACTGGATGCTGAGGAAGAGGAAACAGTGATGATATCCATGACCCCTGAAGATCTTGAAAATTCTCGCCTACAGCAAAGCGGTGTCGATCCTCACGCGAACGACGGCGAGTTTGATCCAGCAGCTCGATTGAAGGCTGGCACTCACGCACACACCTGGACACATTGCGAAATTCATCCTAGCATGATTCTTGGCATTTGTGCCAGCATTATTCCTTTCCCCGATCATCTGCGCCATTCTCATCATTGCTGGTGGTACTGGTGAGTTCGAGGCTGGTATCTCCAAGGATGGCCAGACCCGCGAGCACGCTCTCCTCGCTTTCACCCTCGGTGTGCGGCAACTCATCGTTGCCGTCAACAAGATGGACACCACCAAGGTAAGAAACCTTTGAC-TTATAGTTAGGCTTCTTGCGCATTCTCATCGTCTCA-TGTAGTGGAGCGAGGACCGTTTCAACGAAATCATCAAGGAGACGTCAACTTTCATCAAGAAGGTTGGTTACAACCCCAAGGCTGTTGCCTTTGTCCCTATTTCCGGCTGGCACGGTGACAACATGTTGGAGGAGTCTCCCAAGTAAGCATTC--TCGTATTGTCATGATCGCCCCGTCGTTCATGTTGCACTC-AGCATGCCTTGGTACAAGGGCTGGACCAAGGAGACCAAGGCTGGTGTCGTCAAGGGCAAGACCCTCCTCGATGCTATTGATGCCATTGAGCCCCCCGTCCGACCTTCCGACAAACCTCTCCGTCTCCCCCTCCAGGATGTCTACAAAATTGGCGGTATCGGAACTGTGCCCGTCGGTCGTGTTGAGACTGGTATCATCAAGGCCGGAATGGTCGTCACCTTTGCTCCCTCCAACGTCACCACTGAAGTCAAGTCCGTGGAAATGCACCACGAGCAGCTCGAGCAGGGTAACCCTGGTGACAATGTCGGTTTCAACGTCAAGAACGTGTCAGTGAAGGATATTCGTCGTGGCAACGTGGCCTCCGACTCCAAGAACGATCCCGCCAAGGAAGCCGCCTCTTTCAACGCACAGGTCATCGTCCTCAACCACCCCGGTCAAATTGGTGCTGGTTACGCCCCGGTTCTCGACTGTCACACCGCTCACATCGCCTGCAAGTTCGCCGAGCTCATTGAGAAGATCGATCGTCGTACGGGTAAATCCATTGAAAACTCGCCTAAATTCGTCAAGTCTGGTGACGCCTGCATCGTCAAGCTCGTTCCCAGCAAGCCTATG

>GMM7733_Laccaria_trichodermophora_USA_TX_KM067870JX504157_JX504230_KU686013_

CCTGA-TGTGACTGTTAGCTGGC-TTTTCGAAGCATGTGCTCGT-CCATCATCTTT-ATCTCT--CCA-CCTGTGCACATTTTGTAGTC-TT-GGATACCTCTCGAGGAAA-CTCGGATT----TTAG-GATCGCCGTGC---TGT---ACAAGTCGGCTTTTCTTTCATTTCC-AAGAYTATGTTTT----T--A-TATACA-CCAAAGTATGTTTATAGAATGTTATC-AATGGGAAC---TTGTTTCCTA--T-AAAATTATACAACTTTCAGCAACGGATCTCTTGGCTCTCGCATCGATGAAGAACGCAGCGAAATGCGATAAGTAATGTGAATTGCAGAATTCAGTGAATCATCGAATCTTTGAACGCACCTTGCGCTCCTTGGTATTCCGAGGAGCATGCCTGTTTGAGTGTCATTAAA-TTCTCAA-CCTTCCAAC--------T-TTTATTAGCTTGG--TTAGGCTTGGA-TGT-GGGGG--TTGCAGGCTTC-AT-----C---ACTGAGGTCGGCTCTCCTTAAATGCATTAGCGGAACTTTTGTGGACCGTCTA-TTGGTGTGAT-AATTATCTACGCCGTGGAT-GTGAAGCAG-CTTTAT---GAAGTT-CTGCTTCT-AACCGTCC---ATTGACTTGGACAA-----TTTTGACAA-TTTGAAAGCTCAAATTTAAAATCTGGCAGT-CTTTGGCTGT-CCGAGTTGTAATCTAGAGAAGTATTATCCGCGCTGGACCGTGTACAAGTCTCCTGGAATGGAGCGTCATAGAGGGTGAGAATCCCGTCTTTGACACGGACTGCCAGGGC-TTTGTGATATGCTCTCAAAGAGTCGAGTTGTTTGGGAATGCAGCTCAAAATGGGTGGTAAATTCCATCTAAAGCTAAATATTGGCGAGAGACCGATAGCGAACAAGTACCGTGAGGGAAAGATGAAAAGAACTTTGGAAAGAGAGTTAAACAGTACGTGAAATTGCTGAAAGGGAAACGCTTGAAGTCAGTCGCGTTGGCCAGGGATCAA-CCTTGC------TTTT---GCTTGGCTTACTTTCTGGTCGATGGG-TCAGCATCAATTTTGACTGGTGG-AAAAAGTTCAAGGGAATGTGGCAT-CTTCGGATGTGTTATAGCCTTTGTTCACATACATTGGTTGGGATTGAGGAACTCAGCACGCCGCAAGGCCGGG---TTTTTAACCACGTACGTGCTTAGGATGCTGGCATAATGGCTTTAATCGACCCGTCTTGAAACACGGACCAAGGAGTCTAACATGCCTGCGAGTATTTGGGTGAAAAACCCGAGTGCGTAATGAAAGTG-AAAGTTGAGATCCCTGTCGTGGGGAGCATTGACGCCCGGATCTGACCTTTTGTGACGATTCTGCGGTAGAGCATGTTTTCCGCATGCTTTTCCGAAAGTTGACCAAGGACGTTTATCGTTATCTACAAAAGgttgctgactttcRctcaagtgcc-----atgagacgtRttga-cctctct---tcttcagTGCGTCGAGACCCACAAGGAGTTCAACCTTGCCCTTGCAGTTAAGCATCAAACCATCACAAACGGCCTCAAATACTCTCTGGCGACAGGTAACTGGGGGGATCAGAAAAAGTCAATGTCTTCCAAGGCAGGAGTATCTCAGGTCTTGAACAGATACACATATGCATCCACTCTGTCACATCTYCGTCGGTGTAACACTCCTCTGGGGCGCGAAGGCAAGATCGCCAAACCTCGTCAGTTGCACAACACCCATTGGGGTATGGTGTGCCCYGCGGAAACTCCTGAAGGTCAAGCCTGTGGTCTTGTCAAGAACCTCGCCCTTATGGCGTGCATATCAGTCGGGTCTTACTCCGCGCCCGTTATCGAGTTTTTGGAGGAATGGGGATTGGARTCATTGGAAGAGAACGCRCACTCATCAACTCCCTGCACMAAAGTTTTCGTGAACGGTGTATGGATGGGTGTGCATCGCGATCCTGCCAACTTGGTGAAGACGATAAAGAAATTGAGAAGGAAAGACGACATTAGCCCWGAAGTGTCAGTTGTGCGAGACATCCGAGAGAAGGARTTGAGGTTGTACACCGATGCTGGACGTGTTTGTCGACCACTTTTCATCGTCGAGAACCAACAGYTGGCACTTCAGAAGAAGCACGTCAAATGGCTCAGTAACGGCCTCAACGATGATGGCGACGARTACAAATGGGAACATTTGGTAAAGGGTGGCATCATYGAGTTACTGGATGCTGAGGAAGAGGAAACGGTGATGATATCCATGACCCCTGAAGATCTTGAAAATTCTCGCCTACAACAAAGTGGCGTTGATCCTCACGCGAACGACGGCGAGTTTGATCCAGCRGCTCGATTAAAGGCTGGCACTCACGCRCACACATGGACACATTGCGAAATTCATCCTAGCATGATTCTTGGCATTTGTGCCAGCATTATTCCTTTCCCCGATCAT------------------------------------------------------------------------------------------------------------------------------------------------------------------------------------------------------------------------------------------------------------------------------------------------------------------------------------------------------------------------------------------------------------------------------------------------------------------------------------------------------------------------------------------------------------------------------------------------------------------------------------------------------------------------------------------------------------------------------------------------------------------------------------------------------------------------------------------------------------------------------------------------------------------------------------------------------------------------------------------------------------------------------------------------

>HKAS41483_Laccaria_angustilamella_TYPE_CHINA__JX504233__

-------------------------------------------------------------------------------------------------------------------------------------------------------------------------------------------------------------------------------------------------------------------------------------------------------------------------------------------------------------------------------------------------------------------------------------------------------------------------------------------------------------------------------------------------------------------------------------------------------------------------------------------------------------------------------------------------------AAGCTCAAATTTAAAATCTGGCAGT-CTTTGGCTGT-CCGAGTTGTAATCTAGAGAAGTATTATCCGCGCTGGACCGTGTACAAGTCTCCTGGAATGGRGCGTCATAGAGGGTGAGAATCCCGTCTTTGACACGGACTGCCAGGGC-TTTGTGATGTGCTCTCAAAGAGTCGAGTTGTTTGGGAATGYAGCTCAAAATGGGTGGTAAATTCCATCTAAAGCTAAATATTGGCGAGAGACCGATAGCGAACAAGTACCGTGAGGGARAGATGAAAAGAACTTTGGAAAGAGAGTTAAACAGTACGTGAAATTGCTGAAAGGGAAACGCTTGAAGTCAGTCGCGTTGGCCAGGGATCAA-CCTTGC----TTTTTT---GCYTGGCTTACTTTCTGGTYRATGGG-TCAGCATCAATTTTGRCYGGTGG-AAAAAGTTCAAGGGAATGTGGCAT-CTTCGGATGTGTTATAGCCTTTGTTCGCATAYAYYRGTTGGGATTGAGGAACTCAGCACGCCGCAAGGCCGGG---TTTTTAACCACGTACGTGCTTAGGATGCTGGCRTAATGGCTTTAATCGACCCGTCTTGAAACACGGACCAAGGAGTCTAACATGCCTGCGAGTGTTTGGGTGAAAAACCCGAGCGCGTAATGAAAGTG-AAAGTTGAGATCCCTGTCGTGGGGAGCATTGACGCCCGGAYCTGACCTTTTGTGACGATTCTGCGGTAGAGCATGT-----------------------------------------------------------------------------------------------------------------------------------------------------------------------------------------------------------------------------------------------------------------------------------------------------------------------------------------------------------------------------------------------------------------------------------------------------------------------------------------------------------------------------------------------------------------------------------------------------------------------------------------------------------------------------------------------------------------------------------------------------------------------------------------------------------------------------------------------------------------------------------------------------------------------------------------------------------------------------------------------------------------------------------------------------------------------------------------------------------------------------------------------------------------------------------------------------------------------------------------------------------------------------------------------------------------------------------------------------------------------------------------------------------------------------------------------------------------------------------------------------------------------------------------------------------------------------------------------------------------------------------------------------------------------------------------------------------------------------------------------------------------------------------------------------------------------------------------------------------------------------------------------------------------------------------------------------------------------------------------------------------------------------------------------------------------------------------------------------------------------------------------------------------------

>HKAS44062_Laccaria_bicolor_CHINA_JX504159_JX504235_KU686068_

-CTGA-TGTGATTGTTAGCTGGC-TTTTCGAAGCATGTGCTCGT-CTGTCATTTTT-ATCTTT--CCA-CCTGTGCACATTTTGTAGTC-TT-GGATACCTCTCGAGGAAA-CTCGGATT----TTAG-GATCGCTGTGC---TGT---ACAAGTCGGCTTTTCTTTCATTTCC-AAGACTATGTTTT----T--A-TATACA-CCAAAGTATGTTTATAGAATGTCATC-AATGGGAAC---TTGTTTCCTA--T-AAAATTATACAACTTTCAGCAACGGATCTCTTGGCTCTCGCATCGATGAAGAACGCAGCGAAATGCGATAAGTAATGTGAATTGCAGAATTCAGTGAATCATCGAATCTTTGAACGCACCTTGCGCTCCTTGGTATTCCGAGGAGCATGCCTGTTTGAGTGTCATTAAA-TTCTCAA-CCTTCCAAC--------T-TTTATTAGCTTGG--TTAGGCTTGGA-TGT-GGGGG--TTGCGGGCTTC-AA-----T---AATGAGGTCGGCTCTCCTTAAATGCATTAGTGGAACTTTTGTGGACCGTCTA-TTGGTGTGAT-AATTATCTACGCCGTGGAT-GTGAAGCAG-CTTTAT---GAAGTT-CTGCTTCT-AACCGTCC---ATTGACTTGGACAA----TTTTTGACAA-TTTGAAAGCTCAAATTTAAAATCTGGCAGT-CTTTGGCTGT-CCGAGTTGTAATCTAGAGAAGTATTATCCGCGCTGGACCGTGTACAAGTCTCCTGGAATGGAGCGTCATAGAGGGTGAGAATCCCGTCTTTGACACGGACTGCCAGGGC-TTTGTGATATGCTCTCAAAGAGTCGAGTTGTTTGGGAATGCAGCTCAAAATGGGTGGTAAATTCCATCTAAAGCTAAATATTGGCGAGAGACCGATAGCGAACAAGTACCGTGAGGGAAAGATGAAAAGAACTTTGGAAAGAGAGTTAAACAGTACGTGAAATTGCTGAAAGGGAAACGCTTGAAGTCAGTCGCGTTGGCCAGGGATCAA-CCTTGC----TTTTTT---GCTTGGCTTACTTTCTGGTCGATGGG-TCAGCATCAATTTTGACCGGTGG-AAAAAGTTCAAAGGAATGTGGCAT-CTTCGGATGTGTTATAGCCTTTGTTCACATACATTGGTTGGGATTGAGGAACTCAGCACGCCGCAAGGCCGGG---TTTTTAACCACGTACGTGCTTAGGATGCTGGCATAATGGCTTTAATCGACCCGTCTTGAAACACGGACCAAGGAGTCTAACATGCCTGCGAGTATTTGGGTGAAAAACCCGAGTGCGTAATGAAAGTG-AAAGTTGAGATCCCTGTCGTGGGGAGCATTGACGCCCGGATCTGACCTTTTGTGACGATTCTGCGGTAGAGCATGTTTTCCGCATGCTTTTTCGAAAGTTGACCAAGGACGTTTATCGTTATCTACAAAAGGTCGCTGACTTTCGCTTAAGTGCC-----ATGAGACGTATTGA-CCTCTCT---TCTTCAGTGCGTCGAGACCCATAAGGAGTTCAACCTTGCCCTTGCAGTTAAGCATCAAACCATCACAAACGGCCTCAAATACTCTCTGGCGACAGGTAACTGGGGGGATCAGAAAAAGTCAATGTCTTCCAAGGCAGGAGTATCTCAGGTCTTGAACAGATACACATATGCATCCACTCTGTCACATCTTCGTCGGTGTAACACTCCTCTAGGGCGCGAAGGCAAGATCGCCAAACCTCGTCAGTTGCACAACACCAATTGGGGAATGGTCTGCCC----------------------------------------------------------------------------------------------------------------------------------------------------------------------------------------------------------------------------------------------------------------------------------------------------------------------------------------------------------------------------------------------------------------------------------------------------------------------------------------------------------------------------------------------------------------------------------------------------------------------------------------------------------------------------------------------------------------------------------------------------------------------------------------------------------------------------------------------------------------------------------------------------------------------------------------------------------------------------------------------------------------------------------------------------------------------------------------------------------------------------------------------------------------------------------------------------------------------------------------------------------------------------------------------------------------------------------------------------------------------------------------------------------------------------------------------------------------------------------------------------------------------------------------------------------------------------------------------------------------------------------------------------------------------------------------------------------------------------------------------------------------

>HKAS45998_Laccaria_acanthospora_CHINA_TIBET_KU685719_KU685870_KU686069_

TCTGA-TGTGGCTGTTAGCTGGC-TTTTCGAAGCATGTGCTCGC-CTGTCATCTTT-ATCTCT--CCA-CCTGTGCACCTTTTGTAGTC-TT-GGATACCTCTCGAGGCAA-CTCGGATT----TTGG-GATCGC------------------GTCAGCTTTCCTTGCATTTCC-AAGACTATGTTTT----T--A-TATACA-CCAAAGAATGTTTAAAGAATGTCATC-AATAGGAAC---TTGTTTCCTA--TAAAAAYTATACAACTTTCAGCAACGGATCTCTTGGCTCTCGCATCGATGAAGAACGCAGCGAAATGCGATAAGTAATGTGAATTGCAGAATTCAGTGAATCATCGAATCTTTGAACGCACCTTGCGCTCCTTGGTATTCCGAGGAGCATGCCTGTTTGAGTGTCATTAAA-TTCTCAA-CCTTCCAGC--------T-TTTATTAGCTTGG--TTAGGCTTGGA-TGT-GGGGG--TTGCTGGCTKC-AC-----T---TATGAGGTCAGCTCTCCTTAAATGCATTAGCGGAACTTTTGTGGACCGTCTA-TTGGTGTGAT-AATTATCTACGCCGTGGAT-GTGAAGCAG-CTTTAT---GAAGTT-CAGCTTCT-AACAGTCC---ATTGACTTGGACAA----TTTTTGACAA-TTTGAAAGCTCAAATTTAAAATCTGGCAGT-CTTTGGCTGT-CCGAGTTGTAATCTAGAGAAGCATTATCCGCGCTGGACCGTGTACAAGTCTCCTGGAATGGAGCGTCATAGAGGGTGAGAATCCCGTCTTTGACACGGACTGCCAGGGC-TTTGTGATATGCTCTCAAAGAGTCGAGTTGTTTGGGAATGCAGCTCAAAATGGGTGGTAAATTCCATCTAAAGCTAAATATTGGCGAGAGACCGATAGCGAACAAGTACCGTGAGGGAAAGATGAAAAGAACTTTGGAAAGAGAGTTAAACAGTACGTGAAATTGCTGAAAGGGAAACGCTTGAAGTCAGTCGCGTTGGCCAGGGATCAA-CCTTGC----ATTTTT---GCTTGGCTTATTTCCTGTTTGACGGG-TCAGCATCAATTTTGACTGGTGG-AAAAAGTTCAAGGGAATGTGGCAT-CTTCGGATGTGTTATAGCCTTTGTTCGCATACATCGGTTGGGATTGAGGAACTCAGCACGCCGAAAGGCCGGG---TTTTTAACCACGTACGTGCTTAGGATGCTGGCATAATGGCTTTAATCGACCCGTCTTGAAACACGGACCAAGGAGTCTAACATGCCTGCGAGTGTTTGGGTGGAAAACTCGAGCGCGTAATGAAAGTG-AAAGTTGAGATCCCTGTCATGGGGAGCATCGACGCCCGGATCTGACCTTTTGTGACGATTCCGCGGTAGAGCATGTTTTTCGCATGCTTTTCCGAAAGTTGACCAAGGACGTTTATCGTTATCTTCAAAAGGTTGCTGAGCTTCGCTCAAGTTCC-----ATGAGATGTGCTGA-CCCCTCT---TCTTCAGTGCGTCGAGACCCACAAGGAGTTCAACCTTGCCCTTGCAGTTAAGCATCAAACCATCACAAACGGCCTCAAATACTCTCTGGCGACAGGTAACTGGGGGGACCAGAAGAAGTCAATGTCTTCCAAGGCGGGAGTATCCCAGGTCTTGAACAGATACACATATGCATCCACTCTGTCACACCTTCGTCGGTGTAACACTCCTCTAGGGCGCGAAGGCAAGATTGCCAAACCTCGTCAGTTACACAACACTAATTGGGGAATGGTCTGCCC----------------------------------------------------------------------------------------------------------------------------------------------------------------------------------------------------------------------------------------------------------------------------------------------------------------------------------------------------------------------------------------------------------------------------------------------------------------------------------------------------------------------------------------------------------------------------------------------------------------------------------------------------------------------------------------------------------------------------------------------------------------------------------------------------------------------------------------------------------------------------------------------------------------------------------------------------------------------------------------------------------------------------------------------------------------------------------------------------------------------------------------------------------------------------------------------------------------------------------------------------------------------------------------------------------------------------------------------------------------------------------------------------------------------------------------------------------------------------------------------------------------------------------------------------------------------------------------------------------------------------------------------------------------------------------------------------------------------------------------------------------------

>HKAS57950_Laccaria_alba_CHINA_TIBET_JX504166_JX504242__

CCTGA-TGTGACTGTTAGCTGGC-TTTTCGAAGCATGTGCTCGT-CCATCATCTTTAATCTCT--CCA-CCTGTGCACATTTTGTAGTC-TT-GGATACCTGTCGAGGCAA-CTCGGATT---TTTAG-GATCGCCGTGC---TGT---ACAAGTCGGCTTTTCTTTCATTTCC-AAGACTATGTTTT----T--A-TATACA-CCAAAGTATGTTTAAAGAATGTCATT-AATAGGAAC---TTGTTTCCTA--T-AAAATTATACAACTTTCAGCAACGGATCTCTTGGCTCTCGCATCGATGAAGAACGCAGCGAAATGCGATAAGTAATGTGAATTGCAGAATTCAGTGAATCATCGAATCTTTGAACGCACCTTGCGCTCCTTGGTATTCCGAGGAGCATGCCTGTTTGAGTGTCATTAAA-TTCTCAA-CCTTCCAAC--------T-TTTATTAGCTTGG--ATAGGCTTGGA-TGT-GGGGG--TTGCAGGCTTC-AT-----C---AATGAGGTCAGCTCTTCTTAAATGCATTAGCGGAACTTTTGTGGACCGTCTA-TTGGTGTGAT-AATTATCTACGCCGTGGAT-GAGAAACAG-CTTTAT---CAAGTT-CAGCTTCT-AACCGTCC---ATTGACTTGGACAA-----TTTTGACAA-TTTGAAAGCTCAAATTTAAAATCTGGCAGT-CTTTGGCTGT-CCGAGTTGTAATCTAGAGAAGTATTATCCGCGCTGGACCGTGTACAAGTCTCCTGGAATGGGGCGTCATAGAGGGTGAGAATCCCGTCTTTGACACGGACTGCCAGGGC-TTTGTGATGTGCTCTCAAAGAGTCGAGTTGTTTGGGAATGCAGCTCAAAATGGGTGGTAAATTCCATCTAAAGCTAAATATTGGCGAGAGACCGATAGCGAACAAGTACCGTGAGGGAAAGATGAAAAGAACTTTGGAAAGAGAGTTAAACAGTACGTGAAATTGCTGAAAGGGAAACGCTTGAAGTCAGTCGCGTTGGCCAGGGATCAA-CCTTGC----TTTTTT---GCTTGGCTTACTTTCTGGTCGATGGG-TCAGCATCAATTTTGACTGGTGG-AAAAAGTTCAAGGGAATGTGGCAT-CTTCGGATGTGTTATAGCCTTTGTTCGCATACATTGGTTGGGATTGAGGAACTCAGCACGCCGCAAGGCCGGG---TTTTTAACCACGTACGTGCTTAGGATGCTGGCATAATGGCTTTAATCGACCCGTCTTGAAACACGGACCAAGGAGTCTAACATGCCTGCGAGTGTTTGGGTGAAAAACCCGAGCGCGTAATGAAAGTG-AAAGTTGAGATCCCTGTCGTGGGGAGCATTGACGCCCGGATCTGACCTTTTGTGACGATTCTGCGGTAGAGCATGT-----------------------------------------------------------------------------------------------------------------------------------------------------------------------------------------------------------------------------------------------------------------------------------------------------------------------------------------------------------------------------------------------------------------------------------------------------------------------------------------------------------------------------------------------------------------------------------------------------------------------------------------------------------------------------------------------------------------------------------------------------------------------------------------------------------------------------------------------------------------------------------------------------------------------------------------------------------------------------------------------------------------------------------------------------------------------------------------------------------------------------------------------------------------------------------------------------------------------------------------------------------------------------------------------------------------------------------------------------------------------------------------------------------------------------------------------------------------------------------------------------------------------------------------------------------------------------------------------------------------------------------------------------------------------------------------------------------------------------------------------------------------------------------------------------------------------------------------------------------------------------------------------------------------------------------------------------------------------------------------------------------------------------------------------------------------------------------------------------------------------------------------------------------------

>HMAS_264310_Laccaria_yunnanensis_CHINA_KX496978___

CCTGA-TGTGGCTGTTAGCTGGC-TTTTCGAAGCATGTGCTCGC-CCATCATCTTT-ATCTCT--CCA-CCTGTGCACCTTTTGTAGTC-TT-GAATACCTCTCGAGGCAA-CTCGGATT----TTAG-GATTGCCGTGC-TTTATACACAAAGTCGGCTTTTCTTTCATTTTC-AAGACTATGTTTT--TCT--A-TACACA-CCAAAGTATGTTTAAAGAATGTCATC-AATAGGAAC---TTGTTTCCTA--T-AAAACTATACAACTTTCAGCAACGGATCTCTTGGCTCTCGCATCGATGAAGAACGCAGCGAAATGCGATAAGTAATGTGAATTGCAGAATTCAGTGAATCATCGAATCTTTGAACGCACCTTGCGCTCCTTGGTATTCCGAGGAGCATGCCTGTTTGAGTGTCATTAAA-TTCTCAACCTTTCCAGC--------T-TTTATTAGTTTGG--TCAGGCTTGGA-TGT-GGGGG--TTGCAGGCTTT------------CATTTACTCTGCTCTCCTTAAATGCATTAGCGGAACTTTTGTGGACCGTCTA-TTGGTGTGAT-AATTATCTACACCGTGGAT-GTAAAGCAGTTTTTAT---AAGGTT-CAGCTTCT-AACTGTCC---ATTGACTTGGACAA-----TTTTGACAA-TTTGA-------------------------------------------------------------------------------------------------------------------------------------------------------------------------------------------------------------------------------------------------------------------------------------------------------------------------------------------------------------------------------------------------------------------------------------------------------------------------------------------------------------------------------------------------------------------------------------------------------------------------------------------------------------------------------------------------------------------------------------------------------------------------------------------------------------------------------------------------------------------------------------------------------------------------------------------------------------------------------------------------------------------------------------------------------------------------------------------------------------------------------------------------------------------------------------------------------------------------------------------------------------------------------------------------------------------------------------------------------------------------------------------------------------------------------------------------------------------------------------------------------------------------------------------------------------------------------------------------------------------------------------------------------------------------------------------------------------------------------------------------------------------------------------------------------------------------------------------------------------------------------------------------------------------------------------------------------------------------------------------------------------------------------------------------------------------------------------------------------------------------------------------------------------------------------------------------------------------------------------------------------------------------------------------------------------------------------------------------------------------------------------------------------------------------------------------------------------------------------------------------------------------------------------------------------------------------------------------------------------------------------------------------------------------------------------------------------------------------------------------------------------------------------------------------------------------------------------------------------------------------------------------------------------------------------

>HMAS_271732_Laccaria_aurantia_CHINA_KX496971___

TCTGA-TGTGACTGTTAGCTGGC-TTTTCGAAGCATGTGCTCGT-CCGTCATCTTT-ATATCT--CCA-CCTGTGCACATTTTGTAGTC-TT-GGATACCTCTCGAGGCAA-CTCGGATT----TAAG-GATCGC------------------GTTGGCTTTCCTTTCATTTCC-AAGACTATGTTTT----T--A-TATACA-CCAAAGTATGTTTAAAGAATGTCATC-AATAGGAAC---TTGTTTCCTA--TAAAAACTATACAACTTTCAGCAACGGATCTCTTGGCTCTCGCATCGATGAAGAACGCAGCGAAATGCGATAAGTAATGTGAATTGCAGAATTCAGTGAATCATCGAATCTTTGAACGCACCTTGCGCTCCTTGGTATTCCGAGGAGCATGCCTGTTTGAGTGTCATTAAA-TTCTCAA-CCTTCCAGC--------T-TTTATTAGCTTGG--TTAGGCTTGGA-TGT-GGGGG--TTGCAGGCTTC-AT-----T---ATTGAGGTCAGCTCTCCTTAAATACATTAGCGGAACTTTTGTGGACCGTCTA-TTGGTGTGAT-AATTATCTACGCCGTGGAT-GTGAAGCAG-CTTTAT---GAAGTT-CAGCTTCT-AATCGTCC---ATTGACTTGGACAA---TTTTATGACAA-TTTGA-------------------------------------------------------------------------------------------------------------------------------------------------------------------------------------------------------------------------------------------------------------------------------------------------------------------------------------------------------------------------------------------------------------------------------------------------------------------------------------------------------------------------------------------------------------------------------------------------------------------------------------------------------------------------------------------------------------------------------------------------------------------------------------------------------------------------------------------------------------------------------------------------------------------------------------------------------------------------------------------------------------------------------------------------------------------------------------------------------------------------------------------------------------------------------------------------------------------------------------------------------------------------------------------------------------------------------------------------------------------------------------------------------------------------------------------------------------------------------------------------------------------------------------------------------------------------------------------------------------------------------------------------------------------------------------------------------------------------------------------------------------------------------------------------------------------------------------------------------------------------------------------------------------------------------------------------------------------------------------------------------------------------------------------------------------------------------------------------------------------------------------------------------------------------------------------------------------------------------------------------------------------------------------------------------------------------------------------------------------------------------------------------------------------------------------------------------------------------------------------------------------------------------------------------------------------------------------------------------------------------------------------------------------------------------------------------------------------------------------------------------------------------------------------------------------------------------------------------------------------------------------------------------------------------------

>KH_07192006_1_Laccaria_JP1_USA_IN_KU685720_KU685871_KU686014_

CCTGA-TGTGACTGTTAGCTGGCTTTCCAAAAGCATGTGCTCGT-CCGTCATCTTTAATCTCT--CCA-CCTGTGCACATTTTGTAGTC-TT-GAATAACTCTCGAGGCAA-CTCGGATT----TTAG-GATCGCC---------------AAGTTGGCTTTCCTTTCATTTCC-AAGACTATGTTTT----T--A-TATACA-CCAAAGTATGTTTAAAGAATGTCATT-AATAGGAAC---TTGTTTCCTA--T-AAAATTATACAACTTTCAGCAACGGATCTCTTGGCTCTCGCATCGATGAAGAACGCAGCGAAATGCGATAAGTAATGTGAATTGCAGAATTCAGTGAATCATCGAATCTTTGAACGCACCTTGCGCTCCTTGGTATTCCGAGGAGCATGCCTGTTTGAGTGTCATTAAA-TTCTCAA-CCTTCCAGC------TTTTTTTATAAGCTTGG--TTAGGCTTGGA-TGT-GGGAG--TTGCGGGCTTC-AT-----CAATAATGAGGTCGGCTTTCCTTAAATGCATTAGCGGAACTTTTGTGGACCATCTA-TTGGTGTGAT-AATTATCTACGCCGTGGAT-GTGAAACAG-CTTTAT---GAAGTT-CAGCTTCA-AACCGTCC---ATTCACTTGGACAA-----TTTTGACAA--TTGAAAGCTCAAATTTAAAATCTGGCAGT-CTTTGGCTGT-CCGAGTTGTAATCTAGAGAAGTATTATCCGCGCTGGACCGTGTACAAGTCTCCTGGAATGGAGCGTCATAGAGGGTGAGAATCCCGTCTTTGACACGGACTGCCAGGGC-TTTGTGATGTGCTCTCAAAGAGTCGAGTTGTTTGGGAATGCAGCTCAAAATGGGTGGTAAATTCCATCTAAAGCTAAATATTGGCGAGAGACCGATAGCGAACAAGTACCGTGAGGGAAAGATGAAAAGAACTTTGGAAAGAGAGTTAAACAGTACGTGAAATTGCTGAAAGGGAAACGCTTGAAGTCAGTCACGTTGGCCAGGGATCAA-CCTTGC----TTTTTT---GCTTGGCTTACTTTCTGGTCGATGGG-TCAGCATCAATTTTGACTGGTGG-AAAAAGTTCAAGGGAATGTGGCAT-CTTCGGATGTGTTATAGCCTTTGTTCACATATATCAGTTGGGATTGAGGAACTCAGCACGCCGCAAGGCCGGG---TTTTTAACCACGTACGTGCTTAGGATGCTGGCATAATGGCTTTAATCGACCCGTCTTGAAACACGGACCAAGGAGTCTAACATGCCTGCGAGTGTTTGGGTGAAAAACCCGAGCGCGTAATGAAAGTG-AAAGTTGAGATCCCTGTCGTGGGGAGCATTGACGCCCGGATCTGACCTTTTGTGACGATTCTGCGGTAGAGCATGTTTTCCGCATGCTTTTCCGAAAGTTGACGAAGGACGTTTATCGTTACCTACAAAAGGTTGCTGACCTTCGCTCAAGTGCT-----ATGAGATGTGCTGA-CCTCTCT---TCTTCAGTGCGTCGAAACCCACAAGGAGTTCAACCTTGCCCTTGCAGTTAAGCACCAAACCATCACAAACGGCCTCAAATACTCTCTGGCGACAGGTAACTGGGGAGATCAGAAAAAATCAATGTCTTCCAAGGCAGGAGTATCTCAGGTCTTGAACAGATACACATATGCATCCACTCTGTCACATCTTCGTCGGTGTAACACCCCCCTAGGGCGCGAAGGCAAGATCGCCAAACCTCGTCAGTTGCACAACACTCATTGGGGTATGGTGTGTCCTGCGGAAACTCCTGAAGGTCAAGCCTGTGGTCTTGTCAAGAAYCTCGCTCTTATGGCGTGCATATCAGTCGGGTCTTACTCCGCGCCCGTTATCGAGTTTTTGGAGGAGTGGGGATTGGAGTCATTGGAAGAGAACGCGCACTCATCAACACCTTGCACAAAAGTTTTCGTGAACGGTGTGTGGATGGGTGTGCATCGCGATCCTGCCAACTTGGTGAAGACGATAAAGAAATTGAGAAGAAAAGACGACATTAGTCCAGAAGTGTCAGTTGTGCGAGACATCAGGGAGAAGGAATTGAGGTTGTACACAGACGCTGGACGTGTTTGTCGACCACTCTTCATMGTCGAGAACCAACAGCTGGCACTTCAGAAGAAGCACGTCAAATGGCTCAGTAACGGCCTCAATGATGATGGCGATGAGTACAAATGGGAACATCTGGTAAAGGGTGGCATCATTGAGTTACTGGATGCTGAGGAAGAGGAAACAGTGATGATATCCATGACCCCTGAAGATCTTGAAAATTCTCGCTTACAACAAAGTGGTGTTGATCCTCACGCGAATGACGGCGAGTTTGATCCAGCAGCTCGTTTGAAGGCTGGCACTCACGCGCACACATGGACACATTGCGAAATTCATCCTAGCATGATTCTTGGCATTTGTGCCAGCATTATTCCTTTCCCTGATCAT------------------------------------------------------------------------------------------------------------------------------------------------------------------------------------------------------------------------------------------------------------------------------------------------------------------------------------------------------------------------------------------------------------------------------------------------------------------------------------------------------------------------------------------------------------------------------------------------------------------------------------------------------------------------------------------------------------------------------------------------------------------------------------------------------------------------------------------------------------------------------------------------------------------------------------------------------------------------------------------------------------------------------------------------

>KH_LA06_002_Laccaria_amethystina_PORTUGAL_KU685759_KU685910_KU686059_KU686162

CCTGA-TGTGGCTGTTAGCTGGC-TTTTCGAAGCATGTGCTCGT-CCGTCATCTTTAATCTCT--CCA-CCTGTGCACATTTTGTAGTCTTT-GGATACSTCTCGAGGCAA-CTCGGATA----TTAG-GATTGCCGTGC---TGT---ACAAGTCGGCTTTTCTTTCATTTCC-AAGACTATGTTTT----T--A-TATACC-CCAAAGTATGTTTAAAGAATGTCATC-AATGGGAAC---TTGTTTCCTA--T-AAAATTATACAACTTTCAGCAACGGATCTCTTGGCTCTCGCATCGATGAAGAACGCAGCGAAATGCGATAAGTAATGTGAATTGCAGAATTCAGTGAATCATCGAATCTTTGAACGCACCTTGCGCTCCTTGGTATTCCGAGGAGCATGCCTGTTTGAGTGTCATTAAA-TTCTCAA-CCTTCCAGC--------T-TTTATTAGCTTGG--TTAGGCTTGGA-TGT-GGGGG--TTGCGGGCTTC-AT-----C---AATGAGGTCGGCTCTCCTTAAATGCATTAGCGGAACTTTTGTGGACYGTCTA-TTGGTGTGAT-AATTATCTACGCCGTGGAT-GTAAAGCAG-CTTTAT---AAAGTT-CAGCTTCT-AACCGTCC--TATTGACTTGGACA---------------------AAGCTCAAATTTAAAATCTGGCAGT-CTTTGGCTGT-CCGAGTTGTAATCTAGAGAAGTATTATCCGCGCTGGACCGTGTACAAGTCTCCTGGAATGGAGCGTCATAGAGGGTGAGAATCCCGTCTTTGACACGGACTGCCAGGGC-TTTGTGATGTGCTCTCGAAGAGTCGAGTTGTTTGGGAATGCAGCTCAAAATGGGTGGTAAATTCCATCTAAAGCTAAATATTGGCGAGAGACCGATAGCGAACAAGTACCGTGAGGGAAAGATGAAAAGAACTTTGGAAAGAGAGTTAAACAGTACGTGAAATTGCTGAAAGGGAAACGCTTGAAGTCAGTCGCGTTGGCCAGGGATCAA-CCTTGC----TTTTTT---GCTTGGCTTACTTTCTGGTCGATGGG-TCAGCATCAATTTTGACTGGTGG-AAAAAGTTCAAGGGAATGTGGCAT-CTTCGGATGTGTTATAGCCTTTGTTCACATATATCAGTTGGGATTGAGGAACTCAGCACGCCGCAAGGCCGGG---TTTTTAACCACGTACGTGCTTAGGATGCTGGCATAATGGCTTTAATCGACCCGTCTTGAAACACGGACCAAGGAGTCTAACATGCCTGCGAGTGTTTGGGTGAAAAACCCGAGCGCGTAATGAAAGTG-AAAGTTGAGATCCCTGTCGTGGGGAGCATTGACGCCCGGATCTGACCTTTTGTGACGATTCTGCGGTAGAGCATGT----------------------------------------------------------------------------------------------------------------------------------CAAGGAGTTCAACCTTGCCcTTGCAGTTAAGCATCAAACCATCACAAACGGCCTCAAATACTCTCTGGCGACAGGTAACTGGGGAGATCAGAAAAAGTCAATGTCTTCCAAGGCAGGAGTATCTCAAGTCTTGAACAGATACACATATGCATCGACTCTGTCACATCTTCGTCGGTGTAACACTCCTCTAGGGCGCGAAGGCAAGATCGCCAAACCTCGTCAGTTGCACAACACTCATTGGGGTATGGTGTGTCCTGCGGAAACTCCTGAAGGTCAAGCTTGTGGTCTTGTCAAGAACCTCGCCCTTATGGCGTGCATATCAGTCGGGTCTTACTCTGCGCCCGTTATCGAGTTTTTGGAGGAGTGGGGATTGGAGTCATTGGAAGAGAACGCGCACTCATCAACACCTTGCACAAAAGTTTTCGTGAACGGTGTCTGGATGGGTGTGCATCGCGATCCTGCCAATTTGGTGAAGACGATAAAGAAATTGAGAAGGAAAGACGAcATTAGCCCAGAAGTGTCAGTTGTGAGAGACATcAGGGAGAAGGAATTGAGGTTGTACACCGATGCTGGACGTGTTTGTCGACCACTCTTCATCGTCGAGAACCAACAGCTGGCACTTCAGAAGAAGCAYGTCAAATGGCTCAGTAAYGGCCTCAACGATGATGGCGACGAGTACAAATGGGAACATCTRGTAAAGGGTGGCATCATTGAGTTACTGGATGCTGAGGAAGAAGAAACAGTGATGATATCCATGACCCCTGAAGATCTTGAAAATTCTCGCCTACAGCAAAGTGGTGTTGATCCTCACGCGAACGACGGCGARTTTGATCCAGCAGCTCGAYTGAAGGCTGGCACTCACGCACACACATGGACACATTGCGAAATTCATCCCAGCATGATTCTTGGCATTTGYGCCAGCATCATTCCTTTCCCTGATCAT----------------------------------------------------------------------CGCGAGCACGCTCTCCTCGCTTTCACCCTCGGTGTGCGGCAACTCATCGTTGCCGTCAACAAGATGGACACCACTA-----------------------------------------------------------AGTGGAGCGAGCACCGTTTCAACGAAATCATCAAGGAGACGTCAACCTTGATCAAGAAAGTTGGTTACAACCCCAAAGCCGTTCCCTTTGTCCCTATTTCCGGCTGGCACGGTGACAACATGTCGGAGGAGTCGTCY------------------------------------------------------AATATGCCTTGGTACAAGGGCTGGACCAAGCAGACCAAGGCTGGTGTCGTCAAGGGCAAGACTCTCCTCGATGCTATTGATGCCATTGAACCCCCCGTCCGACCTTCCGACAAACCTCTCCGTCTTCCCCTCCAGGATGTCTACAAAGTTAGCGGTATTGGAACTGTGCCCGTCGGTCGTGTTCAGACTGGTATCATCAAGGCCGGAATGGTCGTCACTTTCGCTCCTTCCAACGTCACCACTGAAGTCAAGTCCGTCGAAATCAATCACGAACAGCTCGAGCAGGGTAATCCTGGTGACAYTGTCCGTTTCCCCGTCAAGAACGTGTCRGTGAAGGATATTCGTCGTGGCAACGTGGCCTCCGACTCCAAGAACGATCCCGCCAAGGAAGCGGCCTCTTTCAACGCACAGGTCATCGTCATCAACCACCCTGGTAAAATTGGTGCTGGTTACACCCCGGTTCTTGACTGTCACACCGCCCACATCGCCTGCAAGTTTGCCGAGCTCATCGAGAAGATTGATCGTCGTACGGGTAAATCCATTGAAAACTCGCCTAAATTCATAAAGTCGGGTGACGCTTGCATCGTCAAGCTCGTTCCCAGCAAGTCTATG

>KU962988_Laccaria_japonica_TYPE__KU962988___

CCTGA-TGTGGCTGTTAGCTGGC-TTTTCGAAGCATGTGCTCGT-CCGTCATCTTT-ATCTCT--CCA-CCTGTGCACATTTTGTAGTC-TT-GGATACCTCTCGAGGCAA-CTCGGATT----TTAG-GATCGCCG------------------TGGCTTTCCTTTCATTTCC-AAGACTATGTTTT----T--A-TATACA-CCAAAGTATGTTTAAAGAATGTTATC-AATAGGAAC---TTGTTTCCTA--T-AAAATTATACAACTTTCAGCAACGGATCTCTTGGCTCTCGCATCGATGAAGAACGCAGCGAAATGCGATAAGTAATGTGAATTGCAGAATTCAGTGAATCATCGAATCTTTGAACGCACCTTGCGCTCCTTGGTATTCCGAGGAGCATGCCTGTTTGAGTGTCATTAAA-TTCTCAA-CCTTCCAGCTTTTTTTTT-TTTATTAGCTTGG--TTTGGCTTGGA-TGT-GGGTG--TTGCAGGCTTC-AT-----T---AATGAGGTCAGCTCTCCTTAAATGCATTAGCGGAACTTTTGTGGACCGTCTA-TTGGTGTGAT-AATTATCTACGCCGTGGAT-GTGAAGCAG-CTATAT-ATGAAGTT-CAGCTTCT-AACTGTCC---ATTGACTTGGACAA----TTTTTGACAA-TTTGA-------------------------------------------------------------------------------------------------------------------------------------------------------------------------------------------------------------------------------------------------------------------------------------------------------------------------------------------------------------------------------------------------------------------------------------------------------------------------------------------------------------------------------------------------------------------------------------------------------------------------------------------------------------------------------------------------------------------------------------------------------------------------------------------------------------------------------------------------------------------------------------------------------------------------------------------------------------------------------------------------------------------------------------------------------------------------------------------------------------------------------------------------------------------------------------------------------------------------------------------------------------------------------------------------------------------------------------------------------------------------------------------------------------------------------------------------------------------------------------------------------------------------------------------------------------------------------------------------------------------------------------------------------------------------------------------------------------------------------------------------------------------------------------------------------------------------------------------------------------------------------------------------------------------------------------------------------------------------------------------------------------------------------------------------------------------------------------------------------------------------------------------------------------------------------------------------------------------------------------------------------------------------------------------------------------------------------------------------------------------------------------------------------------------------------------------------------------------------------------------------------------------------------------------------------------------------------------------------------------------------------------------------------------------------------------------------------------------------------------------------------------------------------------------------------------------------------------------------------------------------------------------------------------------------------

>KU962989_Laccaria_moshuijun_TYPE__KU962989___

CCTGA-TGTGGCTGTTAGCTGGC-TTTTCGAAGCATGTGCTCGT-CCGTCATCTTT-ATCTCT--CCA-CCTGTGCACATTTTGTAGTC-TT-GGATACCTCTCGAGGCCA-CTCGGATT----TTAG-GATCGCTGTGC---TGT---ACAAGTCGGCTTTCCTTTCATTTCC-AAGACTATGTTTT----T--A-TATACA-CCAAAGTATGTTTAAAGAATGTTATC-AATAGGAAC---TTGTTTCCTA--T-AAAACTATACAACTTTCAGCAACGGATCTCTTGGCTCTCGCATCGATGAAGAACGCAGCGAAATGCGATAAGTAATGTGAATTGCAGAATTCAGTGAATCATCGAATCTTTGAACGCACCTTGCGCTCCTTGGTATTCCGAGGAGCATGCCTGTTTGAGTGTCATTAAA-TTCTCAACCCTTCCAGC---CTTTTT-TTTATTGGCTTGG--TTTGGCTTGGA-TGT-GGGTG--TTGCAGGCTTC-AA-----T---AATGAGGTCAGCTCTCCTTAAATGCATTAGCGGAACTTTTGTGGACCGTCTA-TTGGTGTGAT-AATTATCTACGCCGTGGAT-GTGAAGCAG-CTTTAT-ATGAAGTT-CAGCTTCT-AACCGTCC---ATTGACTTGGACAA----TTTTTGACAA-TTTGA-------------------------------------------------------------------------------------------------------------------------------------------------------------------------------------------------------------------------------------------------------------------------------------------------------------------------------------------------------------------------------------------------------------------------------------------------------------------------------------------------------------------------------------------------------------------------------------------------------------------------------------------------------------------------------------------------------------------------------------------------------------------------------------------------------------------------------------------------------------------------------------------------------------------------------------------------------------------------------------------------------------------------------------------------------------------------------------------------------------------------------------------------------------------------------------------------------------------------------------------------------------------------------------------------------------------------------------------------------------------------------------------------------------------------------------------------------------------------------------------------------------------------------------------------------------------------------------------------------------------------------------------------------------------------------------------------------------------------------------------------------------------------------------------------------------------------------------------------------------------------------------------------------------------------------------------------------------------------------------------------------------------------------------------------------------------------------------------------------------------------------------------------------------------------------------------------------------------------------------------------------------------------------------------------------------------------------------------------------------------------------------------------------------------------------------------------------------------------------------------------------------------------------------------------------------------------------------------------------------------------------------------------------------------------------------------------------------------------------------------------------------------------------------------------------------------------------------------------------------------------------------------------------------------------------

>LaAM/08/1_Laccaria_amethystina_JGI Genome_ITS_28S_RPB2_EF1a

CCTGA-TGTGGCTGTTAGCTGGC-TTTTCGAAGCATGTGCTCGT-CCGTCATCTTT-ATCTCT--CCA-CCTGTGCACATTTTGTAGTC-TT-GGATACCTCTCGAGGCAA-CTCGGATT----TTAG-GATCGCTGTGC---TGT---ACAAGTCGGCTTTCCTTTCATTTCC-AAGACTATGTTTT----T--A-TATACA-CCAAAGTATGTTTAAAGAATGTCATC-AATAGGAAC---TTGTTTCCTA--T-ATAATTATACAACTTTCAGCAACGGATCTCTTGGCTCTCGCATCGATGAAGAACGCAGCGAAATGCGATAAGTAATGTGAATTGCAGAATTCAGTGAATCATCGAATCTTTGAACGCACCTTGCGCTCCTTGGTATTCCGAGGAGCATGCCTGTTTGAGTGTCATTAAA-TTCTCAA-CCTTCCAGC-------TT-TTTATAAGCTTGG--TTAGGCTTGGA-TGT-GGGGG--TTGTGGGCTTC-AT-----T---AATGAGGTCAGCTCTCCTTAAATGCATTAGCGGAACTTTTGTGGACCGTCTA-TTGGTGTGAT-AATTATCTACGCCGTGGAT-GTGAAGCAG-TTTTAT---GAAGTT-CAGCTTCT-AACCGTCC---ATTGACTTGGACAA-----TTTTGACAA-TTTGAAAGCTCAAATTTAAAATCTGGCAGT-CTTTGGCTGT-CCGAGTTGTAATCTAGAGAAGTATTATCCGCGCTGGACCGTGTACAAGTCTCCTGGAATGGGGCGTCATAGAGGGTGAGAATCCCGTCTTTGACACGGACTGCCAGGGC-TTTGTGATGTGCTCTCAAAGAGTCGAGTTGTTTGGGAATGCAGCTCAAAATGGGTGGTAAATTCCATCTAAAGCTAAATATTGGCGAGAGACCGATAGCGAACAAGTACCGTGAGGGAAAGATGAAAAGAACTTTGGAAAGAGAGTTAAACAGTACGTGAAATTGCTGAAAGGGAAACGCTTGAAGTCAGTCGCGTTGGCCAGGGATCAA-CCTTGC----TTTTTT---GCTTGGCTTACTTTCTGGTCGATGGG-TCAGCATCAATTTTGACCGGTGG-AAAAAGTTCAAGGGAATGTGGCAT-CTTCGGATGTGTTATAGCCTTTGTTCGCATACATTGGTTGGGATTGAGGAACTCAGCACGCCGCAAGGCCGGG---TTTTTAACCACGTACGTGCTTAGGATGCTGGCATAATGGCTTTAATCGACCCGTCTTGTAACACGGACCAAGGAGTCTAACATGCCTGCGAGTGTTTGGGTGAAAAACCCGAGCGCGTAATGAAAGTG-AAAGTTGAGATCCCTGTCGTAGGGAGCATTGACGCCCGGATCTGACCTTTTGTGACGATTCTGCGGTAGAGCATGTTTTCCGCATGCTTTTCCGAAAGCTGTCCAAGGACGTTTATCGTTATCTACAAAAGGTTGCTGACCTTCGTTTAAGTGCC-----ATGAGATGTGCTGA-CGCCTAA---TCTTCAGTGTGTCGAGACCCACAAAGAGTTCAACCTTGCCCTTGCAGTTAAGCATCAAACCATCACAAACGGTCTCAAATACTCTCTGGCGACAGGTAACTGGGGAGATCAGAAAAAGTCAATGTCTTCCAAGGCAGGAGTATCTCAGGTCTTGAACAGATACACATATGCGTCCACTCTGTCACATCTTCGTCGGTGTAACACTCCTTTAGGGCGCGAAGGCAAGATCGCCAAACCTCGTCAGCTGCACAACACTCATTGGGGTATGGTGTGTCCTGCGGAAACTCCTGAAGGTCAAGCCTGTGGTCTTGTCAAGAACCTCGCCCTTATGGCGTGCATATCAGTCGGGTCTTACTCTGCGCCTGTTATCGAGTTTTTGGAGGAGTGGGGATTGGAGTCATTGGAAGAGAACGCACACTCATCGACACCTTGCACAAAAGTTTTCGTGAACGGTGTCTGGATGGGTGTGCATCGCGATCCTGCCAATTTGGTCAAGACGATAAAGAAACTGAGAAGGAAAGATGACATCAGCCCAGAAGTGTCAGTTGTGCGAGACATTAGGGAGAAGGAACTCAGGTTGTACACCGATGCTGGACGTGTTTGTCGACCACTCTTCATCGTCGAGAACCAACAGCTGGCACTTCAGAAGAAGCACATCAAATGGCTCAGTAACGGCCTCAACGATGATGGCGACGAGTACAAATGGGAACATCTGGTAAAGGGTGGCATCATTGAGTTACTGGATGCTGAGGAAGAGGAAACGGTGATGATATCCATGACCCCTGAAGATCTTGAAAATTCTCGCCTACAGCAAAGTGGTGTTGACCCCCACGCGAACGACGGCGAGTTTGATCCAGCAGCTCGATTGAAGGCTGGCACTCACGCACACACATGGACTCATTGCGAAATTCATCCTAGCATGATTCTTGGCATTTGTGCCAGCATTATTCCTTTCCCTGATCATCTGCGCCATTCTCATCATTGCTGGTGGTACTGGTGAGTTCGAGGCTGGTATCTCCAAGGATGGCCAGACCCGCGAGCACGCTCTCCTCGCTTTCACCCTCGGTGTGCGGCAACTCATCGTGGCCGTCAACAAGATGGACACCACCAAGGTAAGAAACGTTTGAC-TTATAGTTAGGCTTCTTTCACATTTACATCATCTCAT-GTAGTGGAGCGAGGACCGTTTCAACGAAATCATCAAGGAGACGTCGACCTTCATCAAGAAGGTTGGTTACAACCCCAAGGCCGTTGCCTTTGTCCCTATTTCCGGCTGGCACGGTGACAACATGTTGGAGGAGTCCGCTAAGTGAGCATTC--TCGTATCGTTATGGTCGCCCCGTCGCTCATGTTACATTC-AGCATGCCTTGGTACAAGGGCTGGACCAGGGAGACCAAGGCTGGTCCCGTCAAGGGCAAGACCCTCCTCGATGCTATTGATGCCATTGAGCCCCCCGTCCGACCTTCCGACAAACCCCTCCGTCTCCCCCTCCAGGATGTCTACAAAATTGGCGGTATTGGAACTGTGCCCGTCGGTCGTGTTGAGACTGGTATCATCAAGGCCGGAATGGTCGTCACTTTCGCGCCCTCCAACGTCACCACTGAAGTTAAGTCCGTCGAAATGCATCACGAACAGCTCGAGCAGGGTAACCCTGGTGACAATGTCGGTTTCAACGTCAAGAACGTGTCAGTGAAGGATATTCGTCGTGGCAACGTGGCCTccgactccaagaacgatccggccaaggaagcggcctctttcaacgcacaggtcatcgtcctcaaccaccctggtcaaattggtgctggttacgccccagttctcgactgtcacaccgccCACATCGCCTGCAAGTTCGCCGAGCTTATCGAGAAGATCGATCGTCGTACGGGTAAATCTATTGAAAATTCGCCTAAATTCGTCAAATCGGGTGACGCCTGCATCGTCAAGCTCGTTCCCAGCAAGCCTATG

>MB_FB_001101_Laccaria_fulvogrisea_CHINA_JQ670896___

CCTGA-TGTGGCTGTTAGCTGGC-TTTTCGAAGCATGTGCTCGC-CCATCATCTTT-ATATCT--CCA-CCTGTGCACCTTTTGTAGTC-TT-GGATACCTCTCGAGGCAA-CTCGGATT----TTAG-GATCGCCATCT---------------TGGCTTTCCTTGCATTTCC-AAGACTATGTTTT----T--A-TACACA-CCAAAGTATGTTTAAAGAATGTCATC-AATAGGAAC---TTGTTTCCTA--TAAAAACTATACAACTTTCAGCAACGGATCTCTTGGCTCTCGCATCGATGAAGAACGCAGCGAAATGCGATAAGTAATGTGAATTGCAGAATTCAGTGAATCATCGAATCTTTGAACGCACCTTGCGCTCCTTGGTATTCCGAGGAGCATGCCTGTTTGAGTGTCATTAAA-TTCTCAA-CCTTCCAGC--------T-TTTATTAGCTTGG--TTAGGCTTGGA-TGT-GGGGG--TTGCAGGCTTC-AC-----T---TATGAGGTCAGCTCTCCTTAAATGCATTAGCGGAACTTTTGTGGACCGTCTA-TTGGTGTGAT-AATTATCTACGCCGTGGAT-GTGAAGCAG-CTTTAT---AAAGTT-CAGCTTCT-AACAGTCC---ATTGACTTGGACAA-----TTTTGACAA-TTTGA-------------------------------------------------------------------------------------------------------------------------------------------------------------------------------------------------------------------------------------------------------------------------------------------------------------------------------------------------------------------------------------------------------------------------------------------------------------------------------------------------------------------------------------------------------------------------------------------------------------------------------------------------------------------------------------------------------------------------------------------------------------------------------------------------------------------------------------------------------------------------------------------------------------------------------------------------------------------------------------------------------------------------------------------------------------------------------------------------------------------------------------------------------------------------------------------------------------------------------------------------------------------------------------------------------------------------------------------------------------------------------------------------------------------------------------------------------------------------------------------------------------------------------------------------------------------------------------------------------------------------------------------------------------------------------------------------------------------------------------------------------------------------------------------------------------------------------------------------------------------------------------------------------------------------------------------------------------------------------------------------------------------------------------------------------------------------------------------------------------------------------------------------------------------------------------------------------------------------------------------------------------------------------------------------------------------------------------------------------------------------------------------------------------------------------------------------------------------------------------------------------------------------------------------------------------------------------------------------------------------------------------------------------------------------------------------------------------------------------------------------------------------------------------------------------------------------------------------------------------------------------------------------------------------------------

>MB_FB_001107_Laccaria_yunnanensis_CHINA_JQ670897___

CCTGA-TGTGGCTGTTAGCTGGC-TTTTCGAAGCATGTGCTCGC-CCATCATCTTT-ATCTCT--CCA-CCTGTGCACCTTTTGTAGTC-TT-GAATACCTCTCGAGGCAA-CTCGGATT----TTAG-GATTGCCGTGC-TTTATACACAAAGTCGGCTTTTCTTTCATTTTC-AAGACTATGTTTT--TCT--A-TACACA-CCAAAGTATGTTTAAAGAATGTCATC-AGTAGGAAC---TTGTTTCCTA--T-AAAACTATACAACTTTCAGCAACGGATCTCTTGGCTCTCGCATCGATGAAGAACGCAGCGAAATGCGATAAGTAATGTGAATTGCAGAATTCAGTGAATCATCGAATCTTTGAACGCACCTTGCGCTCCTTGGTATTCCGAGGAGCATGCCTGTTTGAGTGTCATTAAA-TTCTCAACCTTTCCAGC--------T-TTTATTAGTTTGG--TCAGGCTTGGA-TGT-GGGGG--TTGCAGGCTTT------------CATTTACTCTGCTCTCCTTAAATGCATTAGCGGAACTTTTGTGGACCGTCTA-TTGGTGTGAT-AATTATCTACACCGTGGAT-GTAAAGCAGTTTTTAT---AAGGTT-CAGCTTCT-AACTGTCC---ATTGACTTGGACAA-----TTTTGACAA-TTTGA-------------------------------------------------------------------------------------------------------------------------------------------------------------------------------------------------------------------------------------------------------------------------------------------------------------------------------------------------------------------------------------------------------------------------------------------------------------------------------------------------------------------------------------------------------------------------------------------------------------------------------------------------------------------------------------------------------------------------------------------------------------------------------------------------------------------------------------------------------------------------------------------------------------------------------------------------------------------------------------------------------------------------------------------------------------------------------------------------------------------------------------------------------------------------------------------------------------------------------------------------------------------------------------------------------------------------------------------------------------------------------------------------------------------------------------------------------------------------------------------------------------------------------------------------------------------------------------------------------------------------------------------------------------------------------------------------------------------------------------------------------------------------------------------------------------------------------------------------------------------------------------------------------------------------------------------------------------------------------------------------------------------------------------------------------------------------------------------------------------------------------------------------------------------------------------------------------------------------------------------------------------------------------------------------------------------------------------------------------------------------------------------------------------------------------------------------------------------------------------------------------------------------------------------------------------------------------------------------------------------------------------------------------------------------------------------------------------------------------------------------------------------------------------------------------------------------------------------------------------------------------------------------------------------------------

>MB_FB_001109_Laccaria_aurantia_CHINA_JQ681209___

WCTGA-TGTGRCTGTTAGCTGGC-TTTTCRAAGCATGTGCTCGT-CCGTCATCTTT-ATATCT--CCA-CCTGTGCACATTTTGTAGTC-TT-GGATACCTCTCGAGGCAA-CTCGGATT----TAAG-GATCGC------------------GTTGGCTTTCCTTTCATTTCC-AAGACTATGTTTT----T--A-TATACA-CCAAAGTATGTTTAAAGAATGTCATC-AATAGGAAC---TTGTTTCCTA--TAAAAACTATACAACTTTCAGCAACGGATCTCTTGGCTCTCGCATCGATGAAGAACGCAGCGAAATGCGATAAGTAATGTGAATTGCAGAATTCAGTGAATCATWGAATCTTTGAACGCACCTTGCGCTCCTTGGTATTCCGAGGAGCATGCCTGTTTGAGTGTCATTAAA-TTCTCAA-CCTTCCAGC--------T-TTTATTAGCTTGG--TTAGGCTTGGA-TGT-GGGGG--TTGCAGGCTTC-AT-----T---ATTGAGGTCAGCTCTCCTTAAATACATTAGCGGAACTTTTGTGGACCGTCTA-TTGGTGTGAT-AATTATCTACGCCGTGGAT-GTGAAGCAG-CTTTAT---GAAGTT-CAGCTTCT-AATCGTCC---ATTGAWTTGGACAA---TTTTATGACAA-------------------------------------------------------------------------------------------------------------------------------------------------------------------------------------------------------------------------------------------------------------------------------------------------------------------------------------------------------------------------------------------------------------------------------------------------------------------------------------------------------------------------------------------------------------------------------------------------------------------------------------------------------------------------------------------------------------------------------------------------------------------------------------------------------------------------------------------------------------------------------------------------------------------------------------------------------------------------------------------------------------------------------------------------------------------------------------------------------------------------------------------------------------------------------------------------------------------------------------------------------------------------------------------------------------------------------------------------------------------------------------------------------------------------------------------------------------------------------------------------------------------------------------------------------------------------------------------------------------------------------------------------------------------------------------------------------------------------------------------------------------------------------------------------------------------------------------------------------------------------------------------------------------------------------------------------------------------------------------------------------------------------------------------------------------------------------------------------------------------------------------------------------------------------------------------------------------------------------------------------------------------------------------------------------------------------------------------------------------------------------------------------------------------------------------------------------------------------------------------------------------------------------------------------------------------------------------------------------------------------------------------------------------------------------------------------------------------------------------------------------------------------------------------------------------------------------------------------------------------------------------------------------------------------------------

>MB_FB_001110_Laccaria_fulvogrisea_CHINA_JQ681210___

CCTGA-TGTGGCTGTTAGCTGGC-TTTTCGAAGCATGTGCTCGC-CCGTCATCTTT-ATACCT--CCA-CCTGTGCACCTTTTGTAGTC-TT-GGATACCTCTCGAGGCAA-CTCGGATT----TTAG-GATCGCCATGC---------------TGGCTTTCCTTGCATTTCC-AAGACTATGTTTT----T--A-TATACA-CCAAAGTATGTTTAAAGAATGTCATC-AATAGGGAC---TTGTTTCCTA--TAAAAACTATACAACTTTCAGCAACGGATCTCTTGGCTCTCGCATCGATGAAGAACGCAGCGAAATGCGATAAGTAATGTGAATTGCAGAATTCAGTGAATCATCGAATCTTTGAACGCACCTTGCGCTCCTTGGTATTCCGAGGAGCATGCCTGTTTGAGTGTCATTAAA-TTCTCAA-CCTTCCAGC--------T-TTTATTAGCTTGG--TTAGGCTTGGA-TGT-GGGGG--TTGCAGGCTTC-AC-----T---TATGAGGTCAGCTCTCCTTAAATGCATTAGTGGAACTTTTGTGGACCGTCTA-TTGGTGTGAT-AATTATCTACGCCGTGGAT-GTGAAGCAG-CTTTAT---AAAGTT-CAGCTTCT-AACAGTCC---ATTGACTTGGACAA----TTTTTGACAA-TTTGA-------------------------------------------------------------------------------------------------------------------------------------------------------------------------------------------------------------------------------------------------------------------------------------------------------------------------------------------------------------------------------------------------------------------------------------------------------------------------------------------------------------------------------------------------------------------------------------------------------------------------------------------------------------------------------------------------------------------------------------------------------------------------------------------------------------------------------------------------------------------------------------------------------------------------------------------------------------------------------------------------------------------------------------------------------------------------------------------------------------------------------------------------------------------------------------------------------------------------------------------------------------------------------------------------------------------------------------------------------------------------------------------------------------------------------------------------------------------------------------------------------------------------------------------------------------------------------------------------------------------------------------------------------------------------------------------------------------------------------------------------------------------------------------------------------------------------------------------------------------------------------------------------------------------------------------------------------------------------------------------------------------------------------------------------------------------------------------------------------------------------------------------------------------------------------------------------------------------------------------------------------------------------------------------------------------------------------------------------------------------------------------------------------------------------------------------------------------------------------------------------------------------------------------------------------------------------------------------------------------------------------------------------------------------------------------------------------------------------------------------------------------------------------------------------------------------------------------------------------------------------------------------------------------------------------

>PRL3777_Laccaria_ochropurpurea_USA_IL_KU685732_JX504246_KU686024_

CCTGA-TGTGGCTGTTAGCTGGC-TTTTTGAAGCATGTGCTCGT-CCGTCGTCTTT-ATCTCT--CCA-CCTGTGCACATTTTGTAGTC-TT-GGATACCTCTCGAGGCAA-CTCGGATT--TTTAGG-GATCGCCGTGC---TGT---ACAAGTCGGCTTTCTTTTCATTTCC-AAGACTATGTTTT----T--A-TATACA-CCAAAGTATGTTTAAAGAATGTCATA-AAAAGGAAC---TTGATTCCTA--T-AAAATTATACAACTTTCAGCAACGGATCTCTTGGCTCTCGCATCGATGAAGAACGCAGCGAAATGCGATAAGTAATGTGAATTGCAGAATTCAGTGAATCATCGAATCTTTGAACGCACCTTGCGCTCCTTGGTATTCCGAGGAGCATGCCTGTTTGAGTGTCATTAAA-TTCTCAA-CCTTCCAGC--------T-TTTATTAGCTTGGTTTTAGGCTTGGA-TGT-GGGGG--TTGCAGGCTTC-AT-----T---AATGAGGTTAGCTCTCCTTAAATGCATTAGCAGAACTTTTGTGGACTGTCTA-TTGGTGTGAT-AATTATCTACGCCGTGGAT-GTGAAGCAG-ATTTAT---GAAGTT-CAGCTTCT-AACTGTCC---ATTGACTTGGACAA-----TTTTGACAA--TTGAAAGCTCAAATTTAAAATCTGGCAGT-CTTTGGCTGT-CCGAGTTGTAATCTAGAGAAGTATTATCCGCGCTGGACCGTGTACAAGTCTCCTGGAATGGGGCGTCATAGAGGGTGAGAATCCCGTCTTTGACACGGACTGCCAGGGC-TTTGTGATGTGCTCTCAAAGAGTCGAGTTGTTTGGGAATGCAGCTCAAAATGGGTGGTAAATTCCATCTAAAGCTAAATATTGGCGAGAGACCGATAGCGAACAAGTACCGTGAGGGAAAGATGAAAAGAACTTTGGAAAGAGAGTTAAACAGTACGTGAAATTGCTGAAAGGGAAACGCTTGAAGTCAGTCGCGTTGGCCAGGGATCAA-CCTTGC----TTTTTT---GCTTGGCTTACTTTCTGGTCGATGGG-TCAGCATCAATTTTGACTGGTGG-AAAAAGTTCAAGGGAATGTGGCAT-CTTCGGATGTGTTATAGCCTTTGTTCGCATACATTGGTTGGGATTGAGGAACTCAGCACGCCGCAAGGCCGGG---TTTTTAACCACGTACGTGCTTAGGATGCTGGCATAATGGCTTTAATCGACCCGTCTTGAAACACGGACCAAGGAGTCTAACATGCCTGCGAGTGTTTGGGTGAAAAACCCGAGCGCGTAATGAAAGTG-AAAGTTGAGATCCCTGTCGTGGGGAGCATTGACGCCCGGATCTGACCTTTTGTGACGATTCTGCGGTAGAGCATGT------------------------------GGATGTTTATCGTTATCTACAAAAGGTTGCTGACCTTCGCTCAAGTGCC-----ATGAGATGTGCTGA-CCCCTCT---TCTTCAGTGCGTTGAGACCCACAAGGARTTCAACCTTGCCCTTGCAGTTAAGCATCAAACCATCACAAACGGCCTCAAATACTCTCTGGCGACAGGTAACTGGGGAGATCAGAAAAAGTCAATGTCTTCCAAGGCAGGAGTATCTCAGGTCTTGAACAGATACACATATGCATCCACTCTGTCACATCTTCGTCGGTGTAACACTCCTCTAGGGCGCGAAGGCAAGATCGCCAAACCCCGTCAGTTGCACAACACTCATTGGGGTATGGTGTGTCCTGCGGAAACTCCTGAAGGTCAAGCCTGTGGTCTTGTCAAGAACCTCGCCCTTATGGCATGCATATCAGTCGGGTCTTACTCCGCGCCCGTTATCGAGTTTTTGGAGGARTGGGGAYTGGAGTCATTGGAAGAGAACGCACATTCATCGACACCTTGCACAAAAGTTTTCGTGAACGGTGTCTGGATGGGTGTGCATCGCGATCCTGCCAATTTGGTGAAGACGATAAAGAAATTGAGAAGGAAAGACGACATCAGCCCAGAAGTGTCAGTTGTGCGAGACATCAGGGAGAAGGAATTGAGGTTGTACACTGATGCTGGACGTGTTTGTCGACCACTCTTCATTGTCGAGAACCAACAGCTGGCACTTCAGAAGAAGCACGTCAAATGGCTCAGTAACGGCCTCAACGATGATGGCGACGAGTACAAATGGGAACATCTGGTAAAGGGTGGCATCATCGAGTTGCTGGATGCTGAGGAAGAGGAAACGGTGATGATATCCATGACCCCTGAAGATCTTGAAAATTCTCGCCTACAGCAAAGTGGTGTTGATCCTCATGCGAACGACGGCGAGTTTGATCCAGCAGCTCGATTGAAGGCTGGCACTCACGCACACACATGGACACATTGCGAAATTCATCCTAGCATGATTCTTGGCATTTGTG--------------------------------------------------------------------------------------------------------------------------------------------------------------------------------------------------------------------------------------------------------------------------------------------------------------------------------------------------------------------------------------------------------------------------------------------------------------------------------------------------------------------------------------------------------------------------------------------------------------------------------------------------------------------------------------------------------------------------------------------------------------------------------------------------------------------------------------------------------------------------------------------------------------------------------------------------------------------------------------------------------------------------------------------------------------------------

>PRL4777_Laccaria_ochropurpurea_USA_IL_KU685733_KU685883_KU686025_

CCTGA-TGTGGCTGTTAGCTGGC-TTTTTGAAGCATGTGCTCGT-CCGTCGTCTTT-ATCTCT--CCA-CCTGTGCACATTTTGTAGTC-TT-GGATACCTCTCGAGGCAA-CTCGGATT--TTTAGG-GATCGCCGTGC---TGT---ACAAGTCGGCTTTCTTTTCATTTCC-AAGACTATGTTTT----T--A-TATACA-CCAAAGTATGTTTAAAGAATGTCATA-AAAAGGAAC---TTGATTCCTA--T-AAAATTATACAACTTTCAGCAACGGATCTCTTGGCTCTCGCATCGATGAAGAACGCAGCGAAATGCGATAAGTAATGTGAATTGCAGAATTCAGTGAATCATCGAATCTTTGAACGCACCTTGCGCTCCTTGGTATTCCGAGGAGCATGCCTGTTTGAGTGTCATTAAA-TTCTCAA-CCTTCCAGC--------T-TTTATTAGCTTGGTTTTAGGCTTGGA-TGT-GGGGG--TTGCAGGCTTC-AT-----T---AATGAGGTTAGCTCTCCTTAAATGCATTAGCAGAACTTTTGTGGACTGTCTA-TTGGTGTGAT-AATTATCTACGCCGTGGAT-GTGAAGCAG-ATTTAT---GAAGTT-CAGCTTCT-AACTGTCC---ATTGACTTGGACAA-----TTTTGACAA--TTGAAAGCTCAAATTTAAAATCTGGCAGT-CTTTGGCTGT-CCGAGTTGTAATCTAGAGAAGTATTATCCGCGCTGGACCGTGTACAAGTCTCCTGGAATGGGGCGTCATAGAGGGTGAGAATCCCGTCTTTGACACGGACTGCCAGGGC-TTTGTGATGTGCTCTCAAAGAGTCGAGTTGTTTGGGAATGCAGCTCAAAATGGGTGGTAAATTCCATCTAAAGCTAAATATTGGCGAGAGACCGATAGCGAACAAGTACCGTGAGGGAAAGATGAAAAGAACTTTGGAAAGAGAGTTAAACAGTACGTGAAATTGCTGAAAGGGAAACGCTTGAAGTCAGTCGCGTTGGCCAGGGATCAA-CCTTGC----TTTTTT---GCTTGGCTTACTTTCTGGTCGATGGG-TCAGCATCAATTTTGACTGGTGG-AAAAAGTTCAAGGGAATGTGGCAT-CTTCGGATGTGTTATAGCCTTTGTTCGCATACATTGGTTGGGATTGAGGAACTCAGCACGCCGCAAGGCCGGG---TTTTTAACCACGTACGTGCTTAGGATGCTGGCATAATGGCTTTAATCGACCCGTCTTGAAACACGGACCAAGGAGTCTAACATGCCTGCGAGTGTTTGGGTGAAAAACCCGAGCGCGTAATGAAAGTG-AAAGTTGAGATCCCTGTCGTGGGGAGCATTGACGCCCGGATCTGACCTTTTGTGACGATTCTGCGGTAGAGCATGT-------------------------------------------TATCTACAAAAGGTTGCTGACCTTCGCTCAAGTGCC-----aTGGAATGTGCTGA-CCCCTCT---TCTTCAGTGCGTTGAGACCCACAAGGAGTTCAACCTGGCCCTTGCAGTTAAGCATCAAACCATCACAAACGGCCTCAAATACTCTCTGGCGACAGGTAACTGGGGAGATCAGAAAAAGTCAATGTCTTCCAAGGCAGGAGTGTCTCAGGTCTTGAACAGATACACATATGCATCCACTCTGTCACATCTTCGTCGGTGTAACACTCCTCTAGGGCGCGAAGGCAAGATCGCCAAACCCCGTCAGTTGCACAACACTCATTGGGGTATGGTGTGTCCTGCGGAAACTCCTGAAGGTCAAGCCTGTGGTCTTGTCAAGAACCTCGCCCTTATGGCATGCATATCAGTCGGGTCTTACTCCGCGCCTGTTATCGAGTTTTTGGAGGAATGGGGATTGGAGTCATTGGAAGAGAACGCACATTCATCGACACCTTGCACAAAAGTTTTCGTGAACGGTGTCTGGATGGGTGTGCATCGCGATCCTGCCAATTTGGTGAAGACGATAAAGAAATTGAGAAGGAAAGACGACATCAGCCCAGAAGTGTCAGTTGTGCGAGACATCAGGGAGAAGGAATTGAGGTTGTACACTGATGCTGGACGTGTTTGTCGACCACTCTTCATCGTCGAGAACCAACAGCTGGCACTTCAGAAGAAGCACGTCAAATGGCTCAGTAACGGCCTCAACGATGATGGCGACGAGTACAAATGGGAACATCTGGTAAAGGGTGGCATCATYGAGTTRCTGGATGCTGAGGAAGAGGAAACGGTGATGATATCCATGACCCCTGAAGATCTTGAAAATTCTCGCCTACAGCAAAGTGGTGTTGATCCTCATGCGAACGACGGCGAGTTTGATCCAGCAGCTCGATTGAAGGCTGGCACTCACGCACACACATGGACACATTGCGAAATTCATCCTAGCATGATTCTTGGCATTTGT---------------------------------------------------------------------------------------------------------------------------------------------------------------------------------------------------------------------------------------------------------------------------------------------------------------------------------------------------------------------------------------------------------------------------------------------------------------------------------------------------------------------------------------------------------------------------------------------------------------------------------------------------------------------------------------------------------------------------------------------------------------------------------------------------------------------------------------------------------------------------------------------------------------------------------------------------------------------------------------------------------------------------------------------------------------------------

>S238N_Laccaria _bicolor_USA_OR_DQ179123__XM001873347_XM001873347

CCTGA-TGTGATTGTTAGCTGGC-TTTTCGAAGCATGTGCTCAT-CCGTCATCTTT-ATCTCT--CCA-CCTGTGCACATTTTGTAGTC-TT-GGATACCTCTCGAGGAAA-CTCGGATT----TTAG-AATCGCTGTGC---TGT---ACAAGTCGGCTTTTCTTTCATTTCC-AAGACTATGTTTT----T--A-TATACA-CCAAAGTATGTTTATAGAATGTCATC-AATGGGAAC---TTGTTTCCTA--T-AAAATTATACAACCTTCAGCAACGGATCTCTTGGCTCTCGCATCGATGAAGAACGCAGCGAAATGCGATAAGTAATGTGAATTGCAGAATTCAGTGAATCATCGAATCTTTGAACGCACCTTGCGCTCCTTGGTATTCCGAGGAGCATGCCTGTTTGAGTGTCATTAAA-TTCTCAA-CCTTCCAAC--------T-TTTATTAGCTTGG--TTAGGCTTGGA-TGT-GGGGG--TTGCGGGCTTC-AT-----T---AATGAGGTCGGCTCTCCTTAAATGCATTAGCGGAACTTTTGT-GACCGTCTA-TTGGTGTGAT-AATTATCTACGCCGTGGAT-GTGAAGCAG-CTTTAT---GAAGTTCCTGCTTCT-AACCGTCC---ATTGACTTGGACAA-----TTTTGATAA--TTGA--------------------------------------------------------------------------------------------------------------------------------------------------------------------------------------------------------------------------------------------------------------------------------------------------------------------------------------------------------------------------------------------------------------------------------------------------------------------------------------------------------------------------------------------------------------------------------------------------------------------------------------------------------------------------------------------------------------------------------------------------------------TTTCCGCATGCTTTTCCGAAAGTTGACCAAGGACGTTTATCGTTATCTACAAAA-------------------------------------------------------------GTGCGTCGAGACCCATAAGGAGTTCAACCTTGCCCTTGCAGTTAAGCATCAAACCATCACAAACGGCCTCAAATACTCTCTGGCGACAGGTAACTGGGGGGATCAGAAAAAGTCAATGTCTTCCAAGGCAGGAGTATCTCAGGTCTTGAACAGATACACATATGCATCCACTCTGTCACATCTTCGTCGGTGTAACACTCCTCTAGGGCGCGAAGGCAAGATCGCCAAACCTCGTCAGTTGCACAACACCCATTGGGGTATGGTGTGCCCTGCGGAAACCCCTGAAGGTCAAGCCTGTGGTCTTGTCAAGAACCTCGCCCTTATGGCGTGCATATCAGTCGGGTCTTACTCCGCGCCCGTTATCGAGTTTTTGGAGGAATGGGGATTGGAGTCATTGGAAGAGAACGCGCACTCATCAACTCCCTGCACAAAAGTTTTCGTGAACGGTGTATGGATGGGTGTGCATCGCGATCCTGCCAACTTGGTGAAGACGATAAAGAAATTGAGAAGGAAAGACGACATTAGCCCTGAAGTGTCAGTTGTGCGAGACATCCGAGAGAAGGAATTGAGGTTGTACACCGATGCTGGACGTGTTTGTCGACCACTTTTCATCGTCGAGAACCAACAGCTGGCACTTCAGAAGAAGCACGTCAAATGGCTCAGTAACGGCCTCAACGATGATGGCGACGAATACAAATGGGAACATCTGGTAAAGGGTGGCATCATTGAGTTACTGGATGCTGAGGAAGAGGAAACGGTGATGATATCCATGACTCCTGAAGATCTTGAAAATTCTCGCCTACAGCAAAGTGGCGTTGATCTTCACGCGAACGACGGCGAGTTTGATCCAGCAGCCCGATTAAAGGCTGGCACTCACGCACACACATGGACACATTGCGAAATTCATCCTAGCATGATTCTTGGCATTTGTGCCAGCATTATTCCTTTCCCCGATCATCTGCGCCATTCTCATCATTGCTGGTGGTACTGGTGAGTTTGAGGCTGGTATCTCCAAGGATGGCCAGACCCGCGAGCACGCTCTCCTCGCTTTCACCCTCGGTGTGCGGCAACTCATCGTTGCCGTCAACAAGATGGACACCACCA-----------------------------------------------------------AGTGGAGCGAGGACCGTTTCAACGAAATTATCAAGGAGACGTCAAGCTTCATCAAGAAGGTTGGTTACAACCCCAAGGCCGTTGCCTTTGTTCCTATTTCCGGCTGGCACGGTGACAACATGTTGGAGGAGTCCCCT------------------------------------------------------AACATGCCTTGGTTCAAGGGCTGGACCAAGGAGACCAAGGCTGGTGTCGTCAAGGGCAAGACCCTCCTCGATGCTATTGATGCCATTGAGCCCCCCGTCCGACCTTCCGACAAGCCCCTCCGTCTTCCCCTCCAGGACGTCTACAAAATTGGCGGTATCGGAACTGTGCCCGTCGGTCGTGTTGAGACTGGTATCATCAAGGCCGGAATGGTCGTCAATTTCGCTCCCTCCAACGTCACCACTGAAGTCAAGTCCGTCGAAATGCATCACGAACAGCTCGAGCAGGGTAACCCCGGTGACAATGTCGGTTTCAACGTCAAGAACGTGTCAGTGAAGGATATTCGTCGTGGCAACGTGGCCTCCGACTCCAAGAACGATCCCGCCAAGGAAGCGGCCTCTTTCAACGCACAGGTCATCGTCCTCAACCACCCTGGTCAAATTGGTGCTGGTTATGCCCCGGTTCTCGACTGTCACACCGCCCACATTGCCTGCAAGTTCGCCGAGCTCATCGAGAAGATCGATCGTCGTACGGGTAAATCCATTGAAAACTCACCCAAATTCGTCAAGTCGGGTGACGCCTGCATCGTCAAGCTCGTTCCCAGCAAGCCTATG

>MS_13_Laccaria_rubroalba_CHINA_KX449360___

TCTGA-TGTGGCTGTTAGCTGGCTTTTTTGAAGCATGTGCTCGT-CCGTCATCTTT-ATATCT--CCA-CCTGTGCACATTTTGTAGTC-TT-GGATACCTCTCGAGGCAA-CTCGGATT----TAAG-GATC------------------GTGTTGGCTTTCCTTTCACTTCC-AAGACTATGTTTT----T--A-TATACA-CCAAAGTATGTTTAAAGAATGTCATC-AATAGGAAC---TTGTTTCCTA--TAAAAACTATACAACTTTCAGCAACGGATCTCTTGGCTCTCGCATCGATGAAGAACGCAGCGAAATGCGATAAGTAATGTGAATTGCAGAATTCAGTGAATCATCGAATCTTTGAACGCACCTTGCGCTCCTTGGTATTCCGAGGAGCATGCCTGTTTGAGTGTCATTAAA-TTCTCAA-CCTTCCAGC--------T-TTTATTAGCTTGG--TTAGGCTTGGA-TGT-GGGGG--TTGCAGGCTTC-AT-----T---ATTGAGGTCAGCTCTCCTTAAATGCATTAGCGGAACTTTTGTGGACCGTCTA-TTGGTGTGAT-AATTATCTACGCCGTGGAT-GTGAAGCAG-CTTTAT---AAAGTT-CAGCTTCT-AACCGTCC---ATTGACTTGGACAA---TTTTATGACAA-TTTGA-------------------------------------------------------------------------------------------------------------------------------------------------------------------------------------------------------------------------------------------------------------------------------------------------------------------------------------------------------------------------------------------------------------------------------------------------------------------------------------------------------------------------------------------------------------------------------------------------------------------------------------------------------------------------------------------------------------------------------------------------------------------------------------------------------------------------------------------------------------------------------------------------------------------------------------------------------------------------------------------------------------------------------------------------------------------------------------------------------------------------------------------------------------------------------------------------------------------------------------------------------------------------------------------------------------------------------------------------------------------------------------------------------------------------------------------------------------------------------------------------------------------------------------------------------------------------------------------------------------------------------------------------------------------------------------------------------------------------------------------------------------------------------------------------------------------------------------------------------------------------------------------------------------------------------------------------------------------------------------------------------------------------------------------------------------------------------------------------------------------------------------------------------------------------------------------------------------------------------------------------------------------------------------------------------------------------------------------------------------------------------------------------------------------------------------------------------------------------------------------------------------------------------------------------------------------------------------------------------------------------------------------------------------------------------------------------------------------------------------------------------------------------------------------------------------------------------------------------------------------------------------------------------------------------------

>MS_28_Laccaria_rubroalba_CHINA_KX449359___

TCTGA-TGTGGCTGTTAGCTGGCTTTTTTGAAGCATGTGCTCGT-CCGTCATCTTT-ATATCT--CCA-CCTGTGCACATTTTGTAGTC-TT-GGATACCTCTCGAGGCAA-CTCGGATT----TAAG-GATC------------------GTGTTGGCTTTCCTTTCACTTCC-AAGACTATGTTTT----T--A-TATACA-CCAAAGTATGTTTAAAGAATGTCATC-AATAGGAAC---TTGTTTCCTA--TAAAAACTATACAACTTTCAGCAACGGATCTCTTGGCTCTCGCATCGATGAAGAACGCAGCGAAATGCGATAAGTAATGTGAATTGCAGAATTCAGTGAATCATCGAATCTTTGAACGCACCTTGCGCTCCTTGGTATTCCGAGGAGCATGCCTGTTTGAGTGTCATTAAA-TTCTCAA-CCTTCCAGC--------T-TTTATTAGCTTGG--TTAGGCTTGGA-TGT-GGGGG--TTGCAGGCTTC-AT-----T---ATTGAGGTCAGCTCTCCTTAAATGCATTAGCGGAACTTTTGTGGACCGTCTA-TTGGTGTGAT-AATTATCTACGCCGTGGAT-GTGAAGCAG-CTTTAT---AAAGTT-CAGCTTCT-AACCGTCC---ATTGACTTGGACAA---TTTTATGACAA-TTTGA-------------------------------------------------------------------------------------------------------------------------------------------------------------------------------------------------------------------------------------------------------------------------------------------------------------------------------------------------------------------------------------------------------------------------------------------------------------------------------------------------------------------------------------------------------------------------------------------------------------------------------------------------------------------------------------------------------------------------------------------------------------------------------------------------------------------------------------------------------------------------------------------------------------------------------------------------------------------------------------------------------------------------------------------------------------------------------------------------------------------------------------------------------------------------------------------------------------------------------------------------------------------------------------------------------------------------------------------------------------------------------------------------------------------------------------------------------------------------------------------------------------------------------------------------------------------------------------------------------------------------------------------------------------------------------------------------------------------------------------------------------------------------------------------------------------------------------------------------------------------------------------------------------------------------------------------------------------------------------------------------------------------------------------------------------------------------------------------------------------------------------------------------------------------------------------------------------------------------------------------------------------------------------------------------------------------------------------------------------------------------------------------------------------------------------------------------------------------------------------------------------------------------------------------------------------------------------------------------------------------------------------------------------------------------------------------------------------------------------------------------------------------------------------------------------------------------------------------------------------------------------------------------------------------------------

>MS_15_Laccaria_rubroalba_CHINA_KX449358___

TCTGA-TGTGGCTGTTAGCTGGCTTTTTTGAAGCATGTGCTCGT-CCGTCATCTTT-ATATCT--CCA-CCTGTGCACATTTTGTAGTC-TT-GGATACCTCTCGAGGCAA-CTCGGATT----TAAG-GATC------------------GTGTTGGCTTTCCTTTCACTTCC-AAGACTATGTTTT----T--A-TATACA-CCAAAGTATGTTTAAAGAATGTCATC-AATAGGAAC---TTGTTTCCTA--TAAAAACTATACAACTTTCAGCAACGGATCTCTTGGCTCTCGCATCGATGAAGAACGCAGCGAAATGCGATAAGTAATGTGAATTGCAGAATTCAGTGAATCATCGAATCTTTGAACGCACCTTGCGCTCCTTGGTATTCCGAGGAGCATGCCTGTTTGAGTGTCATTAAA-TTCTCAA-CCTTCCAGC--------T-TTTATTAGCTTGG--TTAGGCTTGGA-TGT-GGGGG--TTGCAGGCTTC-AT-----T---ATTGAGGTCAGCTCTCCTTAAATGCATTAGCGGAACTTTTGTGGACCGTCTA-TTGGTGTGAT-AATTATCTACGCCGTGGAT-GTGAAGCAG-CTTTAT---AAAGTT-CAGCTTCT-AACCGTCC---ATTGACTTGGACAA---TTTTATGACAA-TTTGA-------------------------------------------------------------------------------------------------------------------------------------------------------------------------------------------------------------------------------------------------------------------------------------------------------------------------------------------------------------------------------------------------------------------------------------------------------------------------------------------------------------------------------------------------------------------------------------------------------------------------------------------------------------------------------------------------------------------------------------------------------------------------------------------------------------------------------------------------------------------------------------------------------------------------------------------------------------------------------------------------------------------------------------------------------------------------------------------------------------------------------------------------------------------------------------------------------------------------------------------------------------------------------------------------------------------------------------------------------------------------------------------------------------------------------------------------------------------------------------------------------------------------------------------------------------------------------------------------------------------------------------------------------------------------------------------------------------------------------------------------------------------------------------------------------------------------------------------------------------------------------------------------------------------------------------------------------------------------------------------------------------------------------------------------------------------------------------------------------------------------------------------------------------------------------------------------------------------------------------------------------------------------------------------------------------------------------------------------------------------------------------------------------------------------------------------------------------------------------------------------------------------------------------------------------------------------------------------------------------------------------------------------------------------------------------------------------------------------------------------------------------------------------------------------------------------------------------------------------------------------------------------------------------------------------

>MS_20_Laccaria_rubroalba_CHINA_KX449357___

TCTGA-TGTGGCTGTTAGCTGGCTTTTTTGAAGCATGTGCTCGT-CCGTCATCTTT-ATATCT--CCA-CCTGTGCACATTTTGTAGTC-TT-GGATACCTCTCGAGGCAA-CTCGGATT----TAAG-GATC------------------GTGTTGGCTTTCCTTTCACTTCC-AAGACTATGTTTT----T--A-TATACA-CCAAAGTATGTTTAAAGAATGTCATC-AATAGGAAC---TTGTTTCCTA--TAAAAACTATACAACTTTCAGCAACGGATCTCTTGGCTCTCGCATCGATGAAGAACGCAGCGAAATGCGATAAGTAATGTGAATTGCAGAATTCAGTGAATCATCGAATCTTTGAACGCACCTTGCGCTCCTTGGTATTCCGAGGAGCATGCCTGTTTGAGTGTCATTAAA-TTCTCAA-CCTTCCAGC--------T-TTTATTAGCTTGG--TTAGGCTTGGA-TGT-GGGGG--TTGCAGGCTTC-AT-----T---ATTGAGGTCAGCTCTCCTTAAATGCATTAGCGGAACTTTTGTGGACCGTCTA-TTGGTGTGAT-AATTATCTACGCCGTGGAT-GTGAAGCAG-CTTTAT---AAAGTT-CAGCTTCT-AACCGTCC---ATTGACTTGGACAA---TTTTATGACAA-TTTGA-------------------------------------------------------------------------------------------------------------------------------------------------------------------------------------------------------------------------------------------------------------------------------------------------------------------------------------------------------------------------------------------------------------------------------------------------------------------------------------------------------------------------------------------------------------------------------------------------------------------------------------------------------------------------------------------------------------------------------------------------------------------------------------------------------------------------------------------------------------------------------------------------------------------------------------------------------------------------------------------------------------------------------------------------------------------------------------------------------------------------------------------------------------------------------------------------------------------------------------------------------------------------------------------------------------------------------------------------------------------------------------------------------------------------------------------------------------------------------------------------------------------------------------------------------------------------------------------------------------------------------------------------------------------------------------------------------------------------------------------------------------------------------------------------------------------------------------------------------------------------------------------------------------------------------------------------------------------------------------------------------------------------------------------------------------------------------------------------------------------------------------------------------------------------------------------------------------------------------------------------------------------------------------------------------------------------------------------------------------------------------------------------------------------------------------------------------------------------------------------------------------------------------------------------------------------------------------------------------------------------------------------------------------------------------------------------------------------------------------------------------------------------------------------------------------------------------------------------------------------------------------------------------------------------------

>DED7419_Laccaria_THAI2_THAILAND_KX513839KX513840_KU685770_KU685925_KU686075

-CTGA-TGCGGCTGTTAGCTGGC-TTTTCGAAGCATGTGCWCGT-CCATCATCTTT-ATATCT--CCA-CCTGTGCACCTTTTGTAGTC-TT-GAATACCTCTCGAGGCAA-CTCGGATT----TTAG-AATTGCCGTGC---TGT---ACAAGTCGGCTTTTCTATCATTTTC-AAGATTATGTTTT--CTT--A-TACACA-CCAAAGTATGTTTAAAGAATGTCATC-AATAGGAAC---------------T-----------------------------------------------------------------------------------------------------------TTTTTNNNNNNNNNNNNNNNNNNNNNNNNNNNNNNNNNNNNNNNNNNNNNNNNNNNNNNNNNN---------NNNNNNNNN-------------------------NNNNNNNNNNNNNNT--------TTGCAGGCTTC------------ATTGAGATCTGCTCTCCTTAAATGCATTAGTGGAACTTTTGTGGACCGTCTA-TTGGTGTGAT-AATTATCTACACCGTGGAT-GTAAAGCAG-TTTTAT---AAAGTT-CAGCTTCT-AACCGTCC---TTTGACTTGGACAA-----TTTTGACAA--TTGAAAGCTCAAATTTAAAATCTGACAGT-CTTTGGCTGT-CCGAGTTGTAATCTAGAGAAGCATTATCCGCGTTGGACCGTGTACAAGTCTCCTGGAATGGAGCGTCATAGAGGGTGAGAATCCCGTCTTTGACACGGACTACCAGGGCTTTTGTGATGTGCTCTCAAAGAGTCGAGTTGTTTGGGAATGCAGCTCAAAATGGGTGGTAAATTCCATCTAAAGCTAAATATTGGCGAGAGACCGATAGCGAACAAGTACCGTGAGGGAAAGATGAAAAGAACTTTGGAAAGAGAGTTAAACAGTACGTGAAATTGCTGAAAGGGAAACGCTTGAAGTCAGTCGCGTTGGCCAGTGATCAA-CCTTGC----TTTTTT---GCTTGGCTTATTTTCTGGCCAATGGG-TCAGCATCAATTTTGACTGGTGG-AAAAAGTTCAAGGGAATGTGGCAT-CCTTGGATGTGTTATAACCTTTGTTCGCATACATCAGTTGGGATTGAGGAACTCAGCACGCCGAAAGGCCGGG-TTTTTTAAACCACGTACGTGCTTAGGATGCTGGCATAATGGCTTTAATCGACCCGTCTTGAAACACGGACCAAGGAGTCTAACATGCCTGCGAGTGTTTTGGTGGAAAACCAGAGCGCATAATGAAAGTG-AAAGTTGAGATCCCTGTCGTGGGGAGCATCGACGCCCAGATCTGACCTTTTGTGACGATTCTGCGGTAGAGCATGTTTTCcGcATGCTTTTCCGAAAGTTGACCAAGGACGTTTATcGTtATTTACAAAAGGTTGCTGAGCTTCGCTGAAGTGCC-----ATAAGATGTGCTGA-CTTWGCT---TCTTCAGTGCGTCGAGACCCACAAGGAGTTTAATCTTGCTCTTGCAGTGAAGCACCAAACCATCACAAACGGCCTCAAATACTCTCTGGCAACAGGTAACTGGGGAGATCAGAAAAAGTCAATGTCTTCCAAGGCAGGGGTATCTCAGGTCTTGAACAGATACACATATGCATCCACTCTGTCACATCTTCGTCGGTGTAACACTCCTCTAGGACGCGAGGGCAAGATCGCCAAACCTCGTCAGTTGCACAACACTAATTGGGGTATGGTGTGTCCTGCGGAAACGCCTGAAGGTCAAGCCTGTGGTCTTGTCAAGAACCTCGCTCTTATGGCGTGCATATCAGTCGGGTCTTATTCCGCACCCGTTATCGAGTTTTTGGAAGAGTGGGGATTGGAGTCATTAGAAGAAAACGCGCACTCATCGACACCTTGCACGAAAGTTTTTGTGAACGGTGTCTGGATGGGTGTGCATCGCGATCCTGCCAACTTGGTAAAGACGATAAAGAAATTGAGAAGGAAGGACGACATCAGCCCAGAAGTGTCAGTTGTGCGAGACATCAGGGAGAAGGAATTGAGGTTGTACACTGATGCTGGACGTGTTTGTCGGCCACTCTTCATCGTCGAGAATCAACAGCTGGCACTTCAGAAGAAGCACGTCAAGTGGCTCAGCAGCGGCCTCAATGATGATGGCGACGAGTATAAATGGGAACATCTGGTAAAGGGTGGTATCATTGAGTTACTGGATGCTGAGGAAGAGGAAACAGTGATGATATCCATGACCCCTGAAGATCTTGAAAATTCTCGCCTACAGCAAAGTGGCGTTGATCCTCACGCAAACGACGGCGAGTTTGATCCAGCAGCTCGATTGAAGGCTGGCACTCACGCACACACATGGACACATTGCGAGATTCATCCTAGCATGATTCTTGGCATCTGTGCTAGCATGATTCCTTTCCCT----------------------------------------------------------------------------CGCGAGCACGCCCTCCTCGCTTTCACGCTTGGTGTGCGGCAACTCATCGTTGCTGTCAACAAGATGGACACCACTAAGGTAAGAAATGTTTCAC-TTGTAGCYAGCCTTTTTGCACATTTTGATCATCTCA-AATAGTGGAGCGAGGACCGTTTCAACGAAATCATCAAGGAGACGTCAACCTTCATCAAGAAGGTTGGTTACAACCCCAAGGCCGTTGCCTTTGTCCCTATCTCCGGTTGGCACGGTGACAACATGTTGGAGGAATCCACCAAGTAAGCATTT--CCATATCACCATGATCGCCCTGTCGCTCATGTCACACTC-AGCATGCCTTGGTACAAGGGCTGGACCAAGGAGACCAAGGCTGGCGTCGTCAAGGGCAAGACACTYCTCGATGCTATTGATGCCATTGAGCCCCCCGTTCGACCTTCCGACAAGCCCCTCCGCCTTCCCCTCCAGGATGTCTACAAAATTGGCGGTATTGGAACTGTACCTGTCGGTCGTGTTGAGACTGGTATCATCAAAGCGGGAATGGTTGTCACTTTCGCTCCCTCCAACGTCACSACTGAAGTCAAGTCTGTTGAAATGCATCACGAACAACTCGAGCAGGGTAACCCTGGTGACAATGTCGGTTTCAACGTCAAGAACGTGTCAGTGAAGGATATTCGTCGTGGCAACGTGGCCTCCGACTCTAAGAACGATCCCGCCAAGGAAGCGGCCTCTTTCAATGCACAGGTCATCGTCCTCAACCACCCTGGTCAAATTGGTGCTGGTTACGCCCCAGTTCTCGACTGTCACACCGCCCACATCGCCTGCAAGTTCGCTGAGCTCATCGAGAAGATCGATCGTCGTACGGGTAAATCCATTGAAAACAACCCCAAATTCGTCAAGTCGGGTGATGCCTGCATCGTCAAGCTCGTTCCCAGCAAGCCTATG

>DED7426_Laccaria_THAI1_THAILAND_KU685628_KU685771_KU685926_KU686076

CCTGA-TGTGGCTGTTAGCTGGC-TTTTCGAAGCATGTGCTCGC-CCATCATCTTT-ACATTT--CCA-CCTGTGCACCTTTTGTAGTC-TT-GAATACCTCTCGAGGCAA-CTCGGATT----TTAG-GATTGCCGTGC---TGT---ACAAGTCGGCTTTTCTATCATTTTCAAAGACTATGTTTT---CT--A-TACACA-CCAAAGTATGTTTAAAGAATGGCATCAAATAGGAAC---TTGTTTCCTA--T-AAAATTATACAACTTTCAGCAACGGATCTCTTGGCTCTCGCATCGATGAAGAACGCAGCGAAATGCGATAAGTAATGTGAATTGCAGAATTCAGTGAATCATCGAATTTTTGAACGCACCTTGCGCTCCTTGGTATTCCGAGGAGCATGCCTGTTTGAGTGTCATTAAA-TTCTCAACCTTTCCAGC--------T-TTTATTAGTTTGG--TCAGGCTTGGA-TGT-GGGGGTTTTGCAGGCTTT------------TATAAGATCTGCTCTCCTTAAATGCATTAGTGGAACTTTTGTGGACCGTCTA-TTGGTGTGAT-AATTATCTACACCGTGGAT-GTAAAGCAG--------------TT-------------------------------------------------------AAGCTCAAATTTAAAATCTGACAGT-CTTTGGCTGT-CTGAGTTGTAATCTAGAGAAGCATTATCCGCGTTGGACCGTGTACAAGTCTCCTGGAATGGAGCGTCATAGAGGGTGAGAATCCCGTCTTTGACACGGACTGCCAGGGCTTTTGTGATATGCTCTCAAAGAGTCGAGTTGTTTGGGAATGCAGCTCAAAATGGGTGGTAAATTCCATCTAAAGCTAAATATTGGCGAGAGACCGATAGCGAACAAGTACCGTGAGGGAAAGATGAAAAGAACTTTGGAAAGAGAGTTAAACAGTACGTGAAATTGCTGAAAGGGAAACGCTTGAAGTCAGTCACGTTGGCCAGTRATCAA-CCTTGC----TTTTTT---GCTTGGCTTATTTTCTGGTCAATGGG-TCAGCATCAATTTTGACTGGTGG-AAAAAGTTCAAGGGAATGTGGCAT-CCTTGGATGTGTTATAGCCTTTGTTCACATACATCAGTTGGGATTGAGGAACTCAGCACGCCGAAAGGCCGGG---TTTTTAACCACGTACGTGCTTAGGATGCTGGCATAATGGCTTTAATCGACCCGTCTTGAAACACGGACCAAGGAGTCTAACATGCCTGCGAGTGTTTTGGTGAAAAACCAGAGCGCATAATGAAAGTG-AAAGTTGAGATCCCTGTCGTGGGGAGCATTGACGCCCAGATCTGACCTTTTGTGACGATTCTGCGGTAGAGCATGTTTTCCGCATGCTTTTCCGAAAGTTGACCAAGGACGTTTATCGTTATCTACAAAAGGTTGCTGAGCTTTGTCCAACTCCc-----ATGAGATATGCTGA-CCCCTCT---TCTTCAGTGCGTCGAGACCCACAAGGAGTTCAATCTTGCTCTTGCAGTGAAGCACCAAACCATCACAAACGGCCTCAAATACTCTCTGGCGACAGGTAACTGGGGAGATCAGAAAAAGTCAATGTCTTCTAAGGCAGGGGTATCTCAGGTCTTGAACAGATACACATATGCATCCACTCTGTCACACCTTCGTCGGTGTAACACTCCTCTAGGACGTGAAGGCAAGATCGCCAAACCTCGTCAGTTGCACAATACTCATTGGGGTATGGTGTGTCCTGCGGAAACACCTGAAGGTCAAGCTTGCGGTCTTGTCAAGAACCTCGCTCTTATGGCGTGCATATCAGTCGGGTCTTACTCCGCACCCGTTATTGAGTTTTTGGAGGAGTGGGGATTGGAGTCATTGGAAGAGAACGCGCACTCATCGACACCTTGCACAAAAGTTTTCGTGAACGGTGTCTGGATGGGTGTGCACCGCGATCCTGCCAACTTGGTCAAGACGATAAAGAAATTGAGAAGGAAAGATGACATCAGTCCAGAAGTGTCAGTTGTGCGAGATATCAGGGAGAAGGAATTGAGGTTGTACACCGATGCTGGACGTGTTTGTCGACCACTCTTCATCGTCGAGAACCAACAGCTGGCACTTCAGAAGAAGCACGTCAAATGGCTCAGCAACGGCCTCAATGATGATGGCGACGAGTATAAATGGGAACATCTGGTAAAGGGTGGCATCATTGAGTTACTGGATGCGGAGGAAGAGGAAACAGTGATGATATCCATGACCCCTGAAGATCTTGAAAATTCTCGCCTACAGCAAAGTGGTGTTGATCCTCACGCGAACGACGGCGAGTTTGATCCAGCAGCTCGATTGAAGGCTGGCACTCACGCGCACACATGGACACATTGCGAGATTCATCCTAGCATGATTCTTGGCATTTGTGCCAGCATTATTCCTTTCCCTGATCAT----------------------------------------------------------------------CGCGAGCACGCCCTTCTCGCTTTCACGCTCGGTGTGCGGCAACTCATTGTTGCTGTCAACAAGATGGACACCACTAAGGTAAGAAAAGTTTCAG-TTATAGTCAGGCTTTTTGCATATTCTCATCATCTCA-AATAGTGGAGCGAGGACCGTTTCAATGAAATCATCAAGGAGACGTCAACTTTCATCAAGAAGGTCGGTTACAACCCCAAGGCCGTTGCCTTTGTCCCTATTTCCGGTTGGCACGGTGACAACATGTTGGAGGAATCCACCAAGTAAGAATTC--TCATATCGCCATAATCACCCCATCGCTCATATTACACTC-AGCATGCCTTGGTACAAGGGCTGGACAAAGGAGACCAAGGCTGGCGTCGTCAAGGGCAAGACTCTTCTCGATGCTATTGATGCCATTGAGCCCCCCGTTCGACCTTCCGACAAGCCCCTCCGTCTTCCCCTCCAGGATGTCTACAAAATTGGTGGTATTGGAACCGTTCCTGTCGGCCGTGTTGAGACTGGTATCATCAAAGCGGGAATGGTTGTCACTTTCGCTCCCTCCAACGTCACCACTGAAGTCAAGTCTGTTGAAATGCATCACGAACAACTCGAGCAAGGTAACCCTGGTGACAATGTCGGTTTCAACGTCAAGAACGTGTCAGTGAAGGATATTCGTCGTGGTAACGTGGCCTCCGACTCCAAGAACGATCCCGCCAAGGAAGCGGCCTCTTTCAATGCACAGGTCATCGTCCTCAACCACCCTGGTCAAATTGGTGCTGGTTACGCCCCAGTTCTCGACTGTCACACCGCCCACATCGCCTGCAAGTTCGCTGAGCTCATCGAGAAGATCGATCGTCGTACGGGTAAATCCATTGAAAATAACCCCAAATTCGTCAAGTCGGGTGATGCCTGCATCGTCAAGCTCGTTCCCAGCAAGCCTATG

>DED7435_Laccaria_THAI2_THAILAND_KX513841_KU685772_KU686060_ef1a

CCTGCATGCGGCTGTTAGCTGGC-TTTTCGAAGCATGTGCTCGT-CCATCATCTTT-ATATCT--CCA-CCTGTGCACCTTTTGTAGTC-TT-GAATACCTCTCGAGGCAA-CTCGGATT----TTAG-AATTGCCGTGC---TGT---ACAAGTCGGCTTTTCTATCATTTTC-AAGATTATGTTTT--CTT--A-TACACA-CCAAAGTATGTTTAAAGAATGTCATC-AATAGGAAC---------------T----------------------------------------------------------------------------------------------------------------------------------------------------------------------------------------------------T-TTT------------------------TTT----------------TTT--------------------------------------------------------------------------------------------------------------------------------------------------------------------------------AAGCTCAAATTTAAAATCTGACRGT-CTYTGGCTGT-CCGAGTTGTAATCTAGAGAAGCATTATCCGCGTTGGACCGTGTACAAGTCTCCTGGAAYGGAKCGTCATAGAGGGTGAGAATCCCGTCTTTGACACGGACTACCAGGGCTTTTGTGATGTGCTCTCAAAGAGTCGAGTTGTTTGGGAATGCAGCTCAAAATGGGTGGTAAATTCCATCTAAAGCTAAATATTGGCGAGAGACCGATAGCGAACAAGTACCGTGAGGGAAAGATGAAAAGAACTTTGGAAAGAGAGTTAAACAGTACGTGAAATTGCTGAAAGGGAAACGCTTGAAGTCAGTCGCGTTGGCCAGTGATCAA-CCTTGC----TTTTTT---------------------------------------------------------------------------------------------------------------------------------------------------------TTTTTTTAACCACGTACGTGCTTAGGATGCYGGCATAATGGCTTTAATCGACCCGTCTTGAAACACGGACCAAGGAGTCTAACATGCCTGCGAGTGTTTTGGTGGAAAACCAGAGCGCATAATGAAAGTG-AAAGTTGAGATCCCTGTCGTGGGGAGCATCGACGCCCAGATCTGACCTTTTGTGACGATTCTGCGGTAGAGCATGTTTTCCGCATGCTTTTCCGAAAGTTGACCAAGGACGTTTATCGTTATTTACAAAAGGTTGCTGAGCTTCGCTGAAGTGCC-----ATAAGATGTGCTGA-CTTTGCT---TCTTCAGTGCGTCGAGACCCACAAGGAGTTTAATCTTGCTCTTGCAGTGAAGCACCAAACCATCACAAACGGCCTCAAATACTCTCTGGCAACAGGTAACTGGGGAGATCAGAAAAAGTCAATGTCTTCCAAGGCAGGGGTATCTCAGGTCTTGAACAGATACACATATGCATCCACTCTGTCACATCTTCGTCGGTGTAACACTCCTCTAGGACGCGAGGGCAAGATCGCCAAACCTCGTCAGTTGCACAACACTCATTGGGG-------------------------------------------------------------------------------------------------------------------------------------------------------------------------------------------------------------------------------------------------------------------------------------------------------------------------------------------------------------------------------------------------------------------------------------------------------------------------------------------------------------------------------------------------------------------------------------------------------------------------------------------------------------------------------------------------------------------------------------CTCATTATATGCTGGTGGTACTGGTGAATTCGAGGCAGGTATCTCCAAGGATGGCCAGACCCGCGAGCACGCYCTCCTCGCTTTCACGCTTGGTGTGCGGCAACTCATCGTTGCTGTCAACAAGATGGACACCACTAAGGTAAGAAATGTTTCAC-TTRTAGCYAGCCTTTTTGCACATTTTGATCATCTCA-AATAGTGGAGCGAGGACCGTTTCAACGAAATCATCAAGGAGACGTCAACCTTCATCAAGAAGGTTGGTTACAACCCCAAGGCCGTTGCCTTTGTCCCTATCTCCGGTTGGCACGGTGACAACATGTTGGAGGAATCCACCAAGTAAGCATTT--CCATATCACCATGATCGCCCTGTCGCTCATGTTACACTC-AGCATGCCTTGGTACAAGGGCTGGACCAAGGAGACCAAGGCTGGCGTCGTCAAGGGCAAGACACTTCTCGATGCTATTGATGCCATTGAGCCCCCCGTTCGACCTTCCGACAAGCCCCTCCGCCTTCCCCTCCAGGATGTCTACAAAATTGGCGGTATTGGAACTGTACCTGTCGGTCGTGTTGAGACTGGTATCATCAAAGCGGGAATGGTTGTCACGTTCGCTCCCTCCAACGTCACCACTGAAGTCAAGTCTGTTGAAATGCATCACGAACAACTCGAGCAGGGTAACCCTGGTGACAATGTCGGTTTCAACGTCAAGAACGTGTCAGTGAAGGATATTCGTCGTGGCAACGTGGCCTCCGACTCTAAGAACGATCCCGCCAAGGAAGCGGCCTCTTTCAATGCACAGGTCATCGTCCTCAACCACCCTGGTCAAATTGGTGCTGGTTACGCCCCAGTTCTCGACTGTCACACCGCCCACATCGCCTGCAAGTTCGCTGAGCTCATCGAGAAGATCGATCGTCGTACGGGTAAATCCATTGAAAACAACCCCAAATTCGTCAAGTCGGGTGATGCCTGCATCGTCAAGCTCGTTCCCAGCAAGCCTATG

>TWO1166_Laccaria_THAI1_THAILAND_KU685744_KU685895_KU686041_ef1a

CCTGA-TGTGGCTGTTAGCTGGC-TTTTCGAAGCATGTGCTCGC-CCATCATCTTT-ACATTT--CCA-CCTGTGCACCTTTTGTAGTC-TT-GAATACCTCTCGAGGCAA-CTCGGATT----TTAG-GATTGCCGTGC---TGT---ACAAGTCGGCTTTTCTATCATTTTCAAAGACTATGTTTT---CT--A-TACACA-CCAAAGTATGTTTAAAGAATGGCATCAAATAGGAAC---TTGTTTCCTA--T-AAAATTATACAACTTTCAGCAACGGATCTCTTGGCTCTCGCATCGATGAAGAACGCAGCGAAATGCGATAAGTAATGTGAATTGCAGAATTCAGTGAATCATCGAATTTTTGAACGCACCTTGCGCTCCTTGGTATTCCGAGGAGCATGCCTGTTTGAGTGTCATTAAA-TTCTCAACCTTTCCAGC--------T-TTTATTAGTTTGG--TCAGGCTTGGA-TGT-GGGGGTTTTGCAGGCTTT------------TATAAGATCTGCTCTCCTTAAATGCATTAGTGGAACTTTTGTGGACCGTCTA-TTGGTGTGAT-AATTATCTACACCGTGGAT-GTAAAGCAGTTTTTAT---AAAGTT-CAGCTTCT-AACTGTCC---ATTGACTTGGACAC-----TTTTGACAA--TTGAAAGCTCAAATTTAAAATCTGACAGT-CTTTGGCTGT-CTGAGTTGTAATCTAGAGAAGCATTATCCGCGTTGGACCGTGTACAAGTCTCCTGGAATGGAGCGTCATAGAGGGTGAGAATCCCGTCTTTGACACGGACTGCCAGGGCTTTTGTGATATGCTCTCAAAGAGTCGAGTTGTTTGGGAATGCAGCTCAAAATGGGTGGTAAATTCCATCTAAAGCTAAATATTGGCGAGAGACCGATAGCGAACAAGTACCGTGAGGGAAAGATGAAAAGAACTTTGGAAAGAGAGTTAAACAGTACGTGAAATTGCTGAAAGGGAAACGCTTGAAGTCAGTCRCATTGGCCAGTGATCAA-CCTTGC----TTTTTT---GCTTGGCTTATTTTCTGGTCAATGGG-TCAGCATCAATTTTGACTGGTGG-AAAAAGTTCAAGGGAATGTGGCAT-CCTTGGATGTGTTATAGCCTTTGTTCACATACATCAGTTGGGATTGAGGAACTCAGCACGCCGAAAGGCCGGG---TTTTTAACCACGTACGTGCTTAGGATGCTGGCATAATGGCTTTAATCGACCCGTCTTGAAACACGGACCAAGGAGTCTAACATGCCTGCGAGTGTTTTGGTGAAAAACCAGAGCGCATAATGAAAGTG-AAAGTTGAGATCCCTGTCGTGGGGAGCATTGACGCCCAGATCTGACCTTTTGTGACGATTCTGCGGTAGAGCATGTTTTCcGCATGCtTTTCCGAAAGTTGACcAAgGACGTTTATCGTTATCTACAAAAGGTTGCTGAGCtTTGTCCAACT-CC-----ATGAGATATGCTGA-CCCCTCT---TCTTCAGTGCGTCGAGACCCACAAGGAGTTCAATCtTGCTCTTGCAGTGAAGCACCAAACCATCACAAACGGCCTCAAATACTCTCTGGCGACAGGTAACTGGGGAGATCAGAAAAAGTCAATGTCTTCTAAGGCAGGGGTATCTCAGGTCTTGAACAGATACACATATGCATCCACTCTGTCACACCTTCGTCGGTGTAACACTCCTCTAGGACGTGAAGGCAAGATCGCCAAACCTCGTCAGTTGCACAATACTCATTGGGGTATGGTGTGTCCTGCGGAAACACCTGAAGGTCAAGCTTGCGGTCTTGTCAAGAACCTCGCTCTTATGGCGTGCATATCAGTCGGGTCTTACTCCGCACCCGTTATTGAGTTTTTGGAGGAGTGGGGATTGGAGTCATTGGAAGAGAACGCGCACTCATCGACACCTTGCACAAAAGTTTTCGTGAACGGTGTCTGGATGGGTGTGCACCGCGATCCTGCCAACTTGGTCAAGACGATAAAGAAATTGAGAAGGAAAGATGACATCAGTCCAGAAGTGTCAGTTGTGCGAGATATCAGGGAGAAGGAATTGAGGTTGTACACCGATGCTGGACGTGTTTGTCGACCACTCTTCATCGTCGAGAACCAACAGCTGGCACTTCAGAAGAAGCACGTCAAATGGCTCAGCAACGGCCTCAATGATGATGGCGACGAGTATAAATGGGAACATCTGGTAAAGGGTGGCATCATTGAGTTACTGGATGCGGAGGAAGAGGAAACAGTGATGATATCCATGACCCCTGAAGATCTTGAAAATTCTCGCCTACAGCAAAGTGGTGTTGATCCTCACGCGAACGACGGCGAGTTTGATCCAGCAGCTCGATTGAAGGCTGGCACTCACGCGCACACATGGACACATTGCGAGATTCATCCTAGCATGATTCTTGGCATTTGTGCCAGCATTATTCCTTTCCCTGATCAT----------------------------------------------------------------------------------CTTCTCGCTTTCACGCTCGGTGTGCGGCAACTCATCGTCGCTGTCAACAAGATGGACACCACTAAGGTAAGAAACGTTTCAG-TTATAGTCAGGCTTTTTGCATATTCTCATCATCTCA-AATAGTGGAGTGAGGACCGTTTCAATGAAATCATCAAGGAGACGTCAACTTTCATCAAGAAGGTCGGTTACAACCCCAAGGCCGTTGCCTTTGTCCCTATTTCCGGTTGGCACGGTGACAACATGTTGGAGGAATCCACCAAGTAAGCATTC--TCATATCGCCATAATCACCC-ATCGTTCATATTACACTC-AGCATGCCTTGGTACAAGGGCTGGACAAAGGAGACTAAGGCTGGCGTCGTCAAGGGAAAGACCCTCCTCGATGCTATTGATGCCATTGAGCCCCCCGTTCGACCTTCCGACAAGCCCCTCCGTCTTCCCCTCCAGGATGTCTACAAAATTGGTGGTATTGGAACCGTTCCTGTCGGTCGTGTTGAGACTGGTATCATCAAAGCGGGAATGGTTGTCACTTTCGCTCCCTCCAACGTCACCACTGAAGTCAAGTCTGTTGAAATGCATCACGAACAACTCGAGCAAGGTAACCCTGGTGACAATGTCGGTTTCAACGTCAAGAACGTGTCAGTGAAGGATATTCGTCGTGGTAACGTGGCCTCCGACTCCAAGAACGATCCCGCCAAGGAAGCGGCCTCTTTCAATGCACAGGTCATCGTCCTCAACCACCCTGGTCAAATTGGTGCTGGTTACGCCCCAGTTCTCGACTGTCACACYGCCCACATCGCCTGCAAGTTCGCTGAGCTCATCGAGAAGATCGATCGTCGTACGGGTAAATCCATTGAAAATAACCCCAAATTCGTCAAGTCGGGTGATGCCTGCATCGTCAAG---------------------

>TWO1168_Laccaria_THAI3_THAILAND_KU685745_KU685896_KU686042_KU686146

CCTGA-TGTGGCTGTTAGCTGGC-TTTTCGAGGCATGTGCTCGC-CCGTCATCTTT-ATCTCT--CCA-CTTGTGCACCTTTTGTAGTG-TT-GAATACCCCTCGAGGCAA-CTCGGATT----TTAG-GATTGCCGTGC---TGT---ACAAGTCGGCTTTTCTTTCATTTTC-AACGCTATGTTTT---CT--A-TATACA-CCAAAGTATGTTTAAAGAACGTCATC-AATAGGAAC---TTGTTTTCTA--TAAAAACTATACAACTTTCAGCAACGGATCTCTTGGCTCTCGCATCGATGAAGAACGCAGCGAAATGCGATAAGTAATGTGAATTGCAGAATTCAGTGAATCATCGAATCTTTGAACGCACCTTGCGCTCCTTGGTATTCCGAGGAGCATGCCTGTTTGAGTGTCATTAAA-TTCTCAACCTTTCCAGC--------T-TTTATTAGTTTGG--TCAGGCTTGGA-TGT-GGGGG--TTGCAGGCTTT-ATTTACTT---TATGAGGTCTGCTCTTCTGAAATGCATTAGCGGAACTTTTGTGGACCGTCTA-TTGGTATGAT-AATTATCTACACCGTAGAT-GTAAAGCAT-TCTTAT---AGAGTT-CAGCTTCT-AACTGTCC---ATTGACTTGGACA---------------------AAGCTCAAATTTAAAATCTGGCAGT-CTTTGGCTGT-CCGAGTTGTAATCTAGAGAAGCATTATCCGCGCTGGACCGTGTACAAGTCTCCTGGAATGGAGCGTCATAGAGGGTGAGAATCCCGTCTTTGACACGGACTGCCAGGGCTTTTGTGATGTGCTCTCAAAGAGTCGAGTTGTTTGGGAATGCAGCTCAAAATGGGTGGTAAATTCCATCTAAAGCTAAATATTGGCGAGAGACCGATAGTGAACAAGTACCGTGAGGGAAAGATGAAAAGAACTTTGGAAAGAGAGTTAAACAGTACGTGAAATTGCTGAAAGGGAAACGCTTGAAGTCAGTCGCGTTGGCCAGGGATCAA-CCTTGC---TTTTTTT---GCTTGGCTTATTTTCTGGTCAATGGG-TCAGCATCAATTTTGACTGGTGG-AAAAAGTTCAAGGGAATGTGGCATCCTTTGGATGTGTTATAACCTTTGTTCGCATACATTAGTTGGGATTGAGGAACTCAGCACGCCGAAAGGCCGGG---TTATTAACCACGTACGTGCTTAGGATGCTGGCATAATGGCTTTAATCGACCCGTCTTGAAACACGGACCAAGGAGTCTAACATGCGTGCGAGTGTTTTGGTGAAAAACCAGAGCGCATAATGAAAGTG-AAAGTTGAGATCCCTGTCGTGGGGAGCATCGACGCCCAGATCTGACCTTTTGTGACGATTCTGCGGTAGAGCATGTTTTCCGAaTGCTTTTCCGAAAGTGgACCAAGgATGTTtATCGTTATCtACAAAAGgtTGCTGAGCTTTGCCCAAGTGCC-----ATGAGATGTGCTGA-CCCCTCT---TCTTCAGTGCGTCGAGACCCACAAGGAGTTCAATCTTGCTCTTGCAGTGAAGCACCAAACCATCACAAACGGCCTCAAATACTCTCTGGCGACAGGTAACTGGGGAGATCAGAAGAAGTCAATGTCTTCCAAGGCAGGGGTATCTCAGGTCTTGAACAGATATACATATGCATCCACTCTGTCACATCTTCGTCGATGTAACACTCCTCTAGGACGGGAAGGCAAGATCGCCAAACCTCGTCAGTTGCACAATACTCATTGGGGTATGGTGTGTCCTGCGGAAACTCCTGAAGGTCAAGCCTGTGGTCTTGTCAAGAACCTCGCTCTTATGGCGTGCATATCAGTYGGGTCTTACTCCGCACCCGTCATTGARTTTTTGGAGGAGTGGGGATTGGAGTCATTGGAAGAGAACGCGCACTCATCRACACCTTGCACAAAAGTTTTCGTGAACGGTGTTTGGATGGGTGTGCATCGCGATCCTGCCAACTTGGTRAAGACGATAAAAAAATTGAGAAGGAAAGATGACATCAGCCCAGAAGTGTCAGTTGTGCGAGACATCAGGGAGAAGGAATTGAGGTTGTACACCGATGCTGGACGTGTTTGTCGACCACTCTTCATCGTCGAGAACCAACAGCTGGCACTTCAGAAGAAGCATGTCAAATGGCTCAGCAACGGCCTCAATGATGAYGGGGACGAGTATAAATGGGAACATTTGGTAAAGGGTGGCATCATTGAGTTACTGGATGCGGARGAAGAGGAAACAGTGATGATATCTATGACTCCTGAAGATCTTGAAAATTCTCGCGTACAGCAAAGTGGTGTTGATCCTCACGCGAACGACGGCGAGTTTGATCCAGCAGCTCGATTGAAGGCTGGCACTCACGCACACACATGGACGCATTGCGAAATTCATCCTAACGTGATTCTTGGCATTTGTGCCAGCATTATTCCTTTCCCTG---------------------------------------------------------------------------CGCGAGCACGCTCTCCTCGCTTTCACGCTCGGTGTGCGGCAACTCATCGTTGCTGTCAACAAGATGGACACCACTAAGGTAAGAAATGCTTCAC-ATATAGTCTGGCTTTTTGCACATTCTCATCATTTCA-AATAGTGGAGTGAGGACCGTTTCAACGAAATTATTAAGGAGACATCAACCTTTATCAAGAAGGTTGGTTACAACCCCAAGGCCGTTGCCTTTGTCCCTATTTCTGGTTGGCACGGTGACAACATGTTGGAGGAATCCACCAAGTAAGCATTC--TCGTATCGTTATGATCGCCCTGTTGCTCATATTTCACTC-AGCATGCCTTGGTAYAAGGGATGGACCAAGGAGACCAAGGCTGGTGTCGTCAAGGGCAAGACCCTCCTCGATGCTATTGATGCCATTGAGCCCCCCGTTCGACCTTCCGACAAACCCCTCCGTCTTCCCCTCCAGGATGTCTACAAAATTGGCGGTATTGGAACCGTGCCTGTCGGTCGTGTTGAGACTGGTATCATCAAAGCGGGAATGGTCGTCACTTTCGCTCCCTCCAACGTCACCACTGAAGTCAAGTCTGTTGAAATGCACCACGAACAGCTCGAGCAGGGTAACCCTGGTGACAATGTCGGTTTCAACGTCAAGAACGTGTCAGTGAAGGATATTCGACGTGGCAACGTGGCCTCCGACTCCAAGAACGATCCCGCCAAGGAAGCGGCCTCTTTCAATGCACAGGTCATCGTCCTCAACCACCCTGGTCAAATCGGTGCTGGCTACGCYCCGGTTCTCGACTGTCACACCGCCCACATCGCCTGCAAGTTCGCTGAGCTCATCGAGAAGATTGATCGTCGTACGGGTAAATCCATTGAAAACAACCCCAAATTCGTCAAGTCGGGTGATGCATGCATCGTCAAACTCGTTCCCAGCAAGCCTATG

>TWO1178_Laccaria_THAI4_THAILAND_KU685746_KU685897_KU686043_KU686147

CCTGA-TGTGGCTGTTAGCTGGC-TTTTCGAAGCATGTGCTCGC-CCATCATCTTT-ATCTCT--CCA-CCTGTGCACCTTTTGTAGTC-TT-GAATACCTCTCGAGATGA-CTCGGATT----TTAG-AATTGCCGTGC---TGT---ACAAGTCGGCTTTTCTTTCATTTTC-AAGACTATGTTTT--TGT--A-TACACA-CCAAAGTATGTTTAAAGAATGTCATC-AATAGGAAC---TTGTTTCCTG--T-AAAACTATACAACTTTCAGCAACGGATCTCTTGGCTCTCGCATCGATGAAGAACGCAGCGAAATGCGATAAGTAATGTGAATTGCAGAATTCAGTGAATCATCGAATCTTTGAACGCACCTTGCGCTCCTTGGTATTCCGAGGAGCATGCCTGTTTGAGTGTCATTAAA-TTCTCAA-CCTTCCAGC--------T-TTTATTAGTTTGG--TTAGGCTTGGA-TGTGGGGGG--TTGCAGGCTTT-ATTCACTT---TATGAAGTCTGCTCTCCTTAAATGCATTAGTGGAACTTTTGTGAACCATCTTATTGGTGTGAT-AATTATCTATGCCATGGAT-GTGAAGCAG--TTTTT---AAAGTT-CAGCTTCT-AACTGTCC---ATTGACTTGGACAA-----TATTGACAA-TTTGAAAGCTCAAATTTAAAATCTGGCAGT-CTTTGGCTGT-CCGAGTTGTAATCTAGAGAAGCATTATCCGCGTTGGACCGTGTACAAGTCTCCTGGAATGGAGCGTCATAGAGGGTGAGAATCCCGTCTTTGACACGGACTGCCAGGGC-TTTGTGATGTGCTCTCAAAGAGTCGAGTTGTTTGGGAATGCAGCTCAAAATGGGTGGTAAATTCCATCTAAAGCTAAATATTGGCGAGAGACCGATAGCGAACAAGTACCGTGAGGGAAAGATGAAAAGAACTTTGGAAAGAGAGTTAAACAGTACGTGAAATTGCTGAAAGGGAAACGCTTGAAGTCAGTCGCGTTGGCCAGGGATCAA-CCTTGC---TTTCTTT---GCTTGGCTAATTTTCTGGTCAATGGG-TCAGCATCAATTTTGACTGGTGG-AAAAAGTTCAAGGGAATGTGGCAT-CTTCGGATGTGTTATAACCTTTGTTCACATACATCAGTTGGGATTGAGGAACTCAGCACGCCGAAAGGCCGGG---TTTTTAACCACGTACGTGCTTAGGATGCTGGCATAATGGCTTTAATCGACCCGTCTTGAAACACGGACCAAGGAGTCTAACATGCCTGCGAGTGTTTTGGTGAAAAACCAGAGCGCGTAATGAAAGTG-AAAGTTGAGATCCCTGTCGTGGGGAGCATTGACGCCCAGATCTGACCTTTTGTGACGATTCTGCGGTAGAGCATGTGTTCCGCATGCTTTtCCGAAAGTtGACcAaGgACGTTtATCGtTATCTACAAAAGgTtGCTGAGCTtCACTCAAGTGcC-----ATGAGATGTGCTGA-CcTCT-----tCtTCAGTGCGTCGAGACCCACAAGGAGTTCAATCTCGCTCtTGCTGTGAAGCACCAAACTATCACAAACGGCCTCAAATACTCTCTGGCCACAGGCAACTGGGGAGATCAGAAAAAGTCGATGTCTTCCAAGGCAGGGGTATCTCAGGTTTTGAACAGATACACATATGCATCCACTCTGTCTCATCTTCGTCGGTGTAACACTCCTCTAGGACGCGAAGGCAAGATCGCCAAGCCTCGTCAGTTGCACAACACTCACTGGGGTATGGTGTGTCCTGCGGAAACTCCTGAAGGTCAAGCCTGTGGTCTTGTCAAGAACCTTGCTCTTATGGCGTGCATATCAGTGGGGTCTTATTCCGCACCCGTTATCGAGTTTTTGGAGGAGTGGGGATTGGAGTCATTGGAAGAGAACGCGCACTCATCGACACCTTGCACAAAAGTTTTCGTGAACGGTGTCTGGATGGGTGTGCATCGTGATCCTGCCAACTTGGTGAAGACGATAAAGAAATTGAGAAGGAAGGATGACATCAGCCCAGAAGTGTCAGTTGTGCGAGACATCAGGGAGAAGGAATTGAGGTTGTACACTGATGCTGGACGTGTTTGTCGACCACTCTTCATCGTCGAGAACCAACAGCTGGCACTACAGAAGAAGCAYGTCAAATGGCTCAGCAACGGTCTCAATGATGATGGAGACGAGTATAAATGGGAGCATTTAGTAAAGGGTGGCATCATTGAGTTGCTAGATGCGGAGGAAGAGGAAACAGTGATGATATCCATGACCCCTGAAGATCTTGAAAATTCTCGCCTACAGCAAAGTGGCCTTGATCCTCACGCTAACGACGGCGAGTTTGACCCAGCAGCTCGATTGAAGGCTGGCACCCACGCACACACATGGACACATTGCGAGATTCATCCTAGCATGATTCTTGGCATTTGTGCTAGCATTATTCCTTTCCcTGATCAT----------------------------------------------------------------------CGCGAGCACGCTCTTCTCGCTTtCACCCTCGGTGtGCGGCAACTTATCGTTGCCGTCAaCAAGATGgACACCACCAAGGTATGAAATGTTTCA--TTATAGTCAGGC--TTTGCACA-TCTCATCATCTCA-AATAGTGGAGCGAGGACCGTTTCAACGAAATCATCAAGGAAACGTCAACCTTCATCAAGAAGGTTGGTTACAACCCAAAGGCCGTTGCCTTTGTTCCTATTTCCGGTTGGCACGGTGACAACATGTTGGAGGAGTCCACTAAGTAAACATTC--TCGTATCTTCATGATCGCTCTGTCTCTCATATTACACTC-AGCATGCCTTGGTACAAGGGCTGGACCAAGGAGACCAAGGCTGGTGTCGTGAAGGGCAAGACCCTTCTCGATGCTATTGATGCCATTGAGCCTCCCGTTCGACCTTCTGACAAACCCCTCCGCCTCCCCCTCCAGGATGTCTACAAAATTGGCGGTATCGGAACCGTACCCGTCGGTCGTGTTGAGACCGGTATCATCAAGGCCGGAATGGTCGTCACTTTCGCTCCCTCCAACGTTACCACTGAAGTCAAGTCTGTTGAAATGCATCACGAACAACTCGAGCAGGGTAACCCCGGTGACAATGTCGGTTTCAATGTCAAGAACGTGTCAGTGAAGGATATTCGTCGTGGCAACGTGGCCTCCGACTCCAAGAACGATCCCGCCAAGGAAGCGGCCTCTTTCAATGCACAGGTCATCGTCCTCAACCACCCTGGTCAAATTGGTGCTGGTTACGCCCCAGTTCTCGATTGTCACACCGCCCACATTGCCTGCAAATTCGCTGAGCTCATCGAGAAGATCGACCGTCGTACGGGTAAATCCATTGAAAACAACCCCAAATTCGTCAAGTCGGGTGATGCCTGCATCGTCAAGCTTGTACCCAGCAAGCCTATG

>TWO1184_Laccaria_THAI6_THAILAND_KU685747_KU685898__KU686148

CCTGA-TGTGGCTGTTAGCTGGC-TTTTTGAAGCATGTGCTCGC-TCATCATCTTT-ATCTCT--CCA-CCTGTGCACCTTTTGTAGTCTTT-GAATACCTCTCGAGGCAA-CTCGGATT----TTAG-GATCGCTGTAC---TGTA--AAAAGTCGACTTTCCTTTCATTTTC-AAGACTATGTTTA----A--AATACACA-CCAAAGAAAGTTTAAAGAATGTCATT-AATAGGAAC---TTGTTTCCTA--T-AAAACTATACAACTTTCAGCAACGGATCTCTTGGCTCTCGCATCGATGAAGAACGCAGCGAAATGCGATAAGTAATGTGAATTGCAGAATTCAGTGAATCATCGAATCTTTGAACGCACCTTGCGCTCCTTGGTATTCCGAGGAGCATGCCTGTTTGAGTGTCATTTAA-TTCTCAA-CCTTCTAGC--------TTTTTATTAGCTTGG--ATAGGCTTGGA-TGT-GGGAG--TTGCAGGCTTC-AC-----T---CATGAGGTTGGCTCTCCTTAAATGCATTAGCAGAACTTTTGTGGACCATCTA-TTGGTGTGAT-AATTATCTACGCCGTGGAT-GTGAAGCAG-CTTTAT---GAAGTT-CAGCTTCT-AGTCGTCC---ATTGACTTGGACAA-----TTTTGACAA--TTGAAAGCTCAAATTTAAAATCTGGCAGT-CTTTGGCTGT-CCGAGTTGTAATCTAGAGAAGCATTATCCGCGCTGGACCGTGTACAAGTCTTCTGGAATGGAGCGTCATAGAGGGTGAGAATCCCGTCTTTGACACGGACTACCAGGGC-TATGTGATGTGCTCTCAAAGAGTCGAGTTGTTTGGGAATGCAGCTCAAAATGGGTGGTAAATTCCATCTAAAGCTAAATATTGGCGAGAGACCGATAGCGAACAAGTACCGTGAGGGAAAGATGAAAAGAACTTTGGAAAGAGAGTTAAACAGTACGTGAAATTGCTGAAAGGGAAACGCTTGAAGTCAGTCGCGTTGGCCAGGGATCAA-CCTTGC---TTTTTTT---GCTTGGCTTACTTTCTGGTTGACGGG-TCAGCATCAATTTTGACTGGTGG-AAAAAGTTCAAGGGAATGTGGCAT-CTTTGGATGTGTTATAGCCTTTGTTCACATACATCGGTTGGGATTGAGGAACTCAGCACGCCGAAAGGCCGGG---TTTTAAACCACGTACGTGCTTAGGATGCTGGCATAATGGCTTTAATCGACCCGTCTTGAAACACGGACCAAGGAGTCTAACATGCCTGCGAGTGTTTGGGTGGAAAACCCAAGTGCGTAATGAAAGTG-AAGGTTGAGATCCCTGTCGTGGGGAGCATTGACGCCCAGATCTGACCTTTTGTGACGATTCTGCGGTAGAGCATGT---------------------------------------------------------------------------------------------------------------------------------------------------------------------------------------------------------------------------------------------------------------------------------------------------------------------------------------------------------------------------------------------------------------------------------------------------------------------------------------------------------------------------------------------------------------------------------------------------------------------------------------------------------------------------------------------------------------------------------------------------------------------------------------------------------------------------------------------------------------------------------------------------------------------------------------------------------------------------------------------------------------------------------------------------------------------------------------------------------------------------------------------------------------------------------------CGCGAGCACGCTCTCCTTGCTTTCACCCTTGGTGTGCGGCAACTTATTGTTGCCGTCAACAAGATGGACACTACTAAAGTAAGAAACGT------TTATAGTTAAGCTTCTTCCACATTCTCATCATCTCA-TATAGTGGAGCGAGGACCGTTTCAACGAAATCATCAAGGAGACGTCAACCTTCATCAAGAAGGTTGGTTACAACCCGAAGGCCGTTGCCTTTGTCCCCATTTCGGGCTGGCATGGTGACAACATGTTGGAGGAGTCCACTAAGTAAGCATTC--TCGTATCGCCTCGATCGCCCTGTCGCTCATGTT-TACTT-AGCATGCCTTGGTACAAGGGCTGGACCAAGGAGACCAAGGCTGGTTCCGTCAAAGGAAAGACCCTCCTTGATGCTATTGATGCCATCGAGCCCCCCGTCCGACCTTCCGACAAACCCCTCCGTCTCCCCCTCCAGGATGTCTACAAAATTGGCGGTATCGGAACTGTGCCCGTCGGTCGTGTTGAAACTGGTATCATCAAGGCCGGAATGGTCGTCACTTTCGCTCCCTCCAACGTCACCACTGAAGTCAAGTCCGTCGAAATGCATCACGAACAGCTGGAGCAGGGTAACCCTGGTGACAATGTCGGCTTCAATGTCAAGAACGTGTCAGTGAAGGATATTCGTCGTGGCAACGTGGCCTCCGACTCCAAGAACGATCCCGCCAAGGAGGCGGCTTCTTTCAATGCACAGGTCATCGTCCTCAACCACCCTGGTCAAATTGGTGCTGGTTACGCCCCAGTTCTAGACTGTCACACCGCCCACATCGCCTGCAAGTTTGCCGAGCTCATTGAGAAGATCGATCGTCGTACTGGTAAATCCATTGAAAACTCGCCCAAATTCGTCAAGTCAGGTGATGCCGCCATCGTCAAGCTCGTTTCTAGCAAGCCAATG

>TWO1194_Laccaria_THAI5_THAILAND_KU685748_KU685899__KU686149

CCTGA-TGTGGCTGTTAGCTGGC-TTTTTGAAGCATGTGCTCGC-CCGTCATCTTT-ATCTCT--CCA-CCTGTGCACCTTTTGTAGTCTTT-GAATAACTCTCGAGGCAA-CTCGGATT----TTAG-GATCGACGTGC---TGT---ACAAGTCGACTTTCCTTTCATTTTC-AGGACTATGTTTT----T--A-TACACACCCAAAGTATGTTTATAGAATGTCGTT-AATAGGAAT---TTGTTTCCTATTTTAAAACTATACAACTTTCAGCAACGGATCTCTTGGCTCTCGCATCGATGAAGAACGCAGCGAAATGCGATAAGTAATGTGAATTGCAGAATTCAGTGAATCATCGAATCTTTGAACGCACCTTGCGCTCCTTGGTATTCCGAGGAGCATGCCTGTTTGAGTGTCATTAAA-TTCTCAACCTTTCCAGC--------T-TTTATTAGCTTGG-TTTAGGCTTGGA-TGT-GGGGG--TTGCAGGCTTCAAT---------AATGAGGTCAGCTCTCCTTAAATGCATTAGTGGAACTTTTGTAGACCGTCTA-TTGGTGTGAT-AATTATCTACGCCGTGGAT-RTGAAACAG-CTTTAT---AAAGTT-CAGCTTCC-AACTGTCC---ATTGACTTGGACAA-----TTTTGACAA-TTTGAAAGCTCAAATTTAAAATCTGGCAGT-CTTTGGCTGT-CCGAGTTGTAATCTAGAGAAGCATTATCCGCGTTGGACCGTGTACAAGTCTCCTGGAATGGAGCGTCATAGAGGGTGAGAATCCCGTCTTTGACACGGACTGCCAGGGC-TATGTGATGTGCTCTCAAAGAGTCGAGTTGTTTGGGAATGCAGCTCAAAATGGGTGGTAAATTCCATCTAAAGCTAAATATTGGCGAGAGACCGATAGCGAACAAGTACCGTGAGGGAAAGATGAAAAGAACTTTGGAAAGAGAGTTAAACAGTACGTGAAATTGCTGAAAGGGAAACGCTTGAAGTCAGTCGCGTTGGCCAGGGATCAA-CCTTGC----TTCTTT---GCTTGGCTTACTTTCTGGTTGACGGG-TCAGCATCAATTTTGACTGTTGG-AAAAAGTTCAAGGGAATGTGGCAT-CTTCGGATGTGTTATAGCCTTTGTTCGCATACATCAGTTGGGATTGAGGAACTCAGCACGCCGAAAGGCCGGG---TTTTAAACCACGTACGTGCTTAGGATGCTGGCATAATGGCTTTAATCGACCCGTCTTGAAACACGGACCAAGGAGTCTAACATGCCTGCGAGTGTTTGGGTGGAAAACCCGAGTGCGTAATGAAAGTG-AAAGTTGAGATCCCTGTCGTGGGGAGCATCGACGCCCGGATCTGACCTTTTGTGACGATTCCGCGGTAGAGCATGT----------------------------------------------------------------------------------------------------------------------------------------------------------------------------------------------------------------------------------------------------------------------------------------------------------------------------------------------------------------------------------------------------------------------------------------------------------------------------------------------------------------------------------------------------------------------------------------------------------------------------------------------------------------------------------------------------------------------------------------------------------------------------------------------------------------------------------------------------------------------------------------------------------------------------------------------------------------------------------------------------------------------------------------------------------------------------------------------------------------------------------------------------------------------------------------GTGAGCACGCTCTCCTCGCTTTCACCCTCGGTGTGCGGCAACTCATCGTTGCCGTCAACAAGATGGACACCACTAAGGTAAGAAACGTTTCAT-TTCTAGATTGGCTTCT-GCACATTCTCATTATCTCA-TATAGTGGAGTGAGGACCGTTTCAACGAAATCATCAAGGAGACATCAACCTTCATTAAGAAGGTTGGTTACAACCCTAAGGCCGTTGCCTTTGTCCCTATTTCCGGCTGGCATGGTGACAACATGTTGGAGGAGTCCTCGAAGTAAGCATTC--TCGTATCGTCATGGTTGCCCTATCGCTCATGTTACACTC-AGCATGCCTTGGTACAAGGGCTGGACCAAGGAGACCAAGGCTGGTGTCGTTAAAGGCAAAACCCTCCTTGATGCTATTGATGCTATTGAGCCTCCCGTCCGACCTTCCGACAAACCCCTCCGTCTCCCTCTCCAGGATGTCTACAAAATTGGCGGTATCGGAACTGTGCCCGTCGGTCGTGTTGAGACTGGTATCATTAAGGCCGGAATGGTTGTCACTTTTGCTCCCTCCAACGTCACTACTGAAGTCAAGTCCGTCGAAATGCATCACGAACAGCTCGAGCAGGGTAACCCTGGTGACAATGTCGGTTTCAACGTCAAGAACGTGTCAGTGAAGGATATTCGTCGTGGCAACGTGGCCTCAGACTCCAAGAACGATCCCGCCAAGGAAGCGGCCTCTTTCAATGCACAGGTCATCGTCCTCAACCACCCTGGTCAAATTGGTGCTGGTTACGCTCCGGTTCTCGACTGTCACACCGCCCACATCGCCTGCAAGTTCGCCGAGCTCATCGAGAaGATTGATCGTCGTACGGGTAAATCTATCGAAAaCTCGCCCAAATTCGTCAAGTCAGgTGATGCCGCTATCGTCAAaCTCATTCCCAGCAaGCCTATG

>TWO1200_Laccaria_THAI3_ThailandAUSTRALIA_KU685743_KU685894_KU686040_ef1a

CCTGA-TGTGGCTGTTAGCTGGC-TTTTAGAGGCATGTGCTCGC-CCGTCATCTTT-ATCTCT--CCA-CTTGTGCACCTTTTGTAGTG-TT-GAATACCCCTCGAGGCAA-CTCGGATT----TTAG-GATTGCCGTGC---TGY---ACAAGTCGGCTTTTCTTTCATTTTC-AACGCTATGTTTT---CT--A-TATACA-CCAAAGTATGTTTAAAGAACGTCATC-AATAGGAAC---TTGTTTTCTA--TAAAAACTATACAACTTTCAGCAACGGATCTCTTGGCTCTCGCATCGATGAAGAACGCAGCGAAATGCGATAAGTAATGTGAATTGCAGAATTCAGTGAATCATCGAATCTTTGAACGCACCTTGCGCTCCTTGGTATTCCGAGGAGCATGCCTGTTTGAGTGTCATTAAA-TTCTCAACCTTTCCAGC--------T-TTTATTAGTTTGG--TCAGGCTTGGA-TGT-GGGGG--TTGCAGGCTTT-ATTTACTT---TATGAGGTCTGCTCTYCTGAAATGCATTAGCGGAACTTTTGTGGACCGTCTA-TTGGTATGAT-AATTATCTACACCGTAGAT-GTAAAGCAT-TCTTAT---AGAGTT-CAGCTTCT-AACTGTCC---ATTGACTTGGACAA-----TTTTAATGA--TTGAAAGCTCAAATTTAAAATCTGGCAGT-CTTTGGCTGT-CCGAGTTGTAATCTAGAGAAGCATTATCCGCGCTGGACCGTGTACAAGTCTCCTGGAATGGAGCGTCATAGAGGGTGAGAATCCCGTCTTTGACACGGACTGCCAGGGCTTTTGTGATGTGCTCTCAAAGAGTCGAGTTGTTTGGGAATGCAGCTCAAAATGGGTGGTAAATTCCATCTAAAGCTAAATATTGGCGAGAGACCGATAGYGAACAAGTACCGTGAGGGAAAGATGAAAAGAACTTTGGAAAGAGAGTTAAACAGTACGTGAAATTGCTGAAAGGGAAACGCTTGAAGTCAGTCGCGTTGGCCAGGGATCAA-CCTTGC---TTTTTTT---GCTTGGCTTATTTTCTGGTCAATGGG-TCAGCATCAATTTTGACTGGTGG-AAAAAGTTCAAGGGAATGTGGCATCCTTTGGATGTGTTATAACCTTTGTTCGCATACATTAGTTGGGATTGAGGAACTCAGCACGCCGAAAGGCCGGG---TTATTAACCACGTACGTGCTTAGGATGCTGGCATAATGGCTTTAATCGACCCGTCTTGAAACACGGACCAAGGAGTCTAACATGCGTGCGAGTGTTTTGGTGAAAAACCAGAGCGCATAATGAAAGTG-AAAGTTGAGATCCCTGTCGTGGGGAGCATCGACGCCCAGATCTGACCTTTTGTGACGATTCTGCGGTAGAGCATGTTTTCCGCATGCTTTTCCGAAAGTTGACTAAGGATGTTTATCGTTATCTACAAAAGGTTGCTGAGCTTTGCCCAAGTGCC-----ATGAGATATGCTGA-CCCCTCT---TCTTCAGTGCGTCGAGACCCACAAGGAGTTCAATCTTGCTCTTGCAGTGAAGCACCAAACCATCACAAACGGCCTCAAATACTCTCTGGCGACAGGTAACTGGGGAGATCAGAAGAAGTCAATGTCTTCCAAGGCAGGGGTATCTCAGGTCTTGAACAGATATACATATGCATCCACTCTGTCACATCTTCGTCGATGTAACACTCCTCTAGGACGGGAAGGCAAGATCGCCAAACCTCGTCAGTTGCACAATACTCATTGGGGTATGGTGTGTCCTGCGGAAACTCCTGAAGGTCAAGCCTGYGGTCTTGTCAAGAACCTCGCTCTTATGGCRTGCATATCAGTCGGGTCTTACTCCGCACCCGTTATTGARTTTTTGGAGGAGTGGGGATTGGAGTCATTGGAAGAGAACGCGCACTCATCGACACCTTGCACAAAAGTTTTCGTGAACGGTGTTTGGATGGGTGTGCATCGCGATCCTGCCAACTTGGTGAAGACGATAAAAAARTTGAGAAGGAAAGATGACATCAGCCCAGAAGTGTCAGTTGTGCGAGACATYAGGGAGAAGGAATTGAGGTTGTACACCGATGCTGGACGTGTTTGTCGACCACTCTTCATCGTCGAGAACCAACAACTGGCACTTCAGAAGAAGCATGTCAAATGGCTCAGCAACGGCCTCAATGATGAYGGGGACGAGTATAAATGGGAACATTTGGTAAAGGGTGGCATCATTGAGTTACTGGATGCGGAGGAAGAGGAAACAGTGATGATATCTATGACTCCTGAAGATCTTGAAAATTCTCGCCTACAGCAAAGTGGTGTTGATCCTCACGCGAACGACGGCGAGTTTGATCCAGCAGCTCGRTTGAAGGCTGGCACTCACGCACACACATGGACGCATTGCGAAATTCATCcTAGCATGATtCTtGGCATTTGTGCcAGCATTATCccTTTCCCTGACCAT-------------------------------------------------------AAAGATGGCCAGACCCGCGAGCACGCTCTCCTCGCTTTCACGCTCGGTGTGCGGCAACTCATCGTTGCTGTCAACAAGATGGACACCACTAAGGTAAGAAATGCTTCAC-ATATAGTCTGGCTTTTTGCACATTCTCATCATTTCA-AATAGTGGAGTGAGGACCGTTTCAACGAAATTATTAAGGAGACATCAACCTTTATCAAGAAGGTTGGTTACAACCCCAAGGCCGTTGCCTTTGTCCCTATTTCTGGTTGGCACGGTGACAACATGTTGGAGGARTCCACCAAGTAAGCATTC--TCGTATCGTTATGATCGCCCTGTTGCTCATATTTCACTC-AGCATGCCTTGGTACAAGGGATGGACCAAGGAGACCAAGGCTGGTGTCGTCAAGGGCAAGACCCTCCTCGATGCTATTGATGCCATTGAGCCCCCCGTTCGACCTTCCGACAAACCCCTCCGTCTTCCCCTCCAGGATGTCTACAAAATTGGCGGTATTGGAACCGTGCCTGTCGGTCGTGTTGAGACTGGTATCATCAAAGCGGGAATGGTCGTCACTTTCGCTCCCTCYAACGTCACCACTGAAGTCAAGTCTGTTGAAATGCACCACGAACAGCTCGAGCAGGGTAACCCTGGTGACAATGTCGGTTTCAACGTCAAGAACGTGTCAGTGAAGGATATTCGACGTGGCAACGTGGCCTCCGACTCCAAGAACGATCCCGCCAAGGAAGCGGCCTCTTTCAATGCACAGGTCATCGTCCTCAACCACCCTGGTCAAATCGGTGCTGGCTACGCCCCGGTTCTCGACTGTCACACCGCCCACATCGCCTGCAAGTTCGCTGAGCTCATCGAGAAGATTGATCGTCGTACGGGTAAATCCATTGAAAACAACCCCAAATTCGTCAAGTCGGGTGATGCCTGCATCGTCAAACTCGTTCCCAGCAAGCCTATG

>TWO319_Laccaria_montana __DQ149862___

CCTGA-TGTGGCTGTTAGCTGGC-TTTTCGAAGCATGTGCTCGT-CCGTCATCTTTAATCTCT--CCA-CCTGTGCACATTTTGTAGTC-TT-GGATAACTCTCGAGGCAA-CTCGGATT----TTAG-GATCGCCGTGC----------------TGCTTTCCTTTCATTTCC-AAGACTATGTTTT----T--A-TATACA-CCAAAGTATGTTTAAAGAATGTCATG-AATGGGAAC---TTGTTTCCTA--T-AAAATTATACAACTTTCAGCAACGGATCTCTTGGCTCTCGCATCGATGAAGAACGCAGCGAAATGCGATAAGTAATGTGAATTGCAGAATTCAGTGAATCATCGAATCTTTGAACGCACCTTGCGCTCCCTGGTATTCCGAGGAGCATGCCTGTTTGAGTGTCATTAAA-TTCTCAA-CCTTCCAGC--------T-TTTATTGGCTTGG--TTAGGCTTGGA-TGT-GGGGG--TTGTGGGCTTC-AT-----T---AATGAGGTCAGCTCTCCTTAAATGCATTAGCGGAACTTTTGTGGACCGTCTA-TTGGTGTGAT-AATTATCTACGCTGTGGAT-GTGAAGCAT-ATTTAT---GAAGTT-CAGCTTCT-AATCGTCC---ATTGACTTGGACAA----TTTTTGACAA-TTTGA-------------------------------------------------------------------------------------------------------------------------------------------------------------------------------------------------------------------------------------------------------------------------------------------------------------------------------------------------------------------------------------------------------------------------------------------------------------------------------------------------------------------------------------------------------------------------------------------------------------------------------------------------------------------------------------------------------------------------------------------------------------------------------------------------------------------------------------------------------------------------------------------------------------------------------------------------------------------------------------------------------------------------------------------------------------------------------------------------------------------------------------------------------------------------------------------------------------------------------------------------------------------------------------------------------------------------------------------------------------------------------------------------------------------------------------------------------------------------------------------------------------------------------------------------------------------------------------------------------------------------------------------------------------------------------------------------------------------------------------------------------------------------------------------------------------------------------------------------------------------------------------------------------------------------------------------------------------------------------------------------------------------------------------------------------------------------------------------------------------------------------------------------------------------------------------------------------------------------------------------------------------------------------------------------------------------------------------------------------------------------------------------------------------------------------------------------------------------------------------------------------------------------------------------------------------------------------------------------------------------------------------------------------------------------------------------------------------------------------------------------------------------------------------------------------------------------------------------------------------------------------------------------------------------------------

>TWO591_Laccaria_montana __DQ149865___

CCTGA-TGTGGCTGTTAGCTGGC-TTTTCGAAGCATGTGCTCGT-CCGTCATCTTTAATCTCT--CCA-CCTGTGCACATTTTGTAGTC-TT-GGATAACTCTCGAGGCAA-CTCGGATT----TTAG-GATCGCCGTGC----------------TGCTTTCCTTTCATTTCC-AAGACTATGTTTT----T--A-TATACA-CCAAAGTATGTTTAAAGAATGTCATG-AATGGGAAC---TTGTTTCCTA--T-AAAATTATACAACTTTCAGCAACGGATCTCTTGGCTCTCGCATCGATGAAGAACGCAGCGAAATGCGATAAGTAATGTGAATTGCAGAATTCAGTGAATCATCGAATCTTTGAACGCACCTTGCGCTCCTTGGTATTCCGAGGAGCATGCCTGTTTGAGTGTCATTAAA-TTCTCAA-CCTTCCAGC--------T-TTTATTGGCTTGG--TTAGGCTTGGA-TGT-GGGGG--TTGTGGGCTTC-AT-----T---AATGAGGTCAGCTCTCCTTAAATGCATTAGCGGAACTTTTGTGGACCGTCTA-TTGGTGTGAT-AATTATCTACGCTGTGGAT-GTGAAGCAT-ATTTAT---GAAGTT-CAGCTTCT-AATCGTCC---ATTGACTTGGACAA----TTTTTGACAA-TTTGA-------------------------------------------------------------------------------------------------------------------------------------------------------------------------------------------------------------------------------------------------------------------------------------------------------------------------------------------------------------------------------------------------------------------------------------------------------------------------------------------------------------------------------------------------------------------------------------------------------------------------------------------------------------------------------------------------------------------------------------------------------------------------------------------------------------------------------------------------------------------------------------------------------------------------------------------------------------------------------------------------------------------------------------------------------------------------------------------------------------------------------------------------------------------------------------------------------------------------------------------------------------------------------------------------------------------------------------------------------------------------------------------------------------------------------------------------------------------------------------------------------------------------------------------------------------------------------------------------------------------------------------------------------------------------------------------------------------------------------------------------------------------------------------------------------------------------------------------------------------------------------------------------------------------------------------------------------------------------------------------------------------------------------------------------------------------------------------------------------------------------------------------------------------------------------------------------------------------------------------------------------------------------------------------------------------------------------------------------------------------------------------------------------------------------------------------------------------------------------------------------------------------------------------------------------------------------------------------------------------------------------------------------------------------------------------------------------------------------------------------------------------------------------------------------------------------------------------------------------------------------------------------------------------------------------

>DKP303_Laccaria_violaceotincta_INDIA_MK141034___

CCTGA-TGTGGCTGTTAGCTGGC-TTTTCGAAGCATGTGCTCGC-CCATCATCTTT-ATCTCT--CCA-CCTGTGCACCTTTTGTAGTC-TT-GAATACCTCTCGAGGCAA-CTCGGATT----TTAG-GATTGCCGTGCTTAT-TACACAAAGTCGGCTTTTCTTTCATTTTC-AAGACTATGTTTT---CT--ATTACACA-CCAAAGTATGTTTAAAGAATGGCATC-AATAGGAAC---TTGTTTCCTA--TAAAAACTATACAACTTTCAGCAACGGATCTCTTGGCTCTCGCATCGATGAAGAACGCAGCGAAATGCGATAAGTAATGTGAATTGCAGAATTCAGTGAATCATCGAATCTTTGAACGCACCTTGCGCTCCTTGGTATTCCGAGGAGCATGCCTGTTTGAGTGTCATTAAA-TTCTCAACCTTTCCAGC--------T-TTTATTAGTTTGG--TCAGGCTTGGA-TGT-GGGGG--TTGCAGGCTTC-ATTTACTTT--TATGAGTTCTGCTCTCCTTAATTGCATTAGCGGAACTTTTGTGGACCGTCTA-TTGGTGTGAT-AATTATCTACACCGTGGATAGTAAAGCAA-TTTTAT---AAAGTT-CAGCTTCT-AACTGTCC---ATTGAATTGGACAA-----TTTTGACAA-TTTGA-------------------------------------------------------------------------------------------------------------------------------------------------------------------------------------------------------------------------------------------------------------------------------------------------------------------------------------------------------------------------------------------------------------------------------------------------------------------------------------------------------------------------------------------------------------------------------------------------------------------------------------------------------------------------------------------------------------------------------------------------------------------------------------------------------------------------------------------------------------------------------------------------------------------------------------------------------------------------------------------------------------------------------------------------------------------------------------------------------------------------------------------------------------------------------------------------------------------------------------------------------------------------------------------------------------------------------------------------------------------------------------------------------------------------------------------------------------------------------------------------------------------------------------------------------------------------------------------------------------------------------------------------------------------------------------------------------------------------------------------------------------------------------------------------------------------------------------------------------------------------------------------------------------------------------------------------------------------------------------------------------------------------------------------------------------------------------------------------------------------------------------------------------------------------------------------------------------------------------------------------------------------------------------------------------------------------------------------------------------------------------------------------------------------------------------------------------------------------------------------------------------------------------------------------------------------------------------------------------------------------------------------------------------------------------------------------------------------------------------------------------------------------------------------------------------------------------------------------------------------------------------------------------------------------------

>ASIS24249_Laccaria_murina_KOREA_MG519552_MG519592_MG551625_MG551658

CCTGA-TGTGGCTGTTAGCTGGC-TTTTCGAAGCATGTGCTCGT-CCGTCATCTTT-ATATCT--CCA-CCTGTGCACATTTTGTAGTC-TT-GGATACCTCTCGAGGCAA-CTCGGATT----TTAG-GATCGC------------------ATCAGCTTTCCTTTCATTTCC-AAGACTATGTTTT----T--A-TATACA-CCAAAGTATGTTTAAAGAATGTCATC-AATGGGAACT--TTGTTTCCTA--T-AAAATTATACAACTTTCAGCAACGGATCTCTTGGCTCTCGCATCGATGAAGAACGCAGCGAAATGCGATAAGTAATGTGAATTGCAGAATTCAGTGAATCATCGAATCTTTGAACGCACCTTGCGCTCCTTGGTATTCCGAGGAGCATGCCTGTTTGAGTGTCATTAAA-TTATCAA-CCTTCCAGC--------T-TTTATTAGCTTGG--TTAGGCTTGGA-TGT-GGGGG--TTGCAGGCTTC-AT-----T---GATGAGGTCAGCTCTCCTTAAATGCATTAGCGGAACTTTTGTGGACTGTCTA-TTGGTGTGAT-AATTATCTACGCCGTGGAT-GTGAAGCAG-CTTTAT---GGAGTT-CAGCTTCT-AACCGTCC---ATTGACTTGGACAA-----TTTTGACAA-TTTG-AAGCTCAAATTTAAAATCTGGCAGT-CTTTGGCTGT-CCGAGTTGTAATCTAGAGAAGTATTATCCGCGCTGGACCGTGTACAAGTCTCCTGGAATGGAGCGTCATAGAGGGTGAGAATCCCGTCTTTGACACGGACTGCCAGGGC-TTTGTGATGTGCTCTCAAAGAGTCGAGTTGTTTGGGAATGCAGCTCAAAATGGGTGGTAAATTCCATCTAAAGCTAAATATTGGCGAGAGACCGATAGCGAACAAGTACCGTGAGGGAAAGATGAAAAGAACTTTGGAAAGAGAGTTAAACAGTACGTGAAATTGCTGAAAGGGAAACGCTTGAAGTCAGTCGCGTTGGCCAGGGATCAA-CCTTGC----TTTTTT---GCTTGGCTTACTTTCTGGTCGATGGG-TCAGCATCAATTTTGACTGGTGG-AAAAAGTTCAAGGGAATGTGGCAT-CTTCGGATGTGTTATAGCCTTTGTTCGCATACATCGGTTGGGATTGAGGAACTCAGCACGCCGCAAGGCCGGG---TTTTTAACCACGTACGTGCTTAGGATGCTGGCATAATGGCTTTAATCGACCCGTCTTGAAACACGGACCAAGGAGTCTAACATGCCTGCGAGTGTTTGGGTGAAAAACCCGAGCGCGTAATGAAAGTG-AAAGTTGAGATCCCTGTCATGGGGAGCATTGACGCCCGGATCTGACCTTTTGTGACGATTCTGCGGTAGAGCATGT--------------------------------------------------------------------------------------------------------------------------------------------------------------------------------------------------------------------------------------------------------------------------------------------------------------------------------------------------------------------------------------------------------------------------------------------CTCGCCCTTATGGCGTGCATATCAGTCGGATCTTACTCCGCGCCCGTTATCGAGTTTTTGGAGGAGTGGGGATTGGAGTCTTTGGAAGAGAACGCACACTCATCGACACCTTGCACAAAAGTTTTTGTGAACGGTGTCTGGATGGGTGTGCATCGCGATCCTGCCAATTTGGTGAAGACGATAAAGAAATTGAGAAGGAAAGACGACATCAGCCCAGAAGTGTCAGTTGTGCGAGACATCAGGGAGAAGGAATTGAGGTTGTACACCGATGCTGGACGTGTTTGTCGACCACTCTTCATCGTCGAGAACCAACAGCTGGCACTTCAGAAGAAGCACGTCAAATGGCTCAGTAACGGCCTCAACGATGATGGCGACGAGTACAAATGGGAACATCTTGTAAAGGGTGGCATCATTGAGCTACTGGATGCCGAGGAAGAAGAAACAGTGATGATATCCATGACCCCTGAAGATCTTGAAAATTCTCGCCTACAGCAAAGTGGCGTTGATCCTCACGCGAACGACGGCGAATTTGATCCAGCGGCTCGATTGAAGGCTGGCACACACGCACACACATGGTCACATTGCGAAA-----------------------------------------------------------------------------------------------------------------GGATGGCCAGACCCGCGAGCACGCTCTCCTCGCTTTCACCCTCGGTGTGCGGCAACTCATCGTTGCCGTCAACAAGATGGACACCACTA-----------------------------------------------------------AGTGGAGCGAGGATCGTTTCAACCAAATCATCAAGGAGACGTCAACCTTSATCAAGAAGGTTGGTTACAACCCCAAGGCCGTTGCCTTTGTCCCTATTTCCGGCTGGCACGGTGACAACATGTTGGAGGAGTCCTCT------------------------------------------------------AACATGCCTTGGTACAAGGGCTGGACCAAGGAGACCAAGGCTGGTGTCGTCAAGGGCAAGACTCTCCTCGATGCTATTGATGCCATTGAGCCCCCCGTCCGGCCTTTCAACAAACCCCTCCGCCTCCCCATCCAGGATGTCTACAAAATTGGCGGTATTGGAACTGTGCCCGTCGGTCGTGTTGAGACTGGGATCATCAAAGCCGGAATGGTCGTCACTTTCGCTCCCTCCCACGTCACCACTGAAGTCAAGTCCATCGAAATGCATCACGAA---------------------------------------------------------------------------------------------------------------------------------------------------------------------------------------------------------------------------------------------------------------------------------------------------------------------------------------------------

>SFC20120919-81_Laccaria_vinaceoavellanea_KOREA_MG519534_MG519577_MG551609_

CCTGA-TGTGGCTGTTAGCTGGC-TTTTCAAAGCATGTGCTCGC-CCATCATCTTT-ATCTCT--CCA-CCTGTGCACCTTTTGTAGTC-TT-GAATACCTCTCGAGGCAA-CTCGGATT----TTAG-GATTGYCGTGCTTTTATACACAAAGTCGGCTTTTCTTTCATTTTC-AAGACTATGTTTT---CT--A-TACACA-CCAAAGTATGTTTAAAGAACGTCATC-AATAGGAAC---TTGTTTCCTA--TAAAAACTATACAACTTTCAGCAACGGATCTCTTGGCTCTCGCATCGATGAAGAACGCAGCGAAATGCGATAAGTAATGTGAATTGCAGAATTCAGTGAATCATCGAATCTTTGAACGCACCTTGCGCTCCTTGGTATTCCGAGGAGCATGCCTGTTTGAGTGTCATTAAA-TTCTCAACCTTTCCAGC--------T-TTTATTAGTTTGG--TCAGGCTTGGA-TGT-GGGGG--TTGCAGGCTTC-AT-----T-------TAGTCTGCTCTCCTTAAATGCATTAGCGGAACTTTTGTGGACCGTCTA-TTGGTGTGAT-AATTATCTACACCGTGGAT-GTAAAGCAGTTTTTAT-AAAAAGTT-CAGCTCCT-AACTGTCC---ATTGACTTGGACAA-----TTTTGACAA-TTTG-AAGCTCAAATTTAAAATCTGGCAGT-CTTTGGCTGT-CCGAGTTGTAATCTAGAGAAGCATTATCCGCGCTGGACCGTGTACAAGTCTCCTGGAATGGAGCGTCATAGAGGGTGAGAATCCCGTCTTTGACACGGACTACCAGGGCTTTTGTGATGTGCTCTCAAAGAGTCGAGTTGTTTGGGAATGCAGCTCAAAATGGGTGGTAAATTCCATCTAAAGCTAAATATTGGCGAGAGACCGATAGCGAACAAGTACCGTGAGGGAAAGATGAAAAGAACTTTGGAAAGAGAGTTAAACAGTACGTGAAATTGCTGAAAGGGAAACGCTTGAAGTCAGTCGCGTTGGCCAGGGATCAA-CCTTGC----TTTTTT---GCTTGGCTTATTTTCTGGTCAATGGG-TCAGCATCAATTTTGACTGGTGG-AAAAAGTTCAAGGGAATGTGGCAT-CTTTGGATGTGTTATAGCCTTTGTTCGCATACATCAGTTGGGATTGAGGAACTCAGCACGCCGAAAGGCCGGG---TTTTTAACCACGTACGTGCTTAGGATGCTGGCATAATGGCTTTAATCGACCCGTCTTGAAACACGGACCAAGGAGTCTAACATGCCTGCGAGTGTTTTGGTGAAAAACCAGAGTGCATAATGAAAGTG-AAAGTTGAGATCCCTGTCGTGGGGAGCATCGACGCCCAGATCTGACCTTTTGTGACGATTCTGCGGTAGAGCATGT--------------------------------------------------------------------------------------------------------------------------------------------------------------------------------------------------------------------------------------------------------------------------------------------------------------------------------------------------------------------------------------------------------------------------------------------CTAGCTCTTATGGCATGCATATCAGTCGGGTCTTACTCCGCACCCGTTATTGAGTTTTTGGAGGAGTGGGGGTTGGAGTCATTGGAAGAGAACGCACACTCAACGACACCTTGCACAAAAGTTTTCGTGAACGGTGTCTGGATGGGTGTGCACCGTGATCCTGCCAACTTGGTGAAGACGATAAAGAAATTGAGAAGGAAAGATGACATCAGCCCAGAAGTGTCTGTTGTGCGAGACATCAGGGAGAAGGAATTGAGGTTGTACACCGATGCTGGACGTGTTTGTCGACCACTCTTCATCGTCGAGAACCAACAGTTGGCACTTCAGAAGAAGCACGTCAAGTGGCTCAGCAACGGCCTCAATGATGATGGCGACGAGTATAAATGGGAACATCTGGTAAAGGGTGGCATCATTGAGTTACTGGATGCGGAGGAAGAGGAAACAGTGATGATATCCATGACCCCTGAAGATCTTGAAAATTCTCGCCTACAGCAAAGTGGCGTTGATCCTCACGCGAACGACGGCGAGTTTGATCCAGCAGCTCGATTGAAGGCTGGCACTCACGCACACACATGGACACTTGGAGA----------------------------------------------------------------------------------------------------------------------------------------------------------------------------------------------------------------------------------------------------------------------------------------------------------------------------------------------------------------------------------------------------------------------------------------------------------------------------------------------------------------------------------------------------------------------------------------------------------------------------------------------------------------------------------------------------------------------------------------------------------------------------------------------------------------------------------------------------------------------------------------------------------------------------------------------------------------------------------------------------------------------------------------------------------------------------------------------------------

>SFC20150810-10_Laccaria_vinaceoavellanea_KOREA_MG519539_MG519580_MG551614_MG551646

CCTGA-TGTGGCTGTTAGCTGGC-TTTTCGAAGCATGTGCTCGC-CCATCATCTTT-ATCTCT--CCA-CCTGTGCACCTTTTGTAGTC-TT-GAATACCTCTCGAGGCAA-CTCGGATT----TTAG-GATTGCCGTGC-TTTATACACAAAGTCGGCTTTTCTTTCATTTTC-AAGACTATGTTTT---CT--A-TACACA-CCAAAGTATGTTTAAAGAACGTCATC-AATAGGAAC---TTGTTTCCTA--TAAAAACTATACAACTTTCAGCAACGGATCTCTTGGCTCTCGRATCGATGAAGAACGCAGCGAAATGCGATAAGTAATGTGAATTGCAGAATTCAGTGAATCATCGAATCTTTGAACGCACCTTGCGCTCCTTGGTATTCCGAGGAGCATGCCTGTTTGAGTGTCATTAAA-TTCTCAACCTTTCCAGC--------T-TTTATTAGTTTGG--TCAGGCTTGGA-TGT-GGGGG--TTGCAGGCTTC-AT-----T-------TAGTCTGCTCTCCTTAAATGCATTAGCGGAACTTTTGTGGACCGTCTA-TTGGTGTGAT-AATTATCTACACCGTGGAT-GTAAAGCAGTTTTTAT-AAAAAGTT-CAGCTCCT-AACTGTCC---ATTGACTTGGACAA-----TTTTGACAA-TTTG-AAGCTCAAATTTAAAATCTGGCAGT-CTTTGGCTGT-CCGAGTTGTAATCTAGAGAAGCATTATCCGCGCTGGACCGTGTACAAGTCTCCTGGAATGGAGCGTCATAGAGGGTGAGAATCCCGTCTTTGACACGGACTACCAGGGCTTTTGTGATGTGCTCTCAAAGAGTCGAGTTGTTTGGGAATGCAGCTCAAAATGGGTGGTAAATTCCATCTAAAGCTAAATATTGGCGAGAGACCGATAGCGAACAAGTACCGTGAGGGAAAGATGAAAAGAACTTTGGAAAGAGAGTTAAACAGTACGTGAAATTGCTGAAAGGGAAACGCTTGAAGTCAGTCGCGTTGGCCAGGGATCAA-CCTTGC----TTTTTT---GCTTGGCTTATTTTCTGGTCAATGGG-TCAGCATCAATTTTGACTGGTGG-AAAAAGTTCAAGGGAATGTGGCAT-CTTTGGATGTGTTATAGCCTTTGTTCGCATACATCAGTTGGGATTGAGGAACTCAGCACGCCGAAAGGCCGGG---TTTTTAACCACGTACGTGCTTAGGATGCTGGCATAATGGCTTTAATCGACCCGTCTTGAAACACGGACCAAGGAGTCTAACATGCCTGCGAGTGTTTTGGTGAAAAACCAGAGTGCATAATGAAAGTG-AAAGTTGAGATCCCTGTCGTGGGGAGCATCGACGCCCAGATCTGACCTTTTGTGACGATTCTGCGGTAGAGCATGT--------------------------------------------------------------------------------------------------------------------------------------------------------------------------------------------------------------------------------------------------------------------------------------------------------------------------------------------------------------------------------------------------------------------------------------------CTAGCTCTTATGGCATGCATATCAGTCGGGTCTTACTCCGCACCCGTTATTGAGTTTTTGGAGGAGTGGGGGTTGGAGTCATTGGAAGAGAACGCACACTCAACGACACCTTGCACAAAAGTTTTCGTGAACGGTGTCTGGATGGGTGTGCACCGTGATCCTGCCAACTTGGTGAAGACGATAAAGAAATTGAGAAGGAAAGATGACATCAGCCCAGAAGTGTCTGTTGTGCGAGACATCAGGGAGAAGGAATTGAGGTTGTAYACCGATGCTGGACGTGTTTGTCGACCACTCTTCATCGTCGAGAACCAACAGTTGGCACTTCAGAAGAAGCACGTCAAGTGGCTCAGCAACGGCCTCAATGATGATGGCGACGAGTATAAATGGGAACATCTGGTAAAGGGTGGCATCATTGAGTTACTGGATGCGGAGGAAGAGGAAACAGTGATGATATCCATGACCCCTGAAGATCTTGAAAATTCTCGCCTACAGCAAAGTGGCGTTRATCCTCACGCGAACGACGGCGAGTTTGATCCAGCAGCTCGATTGAAGGCTGGCACTCACGCACACACATGGACACATTGCGARA-----------------------------------------------------------------------------------------------------------------GGATGGCCAGACCCGCGAGCACGCCCTCCTCGCTTTCACGCTCGGTGTGCGGCAACTCATCGTTGCTGTCAACAAGATGGACACTACTAAGGTAAGAAATGTTTCAC-TTATAGTCAGGCTTTTTGCATATTCTTATC---TCA-AATAGTGGAGCGAGGACCGTTTCAACGAAATCATCAAGGAGACGTCAACCTTCATCAAGAAGGTTGGTTACAACCCCAAGGCCGTTGCCTTTGTCCCTATTTCCGGTTGGCACGGTGACAACATGTTGGAGGAATCCACAAAGTAAGCGTTC--TCGTACCGTCATGAACGCCCTGTCGCTCATATTACATTC-AGCATGCCTTGGTACAAGGGCTGGACCAAGGAGACCAAGGCTGGTGTCGTCAAGGGCAAGACCCTCCTCGATGCTATTGATGCCATTGAGCCCCCCGTTCGACCTTCCGACAAACCCCTTCGTCTTCCCCTCCAGGATGTYTACAAAATCGGCGGTATTGGAACCGTACCTGTCGGTCGTGTTGAGACTGGTATCATCAAAGCGGGAATGGTCGTCACTTTCGCACCCTCYAACGTCACCACTGAAGTCAAGTCTGTTGAAATGCATCACGAACAGCTCGAG------------------------------------------------------------------------------------------------------------------------------------------------------------------------------------------------------------------------------------------------------------------------------------------------------------------------------------------

>TPML20120912-40_Laccaria_araneosa_KOREA_MG519548_MG519588_MG551621_MG551654

CCTGA-TG-GGCTGTTAGCTGGC-TTTTCGAAGCATGTGCTCGT-CTGTCATCTTT-ATCTCT--CCA-CCTGTGCACATTTTGTAGTC-TT-GGATACCTCTCGAGGCAA-CTCGGATT----TTAG-GATCGCTGTGC---TGT---ACAAGTCGGCTTTTCTTTCATTTCC-AAGACTATGTTTT----T--ATTATACA-CCAAAGTATGTTTAAAGAATGTCATC-AATAGGAAC---TTGTTTCCTA--T-AAAAATATACAACTTTCAGCAACGGATCTCTTGGCTCTCGCATCGATGAAGAACGCAGCGAAATGCGATAAGTAATGTGAATTGCAGAATTCAGTGAATCATCGAATCTTTGAACGCACCTTGCGCTCCTTGGTATTCCGAGGAGCATGCCTGTTTGAGTGTCATTAAA-TTCTCAA-CCTTCCAAC--------T-TTTATTAGCTTGG--TTAGGCTTGGA-TGT-GGGGG--TTGTGGGCTTC-AT-----T---AATGAGGTCGGCTCTCCTTAAATGCATTAGCGGAACTTTTGTGGACCGTCTA-TTGGTGTGAT-AATTATCTACGCCGTGGAT-GTGAAGCAG-CTTTAT---GAAGTT-CTGCTTCT-AACCGTCC---ATTGACTTGGACAA-----CTTTGACAA-TTTG-AAGCTCAAATTTAAAATCTGGCAGT-CTTTGGCTGT-CCGAGTTGTAATCTAGAGAAGTATTATCCGCGCTGGACCGTGTACAAGTCTCCTGGAATGGGGCGTCATAGAGGGTGAGAATCCCGTCTTTGACACGGACTGCCAGGGC-TTTGTGATATGCTCTCAAAGAGTCGAGTTGTTTGGGAATGCAGCTCAAAATGGGTGGTAAATTCCATCTAAAGCTAAATATTGGCGAGAGACCGATAGCGAACAAGTACCGTGAGGGAAAGATGAAAAGAACTTTGGAAAGAGAGTTAAACAGTACGTGAAATTGCTGAAAGGGAAACGCTTGAAGTCAGTCGCGTTGGCCAGGGATCAA-CCTTGC----TTTTTT---GCTTGGCTTACTTTCTGGTTGATGGG-TCAGCATCAATTTTGACTGGTGG-AAAAAGTTCAAGGGAATGTGGCAT-CTTCGGATGTGTTATAGCCTTTGTTCACATACATTGGTTGGGATTGAGGAACTCAGCACGCCGCAAGGCCGGG---TTTTTAACCACGTACGTGCTTAGGATGCTGGCATAATGGCTTTAATCGACCCGTCTTGAAACACGGACCAAGGAGTCTAACATGCCTGCGAGTATTTGGGTGAAAAACCCGAGTGCGTAATGAAAGTG-AAAGTTGAGATCCCTGTCGTGGGGAGCATTGACGCCCGGATCTGACCTTTTGTGACGATTCTGCGGTAGAGCATGT--------------------------------------------------------------------------------------------------------------------------------------------------------------------------------------------------------------------------------------------------------------------------------------------------------------------------------------------------------------------------------------------------------------------------------------------CTCGCCCTTATGGCGTGCATATCAGTCGGGTCTTACTCCGCGCCCGTTATCGAGTTTTTGGAGGAGTGGGGAYTGGAGTCATTGGAAGAGAACGCGCACTCATCAACACCCTGCACAAAAGTTTTTGTGAACGGTGTGTGGATGGGTGTGCATCGCGATCCTGCCAATTTGGTGAAGACGATAAAGAAGTTGAGAAGGAAAGACGACATTAGCCCAGAAGTGTCAGTTGTGCGAGACATCAGGGAGAAGGAATTGAGGTTGTACACCGATGCTGGACGTGTTTGTCGACCACTCTTCATCGTTGAGAACCAACAGCTGGCACTTCAGAAGAAGCACGTCAAATGGCTCAGTAACGGCCTCAACGATGATGGCGATGAATACAAATGGGAACATCTGGTAAAGGGTGGCATCATTGAGTTACTGGATGCTGAAGAAGAGGAAACAGTGATGATATCCATGACCCCCGAAGATCTTGAAAATTCTCGCCTACAGCAAAGTGGCGTTGATCCTCACGCGAACGACGGCGAGTTTGATCCAGCAGCTCGATTGAAGGCTGGCACTCACGCACACACATGGACACATTGCGAAA-----------------------------------------------------------------------------------------------------------------GGATGGCCAGACCCGCGAGCACGCTCTCCTCGCTTTCACCCTCGGTGTGCGGCAACTCATCGTTGCTGTCAACAAGATGGACACCACTAAGGTAAGAAACATTTCAC-TTATAGTAAGGCTCCTTGCACATTCTCATCATCTCA-TRTAGTGGAGCGAGGACCGTTTCAACGAAATCATCAAGGAGACGTCAACCTTCATCAAGAAGGTTGGTTACAACCCCAAGGCCGTTGCCTTTGTCCCTATTTCCGGGTGGCACGGCGACAACATGTTGGAGGAGTCCGCCAAGTAAGCAGTC--TCGTATTGTCATGATCGCCCCGTCGCTCATGTTGCACTC-AGCATGCCTTGGTACAAGGGCTGGACCAAGGAGACCAAGGCTGGTGTCGTCAAGGGCAAGACCCTCCTCGATGCTATTGATGCCATTGAGCCCCCTGTCCGACCTTCCGACAAACCCCTCCGTCTCCCCCTCCAGGATGTTTACAAAATTGGTGGTATCGGAACTGTGCCCGTCGGTCGTGTTGAGACTGGTATCATCAAGGCCGGAATGGTCGTCACTTTCGCTCCCTCTAATGTCACCACTGAAGTCAAGTCCGTCGAAATGCATCACGAACAGCTCGAG------------------------------------------------------------------------------------------------------------------------------------------------------------------------------------------------------------------------------------------------------------------------------------------------------------------------------------------

>SFC20130917-21_Laccaria_araneosa_KOREA_MG519549_MG519589_MG551622_MG551655

CCTGA-TG-GGCTGTTAGCTGGC-TTTTCGAAGCATGTGCTCGT-CTGTCATCTTT-ATCTCT--CCA-CCTGTGCACATTTTGTAGTC-TT-GGATACCTCTCGAGGCAA-CTCGGATT----TTAG-GATCGCTGTGC---TGT---ACAAGTCGGCTTTTCTTTCATTTCC-AAGACTATGTTTT----T--ATTATACA-CCAAAGTATGTTTAAAGAATGTCATC-AATAGGAAC---TTGTTTCCTA--T-AAAAATATACAACTTTCAGCAACGGATCTCTTGGCTCTCGCATCGATGAAGAACGCAGCGAAATGCGATAAGTAATGTGAATTGCAGAATTCAGTGAATCATCGAATCTTTGAACGCACCTTGCGCTCCTTGGTATTCCGAGGAGCATGCCTGTTTGAGTGTCATTAAA-TTCTCAA-CCTTCCAAC--------T-TTTATTAGCTTGG--TTAGGCTTGGA-TGT-GGGGG--TTGTGGGCTTC-AT-----T---AATGAGGTCGGCTCTCCTTAAATGCATTAGCGGAACTTTTGTGGACCGTCTA-TTGGTGTGAT-AATTATCTACGCCGTGGAT-GTGAAGCAG-CTTTAT---GAAGTT-CTGCTTCT-AACCGTCC---ATTGACTTGGACAA-----TTTTGACAA-TTTG-AAGCTCAAATTTAAAATCTGGCAGT-CTTTGGCTGT-CCGAGTTGTAATCTAGAGAAGTATTATCCGCGCTGGACCGTGTACAAGTCTCCTGGAATGGGGCGTCATAGAGGGTGAGAATCCCGTCTTTGACACGGACTGCCAGGGC-TTTGTGATATGCTCTCAAAGAGTCGAGTTGTTTGGGAATGCAGCTCAAAATGGGTGGTAAATTCCATCTAAAGCTAAATATTGGCGAGAGACCGATAGCGAACAAGTACCGTGAGGGAAAGATGAAAAGAACTTTGGAAAGAGAGTTAAACAGTACGTGAAATTGCTGAAAGGGAAACGCTTGAAGTCAGTCGCGTTGGCCAGGGATCAA-CCTTGC----TTTTTT---GCTTGGCTTACTTTCTGGTTGATGGG-TCAGCATCAATTTTGACTGGTGG-AAAAAGTTCAAGGGAATGTGGCAT-CTTCGGATGTGTTATAGCCTTTGTTCACATACATTGGTTGGGATTGAGGAACTCAGCACGCCGCAAGGCCGGG---TTTTTAACCACGTACGTGCTTAGGATGCTGGCATAATGGCTTTAATCGACCCGTCTTGAAACACGGACCAAGGAGTCTAACATGCCTGCGAGTATTTGGGTGAAAAACCCGAGTGCGTAATGAAAGTG-AAAGTTGAGATCCCTGTCGTGGGGAGCATTGACGCCCGGATCTGACCTTTTGTGACGATTCTGCGGTAGAGCATGT--------------------------------------------------------------------------------------------------------------------------------------------------------------------------------------------------------------------------------------------------------------------------------------------------------------------------------------------------------------------------------------------------------------------------------------------CTCGCCCTTATGGCGTGCATATCAGTCGGGTCTTACTCCGCGCCCGTTATCGAGTTTTTGGAGGAGTGGGGATTGGAGTCATTGGAAGAGAACGCGCACTCATCAACACCCTGCACAAAAGTTTTTGTGAACGGTGTGTGGATGGGTGTGCATCGCGATCCTGCCAATTTGGTGAAGACGATAAAGAAGTTGAGAAGGAAAGACGACATTAGCCCAGAAGTGTCAGTTGTGCGAGACATCAGGGAGAAGGAATTGAGGTTGTACACCGATGCTGGACGTGTTTGTCGACCACTCTTCATCGTTGAGAACCAACAGCTGGCACTTCAGAAGAAGCACGTCAAATGGCTCAGTAACGGCCTCAACGATGATGGCGATGAATACAAATGGGAACAYCTGGTAAAGGGTGGCATCATTGAGTTACTGGATGCTGAAGAAGAGGAAACAGTGATGATATCCATGACCCCCGAAGATCTTGAAAATTCTCGCCTACAGCAAAGTGGCGTTGATCCTCACGCGAACGACGGCGAGTTTGATCCAGCAGCTCGATTGAAGGCTGGCACTCACGCACACACATGGACACATTGCGAAA-----------------------------------------------------------------------------------------------------------------GGATGGCCAGACCCGCGAGCACGCTCTCCTCGCTTTCACCCTCGGTGTGCGGCAACTCATCGTTGCTGTCAACAAGATGGACACCACTAAGGTAAGAAACATTTCAC-TTATAGTAAGGCTCCTTGCACATTCTCATCATCTCA-TGTAGTGGAGCGAGGACCGTTTCAACGAAATCATCAAGGAGACGTCAACCTTCATCAAGAAGGTTGGTTACAACCCCAAGGCCGTTGCCTTTGTCCCTATTTCCGGGTGGCACGGCGACAACATGTTGGAGGAGTCCGCCAAGTAAGCAGTC--TCGTATTGTCATGATCGCCCCATCGCTCATGTTGCACTC-AGCATGCCTTGGTACAAGGGCTGGACCAAGGAGACCAAGGCTGGTGTCGTCAAGGGCAAGACCCTCCTCGATGCTATTGATGCCATTGAGCCCCCTGTCCGACCTTCCGACAAACCCCTCCGTCTCCCCCTCCAGGATGTTTACAAAATTGGTGGTATCGGAACTGTGCCCGTCGGTCGTGTTGAGACTGGTATCATCAAGGCCGGAATGGTCGTCACTTTCGCTCCCTCTAACGTCACCACTGAAGTCAAGTCCGTCGAAATGCATCACGAACAGCTCGAG------------------------------------------------------------------------------------------------------------------------------------------------------------------------------------------------------------------------------------------------------------------------------------------------------------------------------------------

>SFC20120919-05_Laccaria_parva_KOREA_MG519529_MG519573_MG551604_MG551640

CCTGA-TGTGACTGTTAGCTGGC-TTTTCGAAGCATGTGCTCGT-CCGTCATCTTTAATCTCT--CCA-CCTGTGCACATTTTGTAGTC-TT-GAATACCTCTCGAGGCAA-CTCGGATT----TTAG-GATTGCC---------------AAGTTGGCTTTCCTTTCATTTCC-AAGACTATGTTTT----T--A-TATACA-CCAAAGTATGTTTAAAGAATGTCATT-AATAGGAAC---TTGTTTCCTA--T-AAAATTATACAACTTTCAGCAACGGATCTCTTGGCTCTCGCATCGATGAAGAACGCAGCGAAATGCGATAAGTAATGTGAATTGCAGAATTCAGTGAATCATCGAATCTTTGAACGCACCTTGCGCTCCTTGGTATTCCGAGGAGCATGCCTGTTTGAGTGTCATTAAA-TTCTCAA-CCTTCCAGC--------T-TTTATAAGCTTGG--TTAGGCTTGGA-TGT-GGGAG--TTGCGGGCTTC-AT-----C---AATGAGGTCGGCTTTCCTTAAATGCATTAGCGGAACTTTTGTGGACCGTCTA-TTGGTGTGAT-AATTATCTACGCCGTGGAT-GTGAAACAG-CTTTAT---GAAGTT-CAGCTTCA-AACCGTCC---ATTCACTTGGACAA-----TTTTGACAA-TTTG-AAGCTCAAATTTAAAATCTGGCAGT-CTTTGGCTGT-CCGAGTTGTAATCTAGAGAAGTATTATCCGCGCTGGACCGTGTACAAGTCTCCTGGAATGGAGCGTCATAGAGGGTGAGAATCCCGTCTTTGACACGGACTGCCAGGGC-TTTGTGATGTGCTCTCAAAGAGTCGAGTTGTTTGGGAATGCAGCTCAAAATGGGTGGTAAATTCCATCTAAAGCTAAATATTGGCGAGAGACCGATAGCGAACAAGTACCGTGAGGGAAAGATGAAAAGAACTTTGGAAAGAGAGTTAAACAGTACGTGAAATTGCTGAAAGGGAAACGCTTGAAGTCAGTCACGTTGGCCAGGGATCAA-CCTTGC----TTTTTT---GCTTGGCTTACTTTCTGGTCGATGGG-TCAGCATCAATTTTGACTGGTAG-AAAAAGTTCAAGGGAATGTGGCAT-CTTCGGATGTGTTATAGCCTTTGTTCACATATATCAGTTGGGATTGAGGAACTCAGCACGCCGCAAGGCCGGG---TTTTTAACCACGTACGTGCTTAGGATGCTGGCATAATGGCTTTAATCGACCCGTCTTGAAACACGGACCAAGGAGTCTAACATGCCTGCGAGTGTTTGGGTGAAAAACCCGAGCGCGTAATGAAAGTG-AAAGTTGAGATCCCTGTCGTGGGGAGCATTGACGCCCGGATCTGACCTTTTGTGACGATTCTGCGGTAGAGCATGT--------------------------------------------------------------------------------------------------------------------------------------------------------------------------------------------------------------------------------------------------------------------------------------------------------------------------------------------------------------------------------------------------------------------------------------------CTCGCTCTTATGGCGTGCATATCAGTCGGGTCTTACTCCGCGCCCGTTATCGAGTTTTTGGAGGAGTGGGGATTGGAGTCATTGGAAGAGAACGCGCACTCATCAACACCTTGCACAAAAGTTTTCGTGAACGGTGTGTGGATGGGTGTGCATCGCGATCCTGCCAACTTGGTGAAGACGATAAAGAAATTGAGAAGAAAAGACGACATTAGTCCAGAAGTGTCAGTTGTGCGAGACATCAGGGAGAAAGAACTGAGGTTGTACACAGACGCTGGACGTGTTTGTCGACCACTCTTCATCGTCGAGAACCAACAGCTGGCACTTCAGAAGAAGCACGTCAAATGGCTCAGTAACGGCCTCAATGATGATGGCGATGAGTACAAATGGGAACATCTGGTAAAGGGTGGCATCATTGAGTTACTGGATGCTGAGGAAGAGGAAACAGTGATGATATCCATGACCCCTGAAGATCTTGAAAATTCTCGCTTACAACAAAGTGGTGTTGATCCTCACGCGAATGACGGCGAGTTTGATCCAGCGGCTCGTTTGAAGGCTGGCACTCACGCGCACACATGGACACATTGCGAAA-----------------------------------------------------------------------------------------------------------------GGATGGCCAGACCCGCGAGCACGCTCTCCTCGCTTTCACACTCGGTGTGCGGCAACTCATCGTTGCCGTCAACAAGATGGACACCACTAAGGTAAGAAACGTTACAC-TTATAGTAAGGTTGCTTGCACGTTCYCATCATCTCY-TGTAGTGGAGCGAGGACCGTTTCAACGAAATCATCAAGGAGACGTCAACCTTYATCAAGAAGGTTGGTTACAACCCCAAGGCCGTTGCCTTTGTCCCTATTTCCGGCTGGCACGGTGACAACATGTTGGAGGAGTCCGCTAAGTAAGCATTG--TCGTATCGTCATGATGGCCCCGTCGCTCATGTTGTACTCAAGCATGCCTTGGTACAAGGGCTGGACCAAGGAGACGAAGGCTGGTGTCGTCAAGGGCAAGACCCTCCTCGATGCTATTGATGCCATTGAACCCCCCGTCCGACCTTCCGACAAACCCCTCCGTCTCCCCCTCCAGGATGTCTACAAAATTGGCGGTATCGGAACTGTGCCTGTCGGTCGTGTTGAGACTGGTATCATCAAGGCCGGAATGGTCGTCACTTTCGCTCCCTCTAACGTCACCACTGAGGTCAAGTCCGTTGARATGCATCACGAACAGCTCGAG------------------------------------------------------------------------------------------------------------------------------------------------------------------------------------------------------------------------------------------------------------------------------------------------------------------------------------------

>ASIS21282_Laccaria_parva_KOREA_MG519526_MG519571_MG551601_MG551638

CCTGA-TGTGACTGTTAGCTGGC-TTTTCGAAGCATGTGCTCGT-CCGTCATCTTTAMTCTCT--CCA-CCTGTGCACATTTTGTAGTC-TT-GAATACCTCTCGAGGCAA-CTCGGATT----TTAG-GATTGCC---------------AAGTTGGCTTTCCTTTCATTTCC-AAGACTATGTTTT----T--A-TATACA-CCAAAGTATGTTTAAAGAATGTCATT-AATAGGAAC---TTGTTTCCTA--T-AAAATTATACAACTTTCAGCAACGGATCTCTTGGCTCTCGCATCGATGAAGAACGCAGCGAAATGCGATAAGTAATGTGAATTGCAGAATTCAGTGAATCATCGAATCTTTGAACGCACCTTGCGCTCCTTGGTATTCCGAGGAGCATGCCTGTTTGAGTGTCATTAAA-TTCTCAA-CCTTCCAGC--------T-TTTATAAGCTTGG--TTAGGCTTGGA-TGT-GGGAG--TTGCGGGCTTC-AT-----C---AATGAGGTCGGCTTTCCTYAAATGCATTAGCGGAACTTTTGTGGACCGTCTA-TTGGTGTGAT-AATTATCTACGCCGTGGAT-GTGAAACAG-CTTTAT---GAAGTT-CAGCTTCA-AACCGTCC---ATTCACTTGGACAA-----TTTTGACAA-TTTG-AAGCTCAAATTTAAAATCTGGCAGT-CTTTGGCTGT-CCGAGTTGTAATCTAGAGAAGTATTATCCGCGCTGGACCGTGTACAAGTCTCCTGGAATGGAGCGTCATAGAGGGTGAGAATCCCGTCTCTGACACGGACTGCCAGGGC-TTTGTGATGTGCTCTCAAAGAGTCGAGTTGTTTGGGAATGCAGCTCAAAATGGGTGGTAAATTCCATCTAAAGCTAAATATTGGCGAGAGACCGATAGCGAACAAGTACCGTGAGGGAAAGATGAAAAGAACTTTGGAAAGAGAGTTAAACAGTACGTGAAATTGCTGAAAGGGAAACGCTTGAAGTCAGTCACGTTGGCCAGGGATCAA-CCTTGC----TTTTTT---GCTTGGCTTACTTTCTGGTCGATGGG-TCAGCATCAATTTTGACTGGTAG-AAAAAGTTCAAGGGAATGTGGCAT-CTTCGGATGTGTTATAGCCTTTGTTCACATATATCAGTTGGGATTGAGGAACTCAGCACGCCGCAAGGCCGGG---TTTTTAACCACGTACGTGCTTAGGATGCTGGCATAATGGCTTTAATCGACCCGTCTTGAAACACGGACCAAGGAGTCTAACATGCCTGCGAGTGTTTGGGTGAAAAACCCGAGCGCGTAATGAAAGTG-AAAGTTGAGATCCCTGTCGTGGGGAGCATTGACGCCCGGATCTGACCTTTTGTGACGATTCTGCGGTAGAGCATGT--------------------------------------------------------------------------------------------------------------------------------------------------------------------------------------------------------------------------------------------------------------------------------------------------------------------------------------------------------------------------------------------------------------------------------------------CTCGCTCTTATGGCGTGCATATCAGTCGGGTCTTACTCCGCGCCCGTTATCGAGTTTTTGGAGGAGTGGGGATTGGAGTCATTGGAAGAAAACGCGCACTCATCAACACCTTGCACAAAAGTTTTCGTGAACGGTGTGTGGATGGGTGTGCATCGCGATCCTGCCAACTTGGTGAAGACGATAAAGAAATTGAGGAGAAAAGACGACATTAGTCCAGAAGTGTCAGTTGTGCGAGACATCAGGGAGAAGGAATTGAGGTTGTACACAGACGCTGGACGTGTTTGTCGACCACTCTTCATCGTCGAGAACCAACAGCTGGCACTTCAGAAGAAGCACGTCAAATGGCTCAGYAACGGCCTCAATGATGATGGCGATGAGTACAAATGGGAACATCTGGTAAAGGGTGGCATCATTGAGTTACTGGATGCTGAGGAAGAGGAAACAGTGATGATATCCATGACCCCTGAAGATCTTGAAAATTCTCGCTTACAACAAAGTGGTGTTGATCCTCACGCGAATGACGGCGAGTTTGACCCAGCAGCTCGTTTGAAGGCTGGCACTCACGCGCACACATGGACACATTGCGAAA-----------------------------------------------------------------------------------------------------------------GGATGGCCAGACCCGCGAGCACGCTCTCCTCGCTTTCACACTCGGTGTGCGGCAACTCATCGTTGCCGTCAACAAGATGGACACCACCAAGGTAAGAAACGTTACAC-TTATAGTAAGGTTGCTTGCACGTTCTCATCATCTCC-TGTAGTGGAGCGAGGACCGTTTCAACGAAATCATCAAGGAGACGTCAACCTTCATCAAGAAGGTTGGTTACAACCCCAAGGCCGTTGCCTTTGTCCCTATTTCCGGCTGGCACGGTGACAACATGTTGGAGGAGTCCGCTAAGTAAGCGTTG--TCGTATCGTCATGATGGCCCCGTCGCTCATGTTGTACTCAAGCATGCCTTGGTACAAGGGCTGGACCAAGGAGACGAAGGCTGGTGTCGTCAAGGGCAAGACCCTCCTCGATGCTATTGATGCCATTGAACCCCCCGTCCGACCTTCCGACAAACCCCTCCGTCTCCCCCTCCAGGATGTCTACAAAATTGGCGGTATCGGAACTGTGCCTGTCGGTCGTGTTGAGACTGGTATCATCAAGGCCGGAATGGTCGTCACTTTCGCTCCCTCTAACGTCACCACTGAGGTCAAGTCCGTTGAAATGCATCACGAACAGCTCGAG------------------------------------------------------------------------------------------------------------------------------------------------------------------------------------------------------------------------------------------------------------------------------------------------------------------------------------------

>SFC20150902-17_Laccaria_torosa_KOREA_MG519561_MG519598_MG551631_MG551664

CCTGA-TGTGGCTGTTAGCTGGC-TTTTTGAAGCATGTGCTCGT-CCATCATCTTT-ATCTCTCTCCA-CCTGTGCACATTTTGTAGTC-TT-GGATACCTCTCGAGGCAA-CTCGGATT----TTAG-GATCGCTGTGC---TGT---ACAAGTCAGCTTTCCTTTAATTTCT-AAGACTATGTTTT----T--A-TATACA-CCAAAGTATGTTTAAAGAATGTCATC-AATAGGAACTTGTTGTTTCCTA--TAAAAATTATACAACTTTCAGCAACGGATCTCTTGGCTCTCGCATCGATGAAGAACGCAGCGAAATGCGATAAGTAATGTGAATTGCAGAATTCAGTGAATCATCGAATCTTTGAACGCACCTTGCGCTCCTTGGTATTCCGAGGAGCATGCCTGTTTGAGTGTCATTAAA-TTCTCAA-CCTTCCAGC--------T-TTTATTAGCTTGG--TTAGGCTTGGA-TGTGGGGGG--TTGTGGGCTTC-AT-----T---AATGAGGTCGGCTCTCCTTAAATGTATTAGCAGAACTTTTGTGGACCGTCTA-TTGGTGTGAT-AATTATCTATGCCGTGGAT-GTGAAGCAGCTTTTAC---AAAGTT-CAGCTTCT-AACTGTCC---ATTGACTTGGATAA---ATTTTTGACAA-TTTG-AAGCTCAAATTTAAAATCTGGCAGT-CTTTGGCTGT-CCGAATTGTAATCTAGAGAAGTATTATCCGCGCTGGACCGTGTACAAGTCTCCTGGAATGGAGCGTCATAGAGGGTGAGAATCCCGTCTTTGACACGGACTGCCAGGGC-TTTGTGATATGCTCTCAAAGAGTCGAGTTGTTTGGGAATGCAGCTCAAAATGGGTGGTAAATTCCATCTAAAGCTAAATATTGGCGAGAGACCGATAGCGAACAAGTACCGTGAGGGAAAGATGAAAAGAACTTTGGAAAGAGAGTTAAACAGTACGTGAAATTGCTGAAAGGGAAACGCTTGAAGTCAGTCGCGTTGGCCAGGGATCAA-CCTTGC-TTTTTTTTT---GCTTGGCTTACTTTCTGGTCGATGGG-TCAGCATCAATTTTGACTGGTGG-AAAAAGTTCAAGGGAATGTGGCAT-CTTTGGATGTGTTATAGCCTTTGTTCACATACATTAGTTGGGATTGAGGAACTCAGCACGCCGAAAGGCCGGG---TTTTTAACCACGTACGTGCTTAGGATGCTGGCATAATGGCTTTAATCGACCCGTCTTGAAACACGGACCAAGGAGTCTAACATGCGTGCGAGTGTTTGGGTGAAAAACCCGTGCGCGTAATGAAAGTG-AAAGTTGAGATCCCTGTCGTGGGGAGCATTGACGCCCGGATCTGACCTTTTGTGACGATTCTGCGGTAAAGCATGC--------------------------------------------------------------------------------------------------------------------------------------------------------------------------------------------------------------------------------------------------------------------------------------------------------------------------------------------------------------------------------------------------------------------------------------------CTCGCCCTTATGGCGTGCATATCAGTTGGGTCTTACTCTGCGCCCGTTATCGAGTTTTTGGAGGAGTGGGGATTGGAGTCATTGGAAGAGAACGCACACTCATCAACACCTTGCACAAAAGTTTTCGTGAACGGTGTCTGGATGGGTGTGCATCGCGATCCTGCCAATTTGGTGAAGWCGATAAAGWAATTGAGAAGGAAAGACGACATCAGTCCAGAAGTCTCAGTTSTGCGAGACATCAGGGAGAAAGAATTGAGGYTGTACACCGATGCTGGWCGTGTTTGTCGACCACTCTTCATCGTCGAGAACCAACAGCTGGCACTTCAGAAGAAGCACGTCAAATGGCTCAGTAACGGCCTCAACGATGATGGCGACGAGTACAAATGGGAACATCTGGTAAAGGGTGGTATCATTGAGTTACTGGATGCTGAGGAGGAGGAAACAGYGATGATATCCATGACCCCCGAAGATCTTGAAAATTCTCGCCTACAGCAAAGTGGTGYTGATCCYCACGCAAACGACGGCGAGTTYGATCCAGCAGCTCGATTGAAAGCCGGCACTCACGCACACACATGGACACATTGCGAAA-----------------------------------------------------------------------------------------------------------------GGATGGCCAGACCCGCGAGCACGCTCTCCTCGCTTTCACCCTCGGTGTGCGGCAACTTATCGTTGCCGTCAACAAGATGGACACCACTAAGGTAAGAAACGTTCCAC-TTATAGTTAGGCTTCTTGCACATTCTCATCATCTCA-TTTAGTGGAGCGAGGACCGTTTCAACGAAATCATCAAGGAGACGTCAACCTTCATCAAGAAGGTTGGTTACAACCCCAAGGCCGTTGCCTTTGTCCCTATTTCAGGCTGGCACGGTGACAACATGTTGGAGGAGTCCTCTAAGTAAGCATTC--CCGTATCGTCATGATCGCCCCGTCGCTCATGTTGCACAC-AGCATGCCTTGGTACAAGGGCTGGACCAAGGAGACCAAGGCTGGTGTCGTCAAGGGCAAGACCCTCCTCGATGCTATTGATGCCATTGAGCCCCCCGTCCGACCTTCCGACAAACCCCTCCGTCTCCCCCTTCAGGATGTCTACAAAATTGGCGGTATCGGAACTGTGCCCGTCGGTCGTGTTGAGACTGGTATCATCAAGGCCGGAATGGTCGTCACTTTTGCTCCCTCCAACGTCACCACTGAAGTCAAGTCCGTCGAAATGCATCACGAACAGCTCGAG------------------------------------------------------------------------------------------------------------------------------------------------------------------------------------------------------------------------------------------------------------------------------------------------------------------------------------------

>SFC20121010-51_Laccaria_versiforma_KOREA_MG519555_MG519593_MG551626_MG551659

CCTGA-TGTGGCTGTTAGCTGGC-TTTTCGAAGCATGTGCTCGT-CCGTCATCTTT-ATCTCT--CCA-CCTGTGCACATTTTGTAGTC-TT-GRATATTGCT--------------------------------------------------------------------TCC-AAGACTATGTTTT----TATA-TAYACA-CCAAAGTATGTTTAAAGAATGTCATC-AATAGAAAC---TTGTTTTCTA--TAAAAAATATACAACTTTCAGCAACGGATCTCTTGGCTCTCGCATCGATGAAGAACGCAGCGAAATGCGATAAGTAATGTGAATTGCAGAATTCAGTGAATCATCGAATCTTTGAACGCACCTTGCGCTCCTTGGTATTCCGAGGAGCATGCCTGTTTGAGTGTCATTAAA-TTCTCAA-CCTTCCAAC--------T-TTTATTAGCTTGG--TTAGGCTTGGA-TGT-GGGGG--TTGCAGGCTTCTAT-----T---AATGAGGTCAGCTCTCCTTAAATGCATTAGCGGAACTTTTGTGGACTGTCTA-TTGGTGTGAT-AATTATCTATGCCGTGGAT-GTGAAGCAG-CTTTAT---GAAGTT-CAGCTTCG-AACCGTCC---ATTGACTTGGACAA----TTTTTGACAA-TTTG-AAGCTCAAATTTAAAATCTGGCAGT-CTTTGGCTGT-CCGAGTTGTAATCTAGAGAAGTATTATCCGCGCTGGACCGTGTACAAGTCTCCTGGAATGGAGCATCATAGAGGGTGAGAATCCCGTCTTTGACATGGACTGCCAGGGC-TTTGTGATATGCTCTCGAAGAGTCGAGTTGTTTGGGAATGCAGCTCAAAATGGGTGGTAAATTCCATCTAAAGCTAAATATTGGCGAGAGACCGATAGCGAACAAGTACCGTGAGGGAAAGATGAAAAGAACTTTGGAAAGAGAGTTAAACAGTACGTGAAATTGCTGAAAGGGAAACGCTTGAAGTCAGTCGCGTTGGCCAGGGATCAA-CCTTGC---TTTTTTT---GCTTGGCTTACTTTCTGGTCAATGGG-TCAGCATCAATTTTGACTGGTGG-AAAAAGTTCAAGGGAATGTGGCAT-CTTTGGATGTGTTATAGCCTTTGTTCATATACATTGGTTGGGATTGAGGAACTCAGCACGCCGAAAGGCCGGG---TTTTTAACCACGTACGTGCTTAGGATGCTGGCATAATGGCTTTAATCGACCCGTCTTGAAACACGGACCAAGGAGTCTAACATGCCTGCGAGTGTTTGGGTGAAAAACCCGAGCGCGTAATGAAAGTG-AAAGTTGAGATCCCTGTCGTGGGGAGCATTGACGCCCGGATCTGACCTTTTGTGACGATTCTGCGGTAGAGCATGT--------------------------------------------------------------------------------------------------------------------------------------------------------------------------------------------------------------------------------------------------------------------------------------------------------------------------------------------------------------------------------------------------------------------------------------------CTCGCCCTTATGGCGTGCATATCAGTCGGGTCTTACTCCGCGCCCGTTATCGAGTTTTTGGAGGAGTGGGGGTTGGAGTCATTGGAAGAGAACGCGCACTCATCAACACCTTGCACAAAAGTTTTCGTGAACGGTGTCTGGATGGGTGTACATCGCGATCCTGCCAATTTGGTGAAAACGATAAAGAAATTGAGAAGGAAAGATGACATCAGTCCAGAGGTGTCAGTTGTGCGAGACATCAGGGAGAAGGAACTGAGGTTGTACACCGATGCTGGACGTGTTTGTCGACCACTCTTCATCGTCGAGAACCAACAGCTGGCACTTCAGAAGAAGCACGTCAAATGGCTCAGTAACGGCCTCAACGACGATGGTGACGAGTACAAATGGGAACATCTGGTAAAGGGTGGCATTATTGAGTTGCTGGATGCTGAGGAGGAGGAAACGGTGATGATATCCATGACCCCCGAAGACCTTGAAAATTCTCGCCTACAGCAAAGTGGTGTTGATCCTCACGCGAACGACGGCGAGTTTGATCCGGCGGCTCGATTGAAGGCGGGCACTCACGCACACACGTGGACACATTGCGAAA-----------------------------------------------------------------------------------------------------------------GGATGGCCAGACCCGCGAGCACGCTCTCCTCGCTTTCACCCTCGGTGTACGGCAACTCATCGTTGCCGTCAAYAAGATGGACACYACTAAGGTAAGGGATGTTTCGC-TTATAGTTAGGCTTCCTGCACATTCTCATCATCTCA-TGTAGTGGAGCGAGGACCGTTTCAACGAAATCATCAAGGAGACRTCAACCTTCATCAAGAAGGTTGGTTACAACCCCAAGGCCGTTGCCTTTGTGCCTATTTCCGGGTGGCACGGTGACAACATGTTGGAGGAGTCCTCTAAGTAAGCATTC--TCGTATCGTCATGATCGCCCCGTCGCTCATGTTGCACTC-AGCATGCCTTGGTACAAGGGCTGGACCAAGGAGACCWAGGCTGGTGTCGTCAAGGGCAAGACCCTCCTCGATGCTATTGATGCTATTGAGCCCCCCGTCCGACCTTCCGACAAACCCCTCCGTCTCCCCCTCCAGGATGTCTACAAAATTGGCGGTATCGGAACTGTGCCCGTCGGTCGTGTTGAGACTGGTATCATCAAAGCCGGAATGGTCGTCAATTTCGCTCCCTCCAACGTCACCACTGAAGTCAAGTCTGTCGAAATGCATCACG-----------------------------------------------------------------------------------------------------------------------------------------------------------------------------------------------------------------------------------------------------------------------------------------------------------------------------------------------------

>SFC20120926-01_Laccaria_versiforma_KOREA_MG519556_MG519594_MG551627_MG551660

CCTGA-TGTGGCTGTTAGCTGGC-TTTTCGAAGCATGTGCTCGT-CCGTCATCTTT-ATCTCT--CCA-CCTGTGCACATTTTGTAGTC-TT-GGATATTGCT--------------------------------------------------------------------TCC-AAGACTATGTTTT----T--A-TATACA-CCAAAGTATGTTTAAAGAATGTCATC-AATAGAAAC---TTGTTTTCTA--TGAAAATTATACAACTTTCAGCAACGGATCTCTTGGCTCTCGCATCGATGAAGAACGCAGCGAAATGCGATAAGTAATGTGAATTGCAGAATTCAGTGAATCATCGAATCTTTGAACGCACCTTGCGCTCCTTGGTATTCCGAGGAGCATGCCTGTTTGAGTGTCATTAAA-TTCTCAA-CCTTCCAAC--------T-TTTATTAGCTTGG--TTAGGCTTGGA-AGT-GGGGG--TTGCGGGCTTCTAT-----T---AATGAGGTCAGCTCTCCTTAAATGCATTAGTGGAACTTTTGTGGACCCTCTA-TTGGTGTGAT-AATTATCTACGCCGTGGAT-GTGAAGCAG-CTTTAT---GAAGTT-CAGCTTCT-AACTGTCC---ATTGACTTGGACAA----TTTTTGACAA-TTTG-AAGCTCAAATTTAAAATCTGGCAGT-CTTTGGCTGT-CCGAGTTGTAATCTAGAGAAGTATTATCCGCGCTGGACCGTGTACAAGTCTCCTGGAATGGAGCGTCATAGAGGGTGAGAATCCCGTCTTTGACACGGACTGCCAGGGC-TTTGTGATATGCTCTCAAAGAGTCGAGTTGTTTGGGAATGCAGCTCAAAATGGGTGGTAAATTCCATCTAAAGCTAAATATTGGCGAGAGACCGATAGCGAACAAGTACCGTGAGGGAAAGATGAAAAGAACTTTGGAAAGAGAGTTAAACAGTACGTGAAATTGCTGAAAGGGAAACGCTTGAAGTCAGTCGTGTTGGCCAGGGATCAA-CCTTGC----TCTTTT---GCTTGGCTTATTTTCTGGTCAATGGG-TCAGCATCAATTTTGACTGGTGG-AAAAAGTTCAAGGGAATGTGGCAT-CTTCGGATGTGTTATAGCCTTTGTTCATATGCATTGGTTGGGATTGAGGAACTCAGCACGCCGAAAGGCCGGG---TTTTTAACCACGTACGTGCTTAGGATGCTGGCATAATGGCTTTAATCGACCCGTCTTGAAACACGGACCAAGGAGTCTAACATGCCTGCGAGTGTTTGGGTGAAAAACCCGAGCGCGTAATGAAAGTG-AAAGTTGAGATCCCTGTCGTAGGGAGCATTGACGCCCGGATCTGACCTTTTGTGACGATTCTGCGGTAGAGCATGT--------------------------------------------------------------------------------------------------------------------------------------------------------------------------------------------------------------------------------------------------------------------------------------------------------------------------------------------------------------------------------------------------------------------------------------------CTCGCCCTTATGGCGTGCATATCAGTCGGGTCTTACTCCGCGCCCGTTATCGAGTTTTTGGAGGAGTGGGGGTTGGAGTCATTGGAAGAGAACGCGCACTCATCAACACCTTGCACAAAAGTTTTCGTGAACGGTGTCTGGATGGGTGTACATCGCGATCCTGCCAATTTGGTGAAAACGATAAAGAAATTGAGAAGGAAAGATGACATCAGTCCAGAGGTGTCAGTTGTGCGAGACATCAGGGAGAAGGAACTGAGGTTGTACACCGATGCTGGACGTGTTTGTCGACCACTCTTCATCGTCGAGAACCAACAGCTGGCACTTCAGAAGAAGCACGTCAAATGGCTCAGTAACGGCCTCAACGACGATGGTGACGAGTACAAATGGGAACATCTGGTAAAGGGTGGCATTATTGAGTTGCTGGATGCTGAGGAGGAGGAAACGGTGATGATATCCATGACCCCCGAAGACCTTGAAAATTCTCGCCTACAGCAAAGTGGTGTTGATCCTCACGCGAACGACGGCGAGTTTGATCCGGCGGCTCGATTGAAGGCGGGCACTCACGCACACACGTGGACACATTGCGAAA-----------------------------------------------------------------------------------------------------------------GGATGGCCAGACCCGCGAGCACGCTCTCCTCGCTTTCACCCTCGGTGTACGGCAACTCATCGTTGCCGTCAACAAGATGGACACCACTAAGGTAAGGGATGTTTCGC-TTATAGTTAGGCTTCCTGCACATTCTCATCATCTCA-TGTAGTGGAGCGAGGACCGTTTCAACGAAATCATCAAGGAGACRTCAACCTTCATCAAGAAGGTTGGTTACAACCCCAAGGCCGTTGCCTTTGTGCCTATTTCCGGGTGGCACGGTGACAACATGTTGGAGGAGTCCTCTAAGTAAGCATTC--TCGTATCGTCATGATCGCCCCGTCGCTCATKTTGCACTC-AGCATGCCTTGGTACAAGGGCTGGACCAAGGAGACCAAGGCTGGTGTCGTCAAGGGCAAGACCCTCCTCGATGCTATTGATGCTATTGAGCCCCCCGTCCGACCTTCCGACAAACCCCTCCGTCTCCCCCTCCAGGATGTCTACAAAATTGGCGGTATCGGAACTGTGCCCGTCGGTCGTGTTGAGACTGGTATCATCAAAGCCGGAATGGTCGTCAATTTCGCTCCCTCCAACGTCACCACTGAAGTCAAGTCTGTCGAAATGCATCACGAACAGCTTGAG------------------------------------------------------------------------------------------------------------------------------------------------------------------------------------------------------------------------------------------------------------------------------------------------------------------------------------------

>F1102432_Laccaria_amethystea_COSTA_RICA_KU685638_KU685781_KU686062_

-CTGA-TGTGGCTGTTAGCTGGC-TTTTCGAAGCATGTGCTCGTCCCGTCATCTTT-ATCTCT--CCA-CCTGTGAACATTTTGTAGTC-TT-GGATACCTCTCGAGTCAA-CTCGGATT--T-TAAG-GATTGCCGTGC---TTGT--ACAAGTTGGCTTTCCTTTCACTTCC-AAGACTATGTTTT-------A-YACACA-CCCAAGTATGTTTAAAGAATGTCATC-AATAGGAAC---TTGTTTCCTA--T-AAAATTATACAACTTTCAGCAACGGATCTCTTGGCTCTCGCATCGATGAAGAACGCAGCGAAATGCGATAAGTAATGTGAATTGCAGAATTCAGTGAATCATCGAATCTTTGAACGCACCTTGCGCTCCTTGGTATTCCGAGGAGCATGCCTGTTTGAGTGTCATTAAA-TTCTCAA-CCTTCCAGC--------T-TTTATTAGCTTGG--TTAGGATTGGA-TGT-GGGAG--TTGCAGGCTTC-AT-----T---AAYGAGGTCAGCTCTCCTTAAATGCATTAGCGGAACTTTTGTGGACCGTCTA-TTGGTGTGAT-AATTATCTACGCCGTGGAT-GTGAAGCAG-ATCTAT---GAAGTT-CAGCTTCT-AACCGTCC---ATTGACTTGGACAA-----TTTTGACAA-TTTGAAAGCTCAAATTTAAAATCTGGCGGT-CTTTGGCTGT-CCGAGTTGTAATCTAGAGAAGTATTATCCGCGCTGGACCGTGTACAAGTCTCCTGGAATGGGGCGTCATAGAGGGTGAGAATCCCGTCTTTGACACGGACTGCCAGGGC-TTTGTGATGTGCTCTCAAAGAGTCGAGTTGTTTGGGAATGCAGCTCAAAATGGGTGGTAAATTCCATCTAAAGCTAAATATTGGCGAGAGACCGATAGCGAACAAGTACCGTGAGGGAAAGATGAAAAGAACTTTGGAAAGAGAGTTAAACAGTACGTGAAATTGCTGAAAGGGAAACGCTTGAAGTCAGTCGCATTGGCCAGGGATCAA-CCTTGC----TTTTTT---GCTTGGCTTACTTTCTGGTCAATGGG-TCAGCATCAATTTTGACTGGTGG-AAAAAGTTCAAGGGAATGTGGCAT-CTTTGGATGTGTTATAGCCTTTGTTCACATACATTGGTCGGGATTGAGGAACTCAGCACGCTGCAAGGCCGGG---TTTTTAACCACGTACGTGCTTAGGATGCTGGCATAATGGCTTTAATCGACCCGTCTTGAAACACGGACCAAGGAGTCTAACATGCCTGCGAGTGTTTGGGTGAAAAACCCGAGCGCGTAATGAAAGTG-AAAGTTGAGATCCCTGTCATGGGGAGCATTGACGCCCGGATCTGACCTTTTGTGACGATTCTGCGGTAGAGCATGTTTTCCGCATGCTTTTCCGAAAGTTGACMAaGGATGTTTATCGTTATTTACAAAAGGTTGCTGACCTTCGTTCAAGTGCC-----ATGARATGTGCTGA-CCCCTCT---TCTTCAGTGCGTCGAGACCCACAAGGAGTTCAACCTTGCCCTTGCAGTTAAGCATCAAACCATCACAAACGGCCTCAAATACTCTCTGGCGACCGGWAACTGGGGAGATCARAAAAAGTCAATGTCTTCCAAGGCAGGAGTATCCCAGGTCTTGAACAGATACACATATGCATCCACTCTGTCACATCTTCGTCGGTGTAACACTCCTCTAGGGCGCGAAGGCAAGATCGCCAAACCTC-----------------------------------------------------------------------------------------------------------------------------------------------------------------------------------------------------------------------------------------------------------------------------------------------------------------------------------------------------------------------------------------------------------------------------------------------------------------------------------------------------------------------------------------------------------------------------------------------------------------------------------------------------------------------------------------------------------------------------------------------------------------------------------------------------------------------------------------------------------------------------------------------------------------------------------------------------------------------------------------------------------------------------------------------------------------------------------------------------------------------------------------------------------------------------------------------------------------------------------------------------------------------------------------------------------------------------------------------------------------------------------------------------------------------------------------------------------------------------------------------------------------------------------------------------------------------------------------------------------------------------------------------------------------------------------------------------------------------------------------------------------------------------------------------------

>F1102433_Laccaria_gomezii_COSTA_RICA__F1102433_RPB2_

-------------------------------------------------------------------------------------------------------------------------------------------------------------------------------------------------------------------------------------------------------------------------------------------------------------------------------------------------------------------------------------------------------------------------------------------------------------------------------------------------------------------------------------------------------------------------------------------------------------------------------------------------------------------------------------------------------AAGCTCAAATTTAAAATCTGGCAGT-CTTTGGCTGT-CCGAGTTGTAATCTAGAGAAGTATTATCCGCGCTGGACCGTGTACAAGTCTCCTGGAATGGAGCGTCATAGAGGGTGAGAATCCCGTCTTTGACACGGACTGCCAGGGC-TTTGTGATATGCTCTCAAAGAGTCGAGTTGTTTGGGAATGCAGCTCAAAATGGGTGGTAAATTCCATCTAAAGCTAAATATTGGCGAGAGACCGATAGCGAACAAGTACCGTGAGGGAAAGATGAAAAGAACTTTGGAAAGAGAGTTAAACAGTACGTGAAATTGCTGAAAGGGAAACGCTTGAAGTCAGTCGCGTTGGCCAGGGATCAA-CCTTGC----TTTTTT---GCTTGGCTTACTTTCTGGTTGATGGG-TCAGCATCAATTTTGACTGGTGG-AAAAAGTTCAAGGGAATGTGGCAT-CTTCGGATGTGTTATAGCCTTTGTTCACATACATTGGTTGGGATTGAGGAACTCAGCACGCCGCAAGGCCGGG---TTTTTAACCACGTACGTGCTTAGGATGCTGGCATAATGGCTTTAATCGACCCGTCTTGAAACACGGACCAAGGAGTCTAACATGCCTGCGAGTATTTGGGTGAAAAACCCAAGTGCGTAATGAAAGTG-AAAGTTGAGATCCCTGTCGTGGGGAGCATTGACGCCCGGATCTGACCTTTTGTGACGATTCTGCGGTAGAGCATGTTTTCCGCATGCTGTTCCGAAAGTTGACCAAGGACGTTTATCGTTATCTACAAAAGGTTGCTGACTTTCGCTCAAGTGCC-----ATGAGACATGTTGA-CCTCTCT---TCTTCAGTGCGTCGAGACCCACAAGGAGTTCAACCTTGCCCTTGCAGTTAAGCATCAAACCATTACAAACGGCCTCAAATACTCTCTGGCGACAGGTAACTGGGGGGATCAGAAAAAGTCAATGTCTTCCAAGGCAGGAGTATCTCAGGTCTTGAACAGATACACATATGCATCCACTCTGTCACATCTCCGTCGGTGTAACACTCCTCTAGGACGCGAAGGCAAGATCGCCAAGCCTCGTCAGTTGCACAACACCCACTGGGG----------------------------------------------------------------------------------------------------------------------------------------------------------------------------------------------------------------------------------------------------------------------------------------------------------------------------------------------------------------------------------------------------------------------------------------------------------------------------------------------------------------------------------------------------------------------------------------------------------------------------------------------------------------------------------------------------------------------------------------------------------------------------------------------------------------------------------------------------------------------------------------------------------------------------------------------------------------------------------------------------------------------------------------------------------------------------------------------------------------------------------------------------------------------------------------------------------------------------------------------------------------------------------------------------------------------------------------------------------------------------------------------------------------------------------------------------------------------------------------------------------------------------------------------------------------------------------------------------------------------------------------------------------------------------------------------------------------------------------------------------------------------------

>F1102471_Laccaria_major_COSTA_RICA_F1102471___

-------GTGACTGTTAGCTGGC-TTTTCGAAGCATGTGCTCGT-CCATCGTCTTT-ATCTCT--CCA-CCTGTGCACATTTTGTAGTC-TT-GGATACCTCTCGAGGCAA-CTCGGATA----TTAG-GATCGCC--------------CAAGTTGGCTTTCCTTTCATTTCC-AAGACTATGTTTT-------A-TATACA-CCAAAGTATGTTTTGAGAATGTCATC-AATAGGAAC---TTGTTTTCTA--T-AAAATTATACAACTTTCAGCAACGGATCTCTTGGCTCTCGCATCGATGAAGAACGCAGCGAAATGCGATAAGTAATGTGAATTGCAGAATTCAGTGAATCATCGAATCTTTGAACGCACCTTGCGCTCCTTGGTATTCCGAGGAGCATGCCTGTTTGAGTGTCATCAAA-TTCTCAACCTTTCCAGC--------T-TTTATTAGCTTGG--TTAGGCTTGGA-TGT-GGGGG--CTGCAGGCTTC-AT-----T---AATGAGGTTGGCTCTCCTTAAATGCATTAGCGGAACTTTTGTAGACCGTCTA-TTGGTGTGAT-AAATATCTACGCCGTGGAT-GTAAT--------------GAAGTT-CAGCTTCT-AACCGTCC---------TTGGACAA-----TTTTGACAA-TTTGA-------------------------------------------------------------------------------------------------------------------------------------------------------------------------------------------------------------------------------------------------------------------------------------------------------------------------------------------------------------------------------------------------------------------------------------------------------------------------------------------------------------------------------------------------------------------------------------------------------------------------------------------------------------------------------------------------------------------------------------------------------------------------------------------------------------------------------------------------------------------------------------------------------------------------------------------------------------------------------------------------------------------------------------------------------------------------------------------------------------------------------------------------------------------------------------------------------------------------------------------------------------------------------------------------------------------------------------------------------------------------------------------------------------------------------------------------------------------------------------------------------------------------------------------------------------------------------------------------------------------------------------------------------------------------------------------------------------------------------------------------------------------------------------------------------------------------------------------------------------------------------------------------------------------------------------------------------------------------------------------------------------------------------------------------------------------------------------------------------------------------------------------------------------------------------------------------------------------------------------------------------------------------------------------------------------------------------------------------------------------------------------------------------------------------------------------------------------------------------------------------------------------------------------------------------------------------------------------------------------------------------------------------------------------------------------------------------------------------------------------------------------------------------------------------------------------------------------------------------------------------------------------------------------------------------

>F1104722_Laccaria_calospora_COSTA_RICA_KU685639_KU685782__

-CTGA-TGTGACTGTTAGCTGGC-TTTTCGAAGCATGTGCTCGT-CCATCATCTTT-ATCTCT--CCA-CCTGTGCACATTTTGTAGTC-TT-GGATACCTCTCGAGGAAA-CTCGGATT----TTAG-GATTGTCGTGC---TGT---ACAAGTCGACTTTTCTTTCATTTCC-AAGACTATGTTTT----T--A-TATACA-CCAAAGTATG-TTATAGAATGTCATC-AATGGGAAC---TTGTTTCCTA--T-AAAATTATACAACTTTCAGCAACGGATCTCTTGGCTCTCGCATCGATGAAGAACGCAGCGAAATGCGATAAGTAATGTGAATTGCAGAATTCAGTGAATCATCGAATCTTTGAACGCACCTTGCGCTCCTTGGTATTCCGAGGAGCATGCCTGTTTGAGTGTCATTAAA-TTCTCAA-CCTTCCAAC--------T-TTTATTAGCTTGG--TTAGGCTTGGA-TGT-GGGGG--TTGCAGGCTTC-TT-----C---ACTGAGGTCAGCTCTCCTTAAATGCATTAGCGGAACTTTTGTGGACCGTCTA-TTGGTGTGAT-AATTATCTACGCCGTGGAT-GTGAAGCAG-CTTTAT---GAAGTT-CTGCTTCT-AACTGTCC---ATTGACTTGGACAA-----TTTTGACAA-TTTGAAAGCTCAAATTTAAAATCTGGCAGT-CTTTGGCTGT-CCGAGTTGTAATCTAGAGAAGTATTATCCGCGCTGGACCGTGTACAAGTCTCCTGGAATGGAGCGTCATAGAGGGTGAGAATCCCGTCTTTGACACGGACTGCCAGGGC-TTTGTGATATGCTCTCAAAGAGTCGAGTTGTTTGGGAATGCAGCTCAAAATGGGTGGTAAATTCCATCTAAAGCTAAATATTGGCGAGAGACCGATAGCGAACAAGTACCGTGAGGGAAAGATGAAAAGAACTTTGGAAAGAGAGTTAAACAGTACGTGAAATTGCTGAAAGGGAAACGCTTGAAGTCAGTCGCGTTGGCCAGGGATCAA-CCTTGC----TTTTTT---GCTTGGCTTACTTTCTGGTTGATGGG-TCAGCATCAATTTTGACTGGTGG-AAAAAGTTCAAGGGAATGTGGCAT-CTTCGGATGTGTTATAGCCTTTGTTCACATACATTGGTTGGGATTGAGGAACTCAGCACGCCGCAAGGCCGGG---TTTTTAACCACGTACGTGCTTAGGATGCTGGCATAATGGCTTTAATCGACCCGTCTTGAAACACGGACCAAGGAGTCTAACATGCCTGCGAGTATTTGGGTGAAAAACCCAAGTGCGTAATGAAAGTG-AAAGTTGAGATCCCTGTCGTGGGGAGCATTGACGCCCGGATCTGACCTTTTGTGACGATTCTGCGGTAGAGCATGT-----------------------------------------------------------------------------------------------------------------------------------------------------------------------------------------------------------------------------------------------------------------------------------------------------------------------------------------------------------------------------------------------------------------------------------------------------------------------------------------------------------------------------------------------------------------------------------------------------------------------------------------------------------------------------------------------------------------------------------------------------------------------------------------------------------------------------------------------------------------------------------------------------------------------------------------------------------------------------------------------------------------------------------------------------------------------------------------------------------------------------------------------------------------------------------------------------------------------------------------------------------------------------------------------------------------------------------------------------------------------------------------------------------------------------------------------------------------------------------------------------------------------------------------------------------------------------------------------------------------------------------------------------------------------------------------------------------------------------------------------------------------------------------------------------------------------------------------------------------------------------------------------------------------------------------------------------------------------------------------------------------------------------------------------------------------------------------------------------------------------------------------------------------------

>F1111951_Laccaria_trichodermophora_COSTA_RICA_KU685640_KU685784_KU686063_
[truncated: 159,680 more chars]
